# Preso enhances mGluR1-mediated excitotoxicity by modulating the phosphorylation of mGluR1-Homer1 complex and facilitating an ER stress after traumatic brain injury

**DOI:** 10.1038/s41420-024-01916-5

**Published:** 2024-03-26

**Authors:** Zhuoyuan Zhang, Xiangyu Gao, Zhicheng Tian, Erwan Yang, Yutao Huang, Dan Liu, Shuhui Dai, Haofuzi Zhang, Mingdong Bao, Xiaofan Jiang, Xin Li, Peng Luo

**Affiliations:** 1 Department of Neurosurgery, Xijing Hospital, Fourth Military Medical University, Xi’an, China; 2 School of Life Science, Northwest University, Xi’an, China; 3 Department of Anesthesiology, Xijing Hospital, Fourth Military Medical University, Xi’an, China

**Keywords:** Brain injuries, Trauma, Molecular neuroscience

## Abstract

Glutamate receptor (GluR)-mediated excitotoxicity is an important mechanism causing delayed neuronal injury after traumatic brain injury (TBI). Preso, as a core scaffolding protein of postsynaptic density (PSD), is considered an important regulator during excitotoxicity and TBI and combines with glutamate receptors to form functional units for excitatory glutamatergic neurotransmission, and elucidating the mechanisms of these functional units will provide new targets for the treatment of TBI. As a multidomain scaffolding protein, Preso directly interacts with metabotropic GluR (mGluR) and another scaffold protein, Homer. Because the mGluR-Homer complex plays a crucial role in TBI, modulation of this complex by Preso may be an important mechanism affecting the excitotoxic damage to neurons after TBI. Here, we demonstrate that Preso facilitates the interaction between metabotropic mGluR1 and Homer1 to activate mGluR1 signaling and cause excitotoxic neuronal injury and endoplasmic reticulum (ER) stress after TBI. The regulatory effect of Preso on the mGluR1-Homer1 complex is dependent on the direct association between Preso and this complex and also involves the phosphorylation of the interactive binding sites of mGluR1 and Homer1 by Preso. Further studies confirmed that Preso, as an adaptor of cyclin-dependent kinase 5 (CDK5), promotes the phosphorylation of the Homer1-binding site on mGluR1 by CDK5 and thereby enhances the interaction between mGluR1 and Homer1. Preso can also promote the formation of the mGluR1-Homer1 complex by inhibiting the phosphorylation of the Homer1 hinge region by Ca^2+^/calmodulin-dependent protein kinase IIα (CaMKIIα). Based on these molecular mechanisms, we designed several blocking peptides targeting the interaction between Preso and the mGluR1-Homer1 complex and found that directly disrupting the association between mGluR1 and scaffolding proteins significantly promotes the recovery of motor function after TBI.

## Introduction

According to the report by the World Health Organization on the global disease burden, trauma is the primary cause of death or loss of normal life activity [1]. Among the various traumatic diseases, traumatic brain injury (TBI) has the highest disability and mortality rates and places a great burden on the injured individual, their family, and society [2]. Many studies have demonstrated that neuronal excitotoxicity caused by TBI is a crucial mechanism leading to secondary brain damage and is associated with the abnormal functioning of glutamate receptors (GluRs) in excitatory synapses [3, 4]. Although data from animal experiments have confirmed that pharmacological antagonists of GluRs, including N-methyl-D-aspartate receptors (NMDARs) and metabotropic GluRs (mGluRs), can significantly reduce excitotoxic injury after TBI, these drugs did not achieve optimal results in a series of clinical trials [5, 6]. Therefore, it is crucial to clarify the pathological mechanism of excitotoxic injury following TBI and discover new therapeutic targets.

GluRs are components of the postsynaptic density (PSD) and engage with postsynaptic scaffolding proteins to regulate downstream signaling [7]. Recent studies found that disrupting the interaction between NMDARs and their scaffold protein, postsynaptic density-95 (PSD-95), alleviates TBI-induced excitotoxicity, indicating that interfering with the interaction between GluRs and scaffold proteins is a potential therapeutic strategy [8, 9]. As a crucial group of postsynaptic scaffold proteins, Homer proteins mainly bind with group I mGluRs to form an mGluR-Homer complex, which participates in the regulation of neuronal synaptic function [10, 11]. Inhibiting the activity of mGluR1 or interfering with the expression of Homer1 can reduce TBI-induced neuronal excitotoxicity [12, 13]. The overexpression of Homer1a (the short variant of Homer1), which competitively binds mGluR1 and prevents the mGluR1-Homer1 interaction, inhibits excitotoxic neuronal damage and improves neurological recovery following TBI [14]. Therefore, the mGluR1-Homer1 interaction is a novel target for regulating mGluR1-related excitotoxic neuronal injury after TBI.

Preso (PSD-95-interacting regulator of spine morphogenesis or Preso1) is a scaffolding protein comprising WW, PDZ and FERM domains at the N terminus, a Homer-binding motif (HBM) and a PDZ-binding motif (PBM) at the C terminus, and is therefore also called FERM and PDZ domain containing 4 (FRMPD4) [15, 16]. Preso facilitates NMDAR signaling during glutamate-induced excitotoxicity by interacting with PSD-95 [17]. Research has indicated that Preso enhances the NMDAR and PSD-95 interaction via its C-terminal PBM, thereby inducing TBI-related neurotoxicity [9]. These findings indicate that Preso acts as an important regulator of the interaction between GluRs and scaffold proteins during excitotoxicity and after TBI.

In addition to NMDAR, Preso regulates mGluR function, including mGluR-associated excitotoxicity [17, 18]. Remarkably, Preso binds to group I mGluRs through its FERM domain and also couples with Homer1 via its HBM [16]. Through these interactions, Preso coordinates the coupling of mGluR5 with Homer1 and thus promotes mGluR5-mediated pain signaling [18]. Similarly, Preso modulates the interaction between mGluR1 and Homer1 via a dynamic mechanism [19]. Considering the important role of the mGluR1-Homer1 complex in excitotoxicity, it is possible that the Preso-mediated mGluR1-Homer1 interaction is also involved in regulating neuronal injury after TBI.

Protein kinase-dependent phosphorylation of the binding site between GluRs and scaffold proteins is an important endogenous molecular mechanism that regulates the interaction between GluRs and scaffold proteins [20]. Under the action of cyclin-dependent kinase 5 (CDK5), group I mGluRs can be phosphorylated at their Homer-binding sites and thereby promote the interaction between mGluR and Homer [21, 22]. In contrast, Ca^2+^/calmodulin-dependent protein kinase IIα (CaMKIIα) is involved in phosphorylating Homer proteins at their mGluR-binding sites, which inhibits the formation of the mGluR-Homer complex [23, 24]. Preso has a predicted D-domain, which structurally functions as a binding site for proline-directed kinases, including CDK5 [9, 18, 25]. Preso reportedly interacts with CDK5 and thereby maintains the stability of the mGluR-Homer complex [18, 19]. In accordance with these regulatory mechanisms, the modulation of CDK5 and CaMKIIα by Preso might have a critical role in regulating the mGluR1-Homer1 interaction in the pathogenesis of TBI.

In this study, we found that Preso promoted the mGluR1-Homer1 interaction after TBI, leading to neurotoxicity. This effect was dependent on the role of Preso in differentially regulating the phosphorylation of mGluR1- and Homer1-binding sites via modulation of CDK5 and CaMKIIα activity. Therefore, our work presents a new theoretical basis for the pathogenesis of excitotoxicity after TBI and provides several new targets for the development of clinical drugs for TBI.

## Results

### Preso activates mGluR1 via its FERM domain after traumatic neuronal injury

**Fig. 1:**
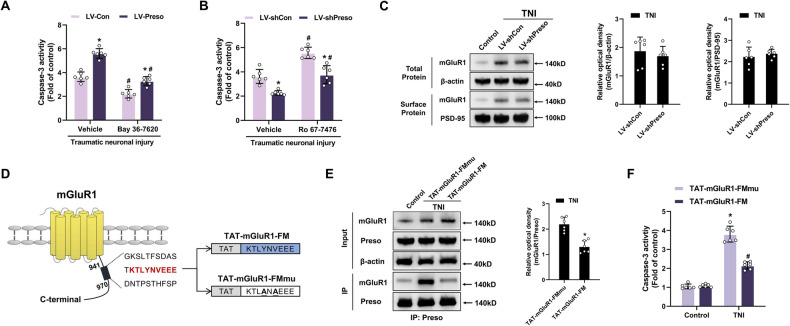
Preso regulates mGluR1-mediated excitotoxicity after TNI. **A** The inhibition of mGluR1 reduced neuronal injury induced by upregulation of Preso. After transfection with different lentiviral vectors (LV-Con or LV-Preso) and pretreatment with mGluR1 antagonist (Bay 36-7620, 10 μM) or vehicle (DMSO), neuronal apoptosis was measured by the caspase-3 activity assay at 24 h after TNI. The data are presented as the means ± SDs of six biological repeats. *p < 0.05 *vs*. the LV-Con group; ^#^*p* < 0.05 *vs*^.^ the vehicle group. **B** The downregulation of Preso attenuated the overactivation of mGluR1-associated excitotoxicity. After transfection with different lentiviral vectors (LV-shCon or LV-shPreso) and pretreatment with mGluR1 antagonist (Ro 67-7476, 1 μM) or vehicle, neuronal apoptosis was measured by the caspase-3 activity assay at 24 h after TNI. The data are presented as the means ± SDs of six biological repeats. *p < 0.05 *vs*. the LV-Con group; ^#^*p* < 0.05 *vs*. the vehicle group. **C** Preso does not affect the surface expression of mGluR1 after TNI. After transfection with LV-shCon or LV-shPreso, the expression levels of mGluR1 and surface mGluR1 were analyzed by western blotting at 24 h after TNI. The data are presented as the means ± SDs of six biological repeats. **D** Schematic diagram of the structure of mGluR1 and TAT-fused peptides. A predicted binding site for the Preso FERM domain in the C-terminus of mGluR1 is shown in red. This site was used as a target sequence of TAT-mGluR1-FM to disrupt the interaction between Preso and mGluR1. The sites in the target sequence that were mutated to generate TAT-mGluR1-FMmu, which was designed as the control for TAT-mGluR1-FM, are bolded and underlined. **E** TAT-mGluR1-FM disrupted the interaction between Preso and mGluR1. After pretreatment with TAT-mGluR-FM (5 μM) or TAT-mGluR1-FMmu (5 μM), immunoprecipitation with an anti-Preso antibody was performed following TNI, and the expression of mGluR1 and Preso was analyzed by western blotting. The data are presented as the means ± SDs of six biological repeats. *p < 0.05 *vs*. the TAT-mGluR1-FMmu group. **F** Disruption of the Preso-mGluR1 interaction by TAT-mGluR-FM reduced excitotoxicity. After pretreatment with TAT-mGluR-FM or TAT-mGluR1-FMmu, neuronal apoptosis was determined following TNI. The data are presented as the means ± SDs of six biological repeats. *p < 0.05 *vs*. the TAT-mGluR1-FMmu control group.

Accumulating evidence suggests that group I mGluRs have a significant impact on the pathogenesis of TBI [14, 26]. To investigate the relationship between Preso and group I mGluRs in traumatic injury, we used a lentivirus expressing Preso-targeting shRNA (LV-shPreso) or Preso (LV-Preso) to modulate the expression of Preso in neuronal primary cultures and a transection model, which has been used as an in vitro model for assessing traumatic neuronal injury (TNI). Pretreatment with antagonists of mGluR1 (Bay 36-7620 and LY367385) prevented the neurotoxic effects of Preso upregulation after TNI (Fig. 1A, S1A-C), demonstrating that the regulatory effect of Preso is closely related to the activation of mGluR1. Next, positive allosteric modulators (PAMs) of mGluR1 (Ro 67-7476 and Ro 0711401) were used to elevate the sensitivity of mGluR1 in neurons. The downregulation of Preso expression in neurons significantly reduced the PAM-induced exacerbation of neuronal injury after TNI (Fig. 1B, S1D-F), demostrating that Preso is associated with the preservation of mGluR1 activation. However, the downregulation of Preso expression in neurons did not have a notable impact on either the surface or total expression of mGluR1 after TNI (Fig. 1C). These results suggest that Preso modulates the function of mGluR1 via a mechanism other than influencing group I mGluR expression and trafficking.

Because Preso has a binding site for group I mGluRs in its FERM domain [16], the interaction between Preso and group I mGluRs is significantly suppressed after deletion of the FERM domain [18, 19]. Furthermore, the mGluR1 C-terminus has a predicted binding site for the FERM domain [27]. To disrupt the interaction between Preso and mGluR1, we constructed a peptide containing a cell-penetrating peptide (TAT) and amino acids 952-960 of mGluR1, which we named TAT-mGluR1-FM. Because multiple point mutations in the FERM-binding site markedly abolish its ability to bind the FERM domain [18], a version of TAT-mGluR1-FM with Y955A and V957A mutations (TAT-mGluR1-FMmu) was designed as a nonbinding control (Fig. 1D). Compared with TAT-mGluR1-FMmu, TAT-mGluR1-FM disrupted the interaction between Preso and mGluR1 in neurons (Fig. 1E). As expected, disrupting the Preso-mGluR1 interaction by interfering with the FERM domain-binding site attenuated TNI-induced neuronal cell death (Fig. 1F, S1G). These results indicate that the binding of Preso to mGluR1 through the FERM domain is key for its ability to activate neuronal mGluR1 after traumatic injury.

### Enhancement of the mGluR1-Homer1 interaction by Preso contributes to neurotoxicity after traumatic injury

**Fig. 2:**
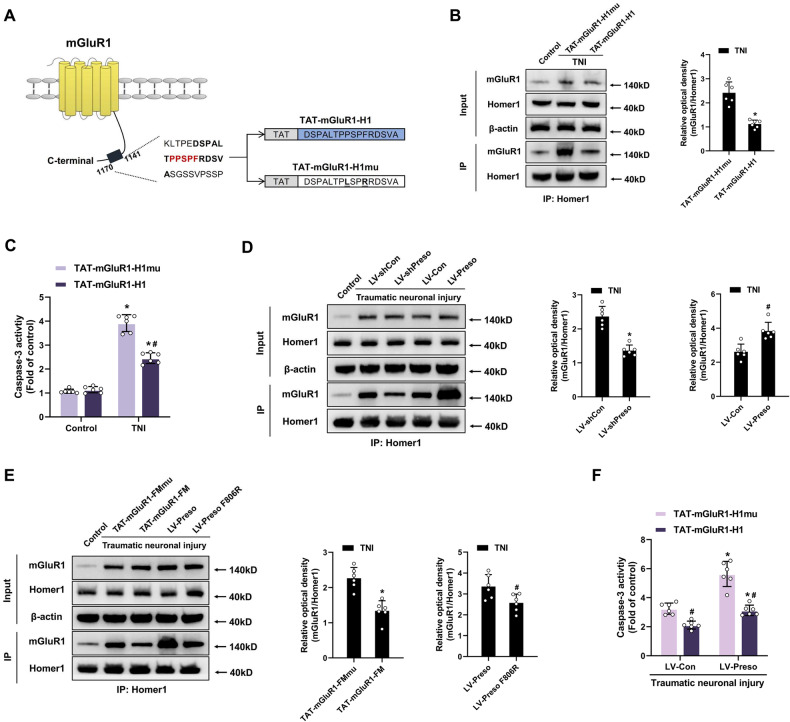
Preso promotes the mGluR1-Homer1 interaction and related excitotoxicity after TNI. **A** Schematic diagram of the structure of mGluR1 and TAT-fused peptides. The target sequence of TAT-mGluR1-H1 is shown in bold, and the HBM in the C-terminus of mGluR1 is shown in red. TAT-mGluR1-H1 was designed to disrupt the interaction between mGluR1 and Homer1. The sites in the target sequence that were mutated to generate TAT-mGluR1-H1mu are bolded and underlined. TAT-mGluR1-H1mu was designed as the control for TAT-mGluR1-H1. **B** TAT-mGluR1-H1 disrupted the interaction between mGluR1 and Homer1. After pretreatment with TAT-mGluR-H1 (5 μM) or TAT-mGluR1-H1mu (5 μM), immunoprecipitation with an anti-Homer1 antibody was performed following TNI, and the expression of mGluR1 and Homer1 was analyzed by western blotting. The data are presented as the means ± SDs of six biological repeats. *p < 0.05 *vs.* the TAT-mGluR1-H1mu control group. **C** Disruption of the mGluR1-Homer1 interaction by TAT-mGluR-H1 reduced excitotoxicity. After pretreatment with TAT-mGluR-H1 or TAT-mGluR1-H1mu, neuronal apoptosis was determined following TNI. The data are presented as the means ± SDs of six biological repeats. *p < 0.05 *vs*. the TAT-mGluR1-H1mu control group. **D** Preso positively regulated mGluR1-Homer1 complex formation. After transfection with LV-shCon, LV-shPreso, LV-Con, or LV-Preso, immunoprecipitation with an anti-Homer1 antibody was performed following TNI, and the expression of mGluR1 and Preso was analyzed by western blotting. The data are presented as the means ± SDs of six biological repeats. *p < 0.05 *vs.* the LV-shCon group; ^#^*p* < 0.05 *vs*. the LV-Con group. **E** Blocking the association between Preso and the mGluR1-Homer1 complex inhibited the effect of Preso on the mGluR1-Homer1 interaction. After pretreatment with different peptides (TAT-mGluR-FM or TAT-mGluR1-FMmu) or transfection with different lentiviral vectors (LV-Preso or LV-Preso F806R), immunoprecipitation with an anti-Homer1 antibody was performed following TNI, and the expression of mGluR1 and Homer1 was analyzed by western blotting. The data are presented as the means ± SDs of six biological repeats. *p < 0.05 *vs.* the TAT-mGluR1-FMmu control group; ^#^*p* < 0.05 *vs*. the LV-Preso group. **F** Disruption of the mGluR1-Homer1 complex suppressed the excitotoxicity induced by Preso upregulation. After transfection with LV-Con or LV-Preso, the neurons were pretreated with TAT-mGluR-H1 or TAT-mGluR1-H1mu, and neuronal apoptosis was determined following TNI. The data are presented as the means ± SDs of six biological repeats. *p < 0.05 *vs.* the LV-Con group; ^#^*p* < 0.05 *vs*. the TAT-mGluR1-H1mu control group.

Homer1 regulates group I mGluR signaling by directly interacting with mGluR [28, 29]. Here, it was discovered that the interaction between mGluR1 and Homer1 was significantly increased by traumatic injury (Fig. S2). Subsequently, we determined whether enhancement of the mGluR1-Homer1 interaction contributed to neuronal cell death following TNI. We constructed a peptide containing the Homer-binding site of mGluR1 to interfere with the mGluR1-Homer1 interaction. The peptide was named TAT-mGluR1-H1 and was composed of TAT and amino acids 1146-1161 of the C-terminus of mGluR1. A version of TAT-mGluR1-H1 with mutations in the Homer-binding site (P1153L and F1156R) (TAT-mGluR1-H1mu) was designed as a nonbinding control (Fig. 2A). Compared with TAT-mGluR1-H1mu, TAT-mGluR1-H1 selectively inhibited the interaction between mGluR1 and Homer1 after TNI (Fig. 2B). Furthermore, blockade of the mGluR1-Homer1 interaction reduced neuronal injury and cell death after TNI (Fig. 2C, S3A). These results suggest that TNI increases the mGluR1-Homer1 interaction and that this interaction leads to neuronal cell death induced by traumatic injury.

Previous studies have reported that Preso acts as a scaffolding protein to facilitate the interaction between group I mGluRs and Homer1 [18, 19]. Therefore, we conducted further investigations into the role of Preso in regulating the mGluR1-Homer1 complex following traumatic injury. Both under normal conditions and after TNI, downregulation of Preso expression induced dissociation of the mGluR1-Homer1 complex, whereas upregulation of Preso expression increased the interaction between mGluR1 and Homer1 (Fig. 2D, S4A). In the above-described study, we confirmed that the binding of Preso to mGluR1 via the FERM domain is an important mechanism for the activation of mGluR1 after TNI. Based on this finding, we further disrupted the Preso-mGluR1 interaction using TAT-mGluR1-FM and found that TAT-mGluR1-FM inhibited the formation of the mGluR1-Homer1 complex after TNI (Fig. 2F). Notably, Preso also has a Homer-binding site [16, 18]. Hence, we constructed a lentiviral vector expressing Preso with a point mutation in the Homer-binding site (F806R) (LV-Preso F806R). Mutation of the Homer-binding site of Preso also affected its ability to enhance the mGluR1-Homer1 interaction (Fig. 2E, S4B). These results indicated that Preso promoted the interaction between mGluR1 and Homer1 by binding to these proteins. We further examined the role of the mGluR1-Homer1 interaction in modulating Preso-mediated neurotoxicity after traumatic injury. Blocking the interaction of mGluR1 with Homer1 via TAT-mGluR1-H1 suppressed the exacerbation of neuronal injury induced by Preso upregulation after TNI (Fig. 2F, S3B). These results indicate that promoting the formation of the mGluR1-Homer1 complex is an important mechanism by which Preso causes neuronal damage after traumatic injury.

### Preso mediates a dynamic mGluR1-Homer1 interaction by regulating the phosphorylation of its interacting binding sites

**Fig. 3:**
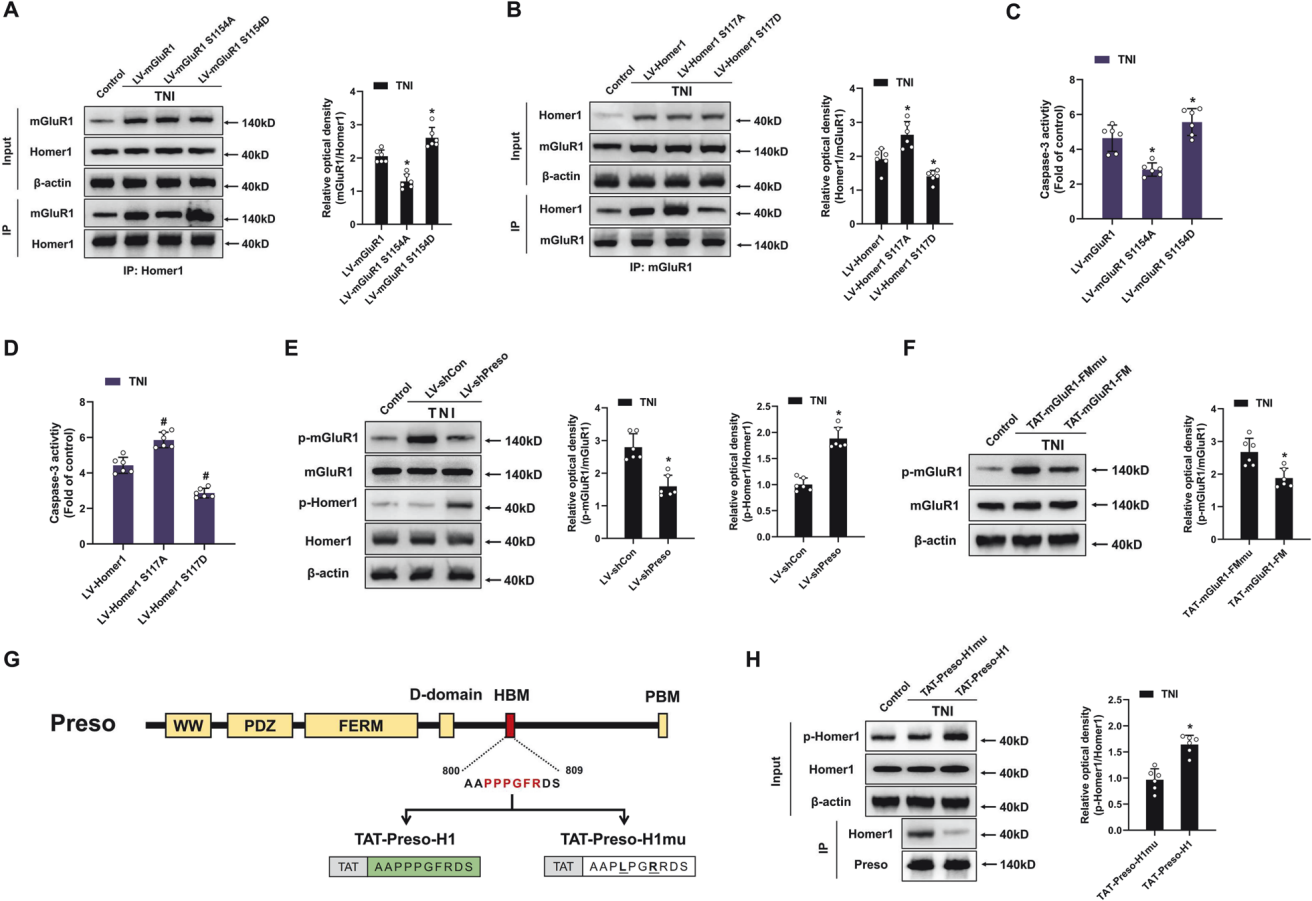
Preso dynamically modulates the phosphorylation of mGluR1 and Homer1 after TNI. **A** Phosphorylation of the Homer-binding site of mGluR1 at S1154 increased the interaction between mGluR1 and Homer1. After transfection with LV-mGluR1, LV-mGluR1 S1154A, or LV-mGluR1 S1154D, the expression of mGluR1 and Homer1 was analyzed by western blotting at 24 h after TNI. The data are presented as the means ± SDs of six biological repeats. *p < 0.05 *vs*. the LV-mGluR1 group. **B** Phosphorylation of the hinge region of Homer1 at S117 reduced the interaction between mGluR1 and Homer1. After transfection with LV-Homer1, LV-Homer1 S117A, or LV-Homer1 S117D, immunoprecipitation with an anti-Homer1 antibody was performed following TNI, and the expression of mGluR1 and Homer1 was analyzed by western blotting. The data are presented as the means ± SDs of six biological repeats. *p < 0.05 *vs*. the LV-Homer1 group. **C**, **D** The phosphorylation of mGluR1 and Homer1 bidirectionally regulates excitotoxicity after TNI. After transfection with LV-mGluR1, LV-mGluR1 S1154A, LV-mGluR1 S1154D, LV-Homer1, LV-Homer1 S117A, or LV-Homer1 S117D, neuronal apoptosis was determined at 24 h after TNI. The data are presented as the means ± SDs of six biological repeats. *p < 0.05 *vs*. the LV-mGluR1 group; ^#^*p* < 0.05 *vs*. the LV-Homer1 group.^.^ **E** The downregulation of Preso expression induced hypophosphorylation of mGluR1 and hyperphosphorylation of Homer1. After transfection with LV-shCon or LV-shPreso, the phosphorylation of mGluR1 at S1154 and the phosphorylation of Homer1 at S117 were analyzed by western blotting following TNI. The data are presented as the means ± SDs of six biological repeats. *p < 0.05 *vs*. the LV-shCon group. **F** Disruption of the Preso-mGluR1 interaction inhibited the phosphorylation of mGluR1. After pretreatment with TAT-mGluR1-FM or TAT-mGluR1-FMmu, the phosphorylation of mGluR1 at S1154 was analyzed by western blotting following TNI. The data are presented as the means ± SDs of six biological repeats. *p < 0.05 *vs*. the TAT-mGluR1-FMmu control group. **G** Schematic diagram of the structure of Preso and TAT-fused peptides. The target sequence of TAT-Preso-H1 is shown in bold, and the HBM of Preso is shown in red. TAT-Preso-H1 was designed to disrupt the interaction between Preso and Homer1. The sites in the target sequence that were mutated to generate TAT-Preso-H1mu are bolded and underlined. TAT-Preso-H1mu was designed as the control for TAT-Preso-H1. **H** Disruption of the Preso-Homer1 interaction increased the phosphorylation of Homer1. After pretreatment with TAT-Preso-H1 (5 μM) or TAT-Preso-H1mu (5 μM), immunoprecipitation with an anti-Preso antibody was performed following TNI, and the phosphorylation of Homer1 at S117 and the expression of Preso and Homer1 were analyzed by western blotting. The data are presented as the means ± SDs of six biological repeats. *p < 0.05 *vs*. the TAT-Preso-H1mu control group.

Because the mGluR-Homer interaction is regulated by the phosphorylation of binding sites in mGluR and Homer, we hypothesized that traumatic injury might increase the mGluR1-Homer1 interaction by modulating the phosphorylation of binding sites responsible for the formation of the mGluR1-Homer1 complex. Traumatic injury induced the phosphorylation of mGluR1 at its Homer-binding site (S1154) but did not affect the phosphorylation of Homer1 at the hinge region (S117) (Fig. S5). We then constructed an mGluR1-expressing lentivirus (LV-mGluR1), a lentiviral vector expressing mGluR1 with a dephosphomimetic mutation in the Homer-binding site (LV-mGluR1 S1154A), and a lentiviral vector expressing mGluR1 with a phosphomimetic mutation of the Homer-binding site (LV-mGluR1 S1154D). Compared with wild-type vectors, the dephosphomimetic mutation-expressing vector attenuated the mGluR1-Homer1 interaction, whereas the phosphomimetic mutation-expressing vectors promoted the formation of the mGluR1-Homer1 complex (Fig. 3A). Although traumatic injury did not significantly affect the phosphorylation of Homer1, we wondered about the potential role of Homer1 phosphorylation in TNI and constructed lentiviral vectors expressing wild-type Homer1 (LV-Homer1), Homer1 with a dephosphomimetic mutation (LV-Homer1 S117A), and Homer1 with a phosphomimetic mutation (LV-Homer1 S117D). Both Homer1 hypophosphorylation and Homer1 hyperphosphorylation affected the mGluR1-Homer1 interaction in manners opposite to those observed with the mGluR1 mutants (Fig. 3B). Furthermore, hyperphosphorylation of mGluR1 and hypophosphorylation of Homer1 aggravated neuronal injury after TNI, whereas mGluR1 with a dephosphomimetic mutation and Homer1 with a phosphomimetic mutation exerted the opposite effects (Fig. 3C, D, S6). These results demonstrate that phosphorylation of interacting binding sites of mGluR1 and Homer1 bidirectionally regulates the mGluR1-Homer1 interaction and its function after traumatic injury.

Subsequently, we verified whether Preso was involved in the regulatory effects of phosphorylation of the interacting binding sites of mGluR1 and Homer1. Both under normal conditions and after TNI, inhibition of Preso expression suppressed the phosphorylation of mGluR1 at its Homer-binding site and elevated the phosphorylation of Homer1 at its mGluR1-binding site (Fig. 3E, S7). Because the abovementioned experiments showed that phosphorylation of these binding sites is important for regulating the mGluR1-Homer1 interaction, this result suggests that Preso’s effect of increasing the mGluR1-Homer interaction was related to its differential regulation of the phosphorylation of interacting binding sites of mGluR1 and Homer1. Based on the mechanism underlying the regulation of the mGluR1-Homer1 interaction by Preso, we further hypothesized that the regulatory effect of Preso on binding site phosphorylation was dependent on its direct interactions with the mGluR1-Homer1 complex. To confirm this finding, we blocked the Preso-mGluR1 interaction using TAT-mGluR1-FM and found that it reduced the effects of Preso on mGluR1 phosphorylation (Fig. 3F). We then constructed a blocking peptide containing TAT and the Homer-binding site of Preso (amino acids 800-809), which was named TAT-Preso-H1, and a control peptide consisting of TAT-Preso-H1 with P803L and F806R mutations, which was named TAT-Preso-H1mu (Fig. 3G). TAT-Preso-H1 induced the dissociation of Preso from Homer1 and increased the phosphorylation of the mGluR1-binding site of Homer1 (Fig. 3H). These data suggest that the Preso-dependent regulation of mGluR1 and Homer1 phosphorylation requires the binding of Preso to mGluR1 and Homer1.

### The CDK5-mediated phosphorylation of mGluR1 is involved in the regulatory effect of Preso on the mGluR1-Homer1 interaction

**Fig. 4:**
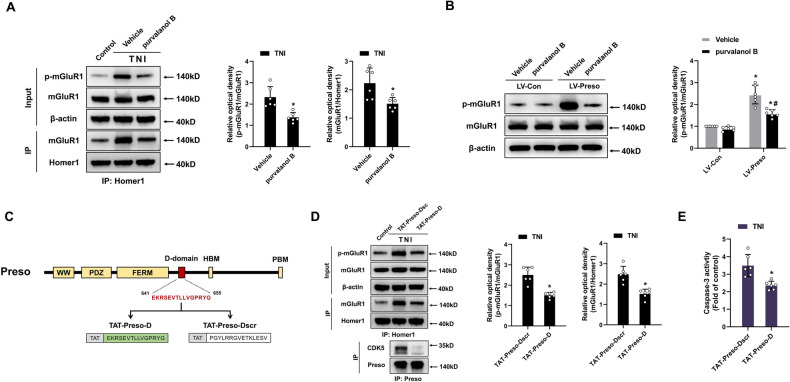
Preso enhances mGluR1 phosphorylation by interacting with CDK5 after TNI. **A** Inhibition of CDK5 attenuated the phosphorylation of mGluR1 and the interaction between mGluR1 and Homer1. After pretreatment with purvalanol B (50 μM), a CDK5 inhibitor, or vehicle (DMSO), immunoprecipitation with an anti-Homer1 antibody was performed at 24 h after TNI, and the phosphorylation of mGluR1 at S1154 and the expression of mGluR1 and Homer1 were analyzed by western blotting. The data are presented as the means ± SDs of six biological repeats. *p < 0.05 *vs*. the vehicle group. **B** Inhibition of CDK5 reduced the phosphorylation of mGluR1 induced by Preso upregulation. After transfection with LV-Con and LV-Preso and pretreatment with purvalanol B (50 μM), the phosphorylation of mGluR1 at S1154 following TNI was analyzed by western blotting. The data are presented as the means ± SDs of six biological repeats. *p < 0.05 *vs*. the LV-Con group; ^#^*p* < 0.05 *vs*. the vehicle group. **C** Schematic diagram of the structure of Preso and TAT-fused peptides. The D-domain of Preso is shown in red. This domain was used as the target sequence for generating TAT-Preso-D to disrupt the interaction between Preso and CDK5. A scramble peptide (TAT-Preso-Dscr) containing the same amino acids in the D-domain in random order was constructed, and this peptide was designed as the control for TAT-Preso-D. **D**, **E** Disruption of the interaction between Preso and CDK5 inhibited the phosphorylation of mGluR1, blocked the interaction between mGluR1 and Homer1, and reduced excitotoxicity. After pretreatment with TAT-Preso-Dscr (5 μM) or TAT-Preso-D (5 μM), immunoprecipitation with an anti-Homer1 or anti-Preso antibody was performed, and the phosphorylation of mGluR1 at S1154 and the expression of mGluR1, Homer1, Preso, and CDK5 following TNI were analyzed by western blotting (**D**). Neuronal apoptosis was also examined (**E**). The data are presented as the means ± SDs of six biological repeats. *p < 0.05 *vs*. the TAT-Preso-Dscr control group.

Proline-directed kinases such as ERK and CDK5 phosphorylate the Homer binding site of group I mGluRs [18, 19]. Because our previous research reported that Preso mediates CDK5 activity after TBI [9], we hypothesized that the interaction between mGluR1 and Homer1 after traumatic injury is dependent on the regulatory effect of Preso on CDK5. The inhibition of CDK5 activity by purvalanol B, a CDK5 antagonist, attenuated the phosphorylation of mGluR1 at the Homer-binding site and disrupted the interaction between mGluR1 and Homer1 after TNI (Fig. 4A). Moreover, this antagonist suppressed the hyperphosphorylation of the Homer-binding site and the increase in mGluR1-Homer1 complex formation induced by upregulation of Preso expression (Fig. 4B). These results suggest that Preso modulates the mGluR1-Homer1 interaction via its regulation of CDK5-mediated mGluR1 phosphorylation.

Because Preso binds CDK5 via its D-domain [9], we constructed a blocking peptide targeting the D-domain to disrupt the interaction between Preso and CDK5 (Fig. 4C). This blocking peptide (TAT-Preso-D) was composed of TAT and the D-domain sequence of Preso (641-655 amino acids). A scramble peptide containing TAT and the amino acids of the D-domain in random order (TAT-Preso-Dscr) was also constructed. As expected, pretreatment with TAT-Preso-D inhibited the phosphorylation of the Homer-binding site, disrupted the mGluR1-Homer1 interaction, and reduced neuronal cell death after TNI (Fig. 4D, E, S8A). These results indicate that the binding of Preso to CDK5 through its D-domain is crucial for promoting the effect of CDK5 on the phosphorylation of mGluR1.

### Preso modulates the mGluR1-Homer1 interaction by negatively regulating CaMKIIα-dependent phosphorylation of Homer1

**Fig. 5:**
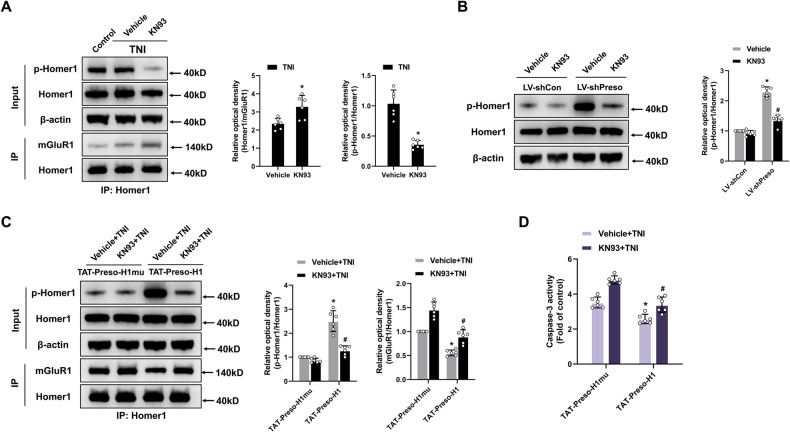
Preso inhibits Homer1 phosphorylation by blocking the effect of CaMKIIα after TNI. **A** Inactivation of CaMKIIα reduced the phosphorylation of Homer1, which promoted the interaction between mGluR1 and Homer1. After pretreatment with KN93 (10 μM), a CaMKIIα inhibitor, or vehicle (DMSO), immunoprecipitation with an anti-Homer1 antibody was performed at 24 h after TNI, and the phosphorylation of Homer1 at S117 and the expression of mGluR1 and Homer1 were analyzed by western blotting. The data are presented as the means ± SDs of six biological repeats. *p < 0.05 *vs*. the vehicle group. **B** Inactivation of CaMKIIα reduces the phosphorylation of Homer1 induced by Preso downregulation. After transfection with LV-shCon or LV-shPreso and pretreatment with KN93 or vehicle, the phosphorylation of Homer1 at S117 was analyzed by western blotting. The data are presented as the means ± SDs of six biological repeats. *p < 0.05 *vs*. the LV-shCon group; ^#^*p* < 0.05 *vs*. the vehicle group. **C**, **D** After disruption of the interaction between Preso and Homer1, inhibition of CaMKIIα attenuated the phosphorylation of Homer1, promoted the interaction between mGluR1 and Homer1, and increased excitotoxicity. After pretreatment with TAT-Preso-H1, TAT-Preso-H1mu, KN93, or vehicle, immunoprecipitation with an anti-Homer1 antibody was performed following TNI, and the phosphorylation of Homer1 at S117 and the expression of mGluR1 and Homer1 were analyzed by western blotting (**C**). Neuronal apoptosis was also examined (**D**). The data are presented as the means ± SDs of six biological repeats. *p < 0.05 *vs*. the TAT-Preso-H1mu control group; ^#^*p* < 0.05 *vs*. the Vehicle + TNI group.

CaMKIIα reportedly phosphorylates Homer proteins, reducing their interaction with their binding partners, such as group I mGluRs [24]. To ascertain if active CaMKIIα can reduce the mGluR1-Homer1 interaction following a traumatic injury, neurons were pretreated with a CaMKIIα antagonist (KN93). The inhibition of active CaMKIIα reduced the phosphorylation of Homer1 at the mGluR1-binding site and increased the interaction of Homer1 with mGluR1 after TNI (Fig. 5A). We then confirmed the involvement of CaMKIIα in regulating Homer1 phosphorylation mediated by Preso. Administration of the CaMKIIα antagonist reversed the effect of Preso downregulation on Homer1 phosphorylation and the disruption of the mGluR1-Homer1 interaction (Fig. 5B). These results indicate that CaMKIIα is associated with the Preso-mediated modulation of the mGluR1-Homer1 interaction by regulating the phosphorylation of Homer1.

As shown above, the interaction between Preso and Homer1 affects the formation of the mGluR1-Homer1 complex after traumatic injury. Therefore, we examined whether this Preso-Homer1 interaction also influences the effect of CaMKIIα on Homer1 phosphorylation. Disruption of the Preso-Homer1 interaction using TAT-Preso-H1 increased the phosphorylation of Homer1 and decreased the mGluR1-Homer1 interaction, and these effects were inhibited by the CaMKIIα antagonist (Fig. 5C). Furthermore, the CaMKIIα antagonist reduced the protective effects of TAT-Preso-H1 against neuronal cell death after TNI (Fig. 5D, S8B). These results demonstrate that the interplay between Preso and Homer influences the regulatory impact of CaMKIIα on Homer1 phosphorylation and the mGluR1-Homer1 complex.

### Preso induces neurotoxicity by facilitating mGluR1-Homer1 complex-mediated endoplasmic reticulum (ER) stress

**Fig. 6:**
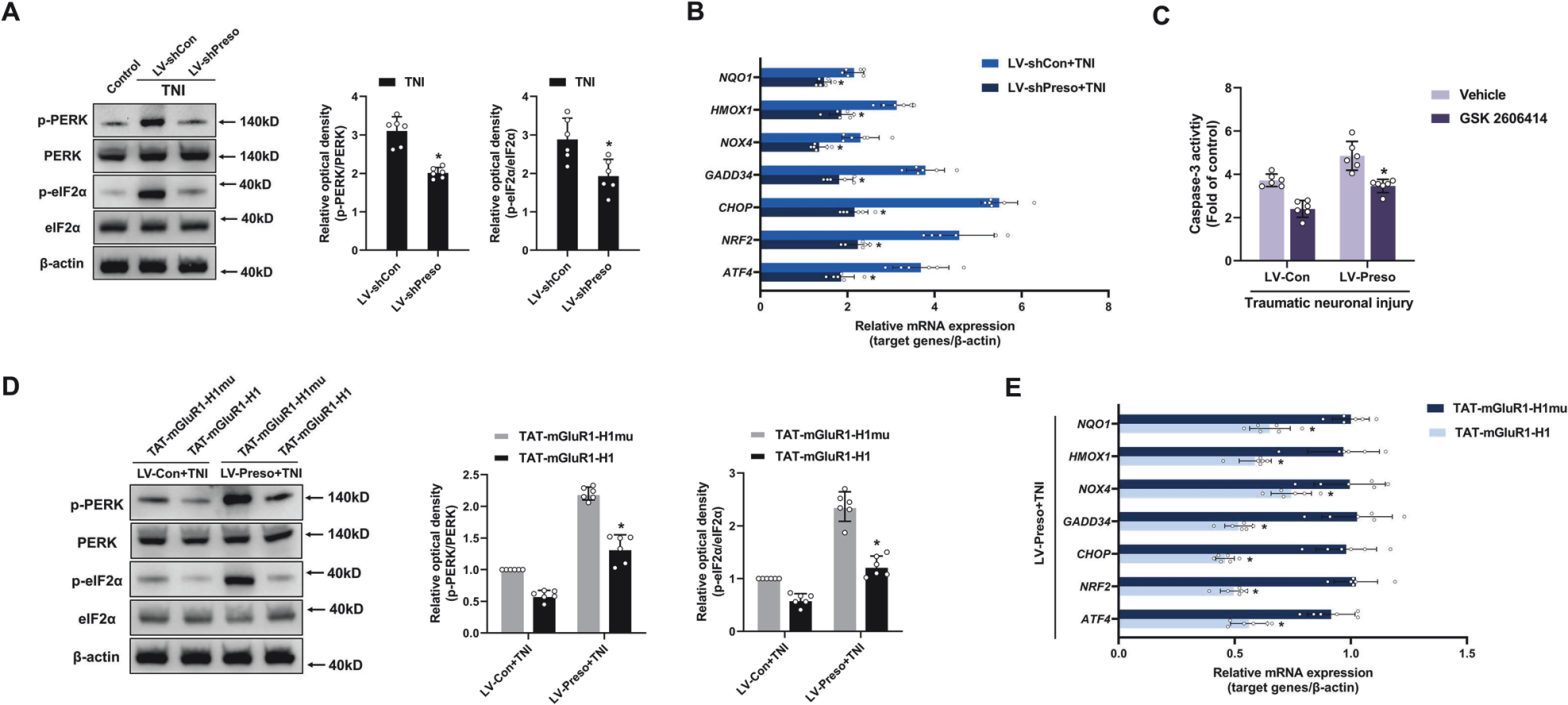
Preso activates ER stress via regulation of the mGluR1-Homer1 interaction after TNI. **A, B** Downregulation of Preso inhibited TNI-induced ER stress. After transfection with LV-shCon or LV-shPreso, the phosphorylation of PERK and eIF2α was analyzed by western blotting at 24 h after TNI (**A**), whereas the expression of ER stress target genes (*ATF4*, *NRF2*, *CHOP*, *GADD34*, *NOXA*, *HMOX1*, and *NQO1*) was examined by PCR (**B)**. The data are presented as the means ± SDs of six biological repeats. *p < 0.05 *vs*. the LV-shCon group or the LV-shCon + TNI group. **C** Inhibition of ER stress attenuated neuronal injury induced by Preso upregulation after TNI. After transfection with LV-Con or LV-Preso and pretreatment with GSK 2606414 (10 μM) or vehicle (DMSO), neuronal apoptosis following TNI was examined. The data are presented as the means ± SDs of six biological repeats. *p < 0.05 *vs*. the vehicle group. **D**, **E** Disruption of the mGluR1-Homer1 interaction reduced the enhancement of ER stress induced by Preso upregulation after TNI. After transfection with LV-Con or LV-Preso and pretreatment with TAT-mGluR1-H1 or TAT-mGluR1-H1mu, the phosphorylation of PERK and eIF2α was analyzed by western blotting (D), whereas the expression of ER stress target genes was examined by PCR (**E**). The data are presented as the means ± SDs of six biological repeats. *p < 0.05 *vs*. the TAT-mGluR1-H1mu control group.

Because Preso reportedly promotes intracellular Ca^2+^ overload after neuronal injury [9, 17], we speculated that Preso is also related to ER stress secondary to Ca^2+^ overload. To verify this speculation, we examined the role of Preso in regulating ER stress signaling after traumatic injury. Under normal conditions, the upregulation of Preso expression induced the phosphorylation of PRKR-like endoplasmic reticulum kinase (PERK) and eukaryotic translation initiation factor 2α (eIF2α) in neuronal cultures (Fig. S9). As expected, the downregulation of Preso expression blocked the phosphorylation of PERK and eIF2α and the expression of their target genes (*ATF4*, *NRF2*, *CHOP*, *GADD34*, *NOXA*, *HMOX1*, and *NQO1*) after TNI (Fig. 6A, B). In contrast, the increase in neurotoxicity induced by Preso upregulation was suppressed by antagonists of PERK (GSK 2606414) and eIF2α (Sal 003) (Fig. 6C, S10A-C). These results demonstrate that Preso contributes to the induction of ER stress after traumatic injury, which is responsible for neuronal cell death.

Because mGluR1 and Homer1 have previously been shown to be involved in the induction of ER stress [26, 30], we believe that Preso-induced ER stress might be associated with the mGluR1-Homer1 interaction. To test this hypothesis, we first investigated the role of the mGluR1-Homer1 complex in ER stress and found that disruption of the mGluR1-Homer1 interaction inhibited ER stress caused by traumatic injury (Fig. S11). This finding indicates that downregulation of Preso and blockade of mGluR1-Homer1 complex formation have similar effects on the regulation of ER stress after TNI. We subsequently determined whether Preso regulates ER stress by promoting the mGluR1-Homer1 interaction in injured neurons. In the presence of TAT-mGluR1-H1, the overactivation of ER stress signaling induced by Preso overexpression was reversed by disruption of the mGluR1-Homer1 complex (Fig. 6E, F). Based on these results, Preso appears to cause neurotoxicity by facilitating mGluR1-Homer1 complex-mediated ER stress induction after traumatic injury.

### Blocking the interaction between Preso and the mGluR1-Homer1 complex improves recovery after TBI

**Fig. 7:**
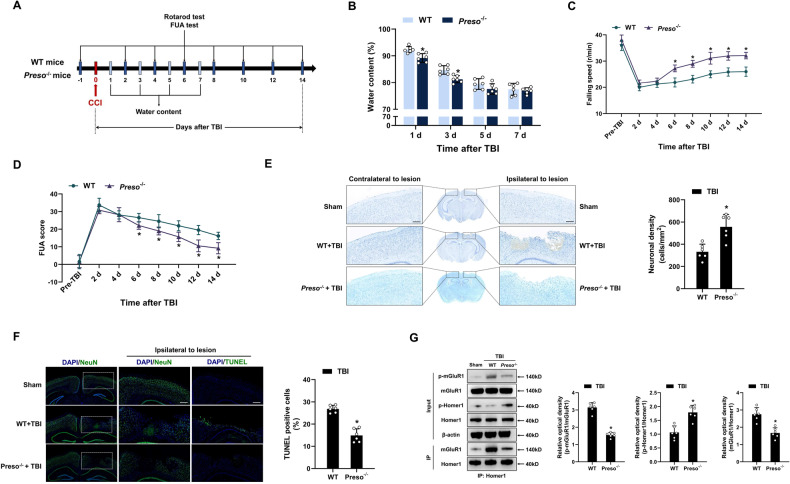
Improvement of neurological function recovery and disruption of the mGluR1-Homer1 complex in *Preso*^-/-^ mice after TBI. **A** Diagram showing the experimental design for the analysis of brain edema and motor function. The extent of brain edema was examined by measuring the brain water content at 1 d, 3 d, 5 d, and 7 d after TBI. Motor function was tested by the rotarod test and FUA test at 2 d, 4 d, 6 d, 8 d, 10 d, 12 d, and 14 d after TBI. **B** The extent of brain edema was reduced in *Preso*^-/-^ mice after TBI. The brain water content was analyzed at designated time points following TBI. The data are presented as the means ± SDs of six biological repeats. *p < 0.05 *vs*. WT mice. **C**, **D** Improvement in motor function in *Preso*^-/-^ mice after TBI. The rotarod test and FUA test were performed at designated time points following TBI. The data are presented as the means ± SDs of eight biological repeats. *p < 0.05 *vs*. the WT mice. **E, F** Reduction in neuronal loss and apoptosis in *Preso*^*-/-*^ mice after TBI. Nissl staining (**E**) and TUNEL staining (**F**) of brain tissues were performed at 14 days after TBI. Scale bar = 200 μm. The data are presented as the means ± SDs of six biological repeats. *p < 0.05 *vs*. the WT mice. **G** Disruption of the mGluR1-Homer1 interaction and bidirectional regulation of mGluR1 phosphorylation and Homer1 phosphorylation in *Preso*^-/-^ mice after TBI. After TBI, immunoprecipitation with an anti-Homer1 antibody was performed, and the phosphorylation of mGluR1 at S1154 and Homer1 at S117 and the expression of mGluR1 and Homer1 were analyzed by western blotting. The data are presented as the means ± SDs of six biological repeats. *p < 0.05 *vs*. WT mice.

To examine the role of Preso in regulating the mGluR1-Homer1 interaction after TBI, we generated Preso-knockout (*Preso*^-/-^) mice and developed a controlled cortical impact (CCI) model of TBI in vivo. The brain water content was measured at 1, 3, 5, and 7 d after TBI, and motor function, including performance on accelerating rotarod and forelimb use asymmetry (FUA) test, was assessed for 14 consecutive days at one-day intervals after injury (Fig. 7A). In agreement with the results of a previous study [9], *Preso*^-/-^ mice exhibited smaller brain edema at 1 d and 3 d after TBI. Although no significant difference between wild-type (WT) and *Preso*^-/-^ mice was found on accelerating rotarod and in the forelimb use asymmetry (FUA) test over the first 4 days after injury, the *Preso*^-/-^ mice exhibited significantly higher fall speeds on accelerating rotarod and significantly lower FUA scores, compared with WT mice at 6 d, 8 d, 10 d and 14 d after injury (Fig. 7B-D). Moreover, no significant differences in fall speeds and FUA scores were found between the WT and *Preso*^*-/-*^ mice in the sham group (Fig. S12). These results suggested better motor recovery in the Preso mice than in WT mice at 6 days after injury. Further histological staining after TBI showed that the degree of neuronal loss in the injured cortex and apoptosis in the peri-injury area were improved in the KO mice compared to the WT mice (Fig. 7E, F). In addition, disruption of the mGluR1-Homer1 interaction, reduction of mGluR1 phosphorylation, and elevation of Homer1 phosphorylation were observed in the cortex of *Preso*^*-/-*^ mice compared to WT mice in both the sham and TBI groups, and these results were consistent with previous in vitro experiments (Fig. 7G, S13).

**Fig. 8:**
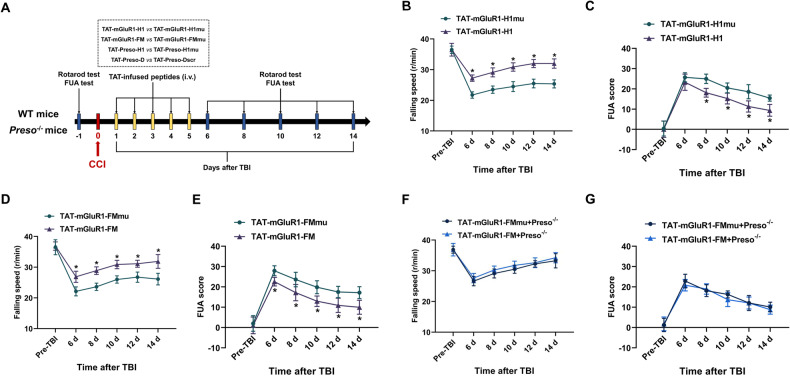
Application of TAT-fused peptides for the treatment of TBI. **A** Diagram showing the experimental design for the administration of TAT-fused peptides and motor function analysis. All peptides were administered via the tail vein (3 nmol/g weight/day) for five consecutive days after TBI. Motor function was tested by the rotarod test and FUA test at 6 d, 8 d, 10 d, 12 d, and 14 d after TBI. **B**, **C** TAT-mGluR1-H1 exerted a neuroprotective effect against TBI. After administration of TAT-mGluR1-H1 or TAT-mGluR1-H1mu, the rotarod test and FUA test were performed at designated time points following TBI. The data are presented as the means ± SDs of eight biological repeats. *p < 0.05 *vs*. the TAT-mGluR1-H1mu group. **D**, **E** TAT-mGluR1-FM exerted a neuroprotective effect against TBI. After administration of TAT-mGluR1-FM or TAT-mGluR1-FMmu, the rotarod test and FUA test were performed at designated time points following TBI. The data are presented as the means ± SDs of eight biological repeats. *p < 0.05 *vs*. the TAT-mGluR1-FMmu group. **F**, **G** TAT-mGluR1-FM exerted no significant effects on the recovery of motor function in *Preso*^-/-^ mice. After administration of TAT-mGluR1-FM or TAT-mGluR1-FMmu, the rotarod test and FUA test were performed at designated time points in *Preso*^-/-^ mice following TBI. The data are presented as the means ± SDs of eight biological repeats.

Based on the findings related to the mechanism by which Preso regulates the mGluR1-Homer1 complex, we hypothesized that the sites responsible for the mGluR1-Homer1 interaction might be potential therapeutic targets for TBI. To target these interaction sites, we constructed numerous blocking peptides, including TAT-mGluR1-H1, TAT-mGluR1-FM, TAT-Preso-H1, and TAT-Preso-D, and validated their effectiveness observed in previous in vitro experiments. On this basis, all peptides were administered via the tail vein for five consecutive days (3 nmol/g weight/day), and the motor functions of the mice were evaluated by the rotarod test and FUA test (Fig. 8A). TAT-mGluR1-H1 administration increased fall speeds in the rotarod test from 6 d to 14 d after injury and decreased FUA scores from 8 d to 14 d after injury. Similarly, the administration of TAT-mGluR1-FM increased fall speeds in the rotarod test and decreased FUA scores from 6 d to 14 d after injury (Fig. 8B, C). In contrast, the administration of TAT-Preso-H1 or TAT-Preso-D did not affect fall speeds in the rotarod test or FUA scores from 6 d to 14 d after injury (Figs. S5, 6). Furthermore, the effect of TAT-mGluR1-FM was not significant in the *P*reso^-/-^ mice (Fig. 8D, E). However, TAT-mGluR1-H1 significantly improved the motor function of *Preso*^-/-^ mice after TBI (Fig. S7). This finding showed that Preso exerts a relatively specific regulatory effect on the mGluR1-Homer1 interaction mediated by the fragment of mGluR1 contained in TAT-mGluR1-FM after TBI, whereas the mGluR1-Homer1 interaction mediated by the fragment of mGluR1 contained in TAT-mGluR1-H1 is regulated by another mechanism in vivo.

## Discussion

**Fig. 9:**
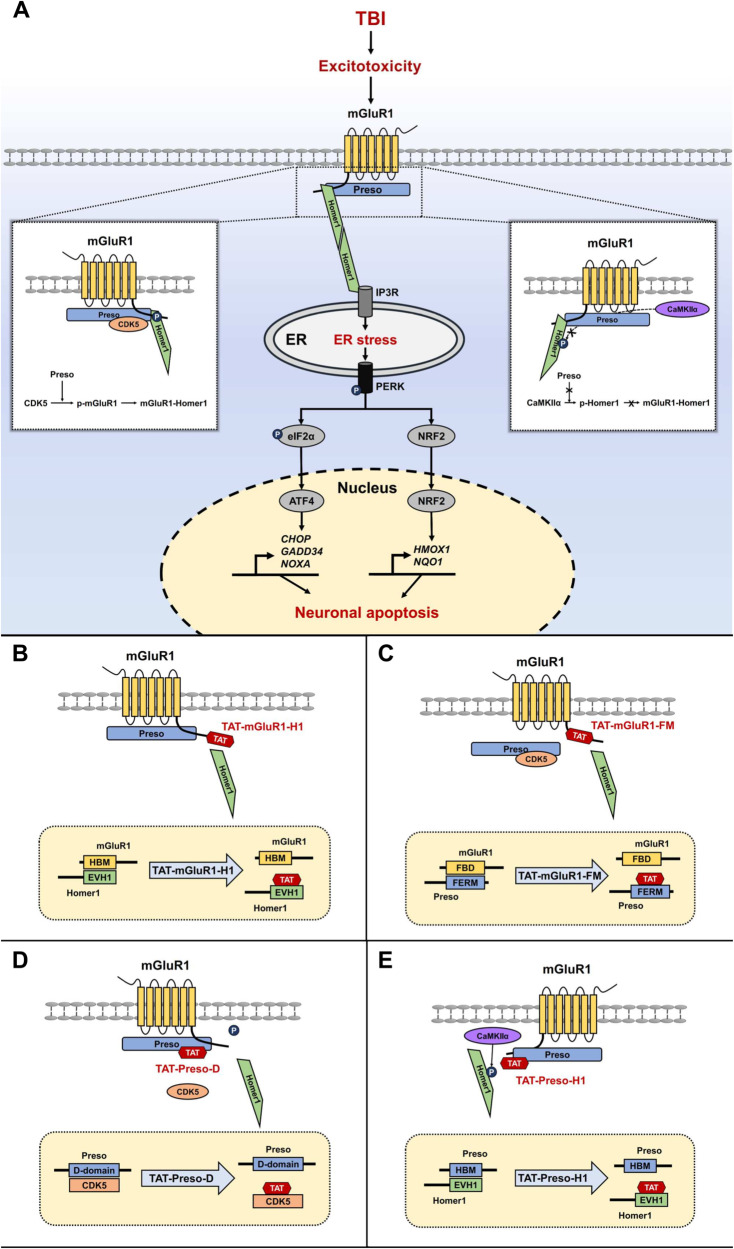
Diagram summarizing the various observations related to the regulation of the mGluR1-Homer1 interaction by Preso after TBI. **A** Schematic representation of the molecular mechanism underlying the Preso-mediated regulation of the mGluR1-Homer1 interaction after TBI. After TBI, Preso promotes the formation of the mGluR1-Homer1 complex by binding to CDK5 to increase the phosphorylation of the Homer1-binding site on mGluR1 and inhibiting CaMKII to reduce the phosphorylation of the mGluR1-binding site on Homer1, which enhances the activating effect of the mGluR1-Homer1 complex on ER stress and ultimately results in neuronal injury. **B**–**E** Mechanistic diagram of different TAT-fused peptides affecting protein‒protein interactions. TAT-mGluR1-H1 competitively binds to the EVH1 structural domain on Homer1 by carrying the HBM protein sequence from mGluR1 and thereby inhibits the interaction between mGluR1 and Homer1 (**B)**. TAT-mGluR1-FM competitively binds the FERM domain on Preso by carrying the FBD protein sequence from mGluR1 and thereby inhibits the interaction between mGluR1 and Preso (**C**). TAT-Preso-D competitively binds to the Preso binding site on CDK5 by carrying a D-domain protein sequence on Preso and thereby inhibits the interaction between Preso and CDK5 (**D**). TAT-Preso-H1 competitively binds the EVH1 domain on Homer1 by carrying the HBM protein sequence from Preso and thereby inhibits the interaction between Preso and CDK5 (**E**). Abbreviations: TBI, traumatic brain injury; mGluR1, metabotropic glutamate receptor 1; Preso, PSD-95-interacting regulator of spine morphogenesis; CDK5, cyclin-dependent kinase 5; CaMKIIα, Ca^2+^/calmodulin-dependent protein kinase IIα; Ca^2+^, calcium; ER, endoplasmic reticulum; PERK, PRKR-like endoplasmic reticulum kinase; eIF2α, eukaryotic translation initiation factor 2α; FBD, FERM-binding domain; HBM, Homer-binding motif.

The molecular mechanisms of neuronal injury and corresponding neuroprotective strategies are popular issues in the field of TBI research. In the present study, we confirmed that the Preso-induced mGluR1-Homer1 interaction is important for the activation of mGluR1 signaling and leads to neuronal injury after TBI. The regulatory effect of Preso on the mGluR1-Homer1 complex is dependent on structural interactions between Preso and this complex and is also closely related to the Preso-mediated modulation of mGluR1 and Homer1 phosphorylation at their interaction sites. Further studies confirmed that Preso promotes CDK5-mediated mGluR1 phosphorylation and that its inhibitory effect on CaMKIIα-mediated Homer1 phosphorylation is essential for the dynamic regulation of the mGluR1-Homer1 interaction. Based on these molecular mechanisms, we designed several blocking peptides targeting the interaction between Preso and the mGluR1-Homer1 complex and revealed that some of these peptides could exert neuroprotective effects against TBI (Fig. 9).

GluR-mediated excitotoxicity is one of the core mechanisms underlying secondary brain injury after TBI [31, 32]. The traditional opinion is that overactivation of ionotropic GluRs (iGluRs), represented by NMDARs, is the main contributor to neuronal injury after TBI and that mGluRs are mainly involved in modulating the effect of iGluRs to inhibit their overactivation [3, 33]. With continuous research on the mechanism of excitotoxicity, group I mGluRs have attracted extensive attention because these receptors, unlike other mGluRs, mainly promote excitotoxic neuronal damage [29, 34]. The specific blockade of mGluR1 can exert neuroprotective effects against TBI, further confirming its specific effects among mGluRs [12, 14, 26]. Therefore, an in-depth exploration of the mechanism of mGluR1 neurotoxicity in TBI is of great value for elucidating the molecular mechanism of excitotoxicity and identifying effective targets for intervention.

In addition to participating in classical ligand‒receptor interactions, GluRs are influenced by scaffold proteins that bind directly to them at postsynapses [31, 35]. Homer proteins, including Homer1-3, are the predominant scaffolding proteins that interact with mGluRs. These proteins directly interact with mGluRs and are further involved in mGluR-associated intracellular signaling through dimerization [36]. Among these Homer proteins, Homer1 is the major isoform that binds to mGluR1, forming the mGluR1-Homer1 complex [37]. Accumulating evidence suggests that the mGluR1-Homer1 complex is key for mGluR1 activation leading to excitotoxicity [30, 38, 39]. In contrast, blocking the formation of the mGluR1-Homer1 complex by downregulating Homer1 expression attenuates neuronal injury in a variety of excitotoxicity-associated neurological diseases, such as neurodegeneration and acute brain injury [13, 40, 41]. Interestingly, the mGluR1-Homer1 interaction can be disrupted by Homer1a, an endogenous short variant of the Homer1 protein. Our previous study showed that Homer1a protects neurons against TBI-induced excitotoxicity [14]. In the present study, we further confirmed that blocking the formation of the mGluR1-Homer1 complex by using an exogenous peptide inhibits neurotoxicity and improves neurological function after TBI. Taken together, these findings indicate that elucidating the mechanism by which the mGluR1-Homer1 interaction is regulated during excitotoxic neuronal injury is important for studying the mechanism of mGluR1 after TBI.

Preso is a scaffold protein with various binding domains and is involved in regulating dendritic spine morphogenesis [15, 42]. In our previous studies, we verified that Preso promoted glutamate-induced excitotoxicity by differentially modulating GluR signaling and facilitating the interaction between NR2B and PSD-95 through the binding of its C-terminus with PSD-95, which contributes to neurotoxicity after TBI [9, 17]. Remarkably, Preso has been shown to be a potential regulator of the mGluR-Homer interaction [18]. The present study showed that Preso promotes the interaction between mGluR1 and Homer1 after TBI. Structurally, Preso contains a FERM domain that binds to mGluR1 and a Homer-binding domain that binds to Homer1 [16]. Our study further confirmed that targeting these domains to prevent Preso from binding to mGluR1 and Homer1 significantly inhibits the promotion of mGluR1-Homer1 complex formation by Preso after TBI. According to this evidence, it is reasonable to speculate that the Preso-mediated promotion of mGluR1-Homer1 complex formation is another mechanism by which the mGluR1-Homer1 interaction is regulated after TBI. Based on the above findings, Preso is simultaneously involved in NMDAR signaling and mGluR1 signaling, which are the two different signaling pathways responsible for activating excitotoxicity, and these effects result in the more significant neuronal protective effect of Preso downregulation compared with that observed with the mGluR1 inhibitor. Furthermore, the findings reported thus far comprise only part of the regulatory mechanism of Preso in TBI. Other downstream mechanisms may exert neuroprotective effects after the downregulation of Preso, and these need to be explored and validated by further experiments.

In addition to the regulatory effect of endogenous Homer1a, phosphorylation of interacting binding sites is an alternative mechanism underlying the regulation of the mGluR-Homer complex structure and function [20]. Phosphorylation of the Homer-binding site of group I mGluRs enhances the mGluR-Homer interaction [21, 43]. In contrast, phosphorylation of Homer proteins at their hinge regions, in addition to Homer1a, results in decreased binding of these proteins to mGluRs [23, 24]. In fact, Preso reportedly enhances the phosphorylation of GluRs, such as NMDARs and group I mGluRs, and thereby regulates the interaction between GluRs and scaffold proteins [9, 18, 19]. Expanding on these prior views, we confirmed that this bidirectional regulatory effect on the phosphorylation of interacting binding sites is implicated in the regulation of the mGluR1-Homer1 interaction after TBI. The findings further clarified that Preso can simultaneously promote mGluR1 phosphorylation and inhibit Homer1 phosphorylation and thereby promotes the formation of the mGluR1-Homer1 complex. Thus, it is apparent that the opposing effect of Preso on the phosphorylation of mGluR1 and Homer1 is a novel mechanism affecting the roles of the mGluR1-Homer1 complex in excitotoxicity and TBI.

Studies have shown that CDK5 can promote the phosphorylation of the Homer-binding sites (TPPSPFR) of group I mGluRs and thereby enhances the mGluR-Homer interaction [21, 43]. A similar effect was observed for the ERK-mediated phosphorylation of group I mGluRs in the same region [18, 19]. Through its predicted D-domain, Preso serves as an adaptor of proline-directed kinases, including CDK5 and ERK [16, 18]. The regulatory effect of Preso on mGluR1 signaling is more dependent on endogenous CDK5 than on ERK [19]. On this basis, the present study confirmed that CDK5 mediates the phosphorylation of the Homer-binding site of mGluR1 by Preso after TBI. Because the activation of CDK5 by Preso is dependent on its D-domain, inducing the dissociation of CDK5 from the D-domain of Preso through the use of a blocking peptide inhibits the Preso-induced phosphorylation of mGluR1. In addition, disrupting the interaction between Preso and mGluR1 prevents the CDK5-mediated phosphorylation of mGluR1. Preso causes CDK5 and mGluR1 to become spatially closer through its multiple domains and thus provides a platform for the CDK5-induced promotion of mGluR1 phosphorylation.

Homer1 is characterized by a conserved enabled/vasodilator-stimulated phosphoprotein (Ena/VASP) homology 1 (EVH1) domain at the N-terminus and a coiled-coil (CC) domain at the C-terminus [37]. Interestingly, Preso affects the mGluR1-Homer1 interaction by dephosphorylating Homer1 in the hinge region between these two classical domains rather than in these domains themselves [24, 44]. This hinge region contains a proline-rich motif that stabilizes the interaction of mGluR with Homer [44]. In response to stimulation, the CaMKIIα-mediated phosphorylation of sites in the hinge region disrupts the mGluR-Homer interaction by affecting the P-motif [45]. After traumatic injury, Preso can inhibit the phosphorylation of the hinge region by CaMKIIα through its interaction with Homer1 and thus maintains the formation of the mGluR1-Homer1 complex. Preso likely interacts with the EVH1 domain of Homer1 through its Homer-binding domain, preventing CaMKIIα from occupying a suitable position to phosphorylate the hinge region [46]. This allows Preso to specifically regulate Homer1 phosphorylation and explains how Preso affects the mGluR1-Homer1 interaction via CaMKIIα-mediated Homer1 phosphorylation after TBI.

ER stress is an important downstream process that causes excitotoxicity after activation of mGluR1 signaling following TBI [26]. Homer1 has likewise been shown to promote excitotoxic neuronal injury through activation of ER stress [30]. These effects are associated with the formation of the mGluR1-Homer1 complex and related intracellular Ca^2+^ overload [38, 47]. In previous studies, we proved that Preso facilitated GluR-dependent neuronal Ca^2+^ overload, which in turn caused excitotoxicity [9, 17]. Moreover, the mGluR1-Homer1 complex is an important modulator of intracellular Ca^2+^ signaling by Preso [18, 19]. These results strongly suggest that the Preso-mediated modulation of the mGluR1-Homer1 interaction after TBI is closely related to the induction of ER stress. To confirm this hypothesis, we interfered with the interaction between Preso and the mGluR1-Homer1 complex and examined the activation of PERK/eIF2α signaling in response to ER stress. Our findings, together with previous results, establish a pathological mechanism by which Preso promotes the formation of the mGluR1-Homer1 complex and consequently activates intracellular Ca^2+^ overload-related ER stress, which ultimately causes apoptosis of neurons after TBI.

The therapeutic effects of different TAT-fused peptides that modulate glutamatergic neurotransmission have been shown in many animal models of neurological diseases, such as Alzheimer’s disease, stroke, and depression [48–50]. Due to their ability to permeate the blood‒brain barrier and cell membranes, TAT-fused peptides can be administered through the peripheral vein at low doses, which reduces the possibility of side effects. Notably, the therapeutic potential of NA-1 (nerinetide), a peptide fused with TAT that obstructs the interaction of NMDAR with PSD-95, has been satisfactorily tested in patients suffering from ischemic stroke [51, 52]. Accordingly, we assessed the neuroprotective effects of TAT-fused peptides targeting the interactions between Preso and the mGluR1-Homer1 complex in TBI. Among the four TAT-fused proteins with different targets, TAT-Preso-FM and TAT-mGluR1-H1 significantly promoted motor function recovery after TBI, whereas TAT-Preso-H1 and TAT-Preso-D showed no significant therapeutic effects. This result suggested that directly intervening with the interaction between mGluR1 and scaffold proteins in a complex in vivo environment had more obvious effects after TBI. Currently, most preclinical studies on TBI, similar to the present study, primarily utilize young male animals [53]. This is due to young males being the predominant population in TBI epidemiology and the avoidance of the influence of female sex hormones on neuroprotection-related animal experiments [53, 54]. However, this leads to a selection bias in the results of the study, which may not be applicable to all TBI populations. Consequently, preclinical studies investigating TBI should include males of all ages and females of various life stages to explore differences in pathogenic processes of TBI across age and gender [54, 55]. Therefore, while these two potent TAT-fused peptides appear to be potential candidates for TBI treatment, further studies are needed to evaluate their side effects, pharmacokinetics, and possible applications for clinical therapy.

## Materials and methods

### Animals

Mice of the C57BL/6JSmoc strain (wild-type, #SM-001) and C57BL/6JSmoc-*Frmpd4*^*em1Smoc*^ strain (Preso knockout, *Preso*^-/-^, #NM-KO-2100884) were obtained from Shanghai Model Organisms (Shanghai, China). Male mice, aged 10-12 weeks (initially weighing approximately 25 g) were utilized in this study. Prior to the commencement of the study, they were housed in a temperature-controlled environment at a steady temperature of around 27 °C for a minimum of 7 d and mantained on a 12-hour light/dark cycle. A total of 48 *Preso*^*-/-*^ mice and 48 wild-type mice were randomly assigned to animal experiments. All animal studies were conducted following the National Institutes of Health Guidelines for the Care and Use of Laboratory Animals and were approved by the Animal Care Committee of the Fourth Military Medical University.

### Primary culture of cortical neurons

A total of 20 mouse dams were used in various in vitro experiments, and cortical neuron cultures were prepared as previously described [9]. Briefly, we extracted cerebral cortices from 16–18-day-old embryos. The tissue dissociation process was carried out by gently triturating with trypsin (0.25%) at 37 °C for 15 min. The neuronal cells were resuspended in a neuronal cell culture medium (Neurobasal Medium, #21103049, Thermo Fisher Scientific, Waltham, MA, USA) supplemented with L-glutamine (0.5 mM) and B27 (2%) (#A3582801, Thermo Fisher Scientific) and then plated at a density of 3 × 10^5^ cells/cm^2^. Prior to seeding, the neuronal culture vessels, i.e., 6-cm dishes, 96-well plates, or 1.5-cm glass slides, were coated with poly-L-lysine (50 μg/mL) for the entire night at room temperature. The cultured neurons were kept in a 5% CO_2_ incubator with a humidified environment at a temperature of 37 °C, and the culture medium was replaced every second day. The neurons, with a viability of over 95%, were utilized for experiments on days in vitro (DIV)12-14.

### Antibodies and reagents

A Preso antibody was generated in rabbits using amino acids 1251-1265 in the N-terminus of Preso as the antigen. A phospho-mGluR1 (S1154) antibody and phospho-Homer1 (S117) antibody were generated in rabbits using a synthetic mGluR1 phosphopeptide (DSPALTPP(pS)PFRD) and a Homer1 phosphopeptide (ARLAKEK(pS)QEKMEL) as antigens. These antibodies were generated by Yurogen (Wuhan, Hubei, China). A primary antibody against Homer1 was obtained from Abcam (#ab184955, Cambridge, UK). Antibodies against mGluR1 (#12551), phospho-PERK (#3179), PERK (#3192), phospho-eIF2α (#3398), eIF2α (#5324), CDK5 (#2506), β-actin (#3700), and NeuN (#94403) were obtained from Cell Signaling Technology (Danvers, MA, USA). For immunoblotting, we used HRP-conjugated goat anti-mouse (#sc-2005) and goat anti-rabbit (#sc-2004) secondary antibodies from Santa Cruz Biotechnology (Dallas, TX, USA). The immunostaining utilized the Alexa Fluor 488 mouse IgG secondary antibody (#A-11029, Thermo Fisher Scientific). As antagonists of mGluR1, LY367385 was obtained from MedChemExpress (Shanghai, China), and Bay 36-7620 was obtained from Tocris Bioscience (Bristol, UK). Ro 67-7476 (#HY-100403) and Ro 0711401 (#HY-124419), two positive allosteric modulators of mGluR1, were obtained from MedChemExpress. KN93 (#HY-15465), an antagonist of CaMKIIα, was obtained from MedChemExpress. Purvalanol B (#1581/10), an antagonist of CDK5, was obtained from Tocris Bioscience (Bristol, UK). GSK 2606414 (#5107/10, antagonist of PERK) and Sal 003 (#3657/10, antagonist of eIF2α) were obtained from Tocris Bioscience.

### Blocking peptides

All peptides were synthesized by Genescripts (Nanjing, Jiangsu, China) and dissolved in saline. The dosage and method of administering TAT-fused peptides were chosen based on prior research [48, 50]. The TAT peptides used were as follows: TAT-mGluR1-H1, grkkrrqrrrpq-DSPALTPPSPFRDSVA, which consisted of amino acids 1146-1161 of mGluR1 and an N-terminal TAT tag (lower case) to allow cell permeability; TAT-mGluR1-H1mu (grkkrrqrrrpq-DSPALTPLSPRRDSVA), which was designed as a nonbinding control for TAT-mGluR1-H1; TAT-mGluR1-FM (grkkrrqrrrpq-KTLYNVEEE), which consisted of amino acids 952-960 of mGluR1 and an N-terminal TAT tag; TAT-mGluR1-FMmu (grkkrrqrrrpq-KTLANAEEE), which was designed as a nonbinding control for TAT-mGluR1-FM; TAT-Preso-H1 (grkkrrqrrrpq-AAPPPGFRDS), which consisted of amino acids 800-809 of Preso and an N-terminal TAT tag; TAT-Preso-H1mu (grkkrrqrrrpq-AAPLPGRRDS), which was designed as a nonbinding control for TAT-Preso-H1; TAT-Preso-D (grkkrrqrrrpq-EKRSEVTLLVGPRYG), which consisted of amino acids 641-655 of Preso and an N-terminal TAT tag; and TAT-Preso-Dscr (grkkrrqrrrpq-PGYLRRGVETKLESV), which was designed as a scramble control for TAT-Preso-D.

### TBI models

A TNI model was used to simulate TBI in vitro. The injury was performed as described in our previous research, with some modifications as described by Mukhin et al. [9, 14, 56]. For TNI modeling, adherent cells were scraped from a culture plate with a plastic probe, disrupting some processes and somata but leaving a substantial proportion of the cells intact. Briefly, a sterile plastic pipette tip was used to manually scratch each confluent cell culture following a square grid with 3 mm spacing between the lines. All TNI models were performed by the same investigator using a standard square grid module to minimize damage inconsistency between experiments. The cultured neurons were utilized for in vitro experiments on DIV 12-14 in culture. The cultured cells were incubated at 37 °C until a specified time post-injury, with regular replacement of the medium. Uninjured cell cultures served as control samples. As scratch injuries initially activate neurons located at the wound edge and subsequently activate the entire neuronal monolayer, all analyses were performed using the entire dish.

In addition, the present study used an in vivo CCI model to simulate TBI. Under 4% isoflurane anaesthesia, the mice were positioned on a stereotaxic frame platform. A longitudinal scalp incision was made along the midline, exposing the skull. A hole with a 5 mm diameter was drilled, while keeping the dura intact. Injury was produced by striking the right cortex with a 3 mm diameter actuator at a velocity of 3 m/s and a depth of 1.5 mm. The wound on the scalp was closed using conventional suture material. Lidocaine cream was applied in the area of the wound. The mice were reintroduced to their cages following the surgical procedure and were granted unrestricted access to food and water.

### Lentivirus preparation for RNAi delivery and overexpression

All lentiviruses were generated by and obtained from GeneChem Company (Shanghai, China). To generate shRNA-expressing lentiviruses, we subcloned an siRNA oligo (CCTTGTGTCCCAAAGAGCA) into the GV248 lentiviral vector (hU6-MCS-Ubiquitin-EGFP-IRES-puromycin). To create lentiviruses that overexpress certain proteins, we subcloned the cDNAs of Preso, Preso with a HBM point mutation (F806R), mGluR1, mGluR1 with a dephosphomimetic Homer-binding site mutation (S1154A), mGluR1 with a phosphomimetic Homer-binding site mutation (S1154D), Homer1, Homer1 with a dephosphomimetic hinge region mutation (S117A), and Homer1 with a phosphomimetic hinge region mutation (S117D) into the G492 lentiviral vector (Ubi-MCS-3FLAG-CBh-gcGFP-IRES-puromycin). Cultured neurons were transfected on DIV12-14. Subsequently, the neurons underwent a 72 hour transfection with various lentiviruses before being prepared for further experiments.

### Western blot analysis

After different treatments, cells or brain samples from the injured area were lysed with buffer supplemented with phosphatase inhibitor (#5892970001) and PhosSTOP protease inhibitor (#4906845001) tablets (Roche Applied Bioscience, Indianapolis, IN, USA). The concentration of protein in the supernatant was quantified by means of a BCA protein assay kit. The protein samples were separated using SDS-PAGE gels with 10-15% concentration and subsequently transferred onto nitrocellulose membranes from Thermo Fisher Scientific. The membranes were sliced at designated molecular weights in line with prestained protein ladders (#26617, Thermo Fisher Scientific). The membranes were immersed in a solution of Tris-buffered saline and 0.05% Tween 20 (TBST) mixed with 5% nonfat milk for one hour at room temperature. Thereafter, they were incubated at 4 °C overnight with specific primary antibodies (Preso, 1:800 dilution; Homer1, 1:1000 dilution; mGluR1, 1:800 dilution; PERK, 1:1000 dilution; eIF2α, 1:1000 dilution; β-actin, 1:1500 dilution; phospho-Homer1 (S117), 1:1000 dilution; phospho-mGluR1 (S1154), 1:800 dilution; phospho-PERK, 1:800 dilution; phospho-eIF2α, 1:800 dilution). The membranes were rinsed with TBST and then exposed to secondary antibodies diluted in the blocking buffer for one hour at room temperature. The Preso, Homer1, phospho-Homer1 (S117), phospho-mGluR1 (S1154), PERK, phospho-PERK, eIF2α, and phospho-eIF2α antibodies were specifically identified using a goat anti-rabbit antibody. The mGluR1 and β-actin antibodies were specifically identified using a goat anti-mouse antibody. Thermo Fisher Scientific SuperSignal West Pico Chemiluminescent Substrate (Thermo Fisher Scientific) was used to detect immunoreactivity. The membranes were stripped with Reblot Plus Strong Solution (#2504, Millipore, Burlington, MA, USA) for 15 min at room temperature and reprobed with antibodies of mGluR1, Homer1, PERK, and eIF2α to standardize phosphorylated protein levels. Image analysis system (ImageJ, National Institutes of Health, MA, USA) was used to quantify the optical densities of the bands.

### Histological staining

#### Mouse perfusion and brain tissue processing

The animals underwent transcardial perfusion and were subsequently postfixed through paraformaldehyde (4%) in phosphate buffer (0.1 M). The brain specimens were embedded in Tissue-Tek (SAKURA 4583, Torrance, CA, USA), an optimal cutting temperature compound, and sliced into 16-μm coronal sections using a freezing microtome (Leica CM 1950, Wetzlar, Germany). The resulting sections were then mounted on slides for analysis.

#### Immunofluorescence staining

Sections underwent three 10-min washes using 0.01 M PBS, then were blocked for 1 h using 5% bovine serum albumin in PBS with 0.3% Triton-X100. The tissue sections underwent overnight incubation at 4 °C with the primary antibody (NeuN, 1:1000). Subsequently, they underwent incubation at room temperature for 3 h with the secondary antibody (Alexa Fluor 488, 1:500). These antibodies were diluted in a solution consisting of 5% bovine serum albumin in PBS with 1% Triton-X100. The cellular nuclei were stained by incubating the slides in PBS diluted 4′,6-diamidino-2-phenylindole (DAPI) (1:1000, Sigma, St. Louis, MO, USA, D9564) for 10 min. The slides were then coverslipped with a mounting medium containing 50% glycerol.

#### TUNEL staining

Sections were treated with 4% paraformaldehyde for 30 min, followed by two 10-minute washes with 0.01 M PBS. Subsequently, the sections were incubated with 0.5% Triton-X100/PBS for 5 minutes at room temperature. Next, a One Step TUNEL Apoptosis Assay Kit (Beyotime Biotechnology, C1086, Shanghai, China) was used. The TUNEL assay solution was applied to the slides. The slides were then covered with an anti-evaporation film and incubated at 37 °C for 60 min in the dark. The cellular nuclei were labeled by incubating the slides with DAPI for 10 minutes, followed by three 5-minute washes. The slides were then coverslipped with a mounting medium containing 50% glycerol.

#### Nissl staining

Coronal sections were dyed using Nissl staining solution (#C0117, Beyotime) for 30 min at 37 °C. The sections were washed with 95% ethanol and examined under an optical microscope. A normal neuron is characterized by a large cell body with plentiful cytoplasm and high levels of Nissl bodies.

#### Staining analysis

The stained sections were imaged using an BX51 microscope and an FV3000 laser confocal microscope (Olympus, Tokyo, Japan). For each animal, we analyzed five randomly selected fields in the cortex, ipsilateral to the lesion. Each field measured 500 × 300 μm^2^ and was analyzed in five non-adjacent sections that were 100 μm apart. The average was calculated from the five sections. The quantification of cells was conducted using Image-Pro Plus 6.0 software (Media Cybernetics, Rockville, MD, USA). Six mice were included in each group for the analysis.

### Real-time reverse transcription-polymerase chain reaction (real-time PCR)

TRIzol (Invitrogen) was utilized to isolate RNA from primary cortical neurons. After diluting the RNA samples to ensure equal concentrations, we used a commercially available kit (TaKaRa, Dalian, China) to perform reverse transcription and real-time PCR. Following the generation of cDNA, we conducted quantitative PCR using the Bio-Rad iQ5 Gradient Real-Time PCR system (Bio-Rad Laboratories). The following primers were used: ATF4, 5’-CCT GAA CAG CGA AGT GTT GG-3’ (forward) and 5’-TGG AGA ACC CAT GAG GTT TCA A-3’ (reverse); NRF2, 5’-CTT TAG TCA GCG ACA GAA GGA C-3’ (forward) and 5’-AGG CAT CTT GTT TGG GAA TGT G-3’ (reverse); CHOP, 5’-CAC ATC CCA AAG CCC TCG CTC TC-3’ (forward) and 5’-TCA TGC TTG GTG CAG GCT GAC CAT-3’ (reverse); GADD34, 5’-CTT TTG GCA ACC AGA ACC G-3’ (forward) and 5’-CAG AGC CGC AGC TTC TAT CT-3’ (reverse); NOXA, 5’-GAC AAA GTG AAT TTA CGG CAG A-3’ (forward) and 5’-GGT TTC ACG TTA TCA CAG CTC A-3’ (reverse); HMOX1, 5’-GCC GAG AAT GCT GAG TTC ATG-3’ (forward) and 5’-TGG TAC AAG GAA GCC ATC ACC-3’ (reverse); NQO1 primers, 5’-CGC CTG AGC CCA GAT ATT GT-3’ (forward) and 5’-GCA CTC TCT CAA ACC AGC CT-3’ (reverse); and β-actin, 5’-CTA AGG CCA ACC GTG AAA AGA TG-3’ (forward) and 5’-ACC GCT CGT TGC CAA TAG TGA TG-3’ (reverse). Fifty cycles of quantitative PCR were executed with the following parameters: 94 °C for 30 seconds, 58 °C for 30 seconds, and 72 °C for 30 seconds. The mRNA levels were normalized using β-actin, and the resulting fold change in relation to the control group was calculated.

### LDH assay

Cytotoxicity was assessed by quantifying the release of LDH, a cytoplasmic enzyme that is discharged from cells into the culture medium when the plasma membrane is damaged, indicating a loss of membrane integrity. LDH release into the culture medium was quantified by utilizing a LDH cytotoxicity assay kit procured from Cayman Chemical (Ann Arbor, MI, USA) as per the provided instructions. Briefly, a fresh 96-well plate was filled with 100 μL of supernatant from each well. Reaction solution of LDH was added to each well. Mixtures were shaken gently and incubated at 37 °C for 30 minutes. LDH activity was determined by measuring the absorbance at 490 nm using a plate reader.

### Measurement of caspase-3 activity

Caspase-3 activity was assessed using the Caspase-3 Colorimetric Assay Kit obtained from Abcam (#ab39401) in strict accordance with the manufacturer’s instructions. Neuronal lysates were incubated with 200 μM DEVD-ρNA substrate at 37 °C for 2 hours. The absorbance of every sample was measured through an ELISA microplate reader.

### Coimmunoprecipitation (Co-IP)

Co-IP experiments were performed to assess protein interactions. The Pierce™ Crosslink Magnetic IP/Co-IP Kit (Thermo Fisher Scientific) was used according to the manufacturer’s instructions. Cortical neurons were cultured in 100-mm dishes, followed by cell harvesting using an ice-cold lysis/wash buffer containing a proteinase inhibitor. The protein extracts were subjected to centrifugation at 13,000 ×g for 15 min at 4 °C. Protein concentrations were analyzed using the BCA protein assay kit (Thermo Fisher Scientific). Magnetic beads were incubated with 2 mg non-specific mouse IgG, anti-Homer1 (Abcam), anti-mGluR1 (NeuroMab) and anti-Preso. After incubation, the beads were washed twice with coupling buffer. The protein extracts were mixed with the beads for overnight incubation at 4 °C. After magnetic isolation, the precipitates underwent three washes with wash buffer, were eluted with elution buffer, neutralized with neutralization buffer, and then made ready for western blotting.

### Motor function tests

#### Rotarod test

The test utilized a rotarod device (Shanghai XinRuan Information Technology, Shanghai, China) in the accelerating version to measure the subjects’ performance. The rod was 3 cm in diameter. The initial rotation speed of the rotarod was set at 5 rpm, with a speed acceleration of 20 rpm/min, reaching a maximum velocity of 40 rpm. The testing period was limited to a maximum of 5 min. After each mouse was positioned on the rod, acceleration was started ten seconds later and the velocity at which the mouse fell off the rod was recorded. Before each experiment and between experiments, the instrument was cleaned with 75% alcohol to ensure that the conditions were the same for each test. Each experiment was repeated 3 times per mouse, with an interval of no less than 45 min to allow the mice to recover. The average velocity of the mouse falling from the rod during three trials was measured.

#### FUA test

A mouse was placed in an enclosed cylinder (15 cm × 9 cm) and forelimb use was recorded during at least 15 exploratory movements (fewer than 20) for up to 10 minutes. The number of forelimb usage on the side of the brain injury was measured and recorded as the ipsilateral (I) value. The number of forelimb usage on the side opposite the brain injury was measured and recorded as the contralateral (C) value. The bilateral (B) value was calculated by counting the number of times both forelimbs were used. The FUA score was calculated as [I-C]/[I + C + B] × 100.

### Water content measurement

The evaluation of the extent of cerebral edema was conducted through the determination of tissue water content in the affected hemisphere, as reported previously [57]. The mice were euthanized under deep anaesthesia using 100 mg/kg pentobarbital. The brains were quickly extracted and the hemispheres were divided along the sagittal plane. Three-millimeter coronal sections of the region adjacent to the lesion were carefully prepared. The sections were weighed based on their wet weight and then subjected to a desiccator oven for 24 hours at a temperature of 95 °C to dry. The water content of the cortex on the same side of the injury (Wi) and on the opposite side of the injury (Wc) was calculated after weighing the dried sections (based on dry weight) using the provided equation: water content (g/g dry weight) = (wet weight – dry weight)/dry weight. The following equation was then used to calculate the difference between the Wi and Wc: % change in water content = [(Wi - Wc) × 100] / Wc.

### Statistical analysis

Statistical analysis was carried out using GraphPad Prism software, version 9.0 (GraphPad, San Diego, CA, USA). The Kolmogorov-Smirnov test was employed to assess whether the data within the sample groups showed normal distribution. For the statistical analysis of two groups, we determined variance homogeneity using the F test and analyzed the significance of the difference between the two groups using an unpaired two-tailed Student’s t-test. For the statistical analysis of more than two groups, we utilized the Brown-Forsythe test to determine variance homogeneity. We then applied one-way ANOVA followed by Bonferroni’s multiple comparisons test to determine the significance of differences among all groups. For samples that underwent multiple measurements at different time points, we utilized repeated measures ANOVA with the Geisser-Greenhouse correction, followed by Bonferroni’s multiple comparisons test, to ascertain the statistical significance of the group differences at specific time points. The data are expressed as the means ± SDs. A *P* value less than 0.05 was considered to indicate statistical significance.

### Supplementary information

Supplementary Materials

Original Data File

Reproducibility Checklist

## Data Availability

The experimental datasets generated and/or analyzed during the current study are available from the corresponding author upon reasonable request. No applicable resources were generated during the current study.
