# Supplementary material for: Preso enhances mGluR1-mediated excitotoxicity by modulating the phosphorylation of mGluR1-Homer1 complex and facilitating an ER stress after traumatic brain injury
Source: Cell Death Discov. 2024 Mar 26;10:153. doi: 10.1038/s41420-024-01916-5 (PMC10965980; doi:10.1038/s41420-024-01916-5)
Supplement: Supplementary file 1 — Supplementary Materials [file 41420_2024_1916_MOESM1_ESM.pdf]

Table S1. Summary of the *in vitro* experimental procedures concerning neuronal cultures

Figure Number	Lentiviral transfection (transfection for 72 h)	Drug pretreatment (after transfection)	*In vitro* model (1 h after drug treatment)	Experiments
Fig. 1A<br>Fig. S1A-C	LV-Con or LV-Preso	DMSO, Bay 36-7620, or CPCCOEt	TNI for 24 h	Caspase-3 activity assay or LDH assay
Fig. 1B<br>Fig. S1D-F	LV-shCon or LV-shPreso	DMSO, Ro-67-7476, or Ro 0711401	TNI for 24 h	Caspase-3 activity assay or LDH assay
Fig. 1C	LV-shCon or LV-shPreso	N/A	TNI for 24 h	Western blot
Fig. 1E-F<br>Fig. S1G	N/A	TAT-mGluR1-FMmu or TAT-mGluR1-FM	TNI for 24 h	Western blot, Co-IP, caspase-3 activity assay, or LDH assay
Fig. 2B-C<br>Fig. S3A	N/A	TAT-mGluR1-H1mu or TAT-mGluR1-H1	TNI for 24 h	Western blot, Co-IP, caspase-3 activity assay, or LDH assay
Fig. 2D	LV-shCon, LV-shPreso, LV-Con, or LV-Preso	N/A	TNI for 24 h	Western blot and Co-IP
Fig. 2E	N/A	TAT-mGluR1-FMmu or TAT-mGluR1-FM	TNI for 24 h	Western blot and Co-IP
	LV-Preso or LV-Preso	N/A		

	F806R			
Fig. 2F<br>Fig. S3B	LV-Con or LV-Preso	TAT-mGluR1-H1mu or TAT-mGluR1-H1	TNI for 24 h	Caspase-3 activity assay or LDH assay
Fig. 3A-B	LV-mGluR1, LV-mGluR1 S1154A, LV-mGluR1, S1154D, LV-Homer1, LV-Homer1 S117A, or LV-Homer1 S117D	N/A	TNI for 24 h	Western blot and Co-IP
Fig. 3C-D<br>Fig. S6A-B	LV-mGluR1, LV-mGluR1 S1154A, LV-mGluR1, S1154D, LV-Homer1, LV-Homer1 S117A, or LV-Homer1 S117D	N/A	TNI for 24 h	Caspase-3 activity assay or LDH assay
Fig. 3E	LV-shCon or LV-shPreso	N/A	TNI for 24 h	Western blot
Fig. 3F	N/A	TAT-mGluR1-FMmu or TAT-mGluR1-FM	TNI for 24 h	Western blot
Fig. 3H	N/A	TAT-mGluR1-H1mu or TAT-mGluR1-H1	TNI for 24 h	Western blot and Co-IP

Fig. 4A	N/A	DMSO or purvalanol B	TNI for 24 h	Western blot and Co-IP
Fig. 4B	LV-Con or LV-Preso	DMSO or purvalanol B	TNI for 24 h	Western blot
Fig. 4D-E<br>Fig. S8A	N/A	TAT-Preso-Dsc or TAT-Preso-D	TNI for 24 h	Western blot, Co-IP, caspase-3 activity assay, or LDH assay
Fig. 5A	N/A	DMSO or KN93	TNI for 24 h	Western blot and Co-IP
Fig. 5B	LV-shCon or LV-shPreso	DMSO or KN93	TNI for 24 h	Western blot
Fig. 5C-D<br>Fig. S8B	N/A	TAT-mGluR1-H1mu, DMSO, or KN93<br>TAT-mGluR1-H1, DMSO, or KN93	TNI for 24 h	Western blot, Co-IP, caspase-3 activity assay, or LDH assay
Fig. 6A-B	LV-shCon or LV-shPreso	N/A	TNI for 24 h	Western blot or PCR
Fig. 6C<br>Fig. S10A-C	LV-Con or LV-Preso	DMSO, GSK 2606414, or Sal 003	TNI for 24 h	Caspase-3 activity assay or LDH assay
Fig. 6D	LV-Con or LV-Preso	TAT-mGluR1-H1mu or TAT-mGluR1-H1	TNI for 24 h	Western blot
Fig. 6E	LV-Preso	TAT-mGluR1-H1mu or TAT-mGluR1-H1	TNI for 24 h	PCR
Fig. S2	N/A	N/A	TNI for 24 h	Western blot and Co-IP
Fig. S4A	LV-shCon, LV-shPreso, LV-Con, or LV-Preso	N/A	N/A	Western blot and Co-IP
Fig. S4B	LV-Preso or LV-Preso	N/A	N/A	Western blot and Co-IP

	F806R			
Fig. S5	N/A	N/A	TNI for 24 h	Western blot
Fig. S7	LV-shCon or LV-shPreso	N/A	N/A	Western blot
Fig. S9	LV-Con or LV-Preso	N/A	N/A	Western blot
Fig. S11	N/A	TAT-mGluR1-H1mu or TAT-mGluR1-H1	TNI for 24 h	Western blot

Table S2. Summary of the statistical analysis of the data from each experiment

Figure Number	Biological repeats	Statistical test	Post hoc test
Fig. 1A-B, Fig. 1F<br>Fig. 2C, Fig. 2F<br>Fig. 4B<br>Fig. 5B-D<br>Fig. 6B-E<br>Fig. 7B<br>Fig. S1<br>Fig. S3<br>Fig. S8B<br>Fig. S10<br>Fig. S12A	6	Two-way ANOVA	Bonferroni's multiple comparisons test
Fig. 1C, Fig 1E<br>Fig. 2B, Fig. 2D-E<br>Fig. 3A-F, Fig. 3H<br>Fig. 4A, Fig. 4D-E<br>Fig. 5A<br>Fig. 6A<br>Fig. 7E-G<br>Fig. S4<br>Fig. S6<br>Fig. S7<br>Fig. S8A<br>Fig. S9	6	One-way ANOVA	Bonferroni's multiple comparisons test
Fig. 8C-D<br>Fig. 9B-G<br>Fig. S12B-C<br>Fig. S14<br>Fig. S15<br>Fig. S16	8	Two-way ANOVA	Bonferroni's multiple comparisons test
Fig. S2<br>Fig. S5<br>Fig. S11<br>Fig. S13	6	T test	N/A

Supplementary figure legends

Figure S1. Preso regulates mGluR1-mediated excitotoxicity after TNI. (A-C) Inhibition of mGluR1 reduced neuronal injury induced by upregulation of Preso. After transfection with LV-Con or LV-Preso and pretreatment with Bay 36-7620 (10 μM), CPCCOEt (10 μM) or vehicle (DMSO), cytotoxicity (A, C) and neuronal apoptosis (B) were measured by LDH assay and caspase-3 activity assay at 24 h after TNI, respectively. The data are presented as the means ± SDs of six biological repeats. *$p < 0.05$ *vs*. the LV-Con group; $^{\#}p < 0.05$ *vs*. the vehicle group. **(D-F)** Downregulation of Preso attenuated the overactivation of mGluR1-associated excitotoxicity. After transfection with LV-shCon or LV-shPreso and pretreatment with Ro 67-7476 (1 μM), Ro 0711401 (1 μM) or vehicle, cytotoxicity (D, F) and neuronal apoptosis (E) were measured by LDH assay and the caspase-3 activity assay at 24 h after TNI. The data are presented as the means ± SDs of six biological repeats. *$p < 0.05$ *vs*. the LV-Con group; $^{\#}p < 0.05$ *vs*. the vehicle group. **(G)** Disruption of the Preso-mGluR1 interaction by TAT-mGluR-FM reduced excitotoxicity. After pretreatment with TAT-mGluR-FM or TAT-mGluR1-FMmu, cytotoxicity following TNI was determined by LDH assay. The data are presented as the means ± SDs of six biological repeats. *$p < 0.05$ *vs*. the TAT-mGluR1-FMmu control group.

Figure S2. Enhancement of the mGluR1-Homer1 interaction after TNI. Immunoprecipitation with an anti-Homer1 antibody was performed at 24 h after TNI, and the expression levels of mGluR1 and Homer1 were analyzed by western blotting. The data are presented as the means ± SDs of six biological repeats. *$p < 0.05$ *vs*. the control group.

Figure S3. Preso regulates excitotoxicity related to the mGluR1-Homer1 interaction after TNI. (A) Disruption of the mGluR1-Homer1 interaction by TAT-mGluR-H1 reduced excitotoxicity. After pretreatment with TAT-mGluR-H1 or TAT-mGluR1-H1mu, cytotoxicity following TNI was determined by LDH assay. The data are presented as the means ± SDs of six biological repeats. *$p < 0.05$ *vs*. the TAT-mGluR1-H1mu control group. (B) Disruption of the

mGluR1-Homer1 complex suppressed the excitotoxicity induced by Preso upregulation. After transfection with LV-Con or LV-Preso and pretreatment with TAT-mGluR-H1 or TAT-mGluR1-H1mu, cytotoxicity following TNI was determined by LDH assay. The data are presented as the means ± SDs of six biological repeats. *$p < 0.05$ *vs*. the LV-Con group; #$p < 0.05$ *vs.* the TAT-mGluR1-H1mu control group.

Figure S4. Preso regulates the interaction between mGluR1 and Homer1 under normal conditions. (A) Preso positively regulated mGluR1-Homer1 complex formation. After transfection with LV-shCon, LV-shPreso, LV-Con, or LV-Preso, immunoprecipitation with an anti-Homer1 antibody was performed, and the expression of mGluR1 and Preso was analyzed by western blotting. The data are presented as the means ± SDs of six biological repeats. *$p < 0.05$ *vs*. the LV-shCon group; $^{\#}p < 0.05$ *vs*. the LV-Con group. **(B)** Blocking the association between Preso and Homer1 inhibited the effect of Preso on the mGluR1-Homer1 interaction. After transfection with LV-Preso or LV-Preso F806R, immunoprecipitation with an anti-Homer1 antibody was performed, and the expression of mGluR1 and Homer1 was analyzed by western blotting. The data are presented as the means ± SDs of six biological repeats. *$p < 0.05$ *vs.* the LV-Preso group.

Figure S5. Traumatic injury differentially regulates the phosphorylation of mGluR1 and Homer1. The phosphorylation of mGluR1 at S1154 and Homer1 at S117 at 24 h after TNI was analyzed by western blotting. The data are presented as the means ± SDs of six biological repeats. *$p < 0.05$ *vs*. the control group.

Figure S6. The phosphorylation of mGluR1 and Homer1 bidirectionally regulates excitotoxicity after TNI. (A) The phosphorylation of mGluR1 increased excitotoxicity. After transfection with LV-mGluR1, LV-mGluR1 S1154A, or LV-mGluR1 S1154D, cytotoxicity following TNI was determined by LDH assay. The data are presented as the means ± SDs of six biological repeats. *$p < 0.05$ *vs*. the LV-mGluR1 group; $^{\#}p < 0.05$ *vs.* the LV-Homer1 group. **(B)** The phosphorylation of Homer1 decreased excitotoxicity. After transfection with LV-Homer1,

LV-Homer1 S117A, or LV-Homer1 S117D, cytotoxicity following TNI was determined by LDH assay. The data are presented as the means ± SDs of six biological repeats. *p < 0.05 *vs.* the LV-mGluR1 group; $^{\#}$p < 0.05 *vs.* the LV-Homer1 group.

Figure S7. The downregulation of Preso expression induces hypophosphorylation of mGluR1 and hyperphosphorylation of Homer1 under normal conditions. After transfection with LV-shCon or LV-shPreso, the phosphorylation of mGluR1 at S1154 and the phosphorylation of Homer1 at S117 were analyzed by western blotting. The data are presented as the means ± SDs of six biological repeats. *p < 0.05 *vs*. the LV-shCon group.

Figure S8. Preso regulates CDK5- and CaMKIIα-related excitotoxicity. (A) Disruption of the interaction between Preso and CDK5 reduced excitotoxicity. After pretreatment with TAT-Preso-Dscr or TAT-Preso-D, cytotoxicity following TNI was determined by LDH assay. The data are presented as the means ± SDs of six biological repeats. *p < 0.05 *vs*. the TAT-Preso-Dscr control group. (B) After disruption of the interaction between Preso and Homer1, inhibition of CaMKIIα increased excitotoxicity. After pretreatment with TAT-Preso-H1, TAT-Preso-H1mu, KN93, or vehicle, cytotoxicity following TNI was determined by LDH assay. The data are presented as the means ± SDs of six biological repeats. *p < 0.05 *vs*. the TAT-Preso-H1mu control group; $^{\#}$p < 0.05 *vs*. the Vehicle + TNI group.

Figure S9. Preso upregulation induces ER stress signaling. After transfection with LV-Con or LV-Preso, the phosphorylation of PERK and eIF2α was analyzed by western blotting. The data are presented as the means ± SDs of six biological repeats. *p < 0.05 *vs*. the LV-Con group.

Figure S10. Inhibition of ER stress attenuates neuronal injury induced by Preso upregulation after TNI. After transfection with LV-Con or LV-Preso and pretreatment with GSK 2606414 (10 μM), Sal 003 (10 μM), or vehicle (DMSO), cytotoxicity (A, C) and neuronal apoptosis (B) were measured by LDH assay and the caspase-3 activity assay at 24 h after TNI.

The data are presented as the means ± SDs of six biological repeats. *$p < 0.05$ *vs.* the vehicle group.

Figure S11. Disruption of the mGluR1-Homer1 interaction reduces ER stress after TNI. After pretreatment with TAT-mGluR1-H1 or TAT-mGluR1-H1mu, the phosphorylation of PERK and eIF2α was analyzed by western blotting (A) at 24 h after TNI, whereas the expression of ER stress target genes (*ATF4*, *NRF2*, *CHOP*, *GADD34*, *NOXA*, *HMOX1*, and *NQO1*) was examined by PCR (B). The data are presented as the means ± SDs of six biological repeats. *$p < 0.05$ *vs.* the TAT-mGluR1-H1mu control group.

Figure S12. *Preso*$^{-/-}$ mice exhibit no significant difference in motor function after sham treatment. Motor function did not change in *Preso*$^{-/-}$ mice. The rotarod test **(A)** and FUA test **(B)** were performed at designated time points following sham treatment. The data are presented as the means ± SDs of eight biological repeats. *$p < 0.05$ *vs.* the WT mice.

Figure S13. Disruption of the mGluR1-Homer1 interaction and bidirectional regulation of mGluR1 phosphorylation and Homer1 phosphorylation in *Preso*$^{-/-}$ mice. After sham treatment, immunoprecipitation with an anti-Homer1 antibody was performed following TBI, and the phosphorylation of mGluR1 at S1154 and Homer1 at S117 and the expression of mGluR1 and Homer1 were analyzed by western blotting. The data are presented as the means ± SDs of six biological repeats. *$p < 0.05$ *vs.* the WT mice.

Figure S14. TAT-Preso-H1 exerts no significant effect on motor function after TBI. After administration of TAT-Preso-H1 or TAT-Preso-H1mu, the rotarod test and FUA test were performed at 6 d, 8 d, 10 d, 12 d, and 14 d following TBI. The data are presented as the means ± SDs of eight biological repeats.

Figure S15. TAT-Preso-D exerts no significant effect on motor function after TBI. After administration of TAT-Preso-D or TAT-Preso-Dscr, the rotarod test and FUA test were performed at 6 d, 8 d, 10 d, 12 d, and 14 d following TBI. The data are presented as the means ± SDs of eight biological repeats.

Figure S16. TAT-mGluR1-H1 improves the recovery of motor function in *Preso*$^{-/-}$ mice. After *Preso*$^{-/-}$ mice were treated with TAT-mGluR1-H1 or TAT-mGluR1-H1mu, the rotarod test and FUA test were performed at 6 d, 8 d, 10 d, 12 d, and 14 d following TBI. The data are presented as the means ± SDs of eight biological repeats. *$p < 0.05$ *vs.* the TAT-mGluR1-FMmu+*Preso*$^{-/-}$ group.

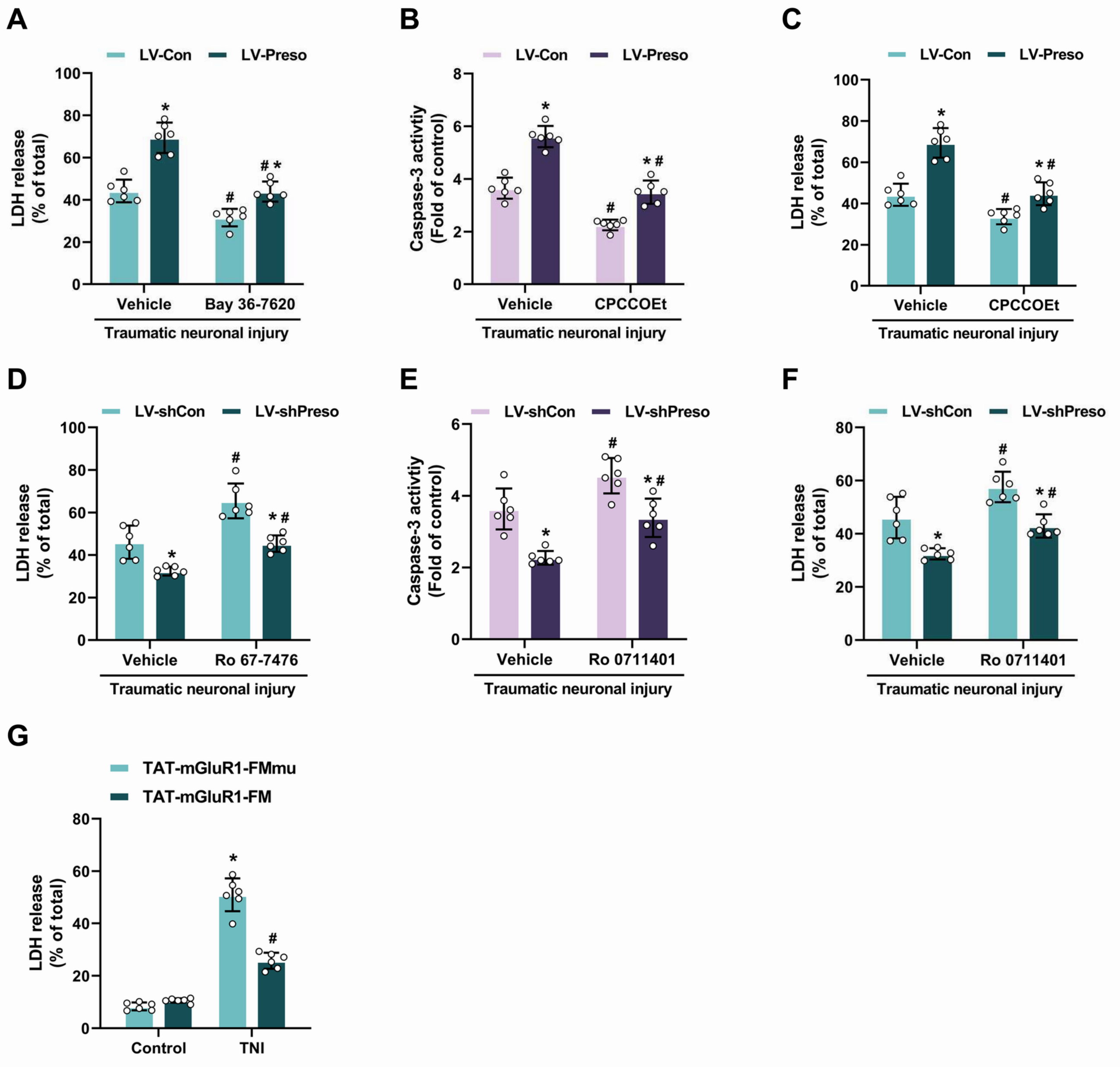
A
LV-Con
LV-Preso
LDH release (% of total)
100
80
60
40
20
0
Vehicle
Bay 36-7620
Traumatic neuronal injury
B
LV-Con
LV-Preso
Caspase-3 activtiy (Fold of control)
8
6
4
2
0
Vehicle
CPCCOEt
Traumatic neuronal injury
C
LV-Con
LV-Preso
LDH release (% of total)
Vehicle
CPCCOEt
Traumatic neuronal injury
D
LV-shCon
LV-shPreso
LDH release (% of total)
Vehicle
Ro 67-7476
Traumatic neuronal injury
E
LV-shCon
LV-shPreso
Caspase-3 activtiy (Fold of control)
Vehicle
Ro 0711401
Traumatic neuronal injury
F
LV-shCon
LV-shPreso
LDH release (% of total)
Vehicle
Ro 0711401
Traumatic neuronal injury
G
TAT-mGluR1-FMmu
TAT-mGluR1-FM
LDH release (% of total)
Control
TNI

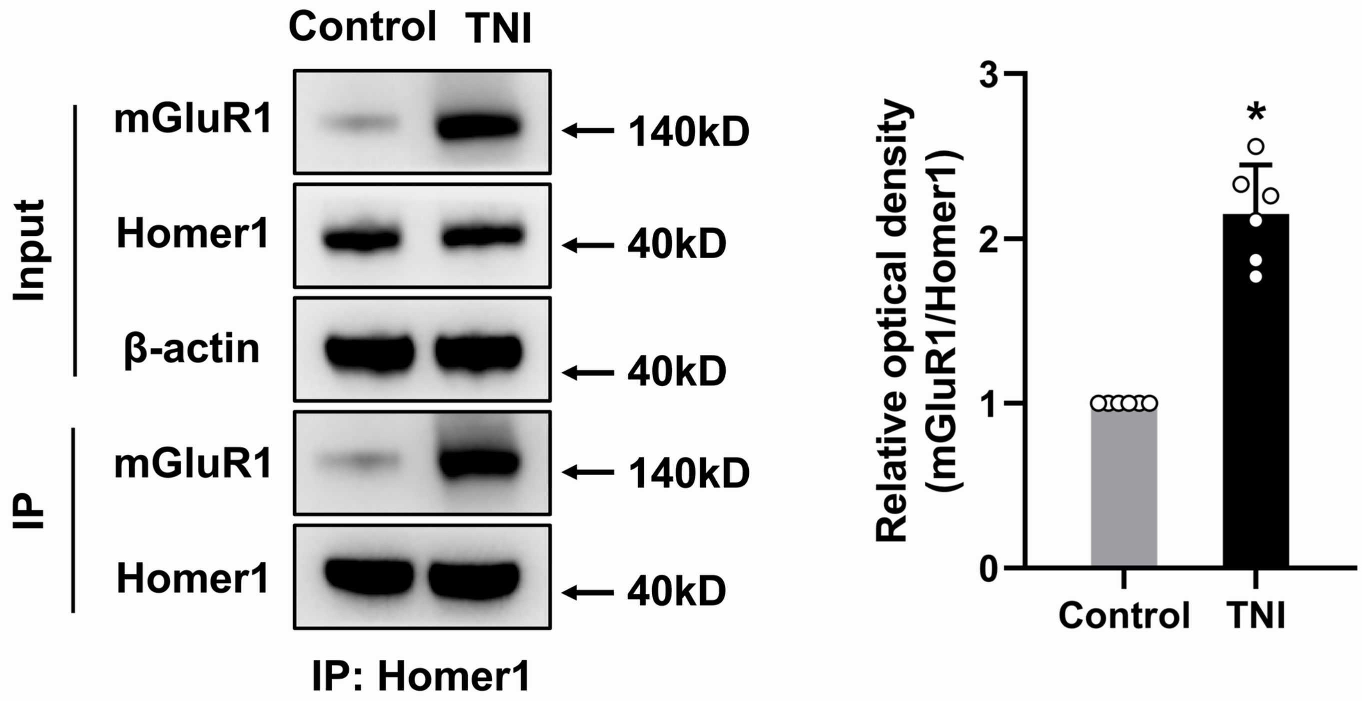

Control
TNI
Input
mGluR1
140kD
Homer1
40kD
β-actin
40kD
IP
mGluR1
140kD
Homer1
40kD
IP: Homer1
Relative optical density
(mGluR1/Homer1)
3
2
1
0
*
Control
TNI

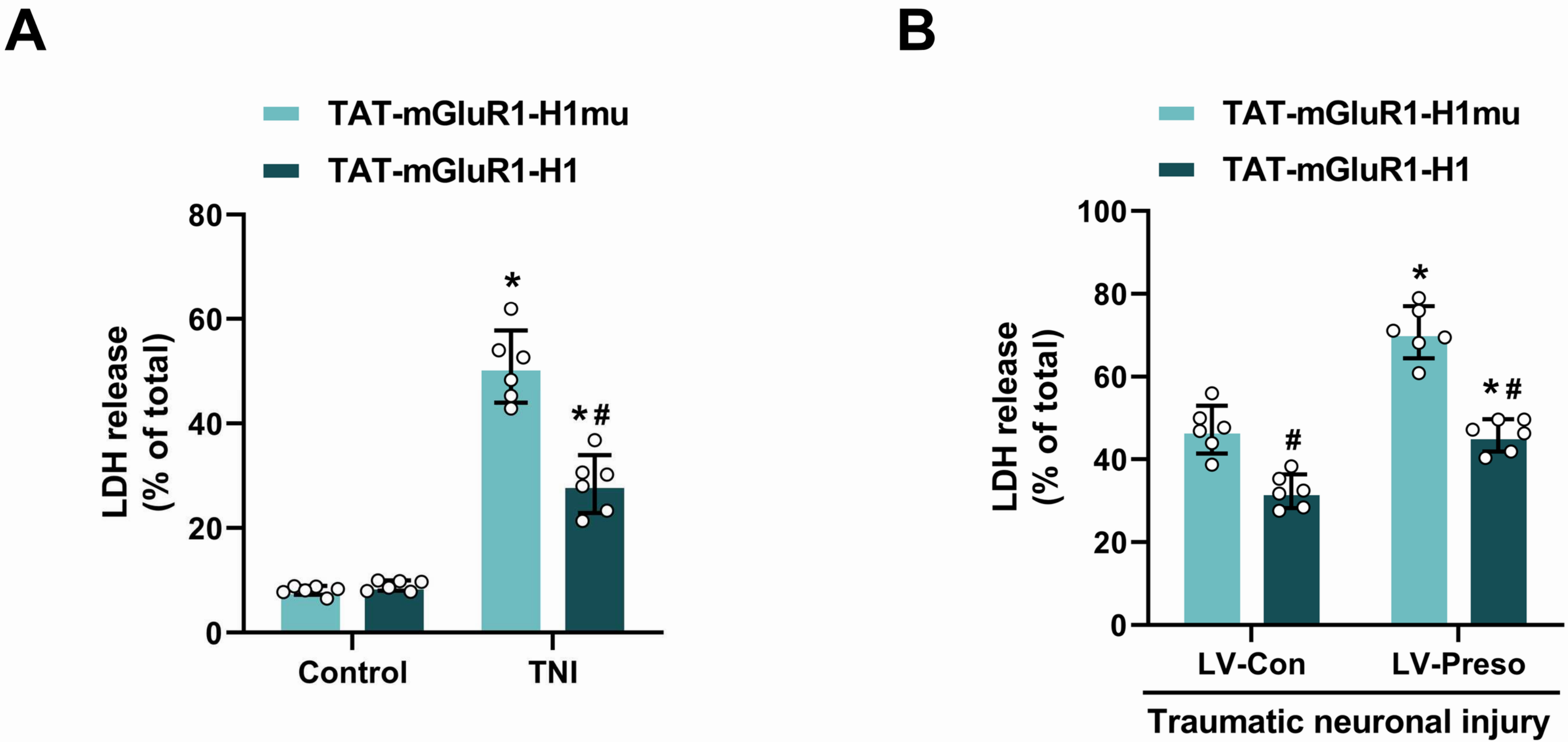
A
TAT-mGluR1-H1mu
TAT-mGluR1-H1
LDH release (% of total)
0
20
40
60
80
*
* #
Control
TNI
B
TAT-mGluR1-H1mu
TAT-mGluR1-H1
LDH release (% of total)
0
20
40
60
80
100
#
*
* #
LV-Con
LV-Preso
Traumatic neuronal injury

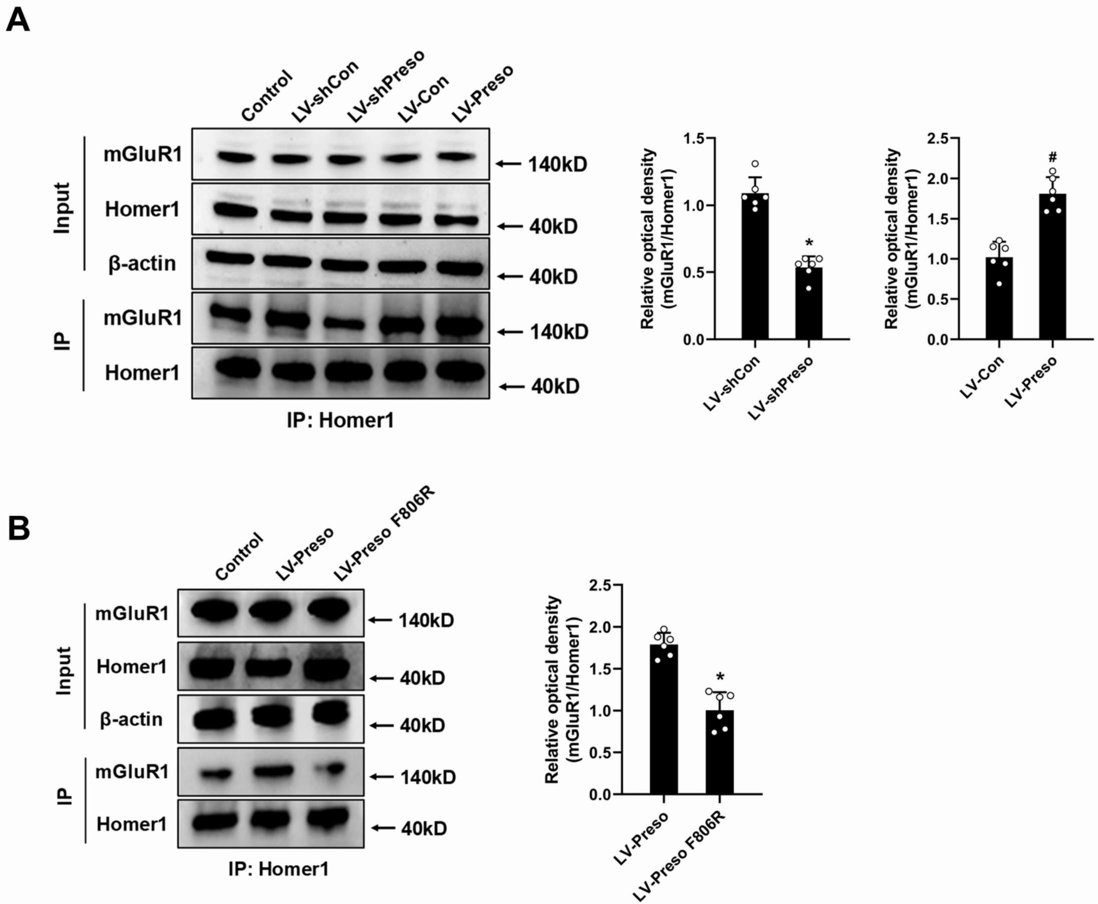
A
Control
LV-shCon
LV-shPreso
LV-Con
LV-Preso
Input
mGluR1
Homer1
β-actin
IP
mGluR1
Homer1
140kD
40kD
40kD
140kD
40kD
IP: Homer1
Relative optical density (mGluR1/Homer1)
1.5
1.0
0.5
0.0
*
LV-shCon
LV-shPreso
2.5
2.0
1.5
1.0
0.5
0.0
#
LV-Con
LV-Preso
B
Control
LV-Preso
LV-Preso F806R
Input
mGluR1
Homer1
β-actin
IP
mGluR1
Homer1
140kD
40kD
40kD
140kD
40kD
IP: Homer1
Relative optical density (mGluR1/Homer1)
2.5
2.0
1.5
1.0
0.5
0.0
*
LV-Preso
LV-Preso F806R

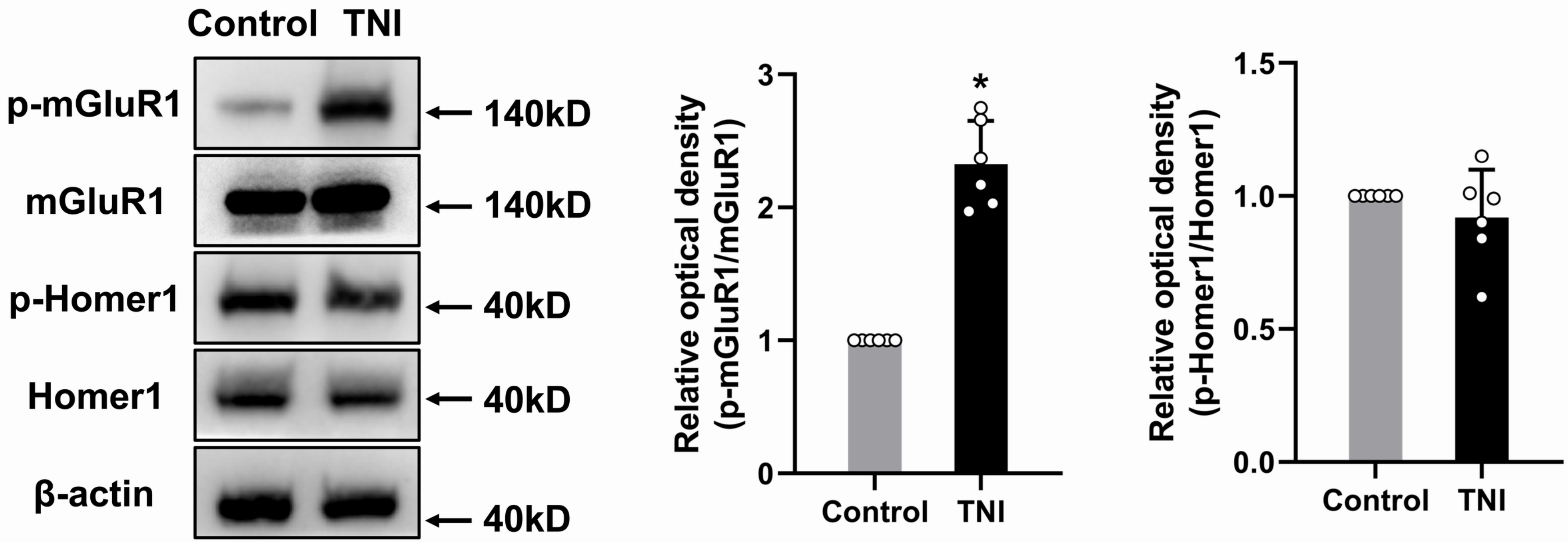

Control
TNI
p-mGluR1
140kD
mGluR1
140kD
p-Homer1
40kD
Homer1
40kD
β-actin
40kD
Relative optical density
(p-mGluR1/mGluR1)
3
2
1
0
*
Control
TNI
Relative optical density
(p-Homer1/Homer1)
1.5
1.0
0.5
0.0
Control
TNI

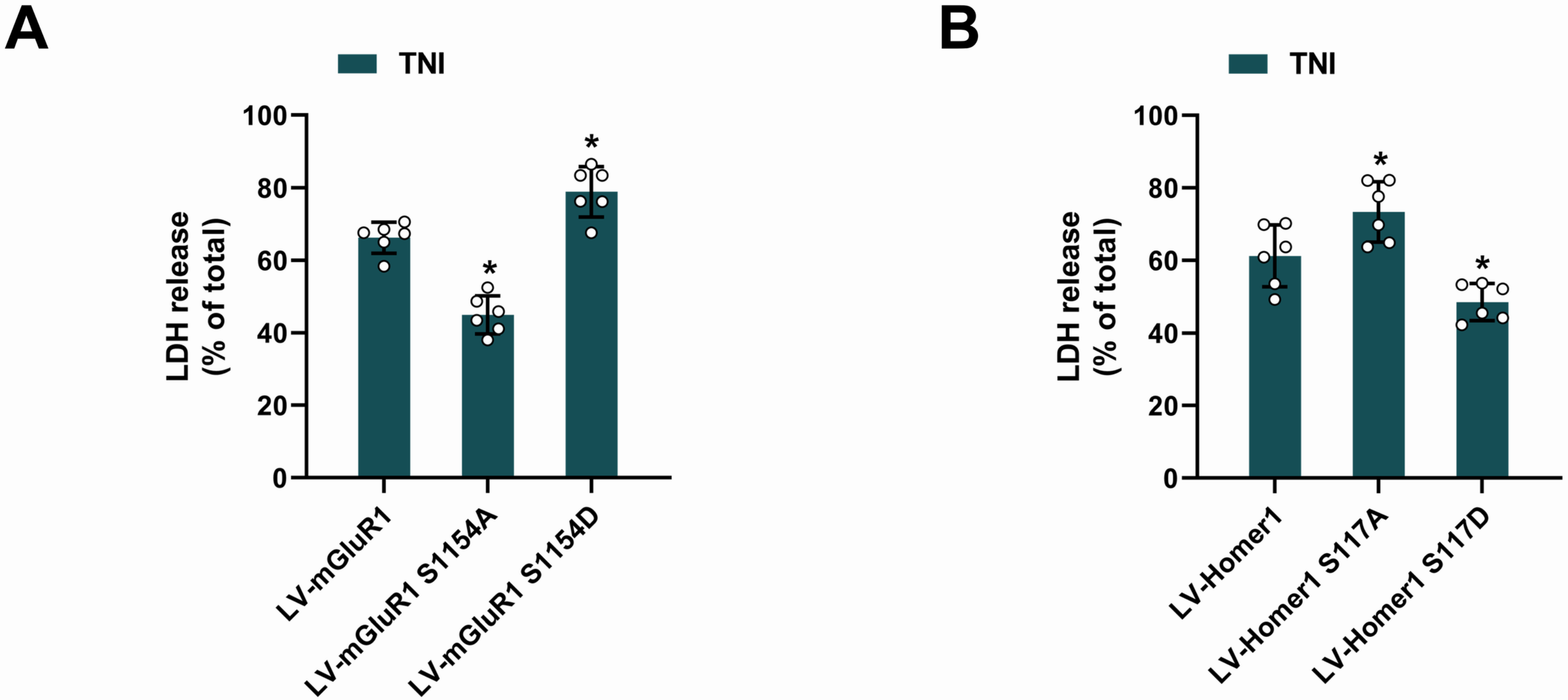

A
TNI
LDH release (% of total)
100
80
60
40
20
0
*
*
LV-mGluR1
LV-mGluR1 S1154A
LV-mGluR1 S1154D
B
TNI
LDH release (% of total)
100
80
60
40
20
0
*
*
LV-Homer1
LV-Homer1 S117A
LV-Homer1 S117D

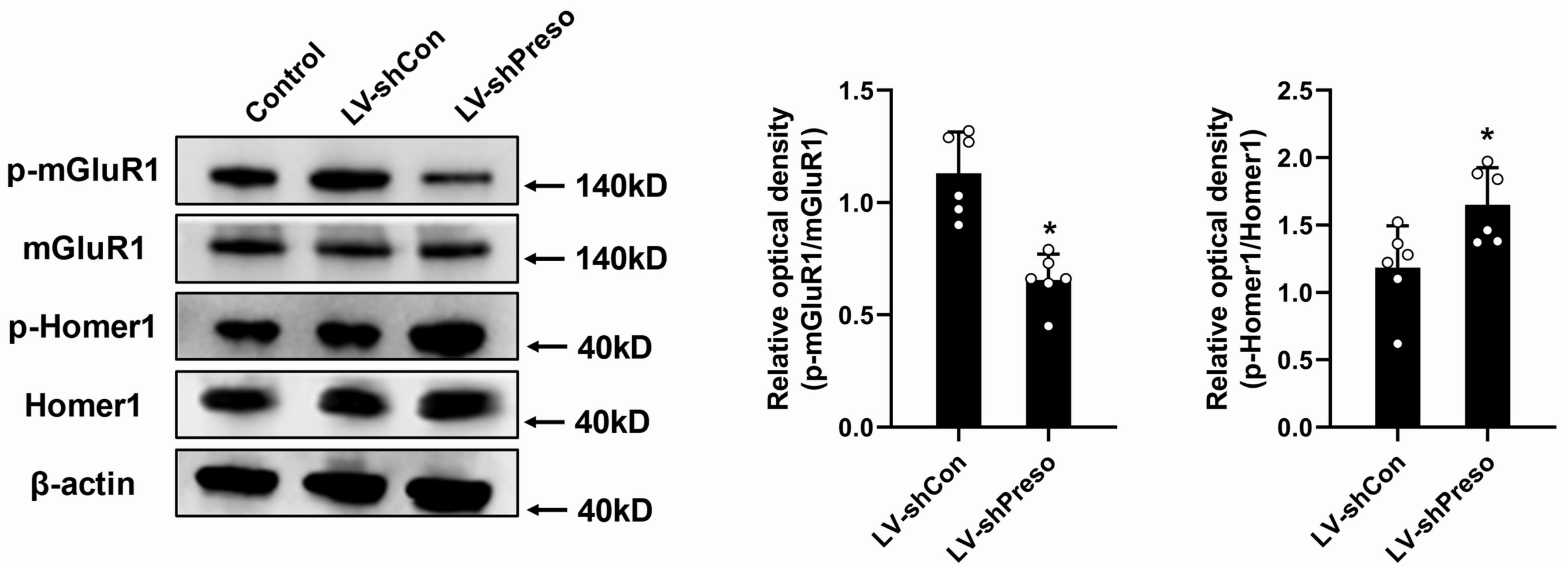

Control
LV-shCon
LV-shPreso
p-mGluR1
mGluR1
p-Homer1
Homer1
β-actin
140kD
140kD
40kD
40kD
40kD
Relative optical density (p-mGluR1/mGluR1)
1.5
1.0
0.5
0.0
*
LV-shCon
LV-shPreso
Relative optical density (p-Homer1/Homer1)
2.5
2.0
1.5
1.0
0.5
0.0
*
LV-shCon
LV-shPreso

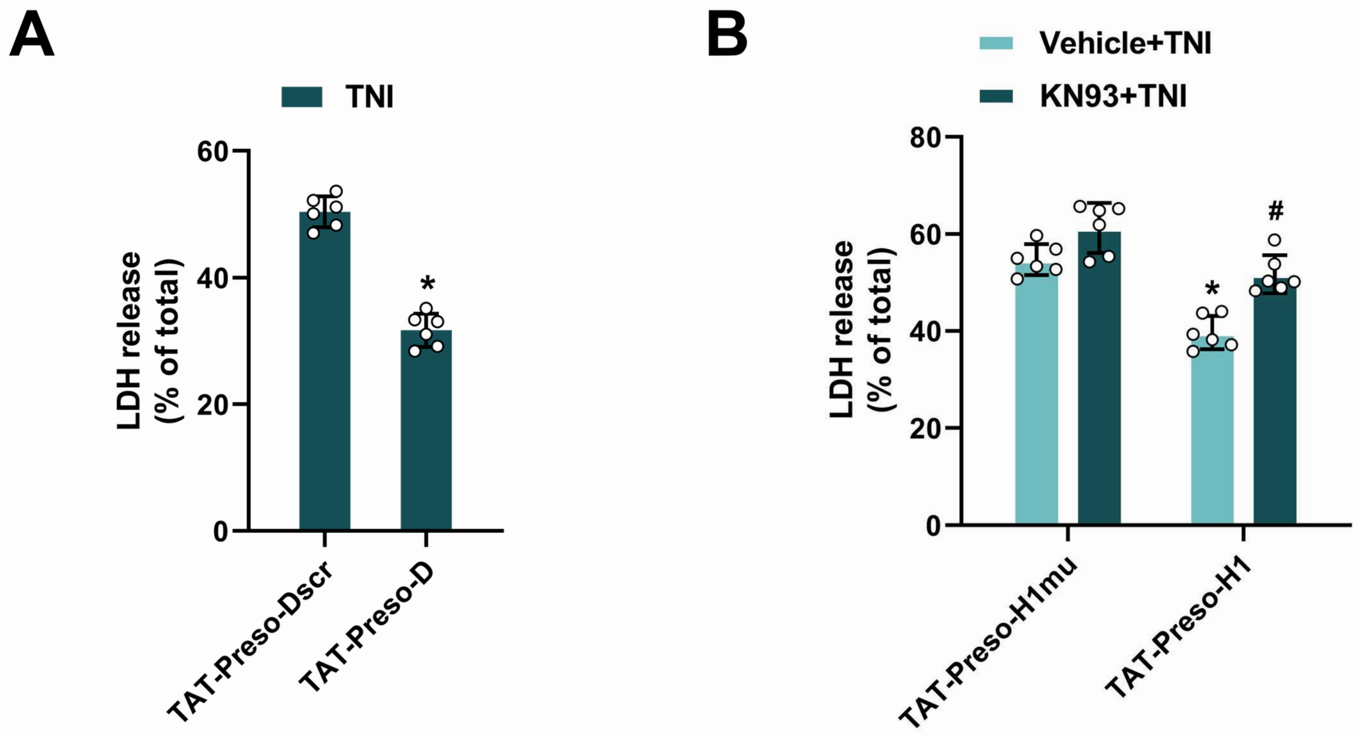
A
TNI
LDH release
(% of total)
60
40
20
0
*
TAT-Preso-Dscr
TAT-Preso-D
B
Vehicle+TNI
KN93+TNI
80
60
40
20
0
*
#
TAT-Preso-H1mu
TAT-Preso-H1

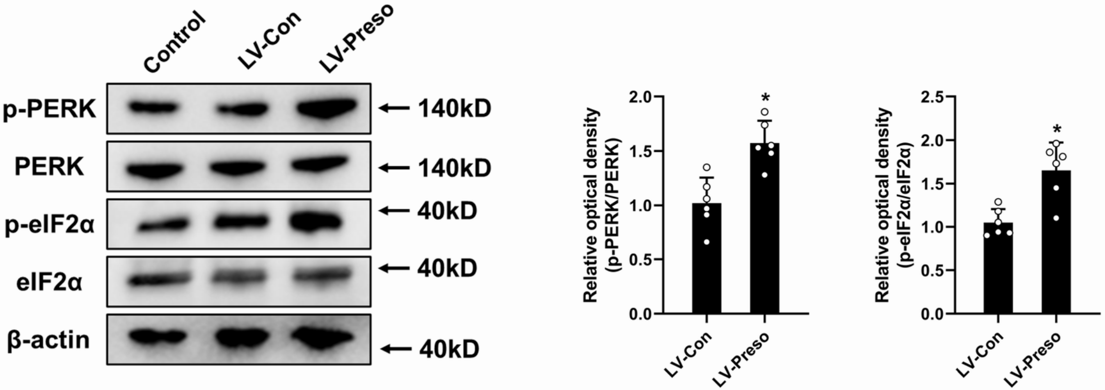
Control
LV-Con
LV-Preso
p-PERK
PERK
p-eIF2α
eIF2α
β-actin
140kD
140kD
40kD
40kD
40kD
Relative optical density (p-PERK/PERK)
Relative optical density (p-eIF2α/eIF2α)
LV-Con
LV-Preso
*

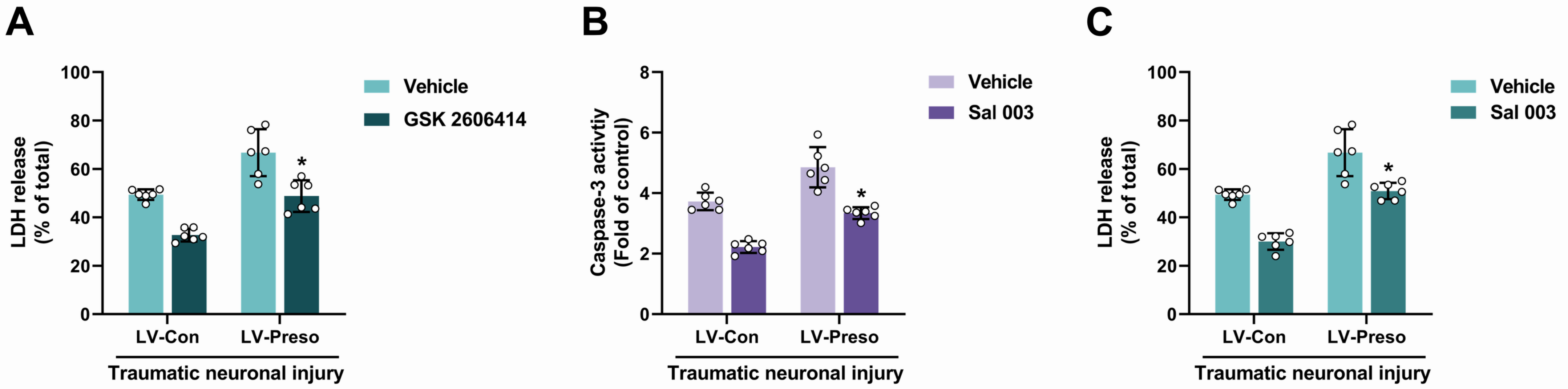
A
LDH release
(% of total)
100
80
60
40
20
0
Vehicle
GSK 2606414
*
LV-Con
LV-Preso
Traumatic neuronal injury
B
Caspase-3 activtiy
(Fold of control)
8
6
4
2
0
Vehicle
Sal 003
*
LV-Con
LV-Preso
Traumatic neuronal injury
C
LDH release
(% of total)
100
80
60
40
20
0
Vehicle
Sal 003
*
LV-Con
LV-Preso
Traumatic neuronal injury

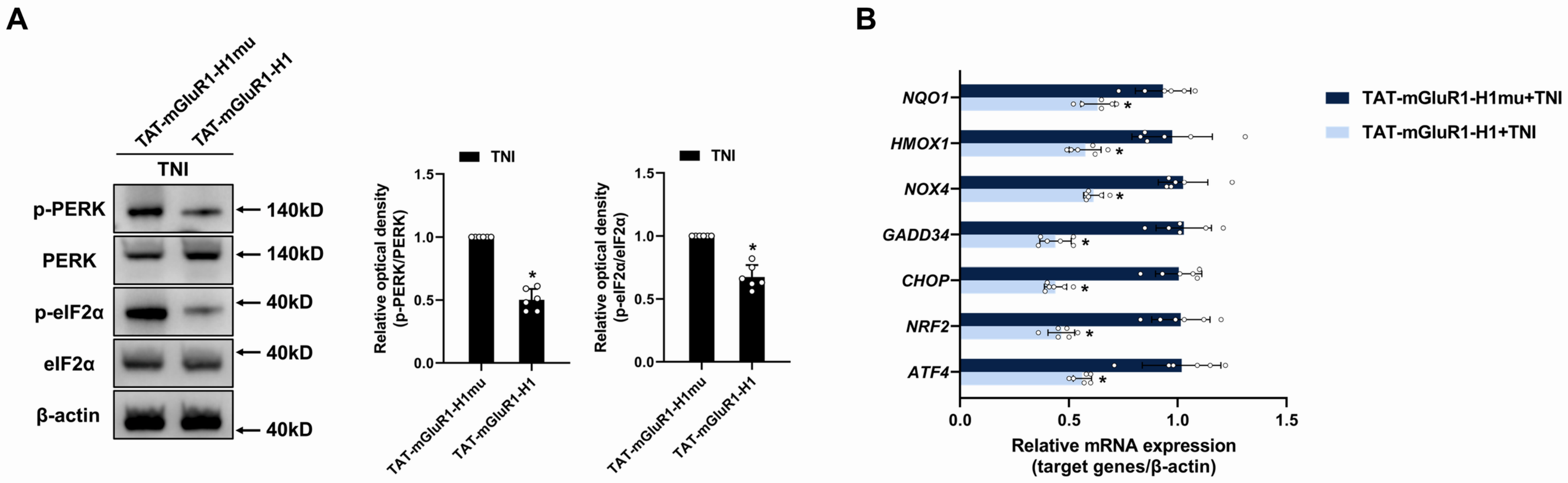

A
TAT-mGluR1-H1mu
TAT-mGluR1-H1
TNI
p-PERK
140kD
PERK
140kD
p-eIF2α
40kD
eIF2α
40kD
β-actin
40kD
TNI
Relative optical density (p-PERK/PERK)
1.5
1.0
0.5
0.0
TAT-mGluR1-H1mu
TAT-mGluR1-H1
TNI
Relative optical density (p-eIF2α/eIF2α)
B
NQO1
HMOX1
NOX4
GADD34
CHOP
NRF2
ATF4
TAT-mGluR1-H1mu+TNI
TAT-mGluR1-H1+TNI
0.0
0.5
1.0
1.5
Relative mRNA expression (target genes/β-actin)

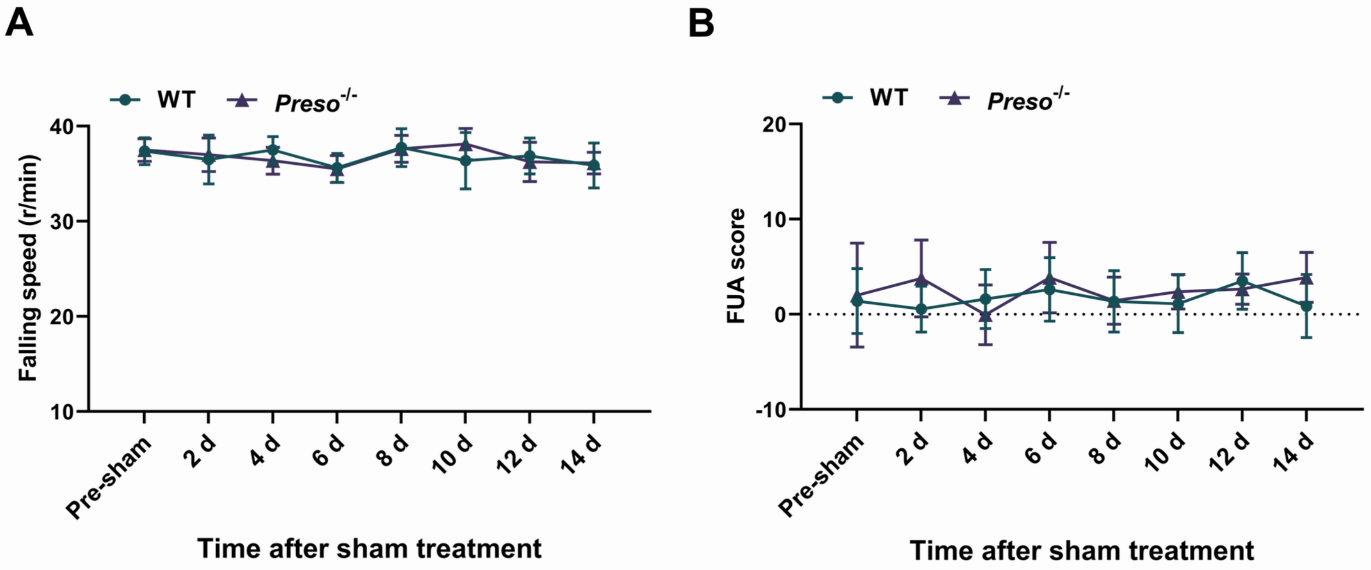

A
WT
Preso-/-
Falling speed (r/min)
40
30
20
10
Pre-sham
2 d
4 d
6 d
8 d
10 d
12 d
14 d
Time after sham treatment
B
WT
Preso-/-
FUA score
20
10
0
-10
Pre-sham
2 d
4 d
6 d
8 d
10 d
12 d
14 d
Time after sham treatment

A

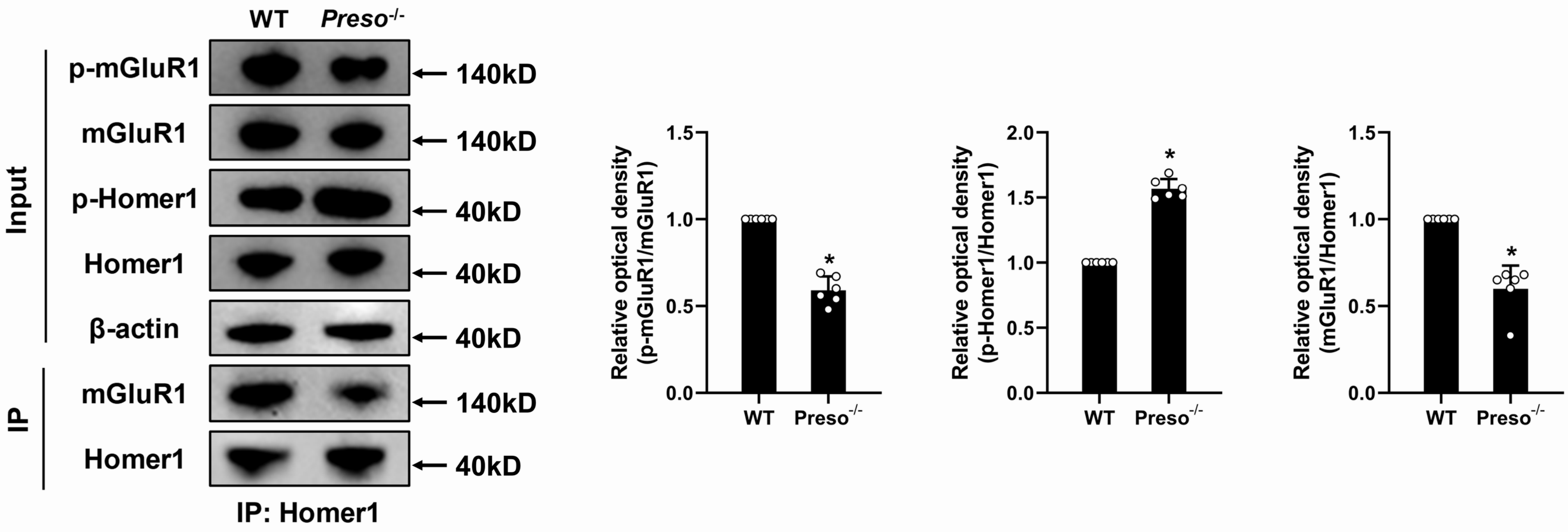
WT
Preso-/-
Input
IP
p-mGluR1
mGluR1
p-Homer1
Homer1
β-actin
mGluR1
Homer1
140kD
140kD
40kD
40kD
40kD
140kD
40kD
IP: Homer1
Relative optical density (p-mGluR1/mGluR1)
Relative optical density (p-Homer1/Homer1)
Relative optical density (mGluR1/Homer1)
0.0
0.5
1.0
1.5
2.0
WT
Preso-/-
*

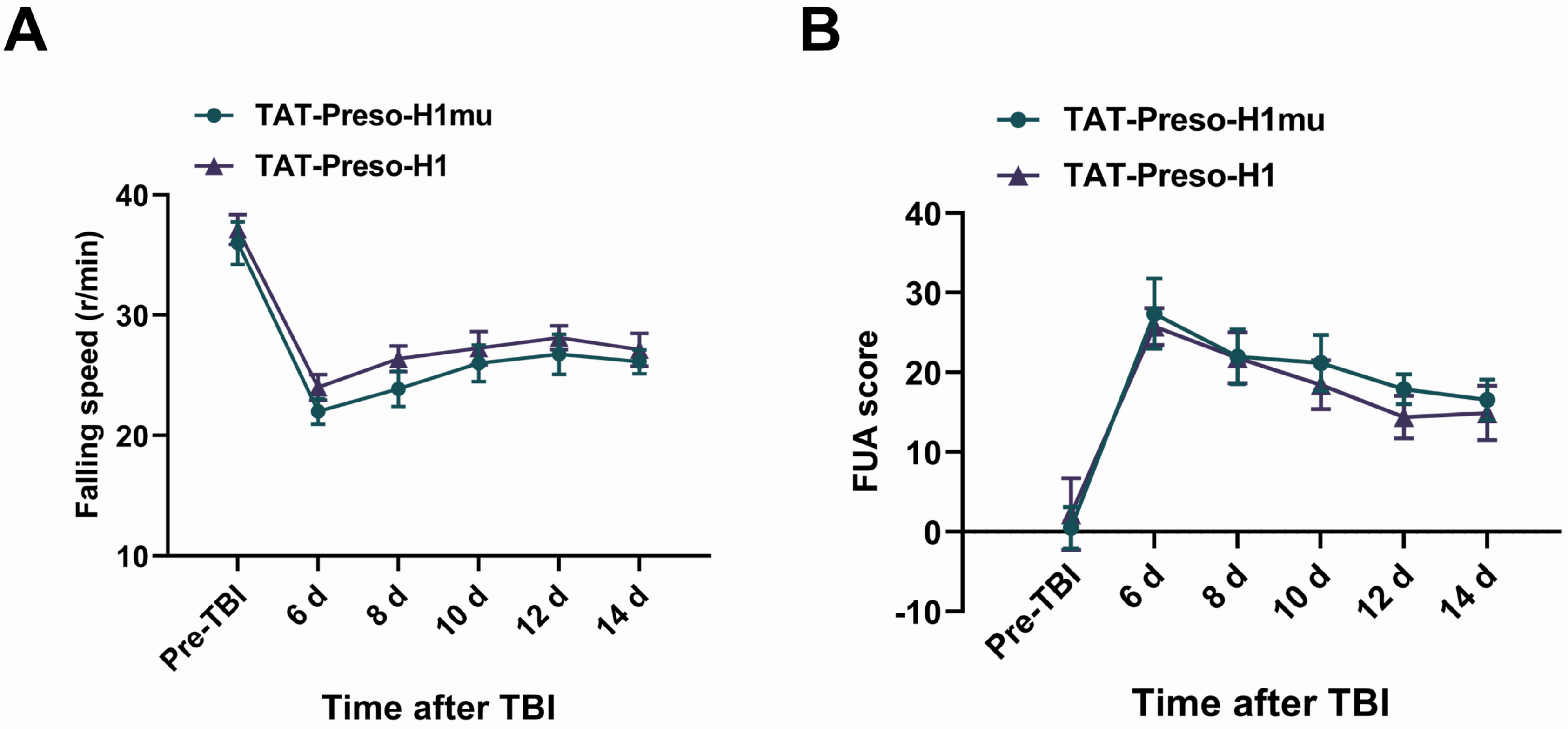

A
TAT-Preso-H1mu
TAT-Preso-H1
Falling speed (r/min)
40
30
20
10
Pre-TBI
6 d
8 d
10 d
12 d
14 d
Time after TBI
B
TAT-Preso-H1mu
TAT-Preso-H1
FUA score
40
30
20
10
0
-10
Pre-TBI
6 d
8 d
10 d
12 d
14 d
Time after TBI

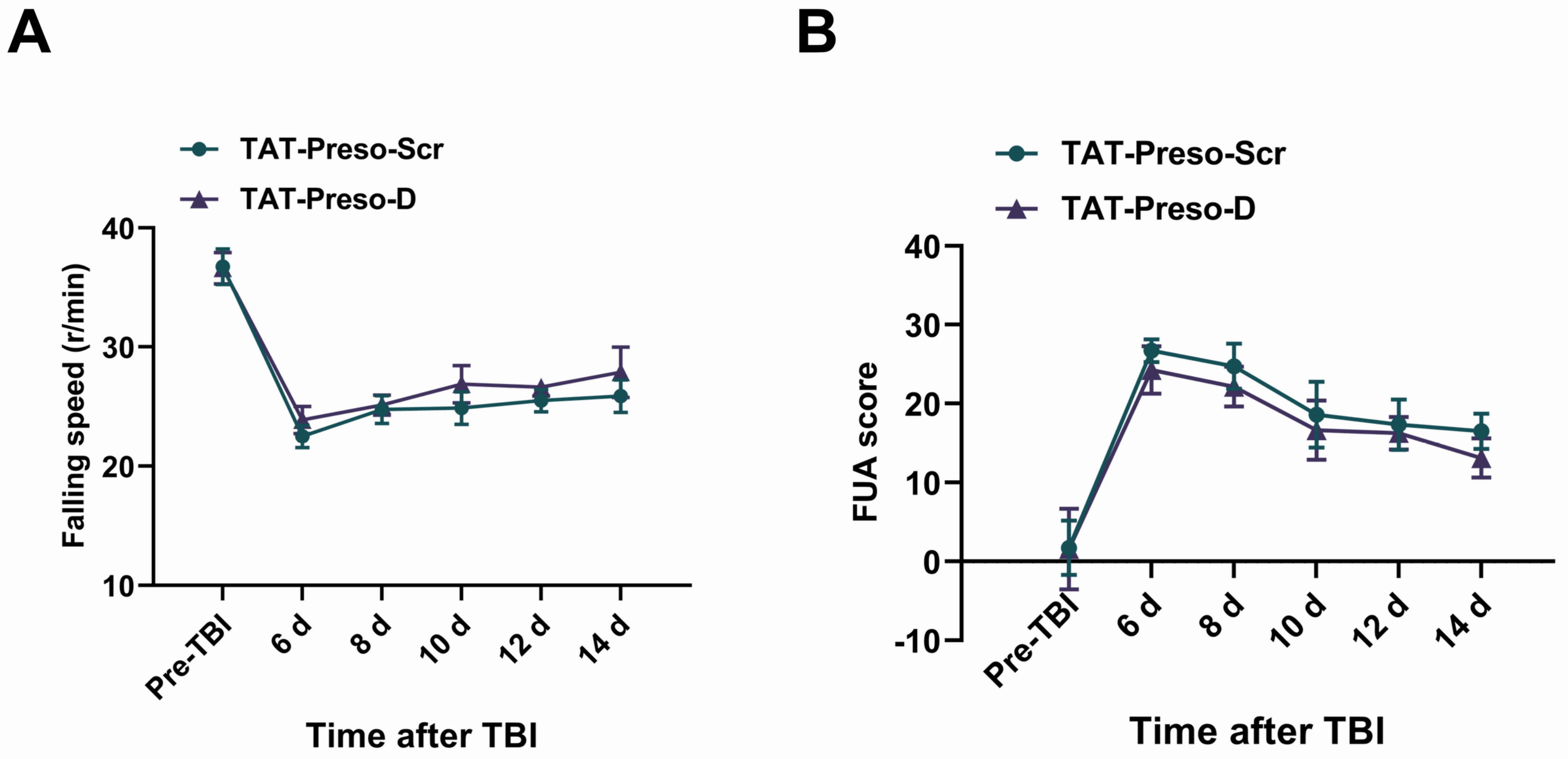
A
TAT-Preso-Scr
TAT-Preso-D
Falling speed (r/min)
40
30
20
10
Pre-TBI
6 d
8 d
10 d
12 d
14 d
Time after TBI
B
TAT-Preso-Scr
TAT-Preso-D
FUA score
40
30
20
10
0
-10
Pre-TBI
6 d
8 d
10 d
12 d
14 d
Time after TBI

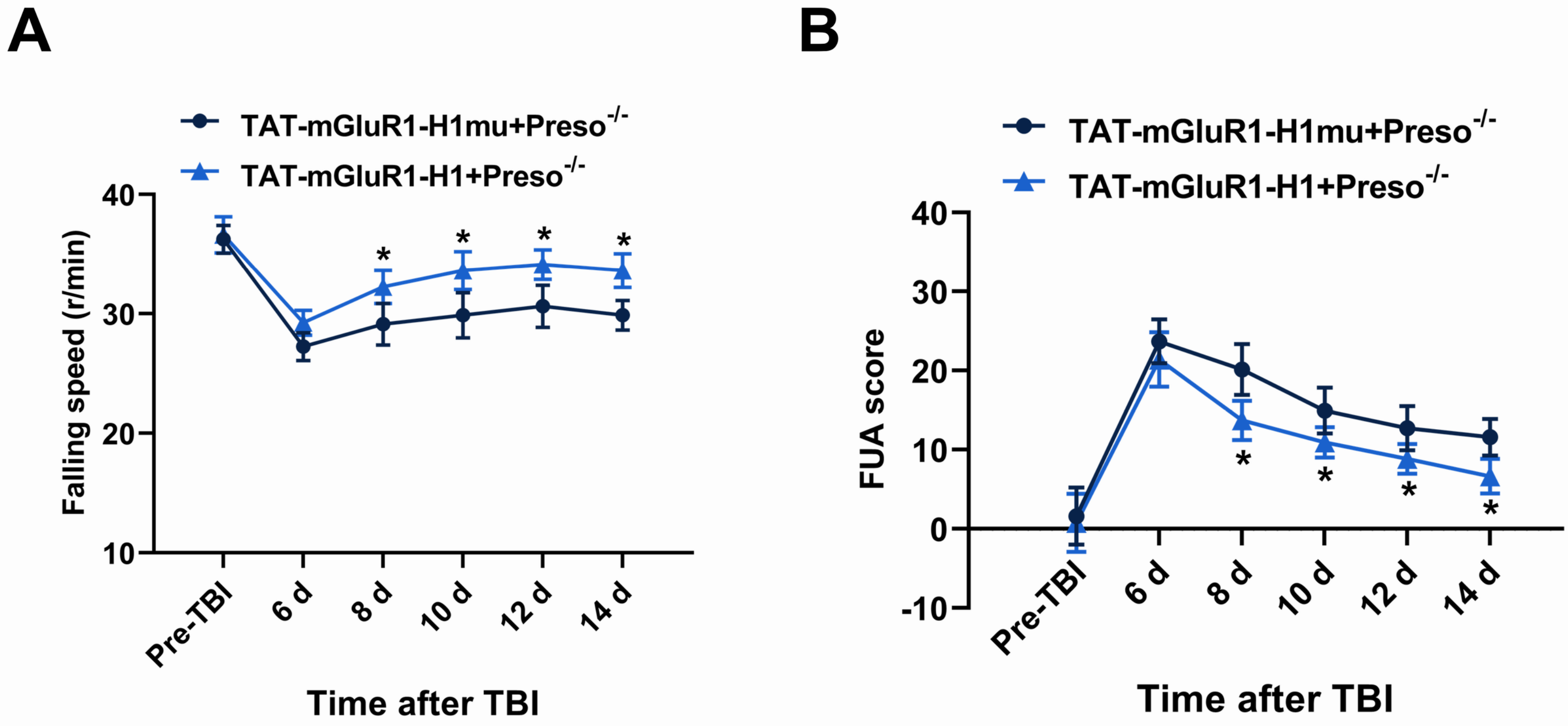
A
B
TAT-mGluR1-H1mu+Preso-/-
TAT-mGluR1-H1+Preso-/-
Falling speed (r/min)
40
30
20
10
Pre-TBI
6 d
8 d
10 d
12 d
14 d
Time after TBI
*
FUA score
0
-10
Time after TBI
