# Supplementary material for: Preso enhances mGluR1-mediated excitotoxicity by modulating the phosphorylation of mGluR1-Homer1 complex and facilitating an ER stress after traumatic brain injury
Source: Cell Death Discov. 2024 Mar 26;10:153. doi: 10.1038/s41420-024-01916-5 (PMC10965980; doi:10.1038/s41420-024-01916-5)
Supplement: Supplementary file 2 — Original Data File [file 41420_2024_1916_MOESM2_ESM.pdf]

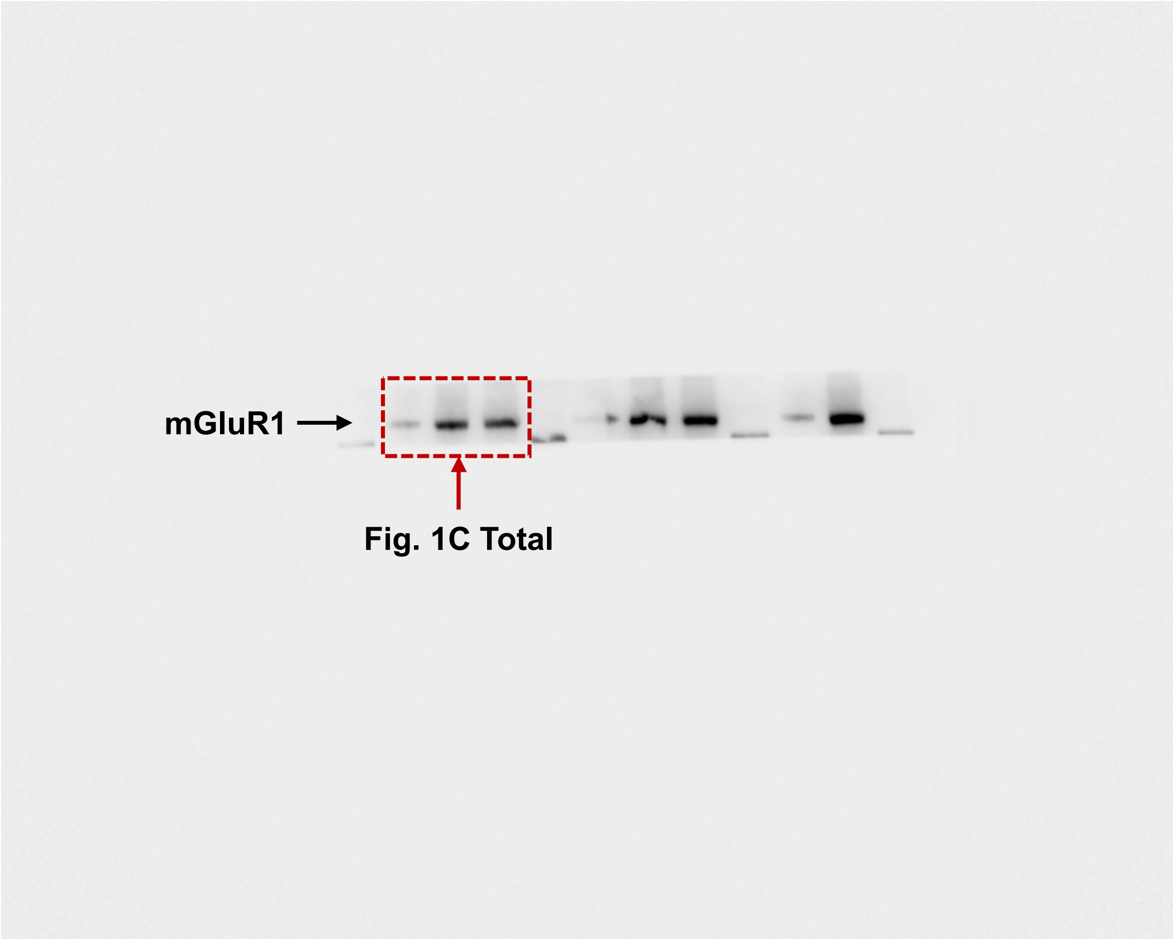
mGluR1
Fig. 1C Total

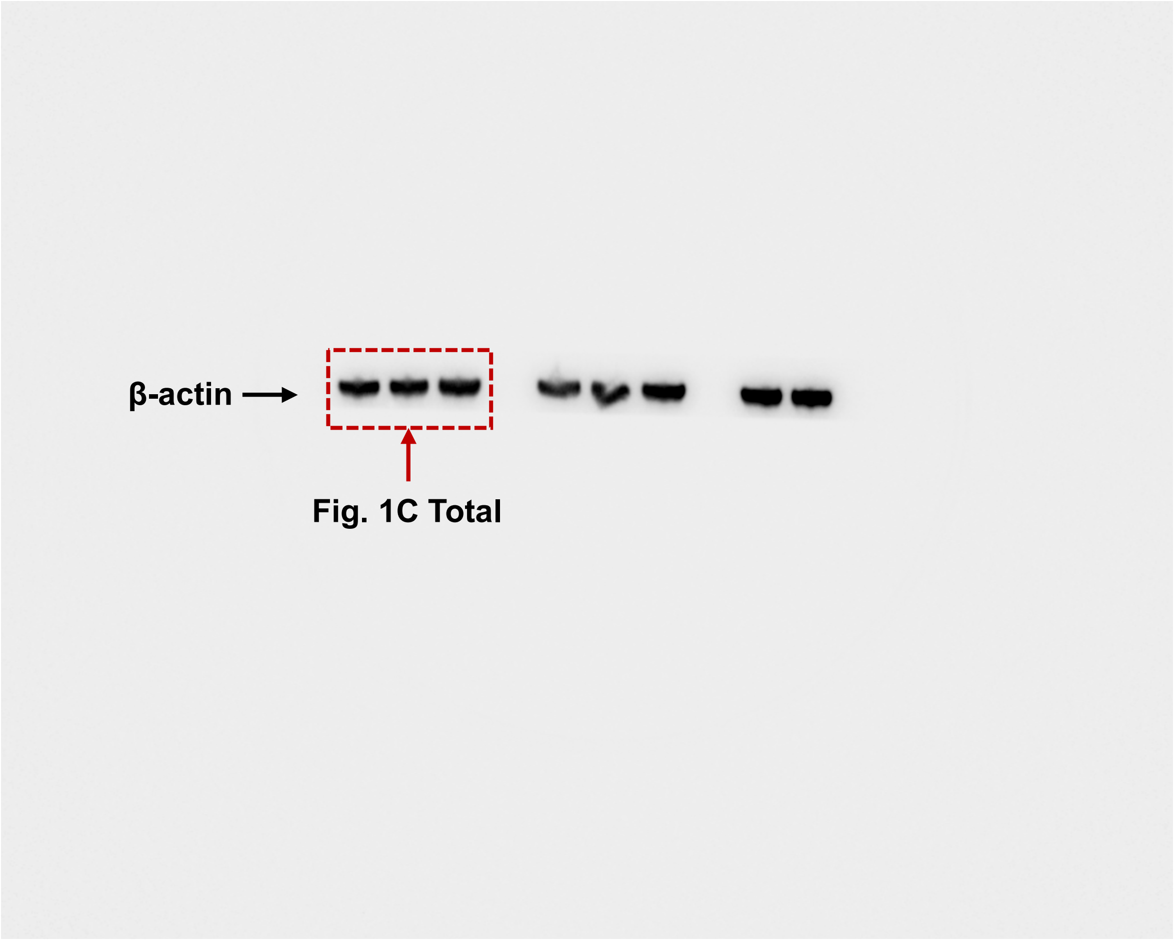

β-actin
Fig. 1C Total

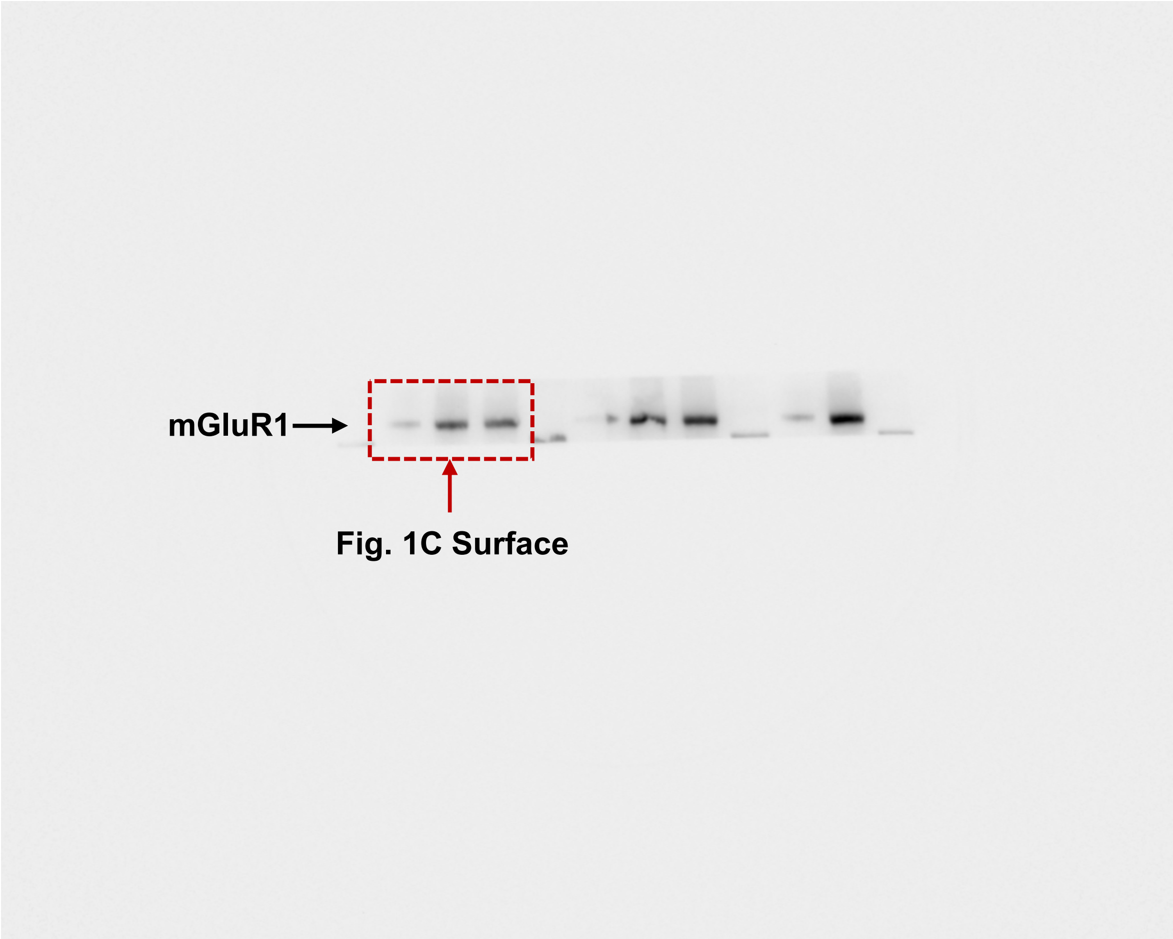

mGluR1
Fig. 1C Surface

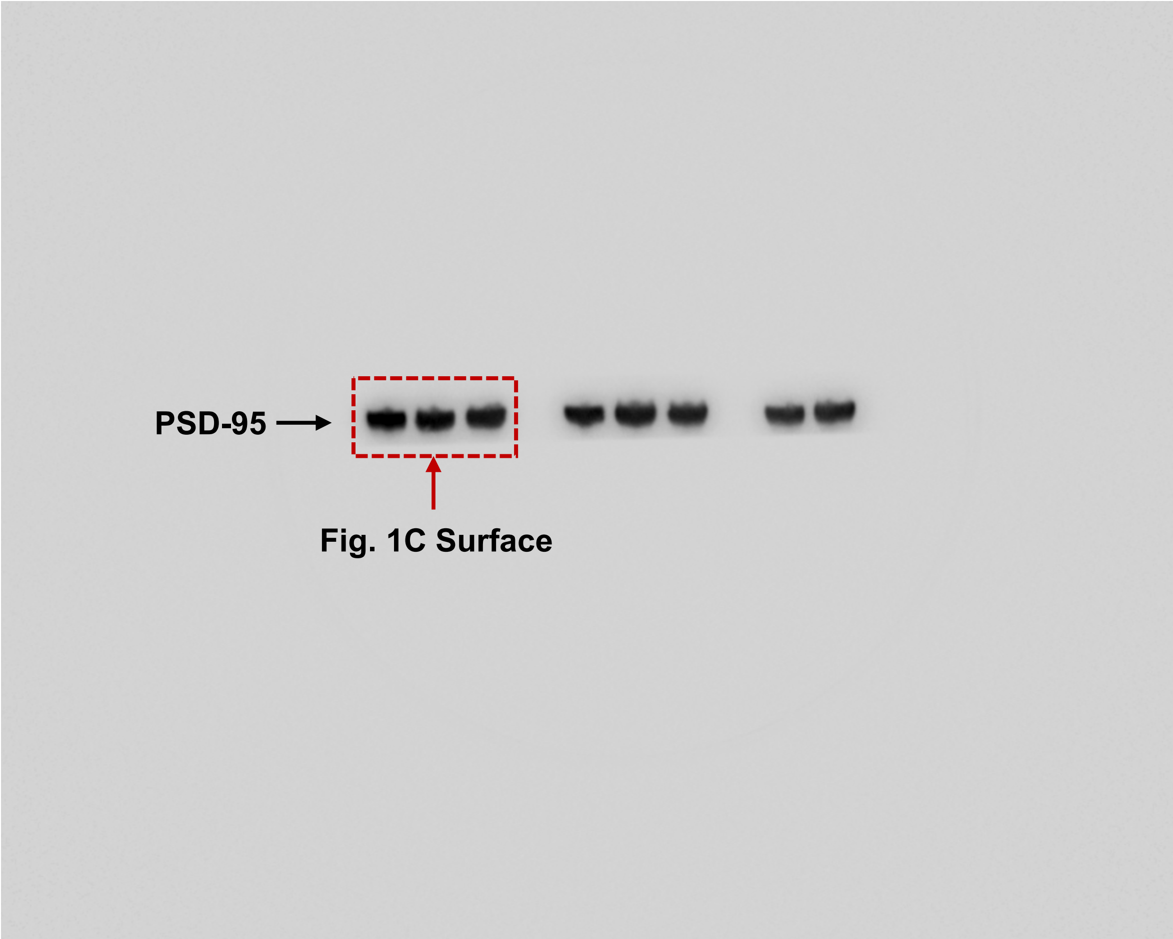
PSD-95
Fig. 1C Surface

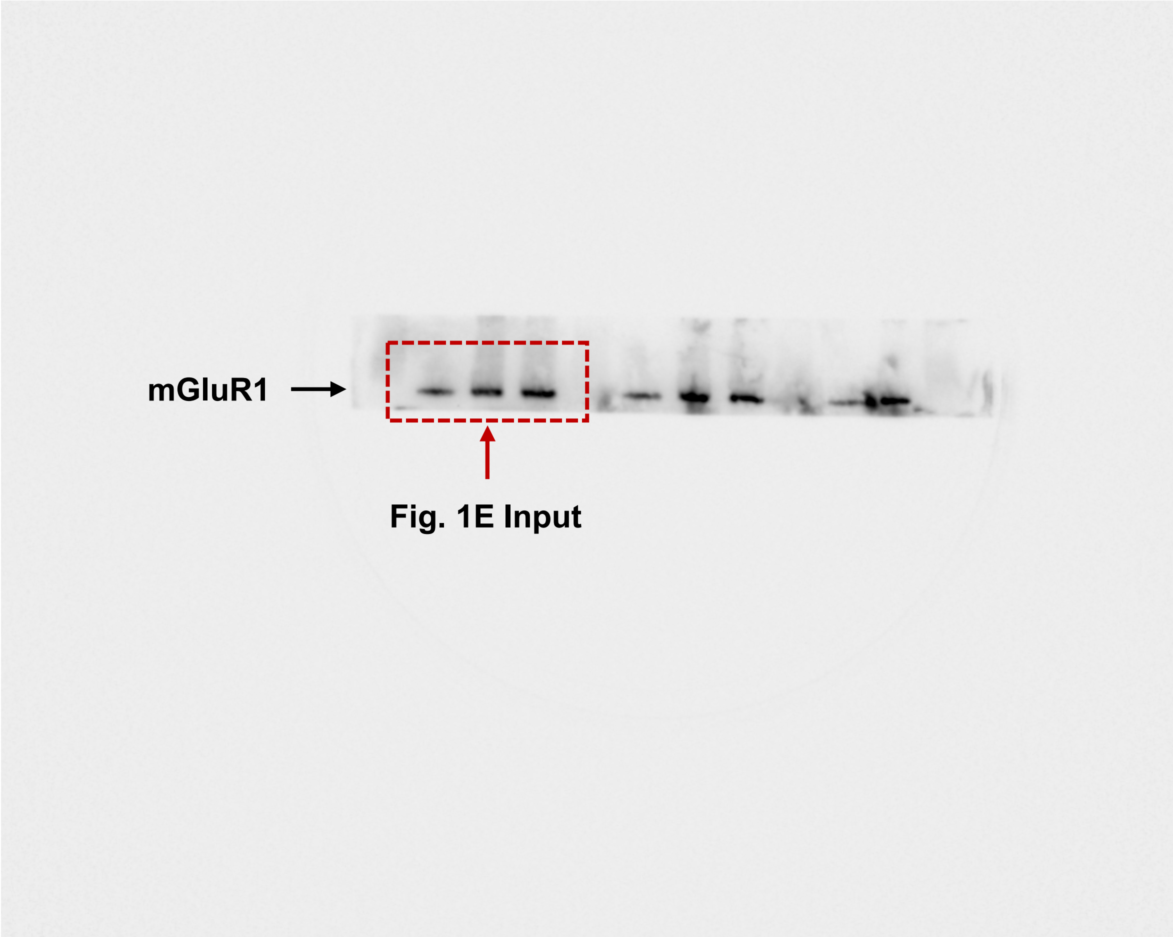
mGluR1
Fig. 1E Input

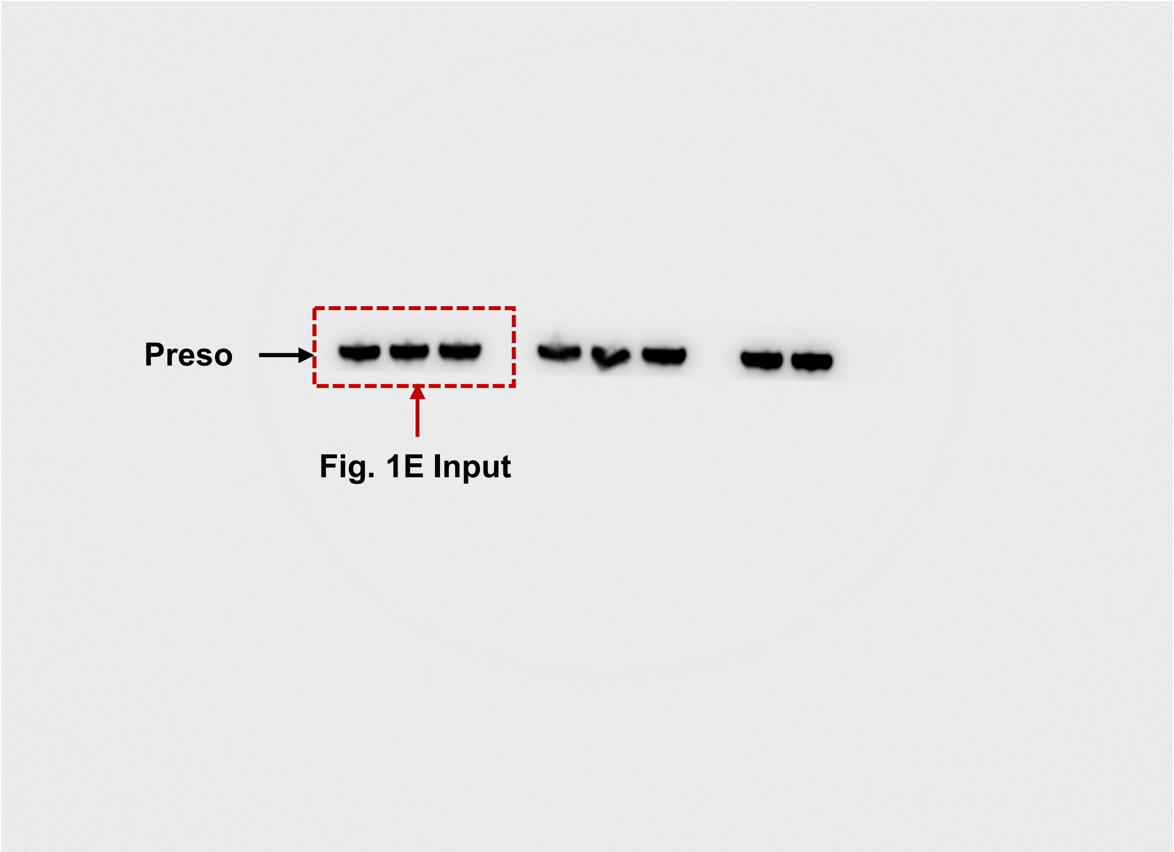
Preso
Fig. 1E Input

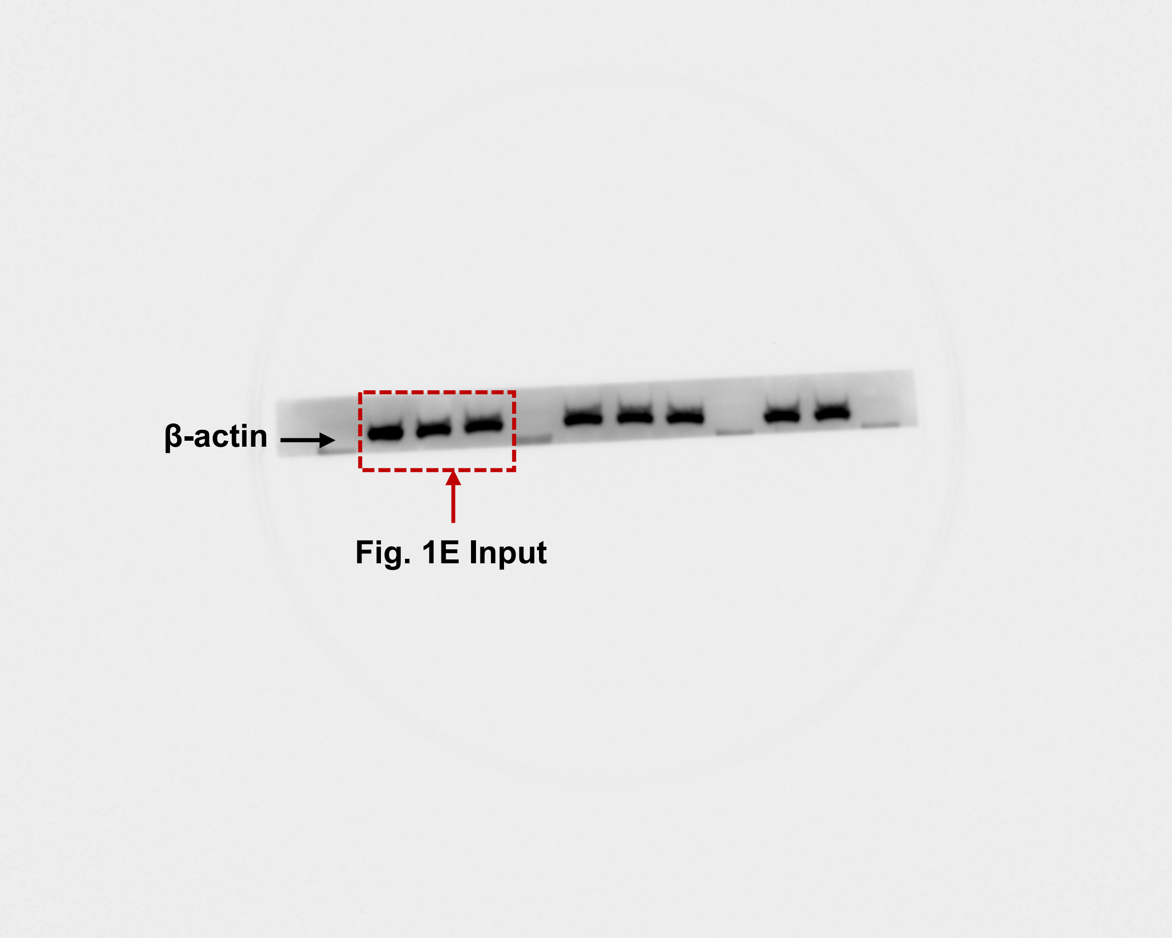
β-actin
Fig. 1E Input

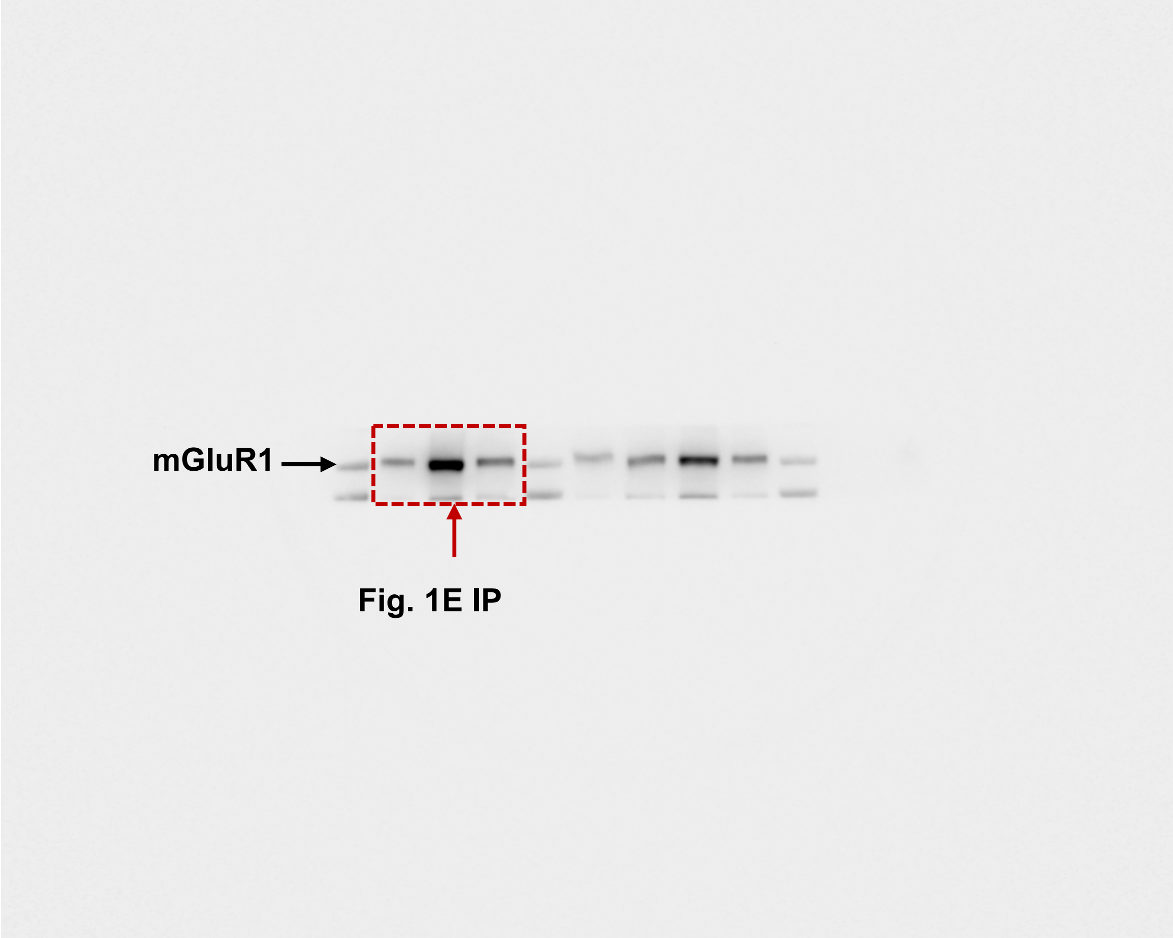
mGluR1
Fig. 1E IP

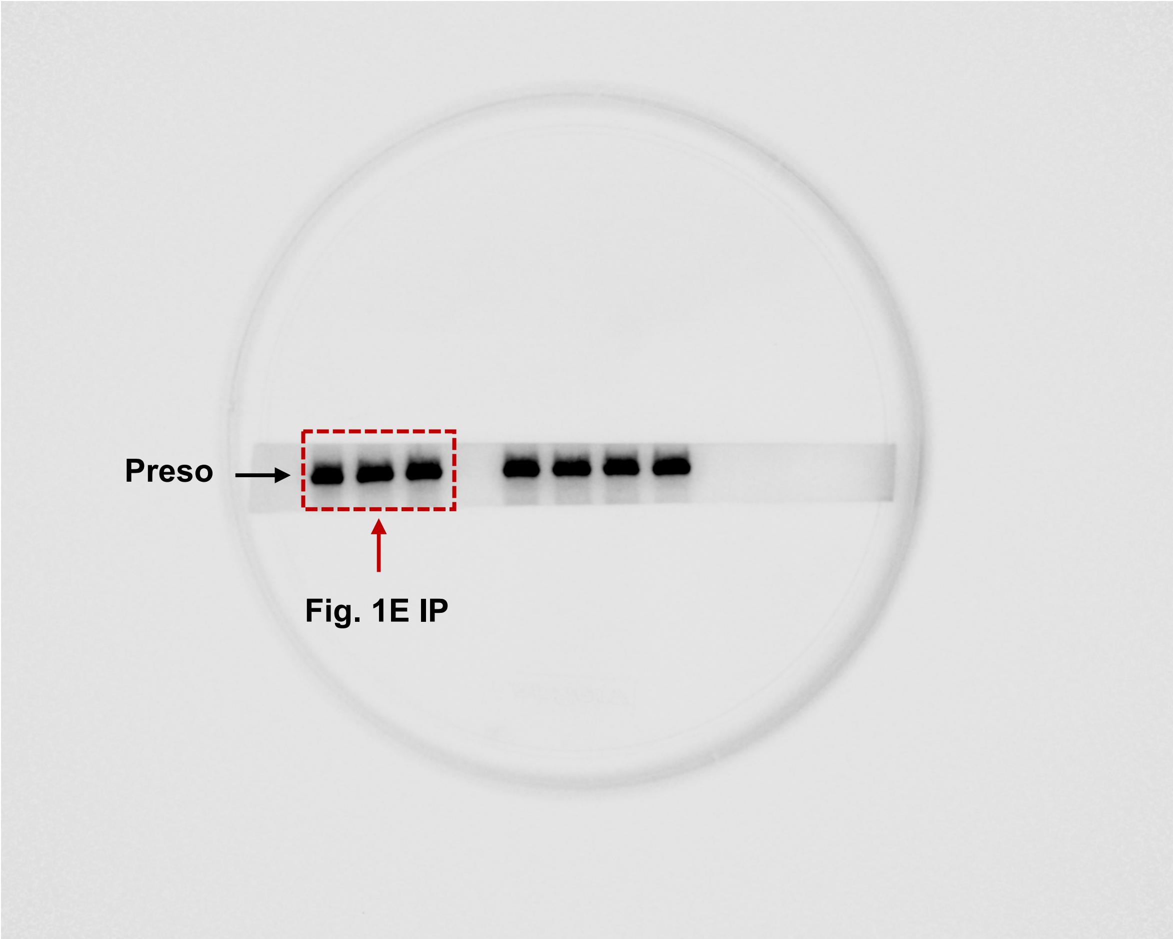
Preso
Fig. 1E IP

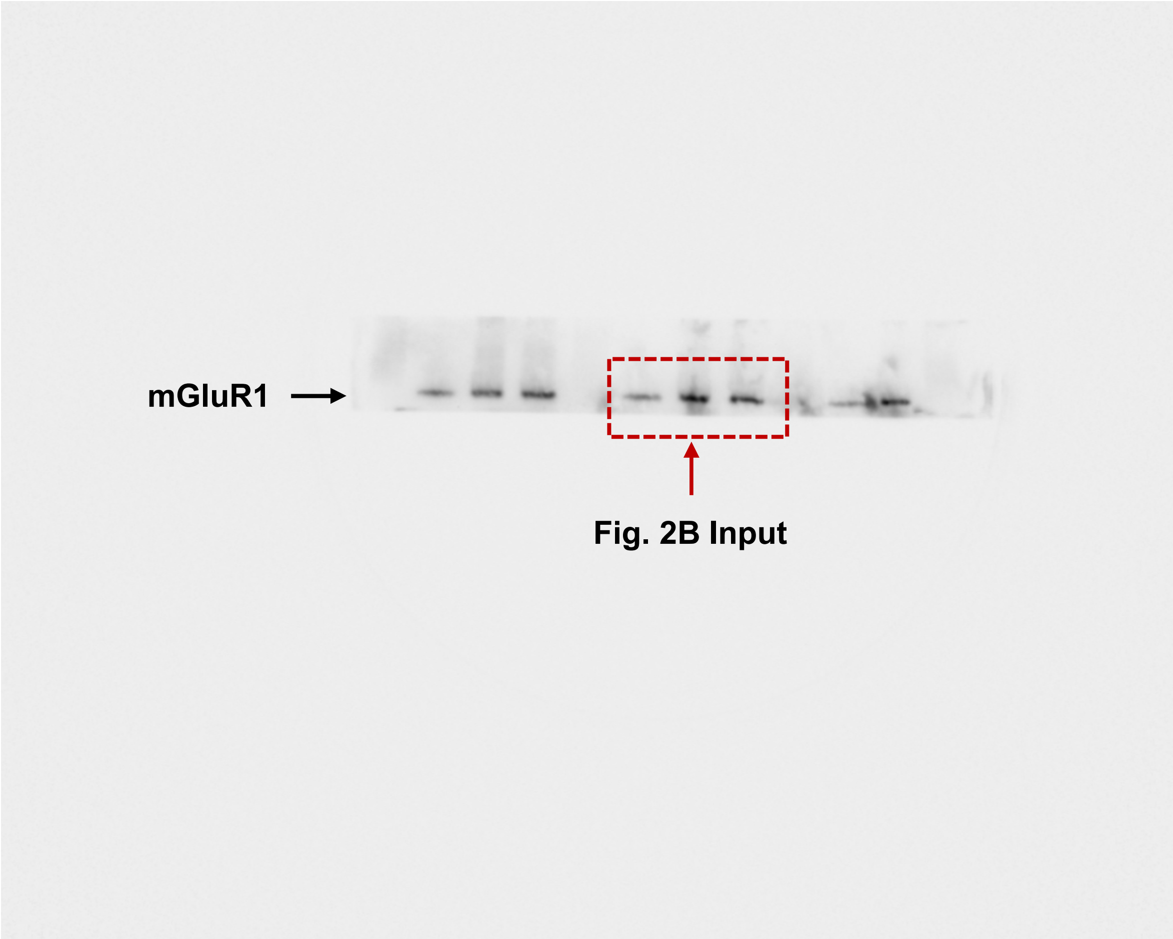
mGluR1
Fig. 2B Input

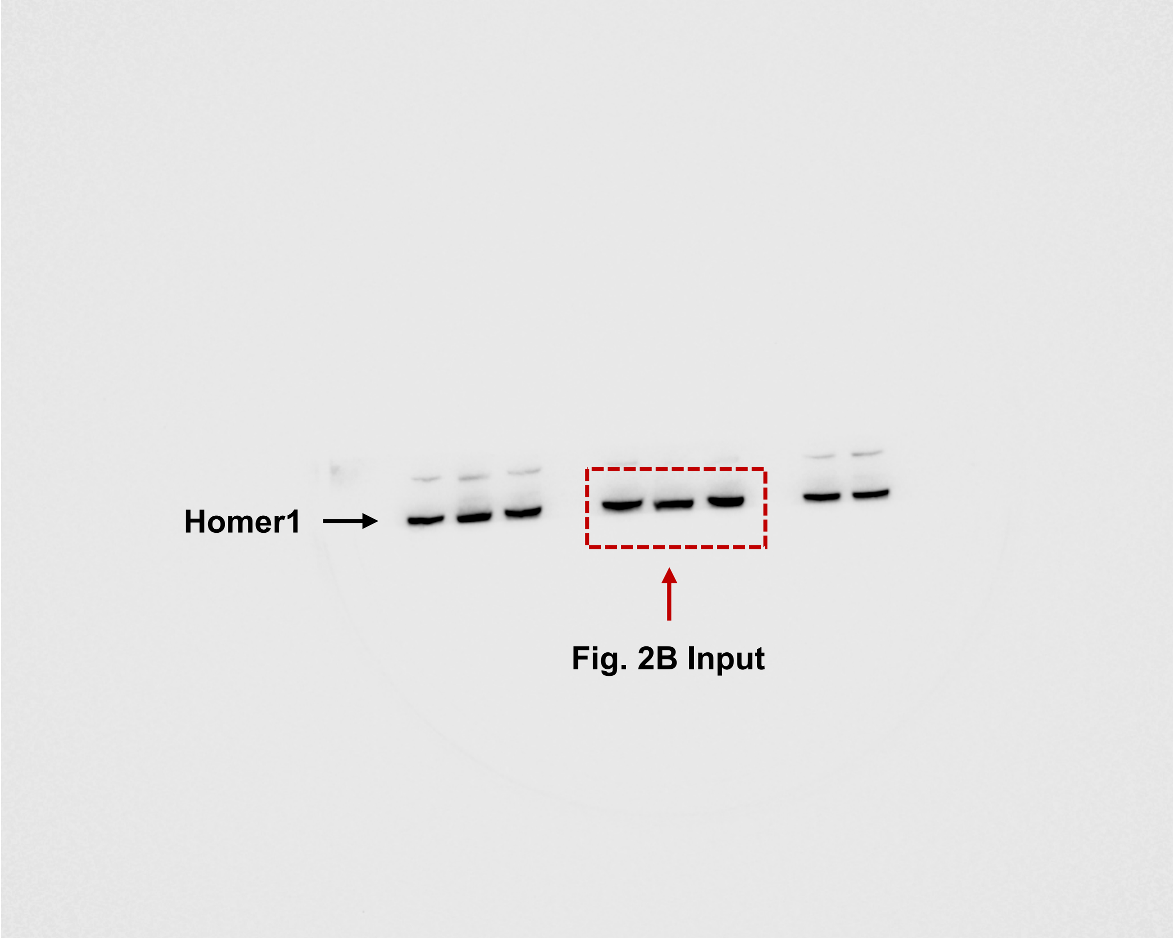
Homer1
Fig. 2B Input

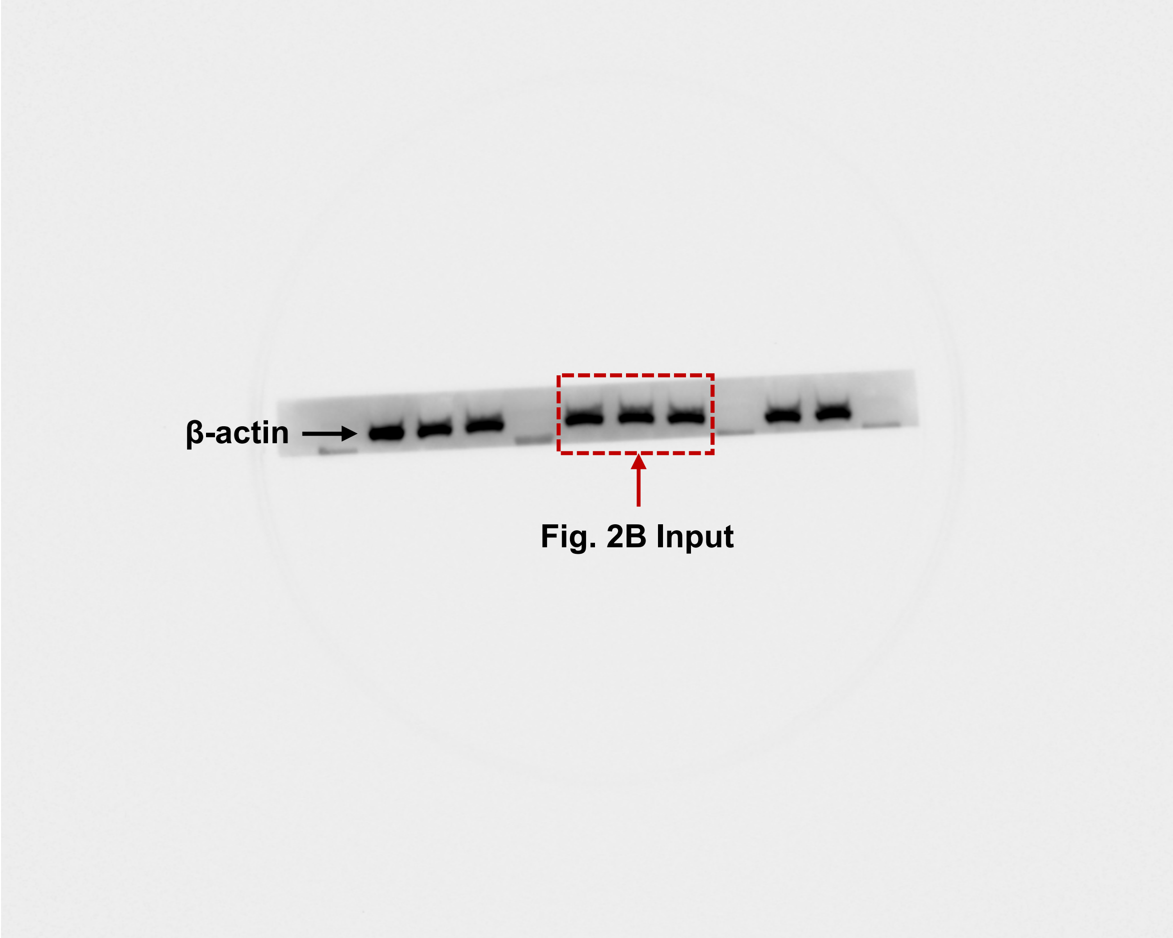
β-actin
Fig. 2B Input

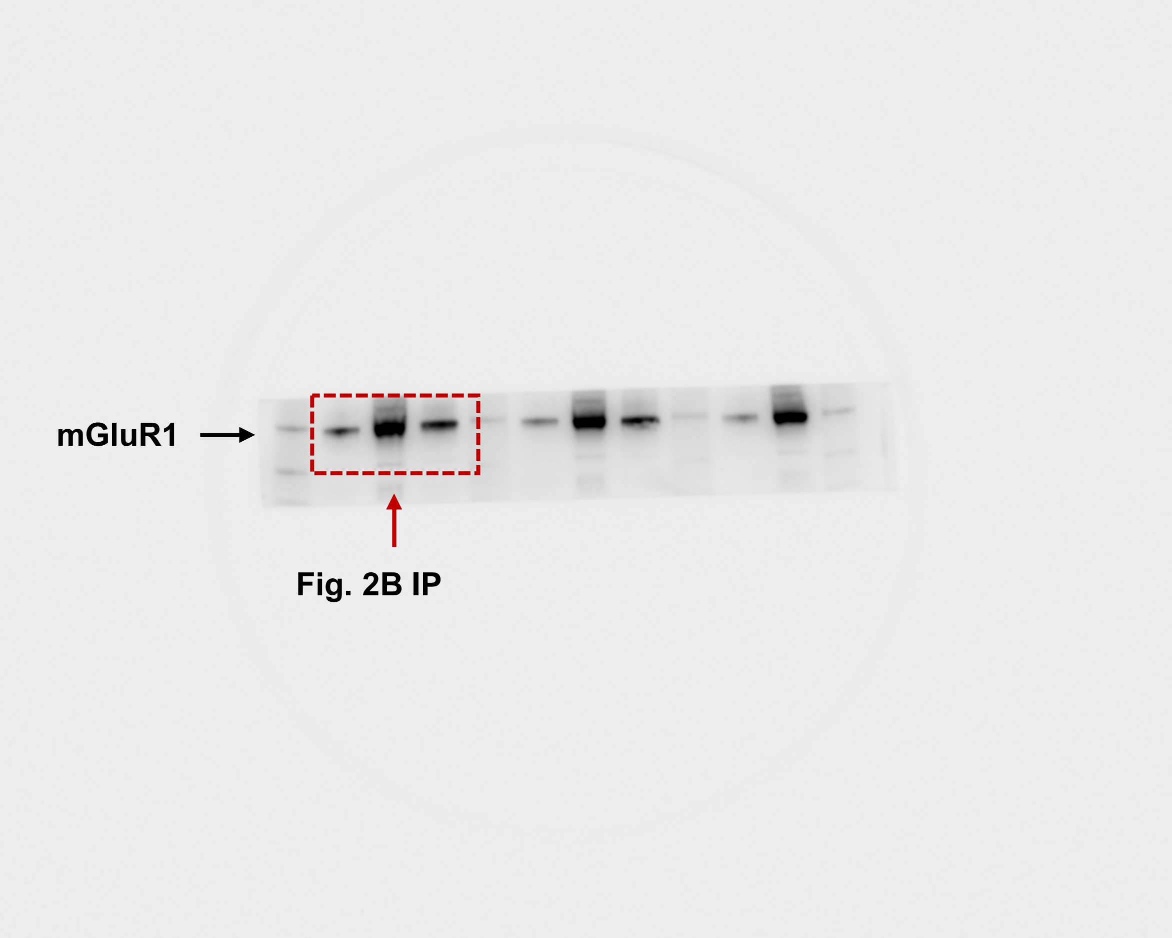
mGluR1
Fig. 2B IP

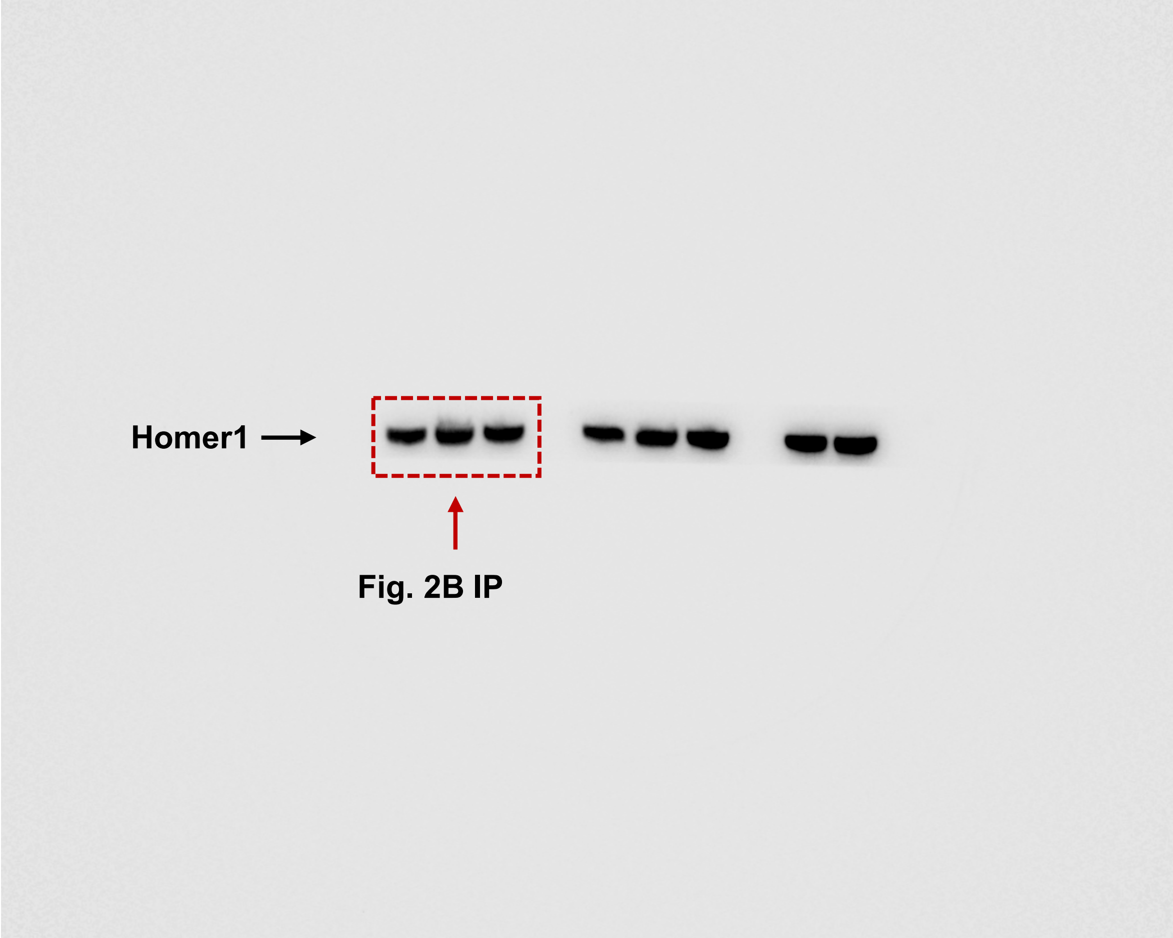
Homer1
Fig. 2B IP

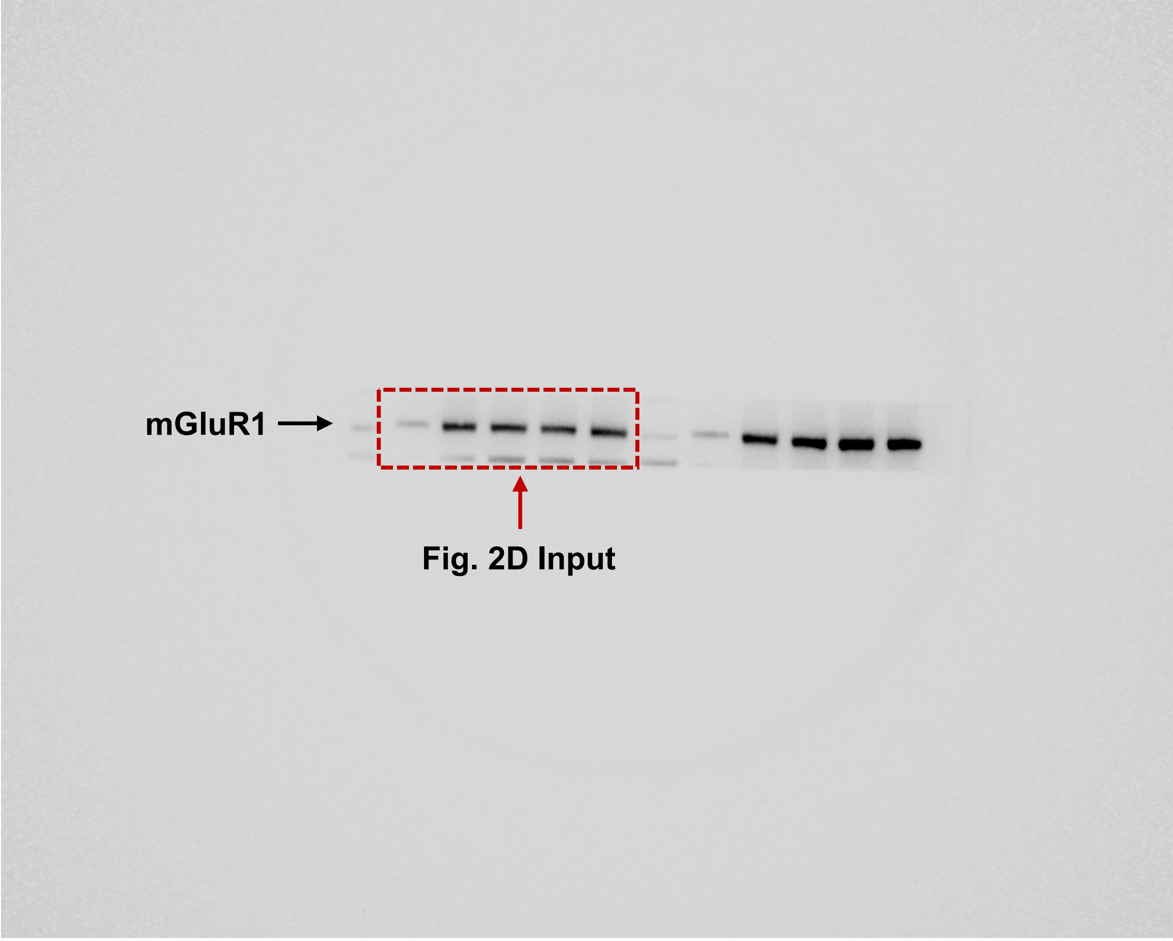
mGluR1
Fig. 2D Input

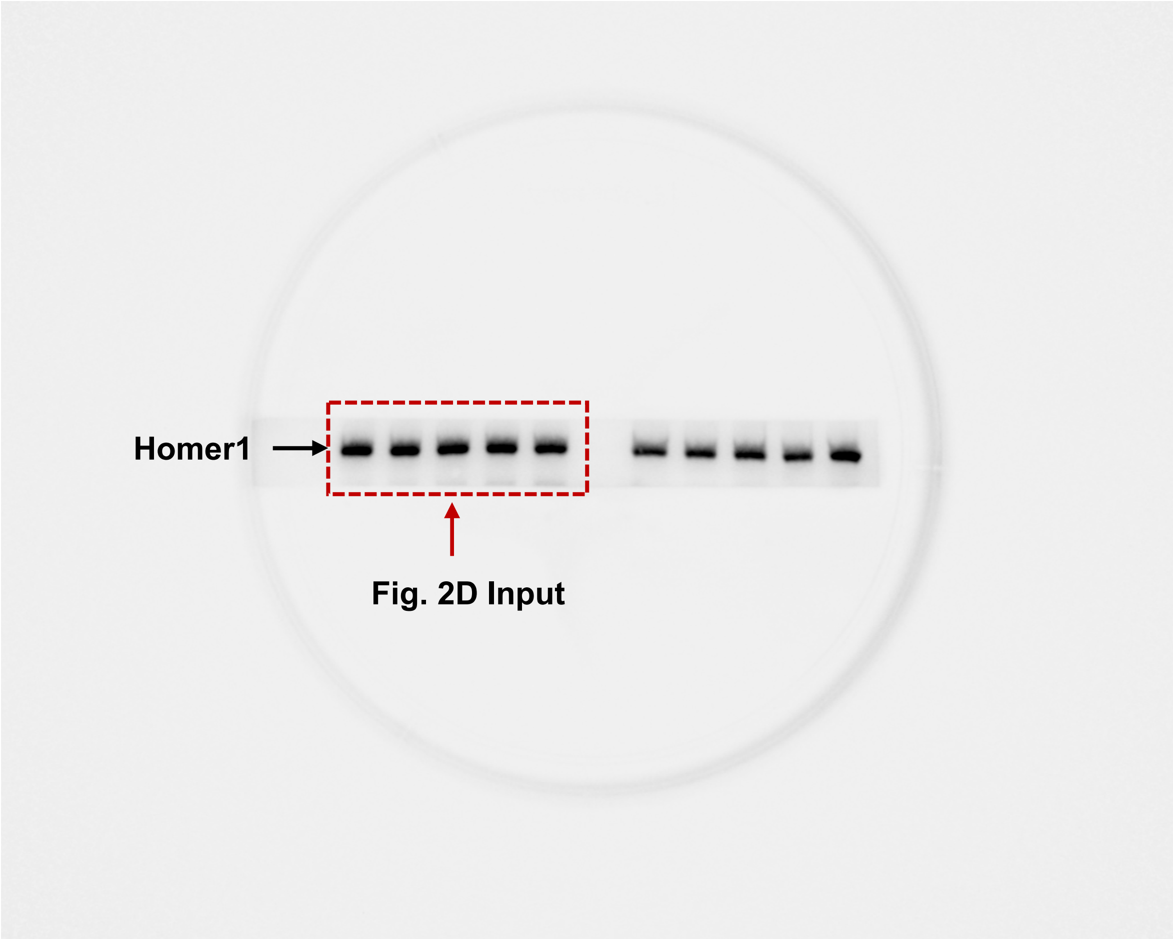
Homer1
Fig. 2D Input

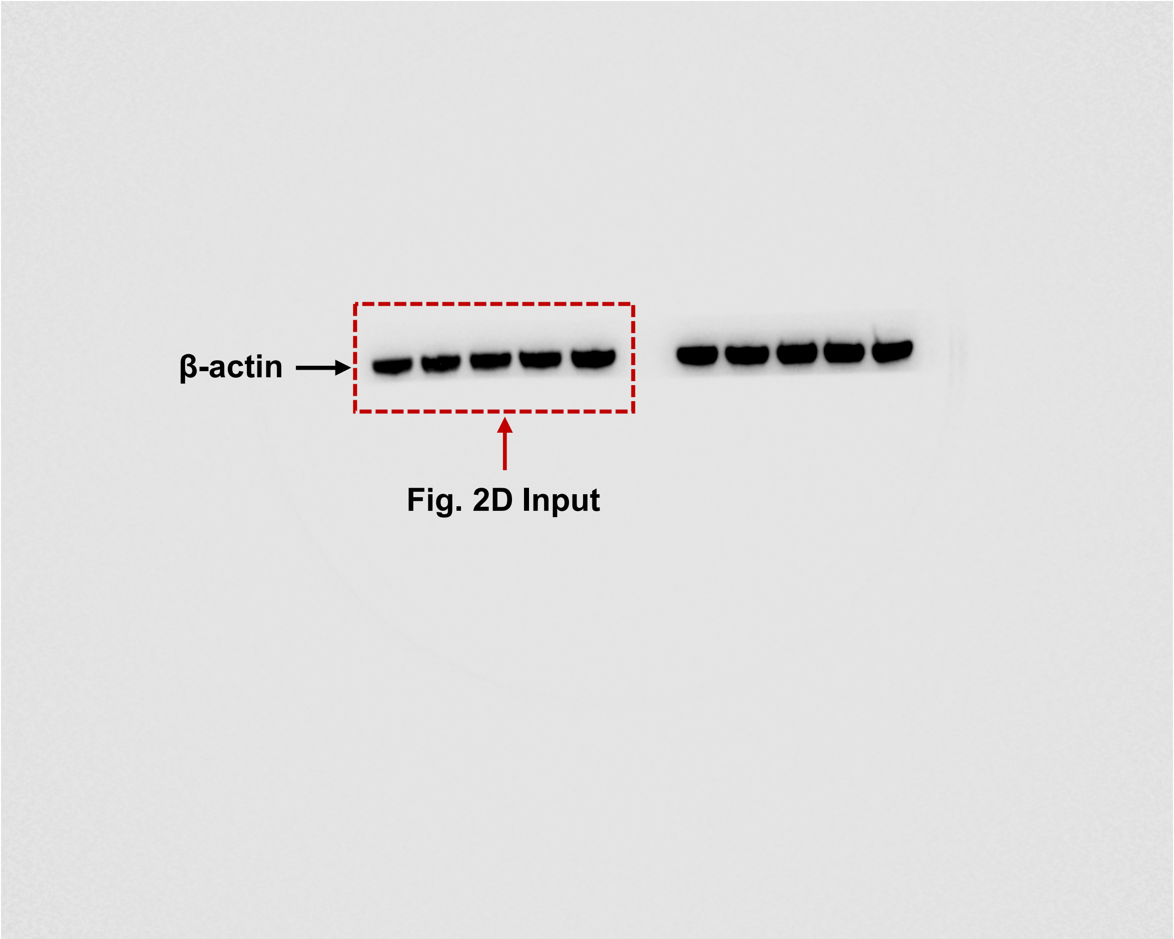
β-actin
Fig. 2D Input

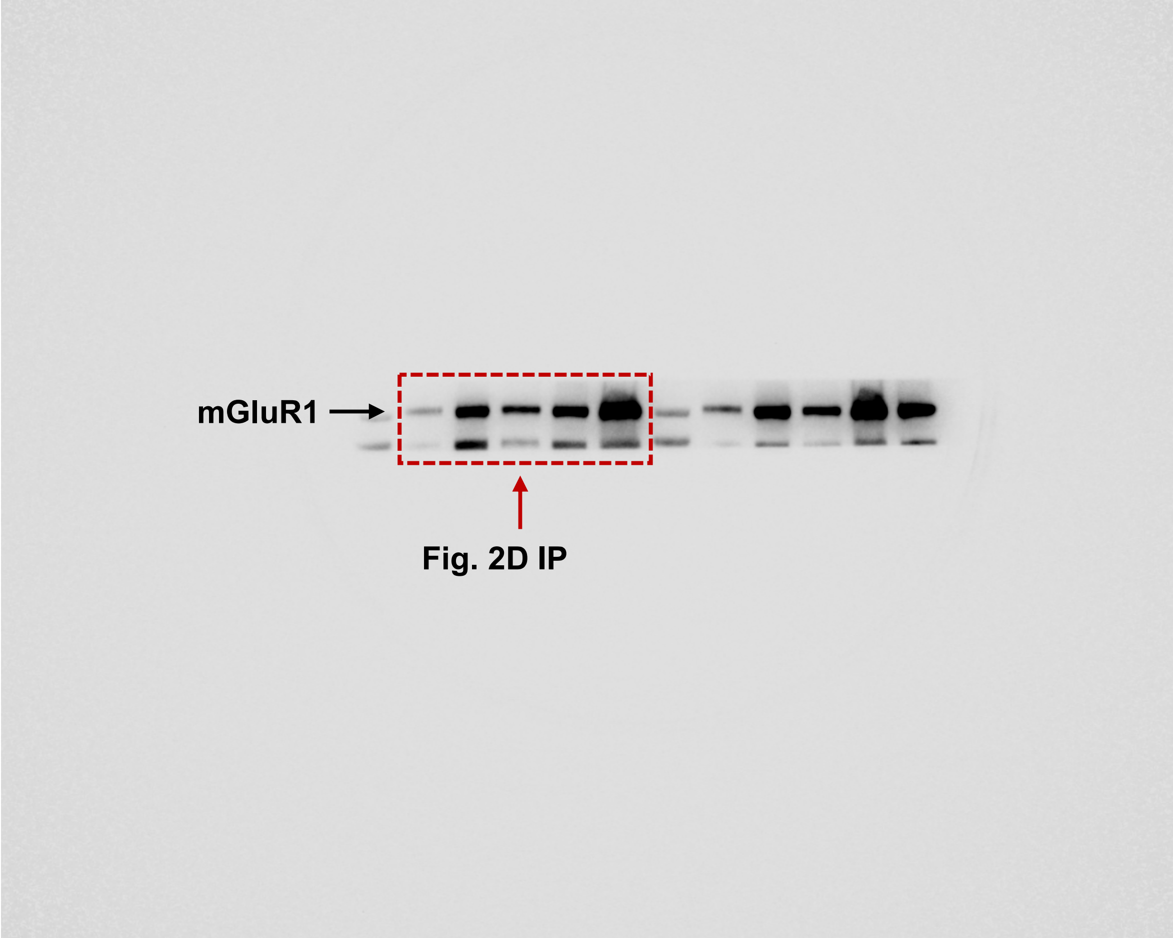
mGluR1
Fig. 2D IP

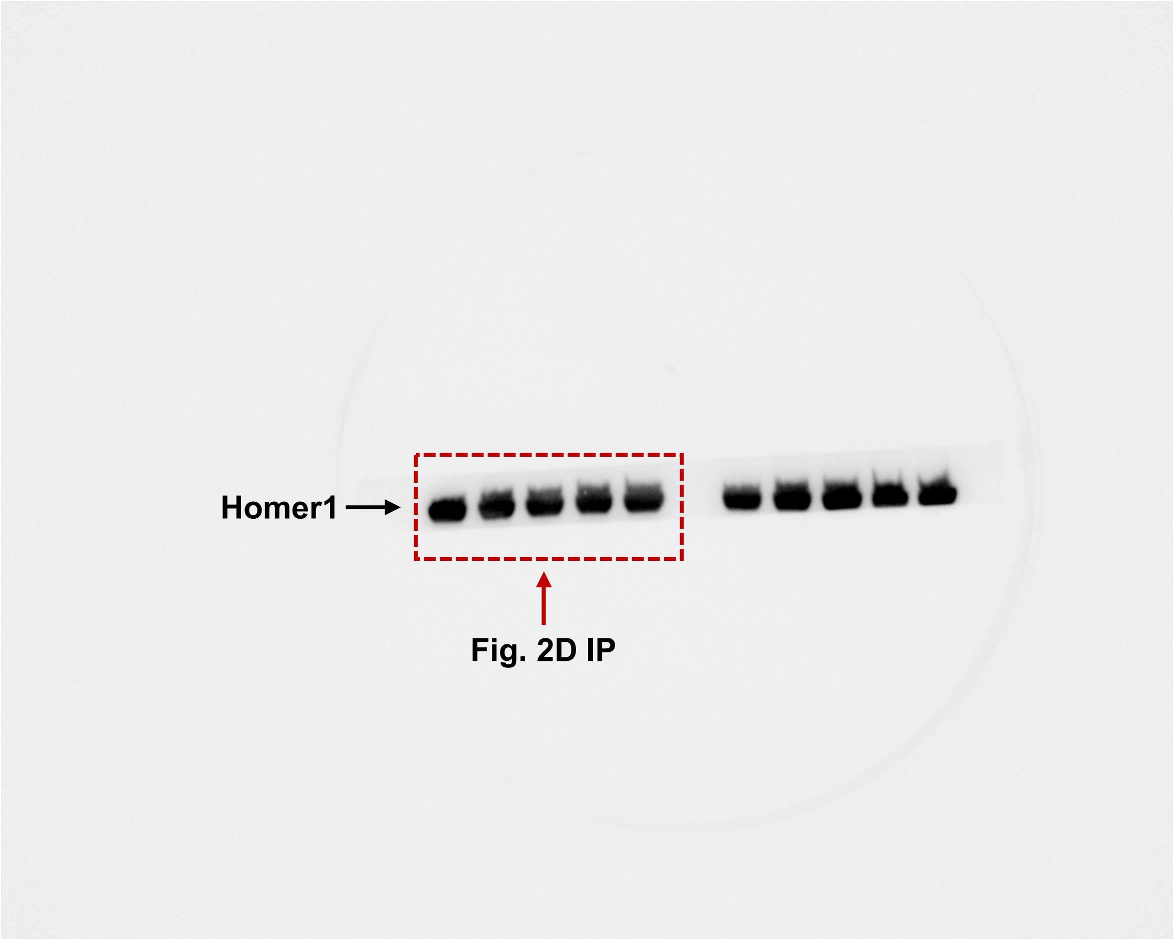
Homer1
Fig. 2D IP

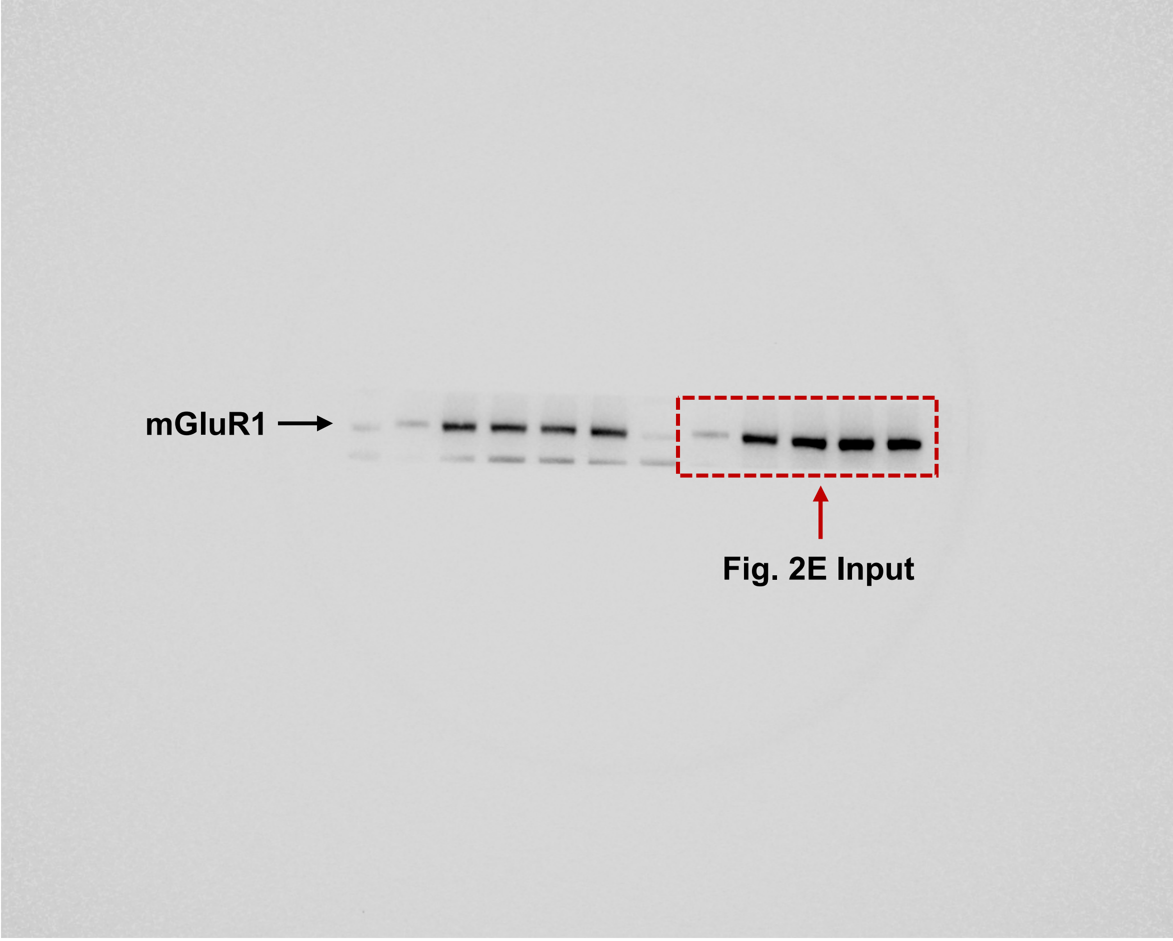
mGluR1
Fig. 2E Input

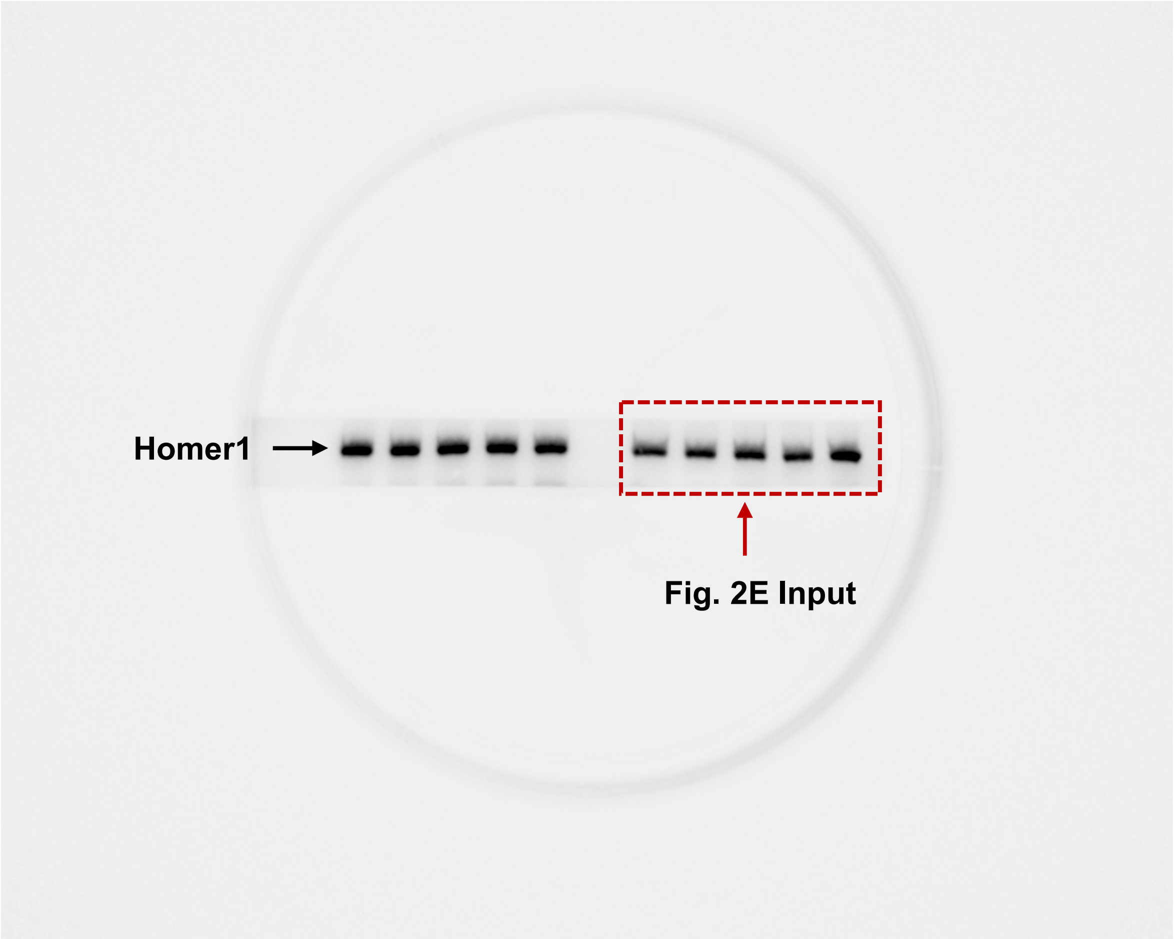
Homer1
Fig. 2E Input

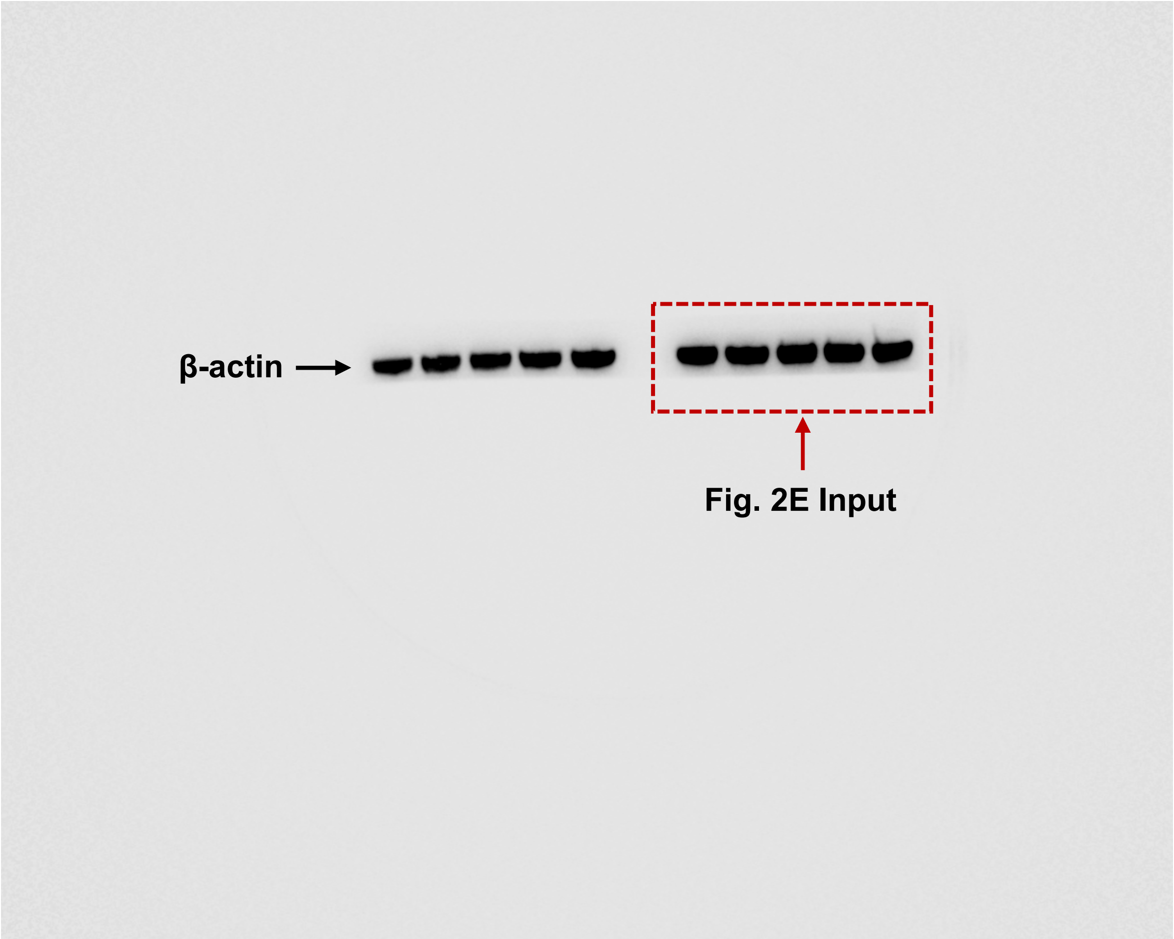

β-actin
Fig. 2E Input

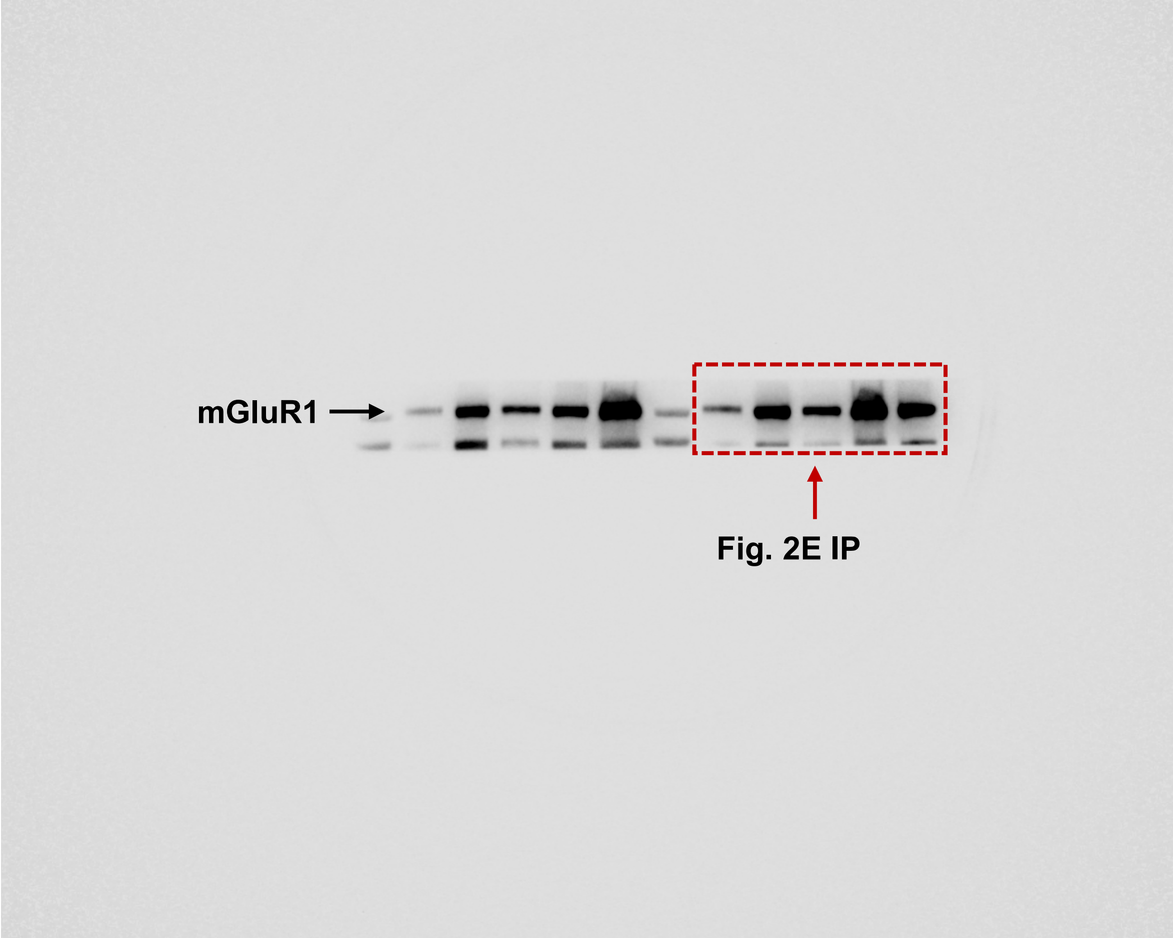

mGluR1
Fig. 2E IP

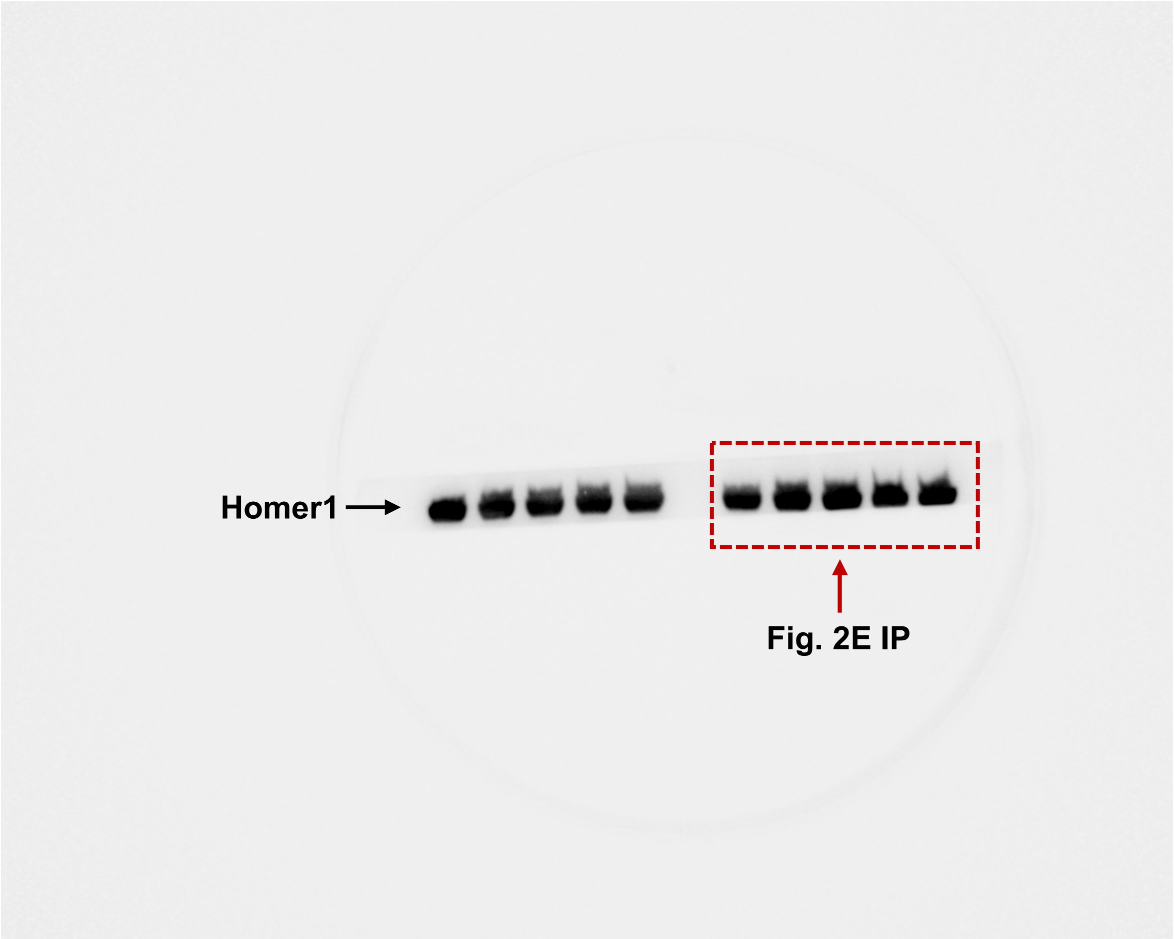
Homer1
Fig. 2E IP

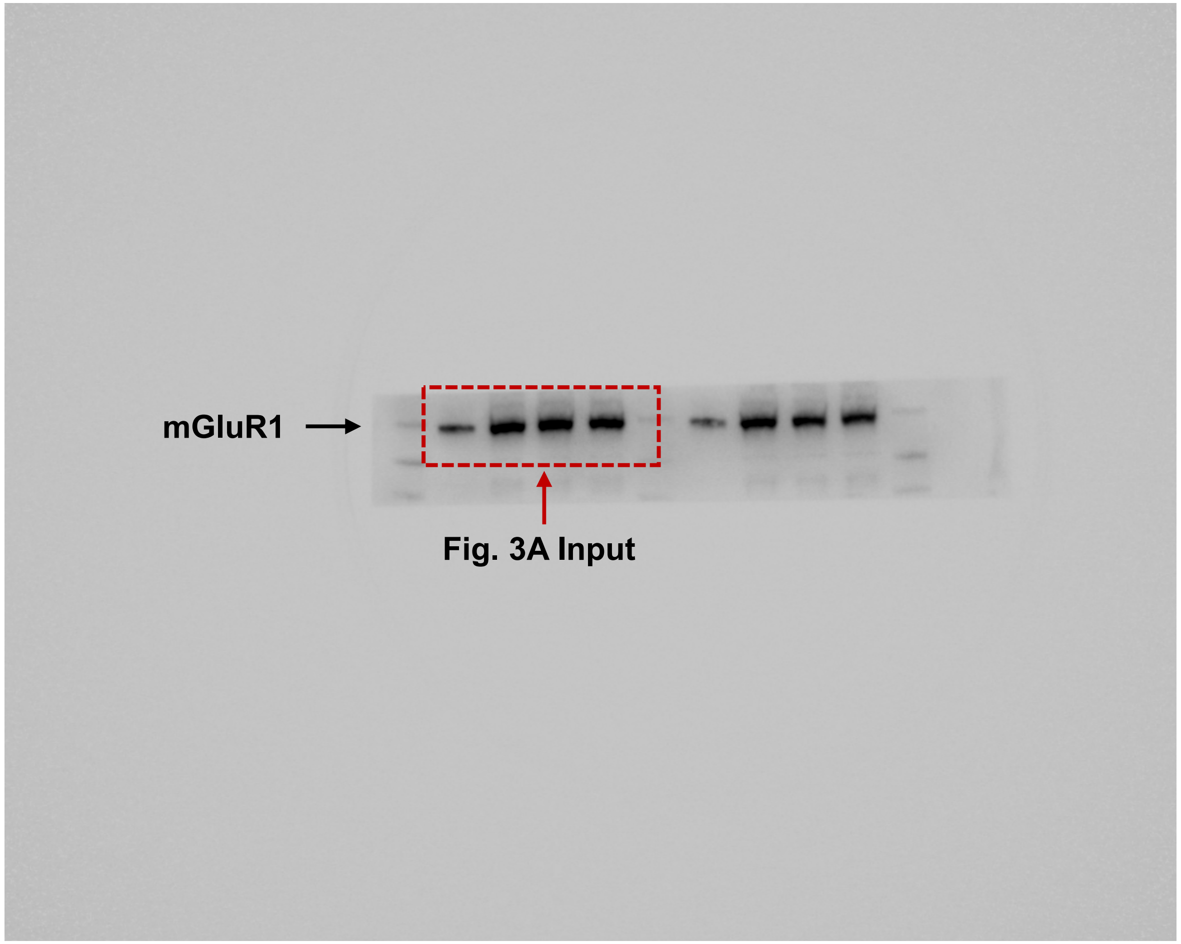
mGluR1
Fig. 3A Input

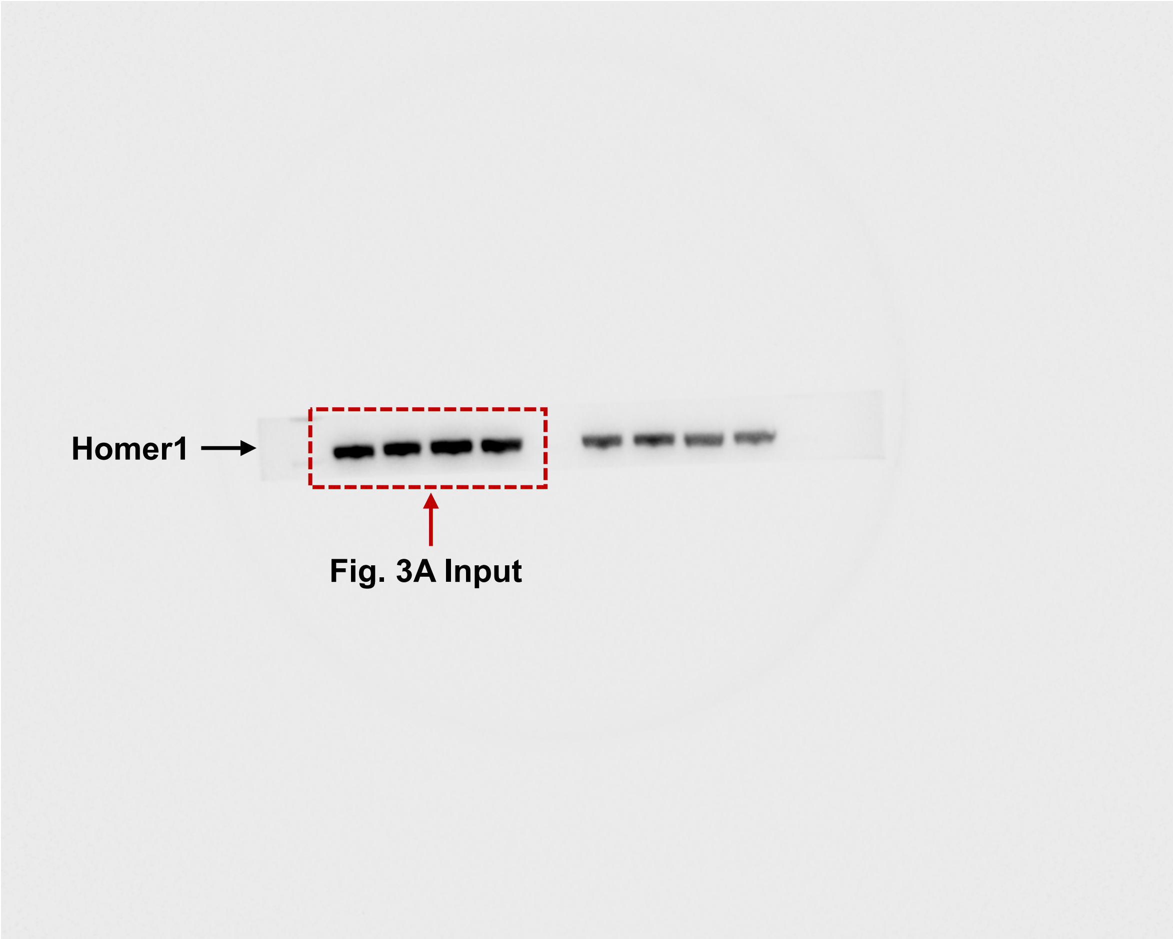
Homer1
Fig. 3A Input

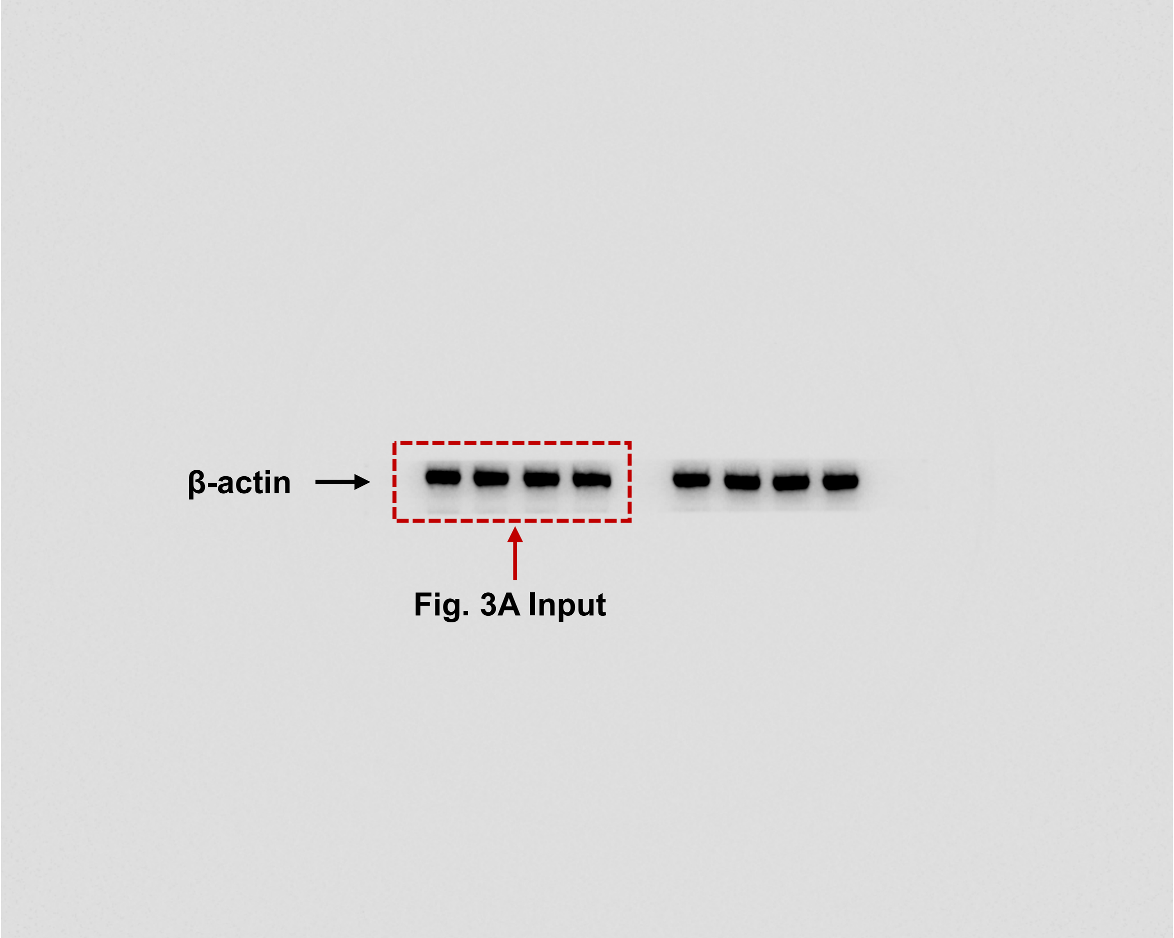
β-actin
Fig. 3A Input

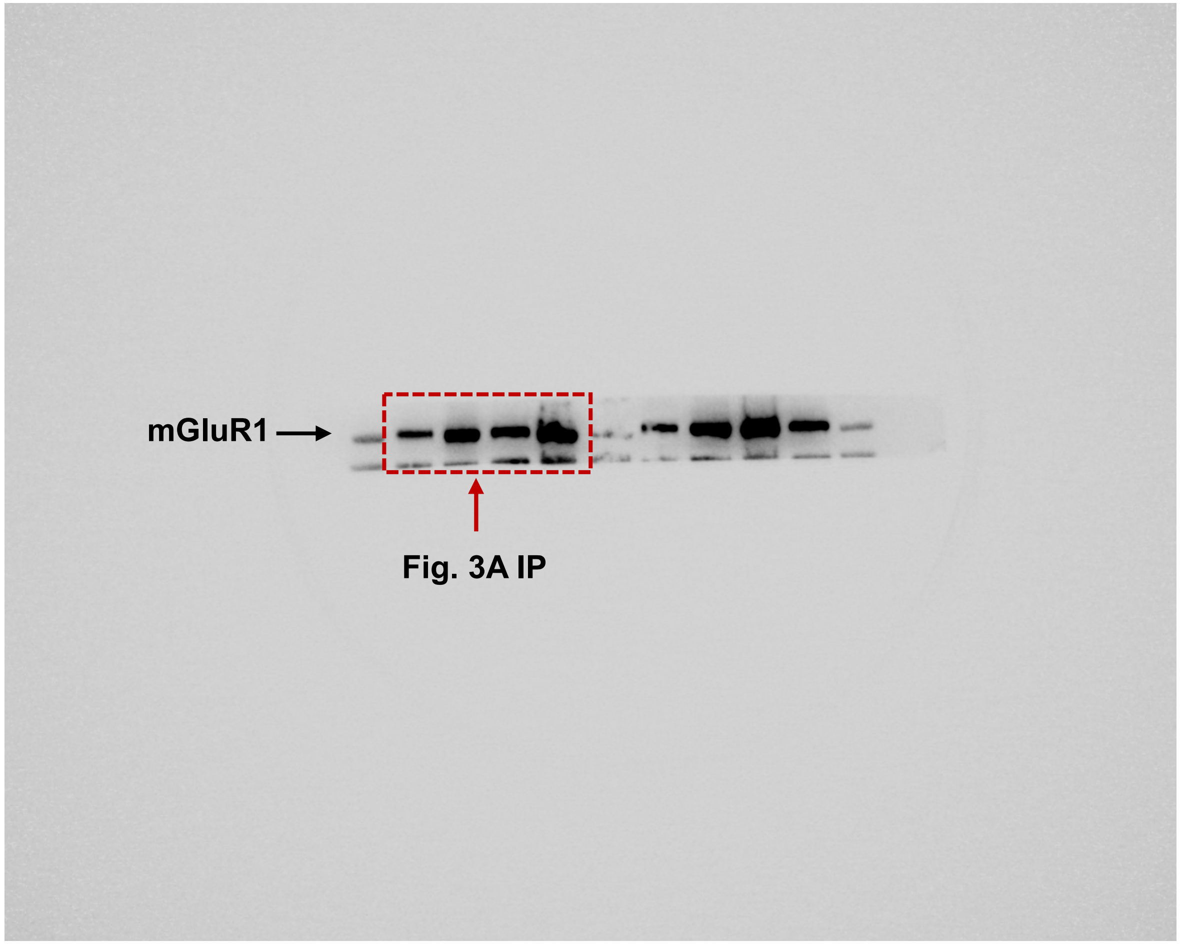
mGluR1
Fig. 3A IP

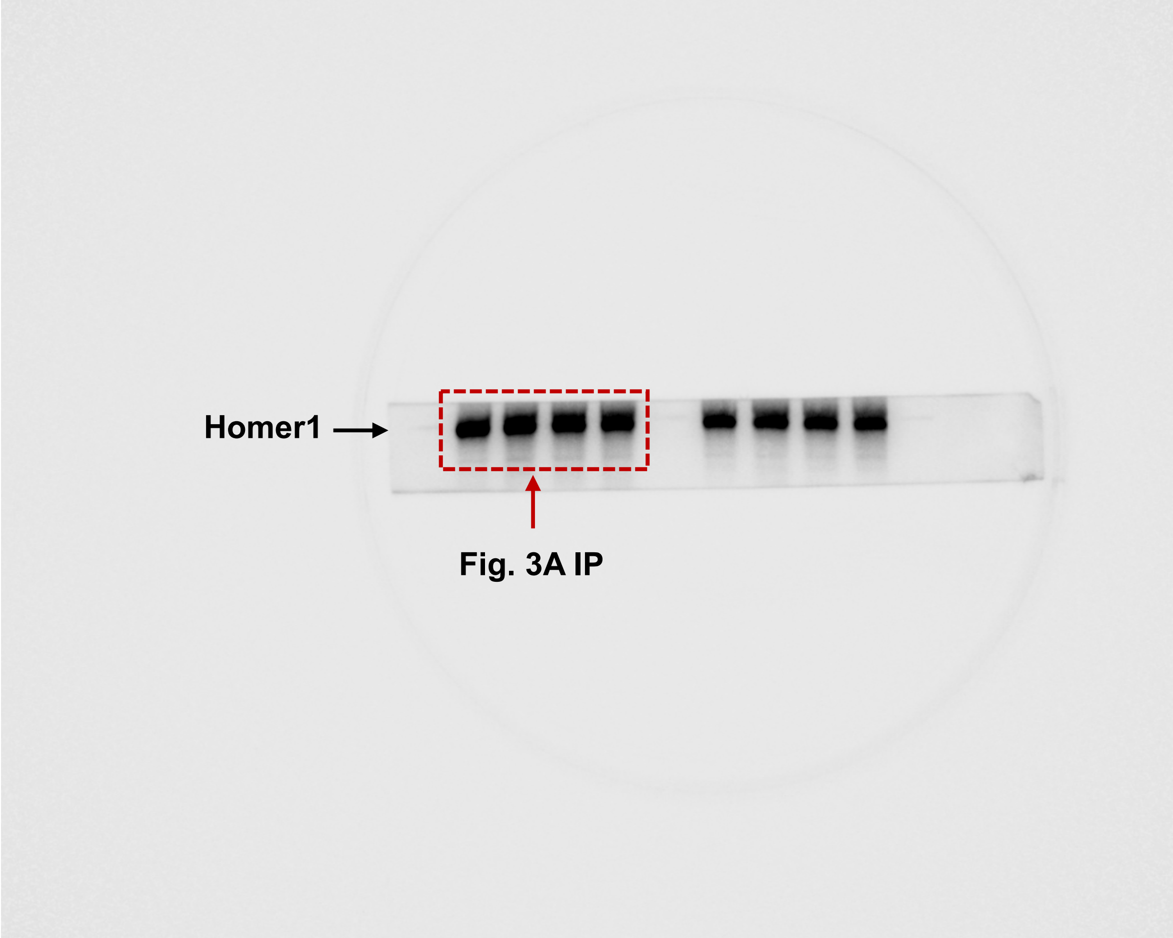
Homer1
Fig. 3A IP

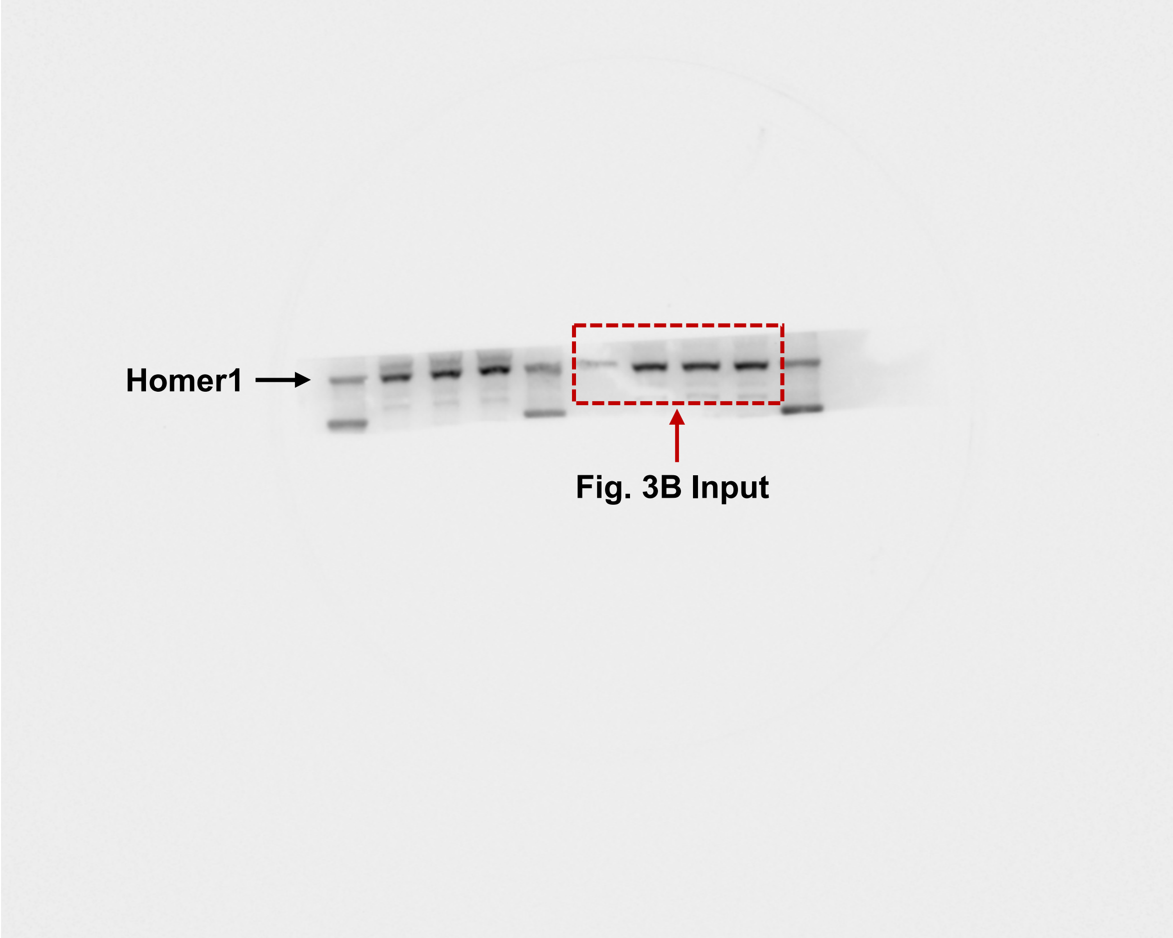

Homer1
Fig. 3B Input

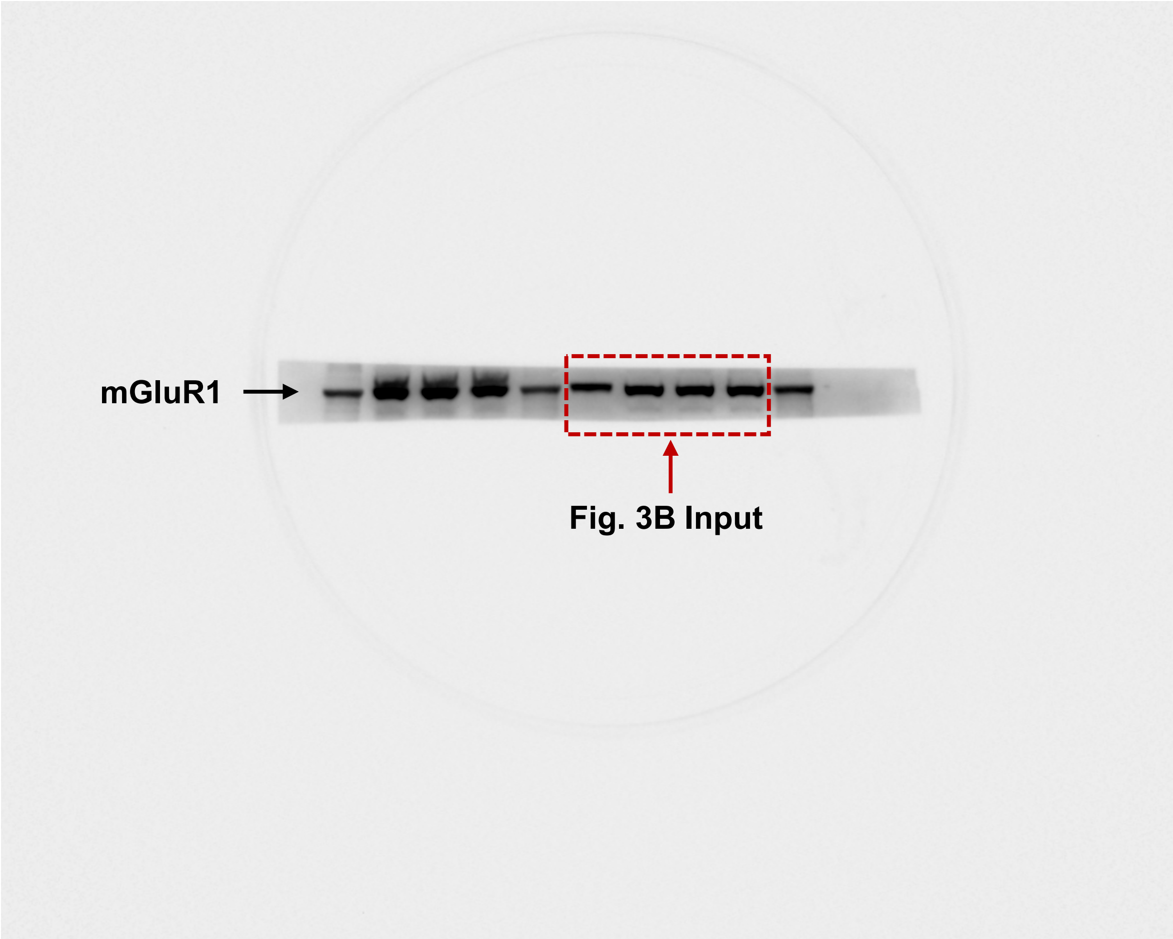
mGluR1
Fig. 3B Input

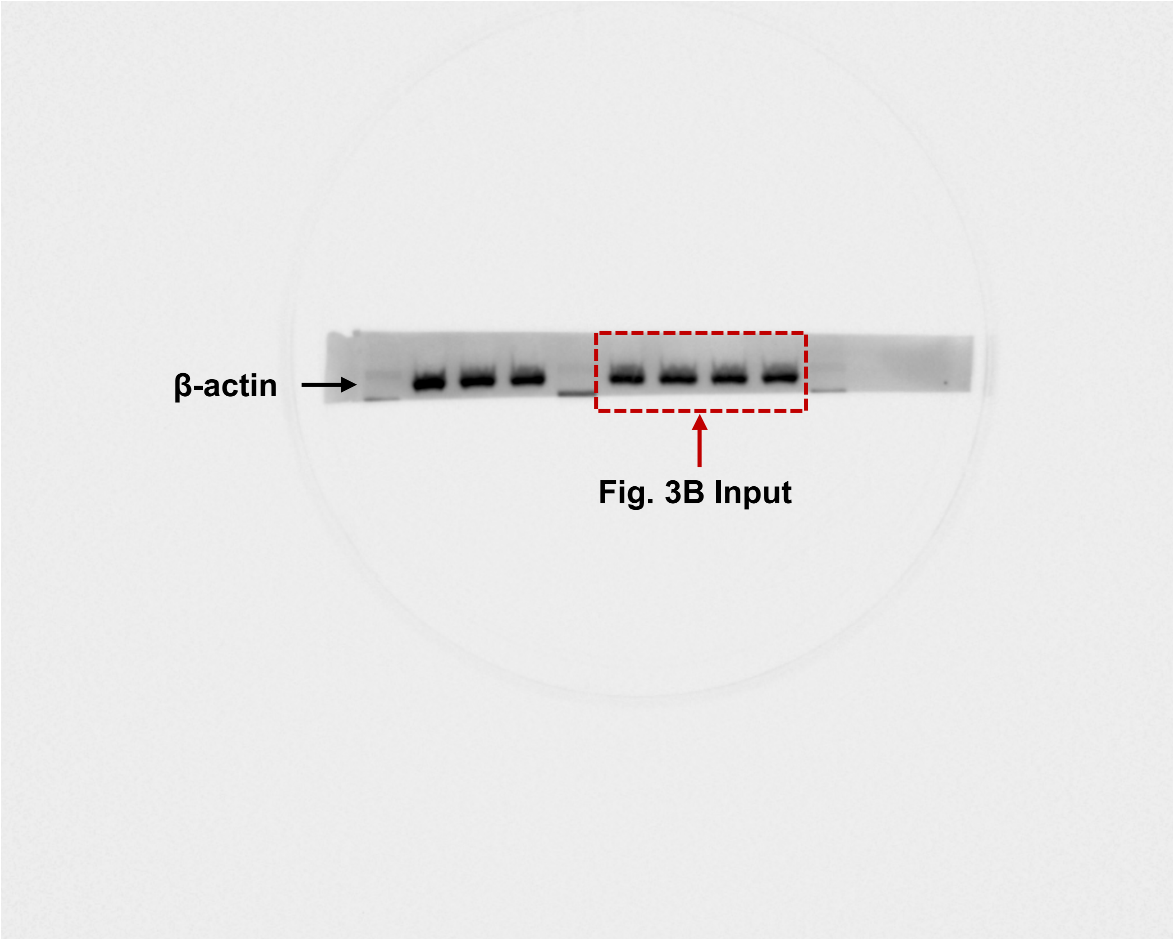
β-actin
Fig. 3B Input

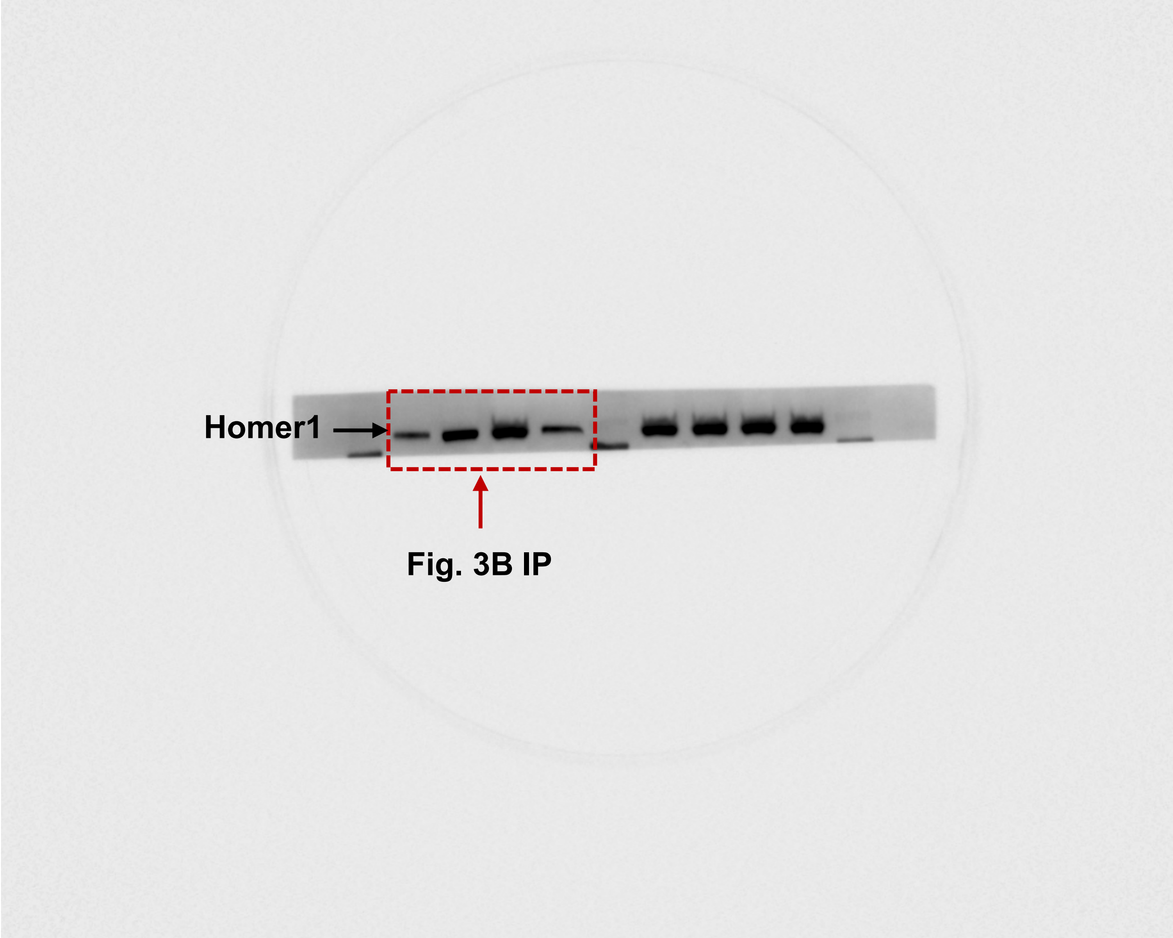
Homer1
Fig. 3B IP

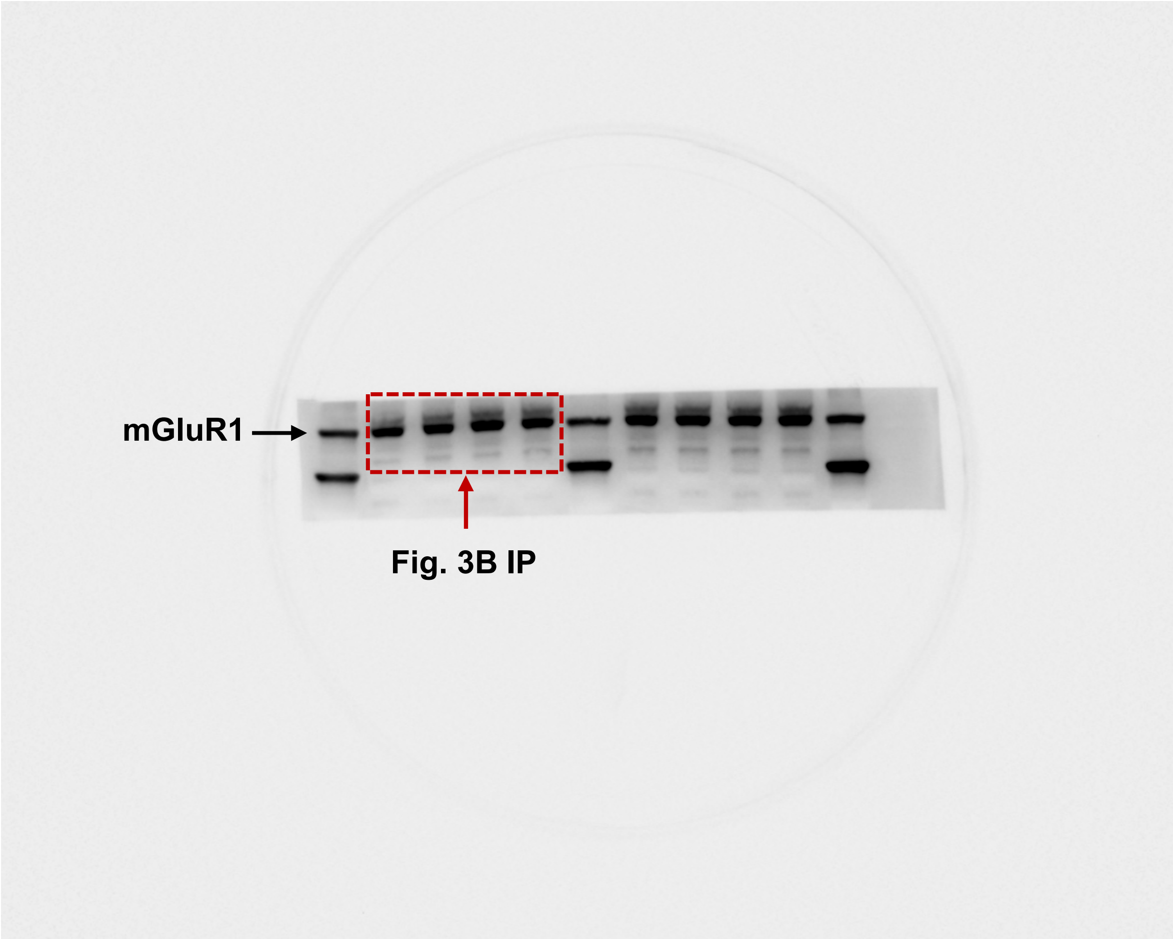
mGluR1
Fig. 3B IP

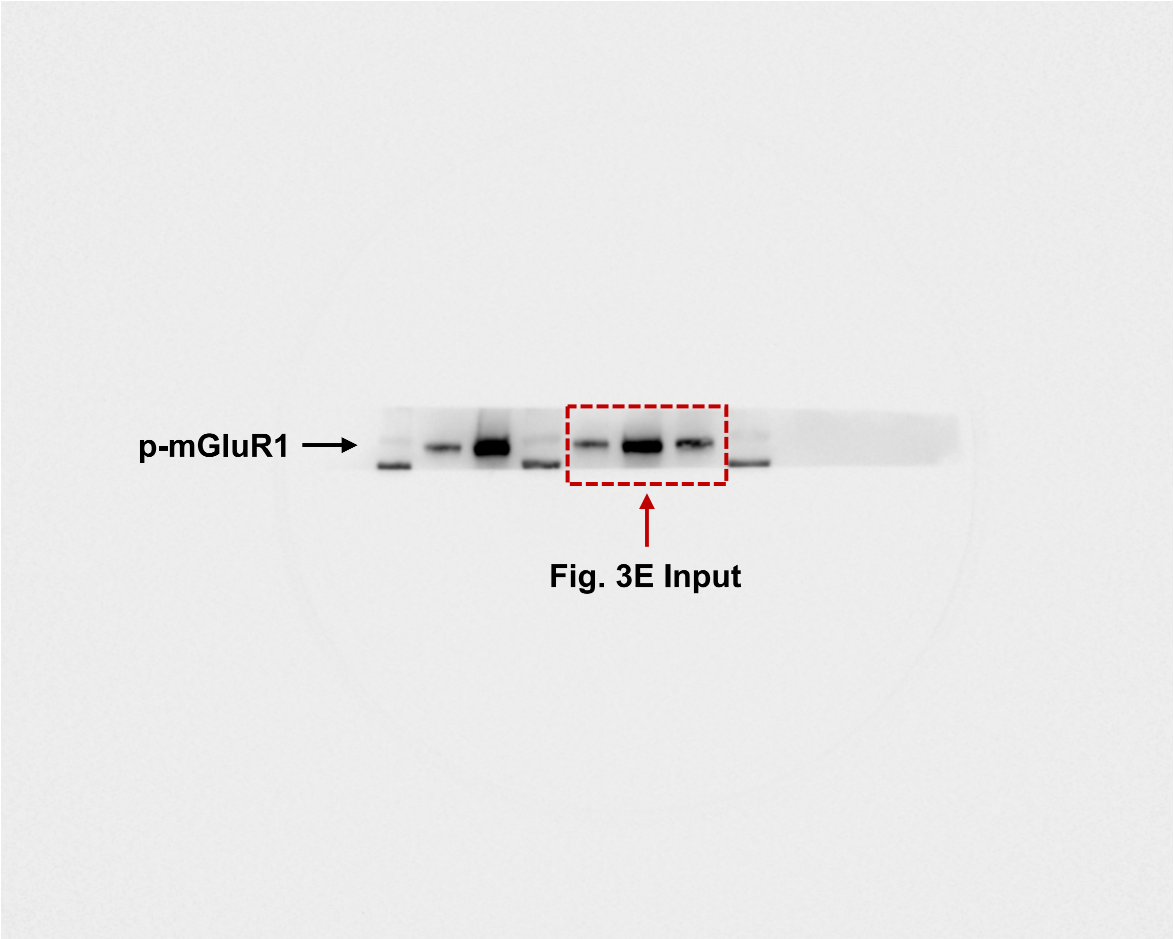
p-mGluR1
Fig. 3E Input

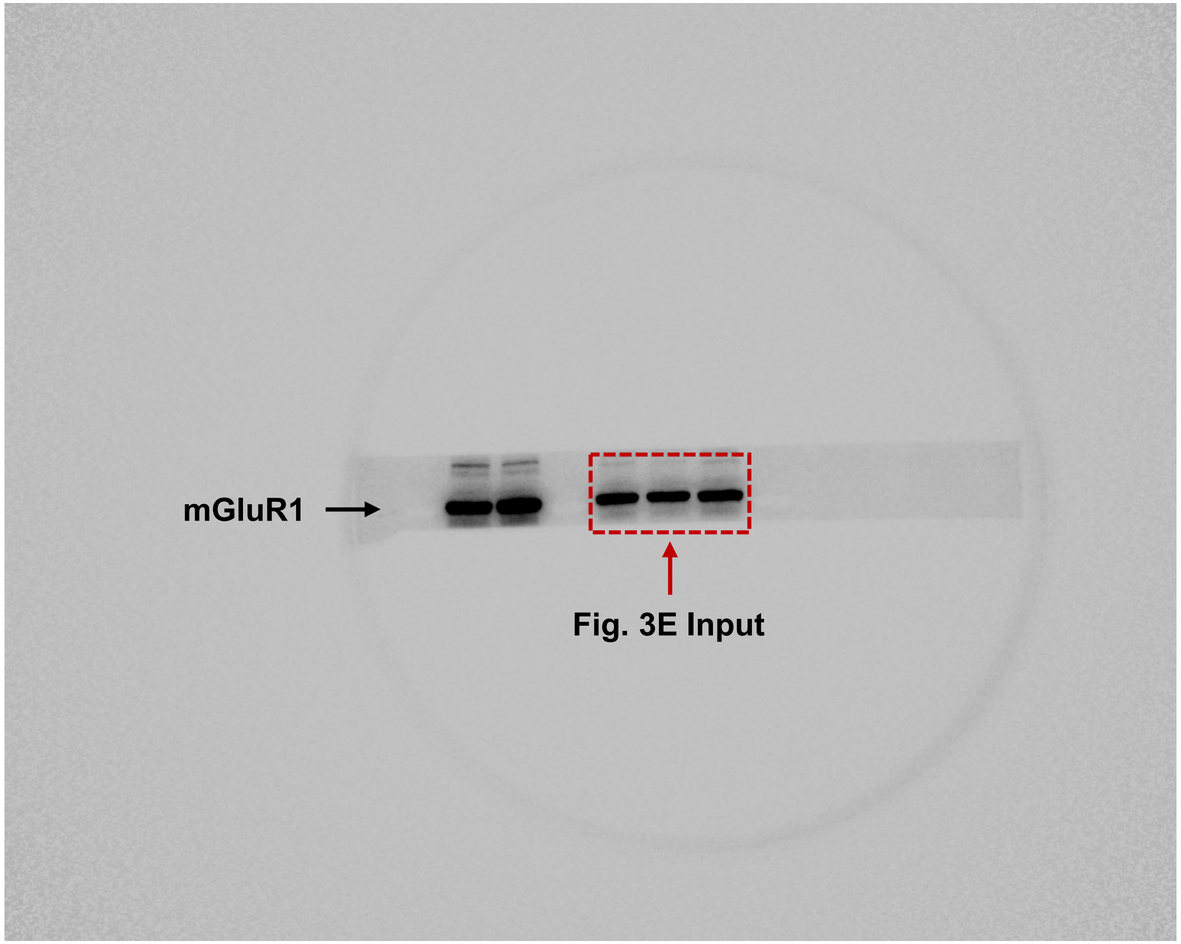
mGluR1
Fig. 3E Input

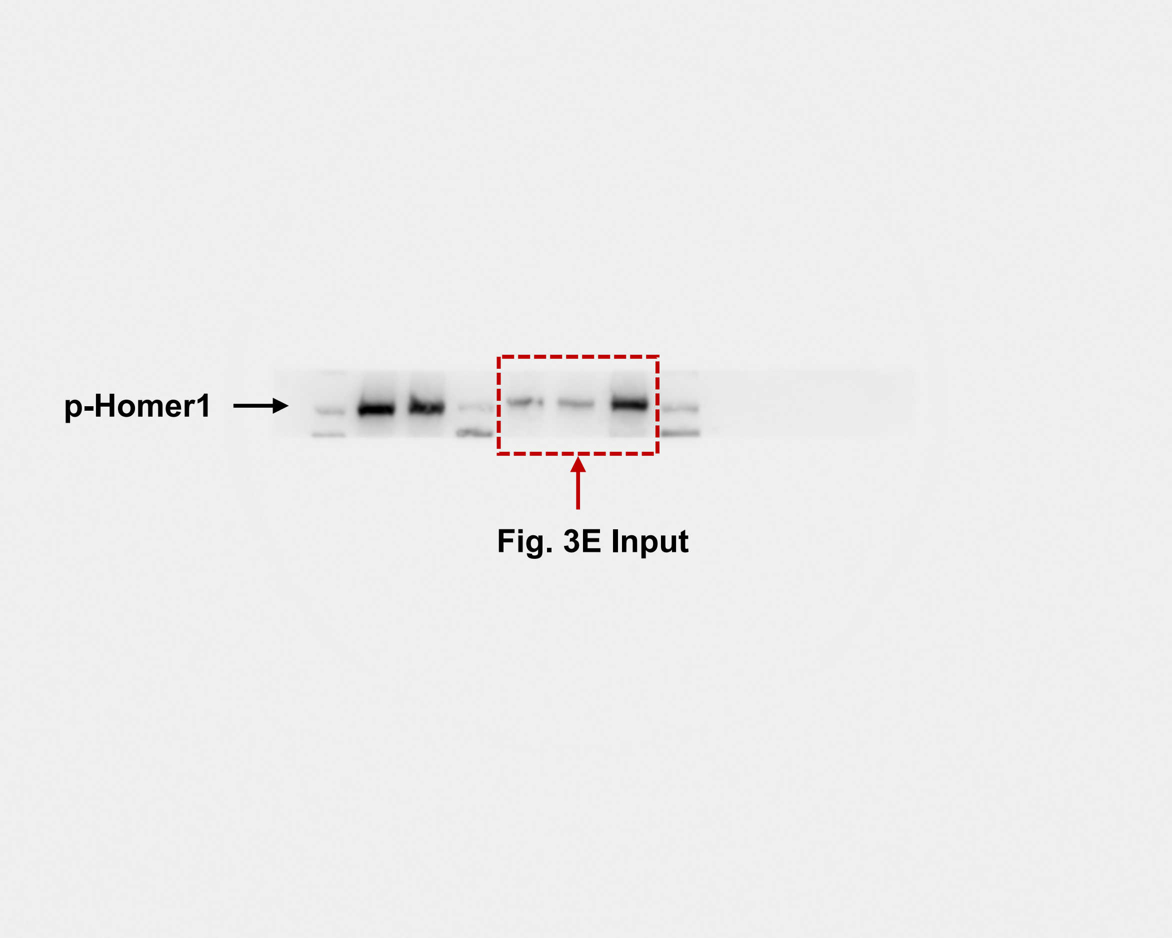
p-Homer1
Fig. 3E Input

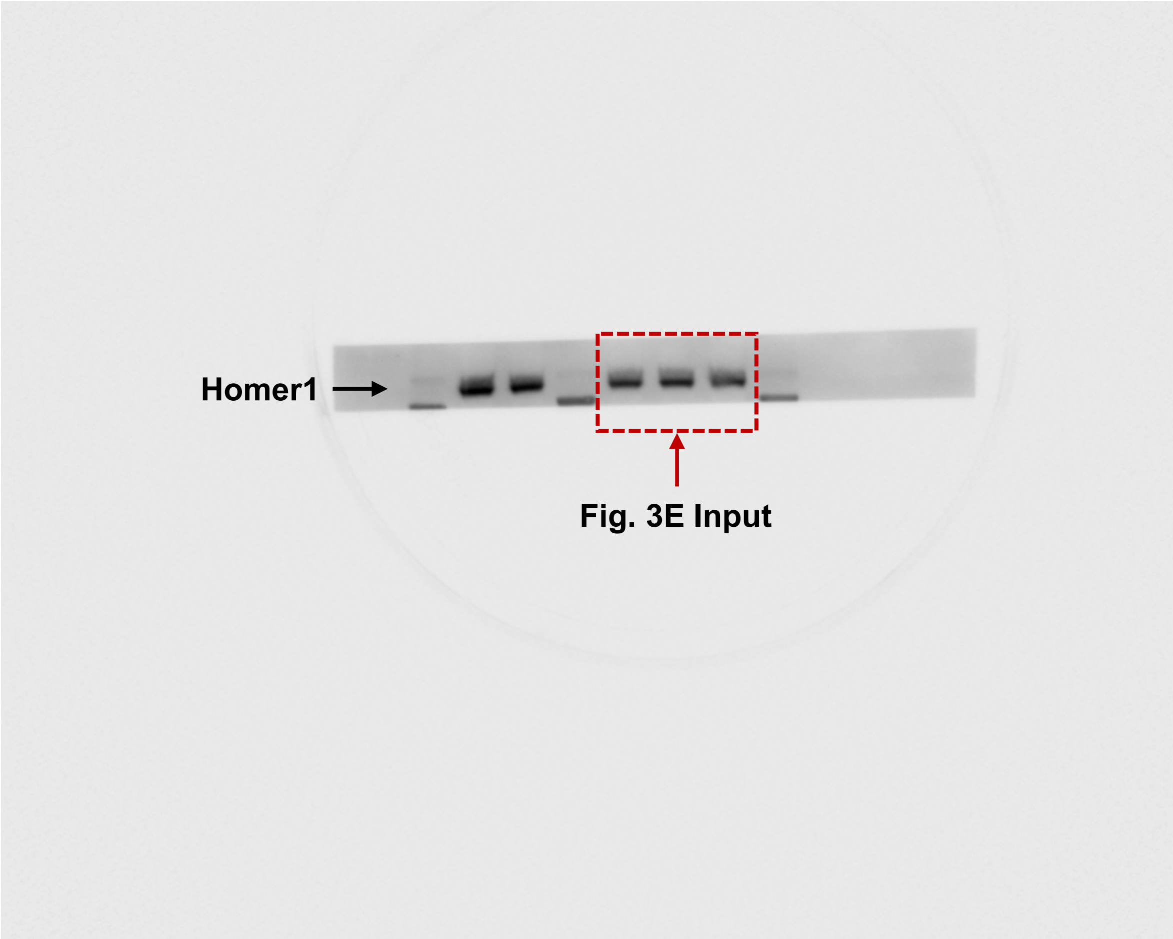
Homer1
Fig. 3E Input

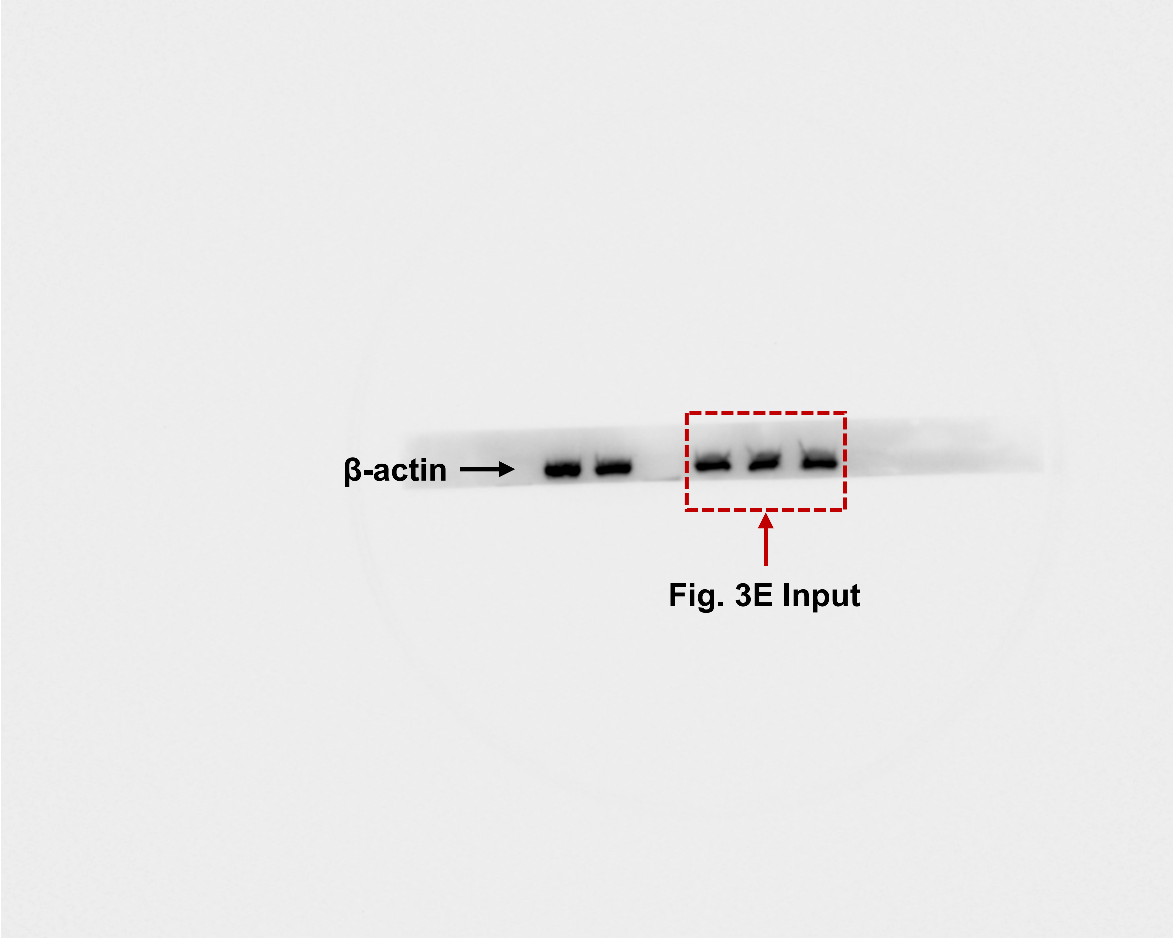

β-actin
Fig. 3E Input

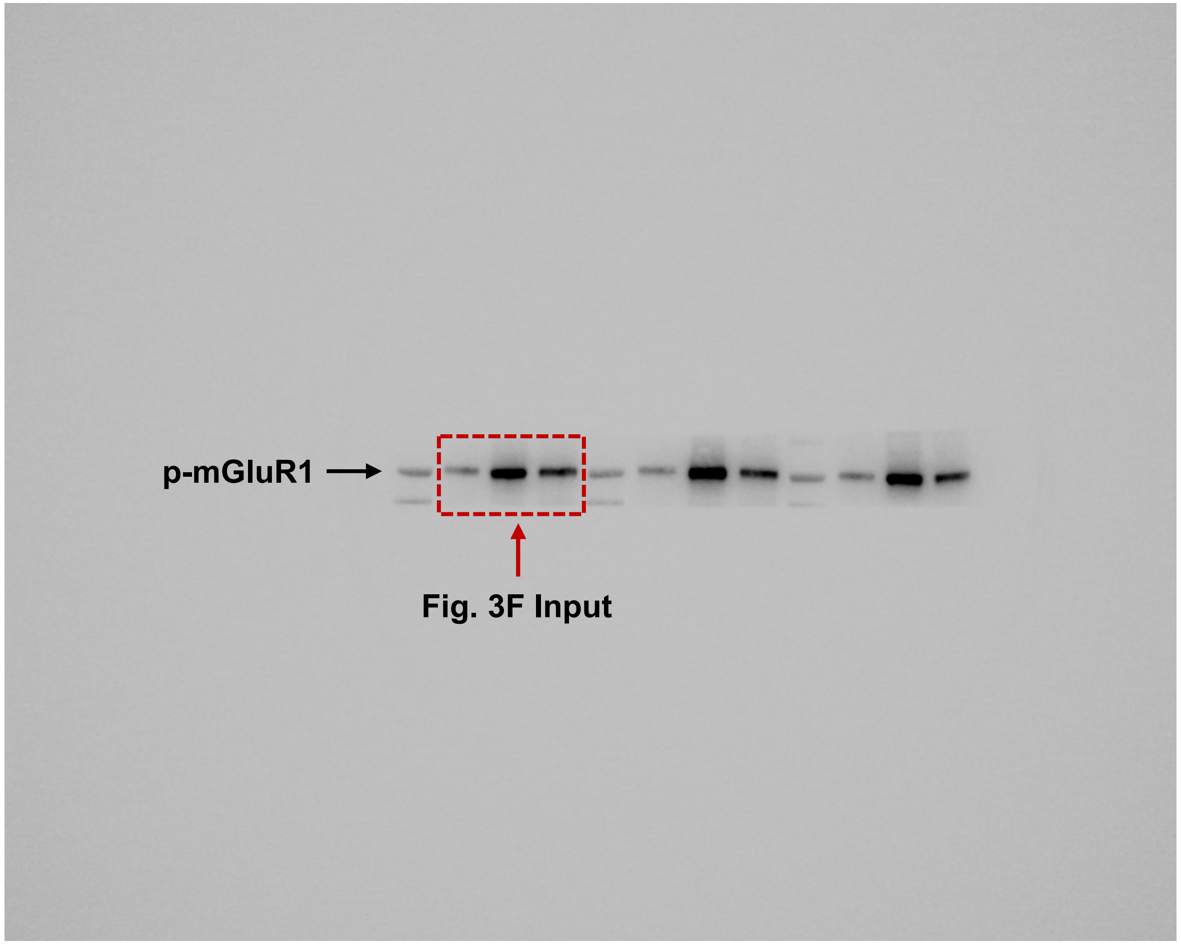
p-mGluR1
Fig. 3F Input

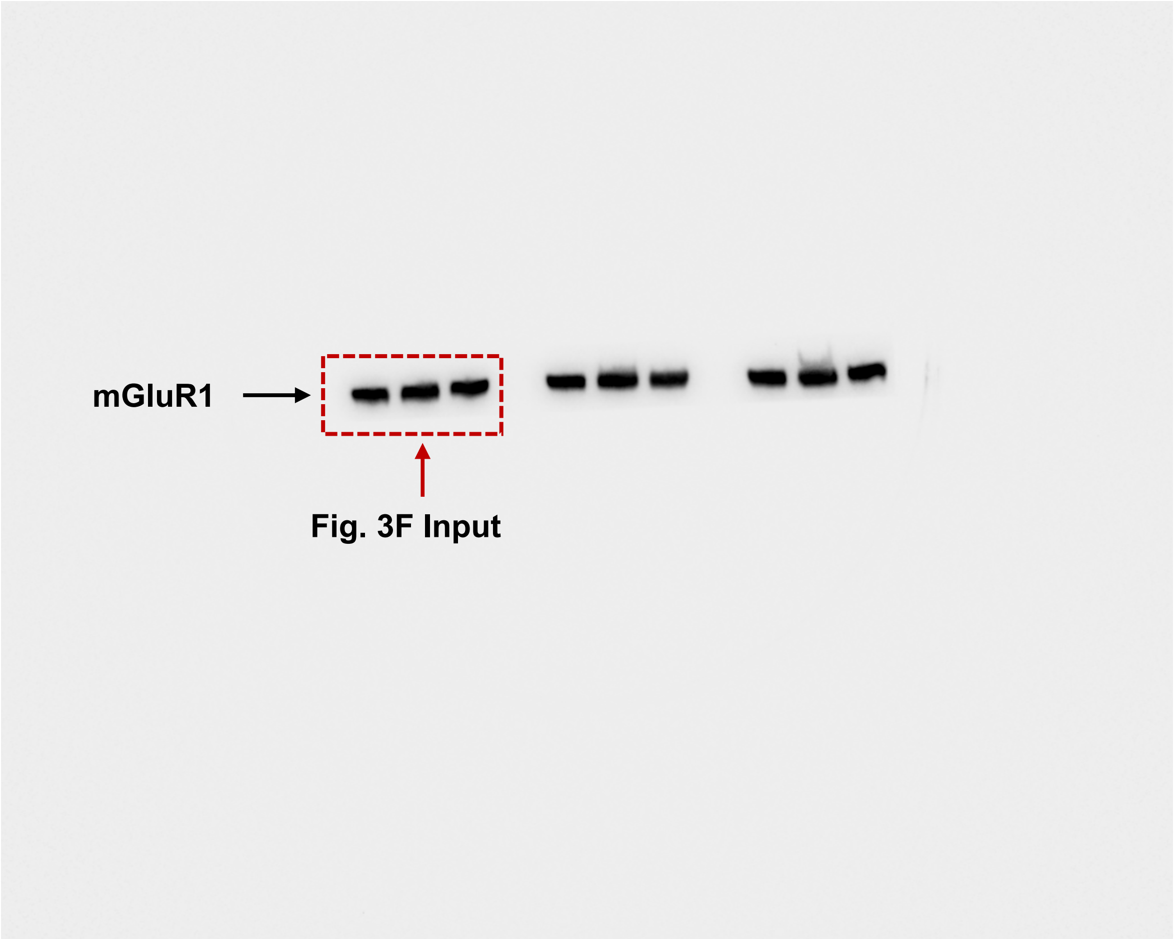
mGluR1
Fig. 3F Input

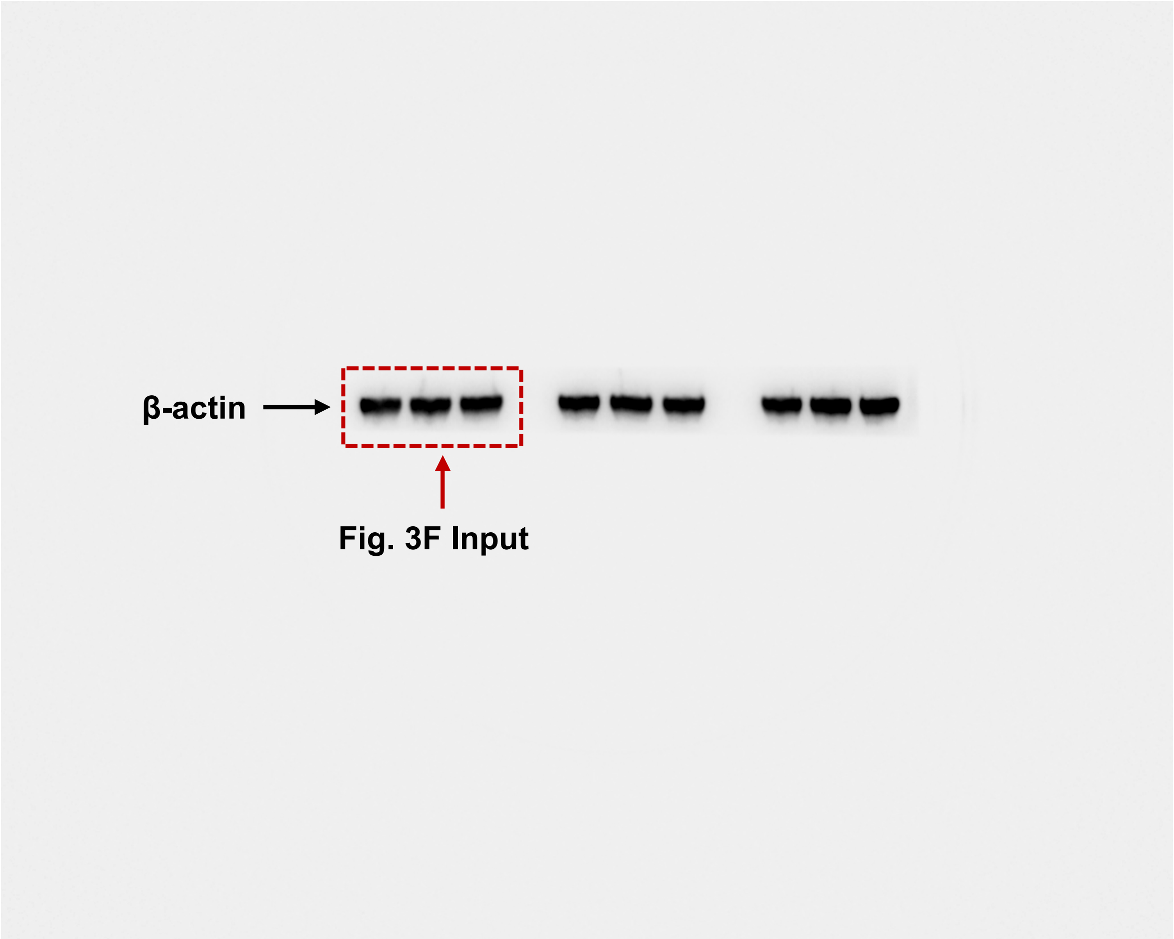
β-actin
Fig. 3F Input

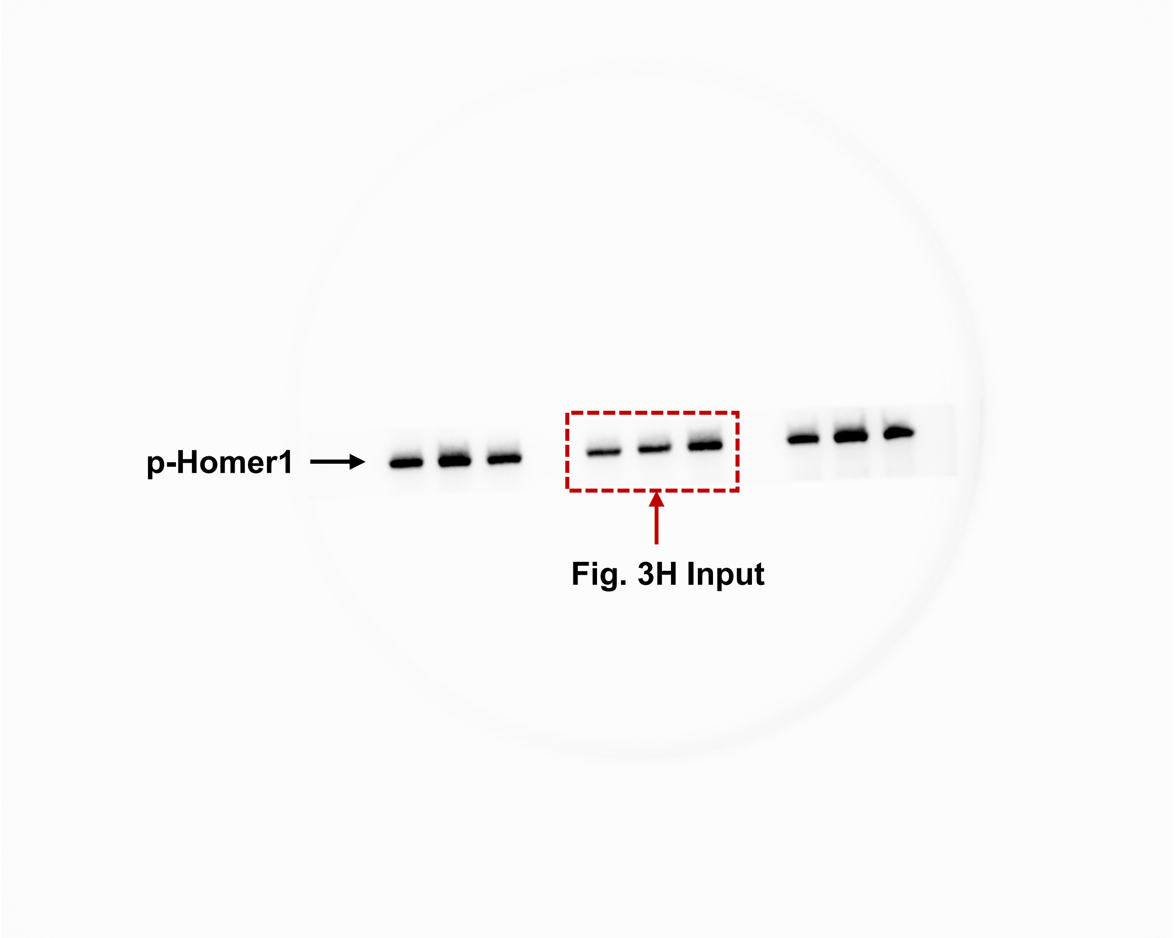
p-Homer1
Fig. 3H Input

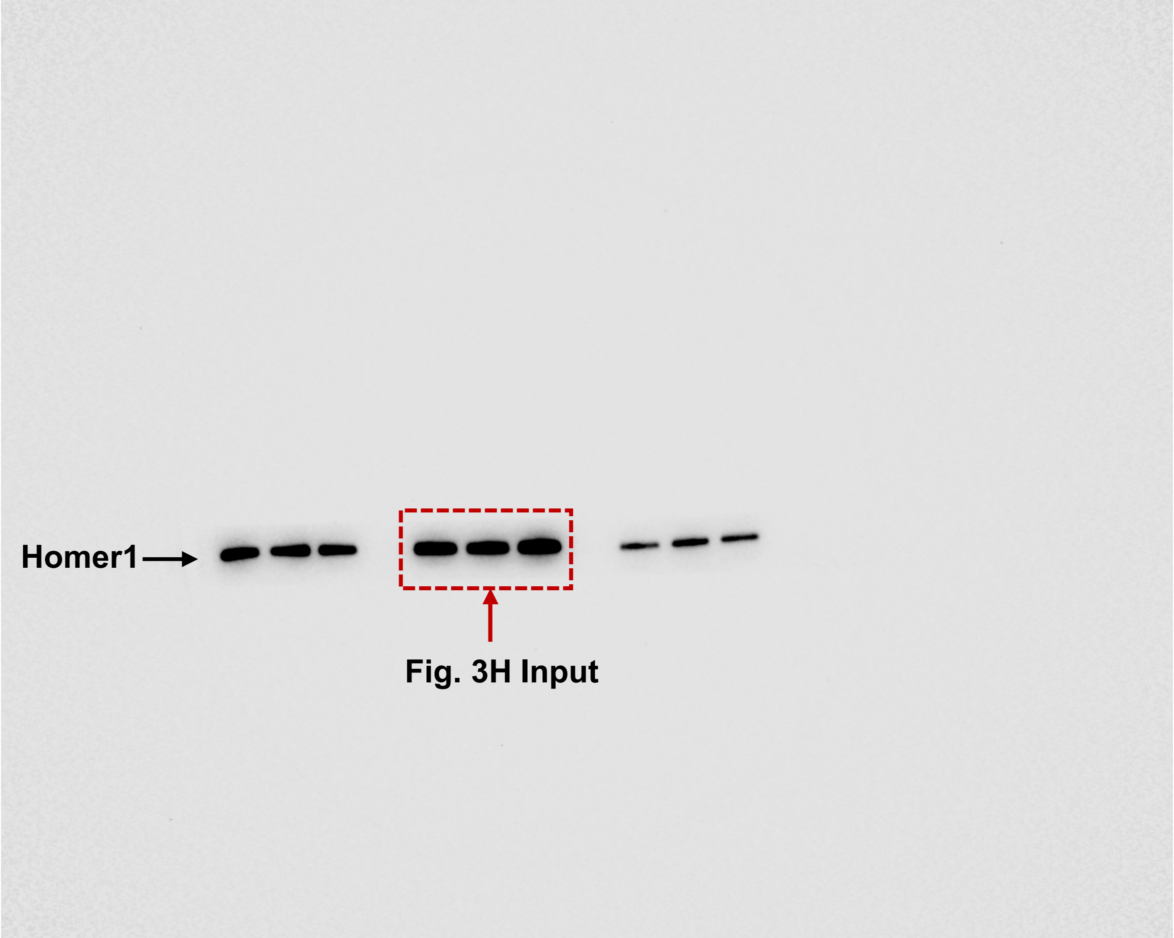

Homer1
Fig. 3H Input

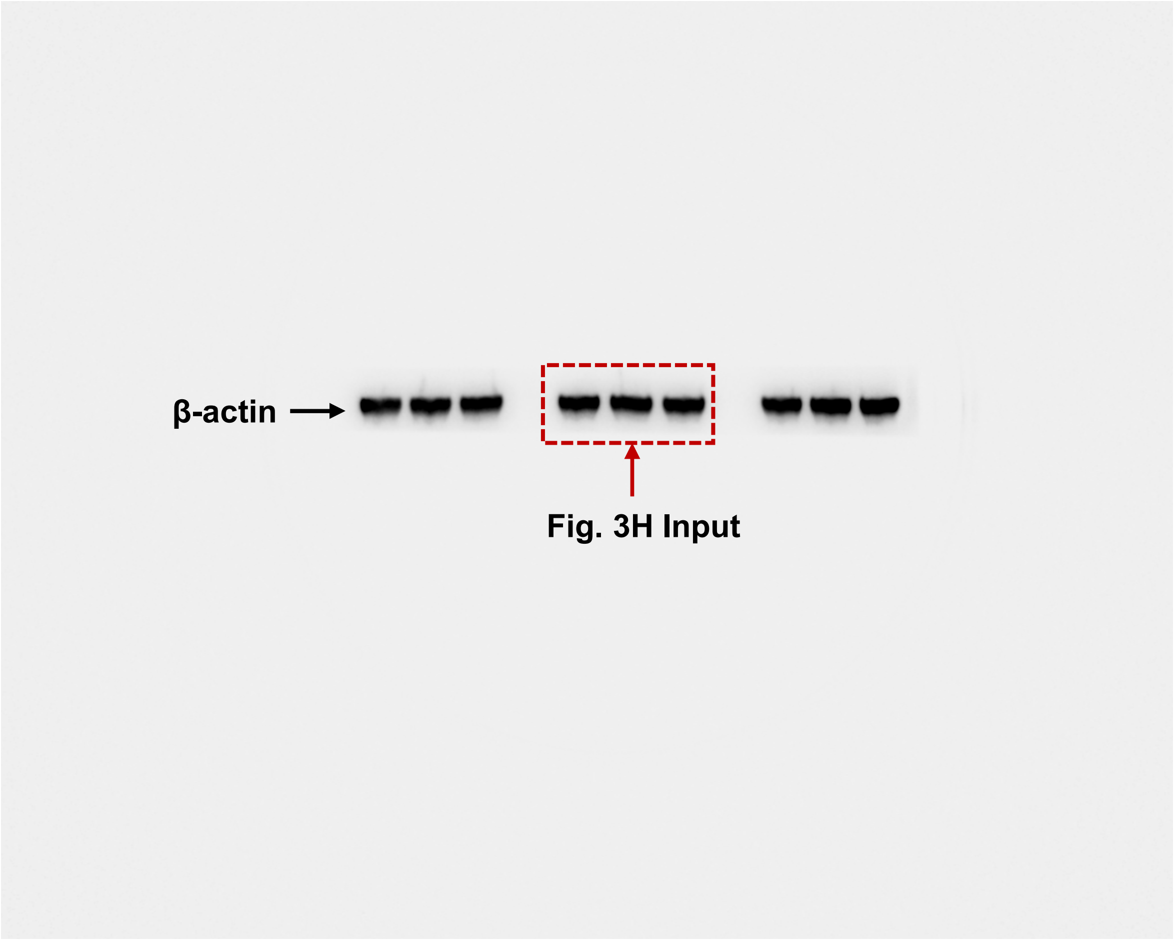
β-actin
Fig. 3H Input

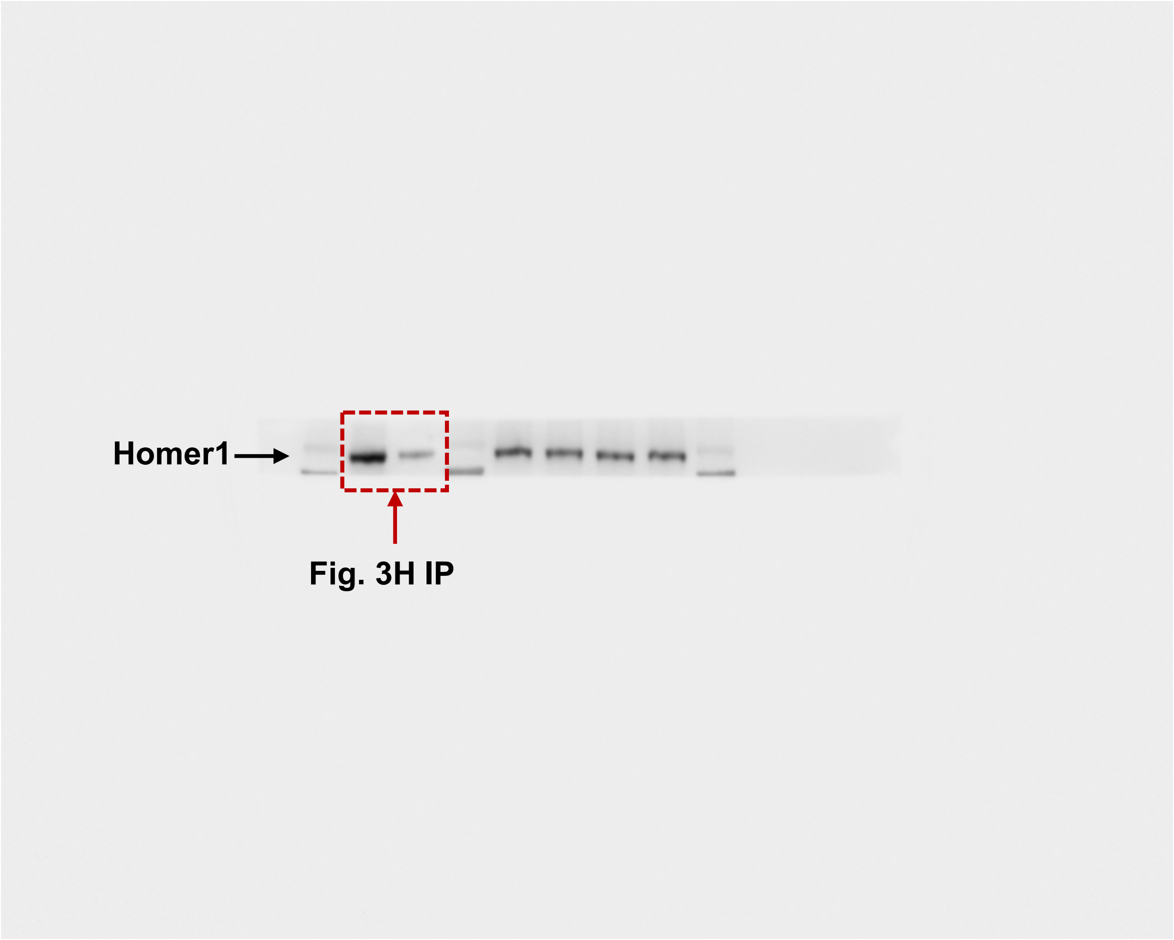

Homer1
Fig. 3H IP

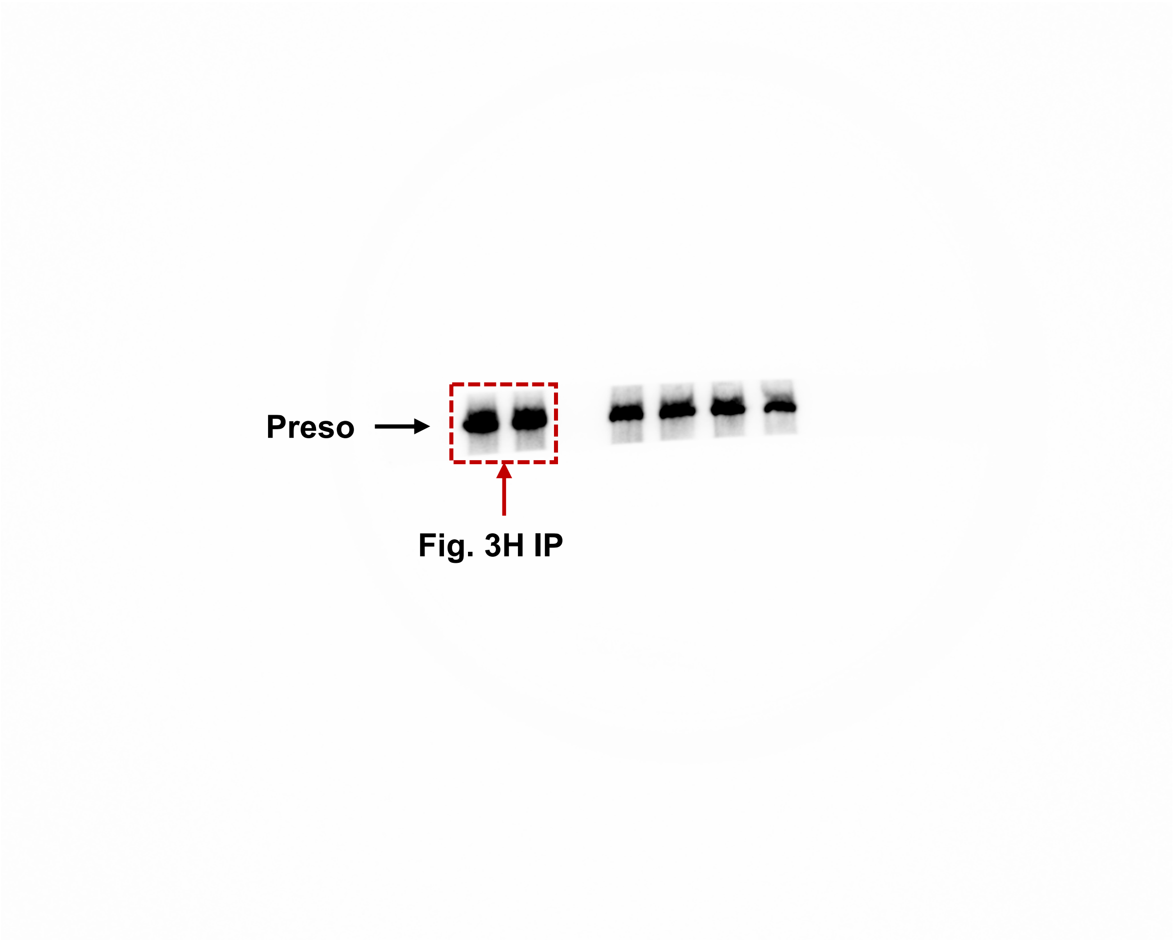
Preso
Fig. 3H IP

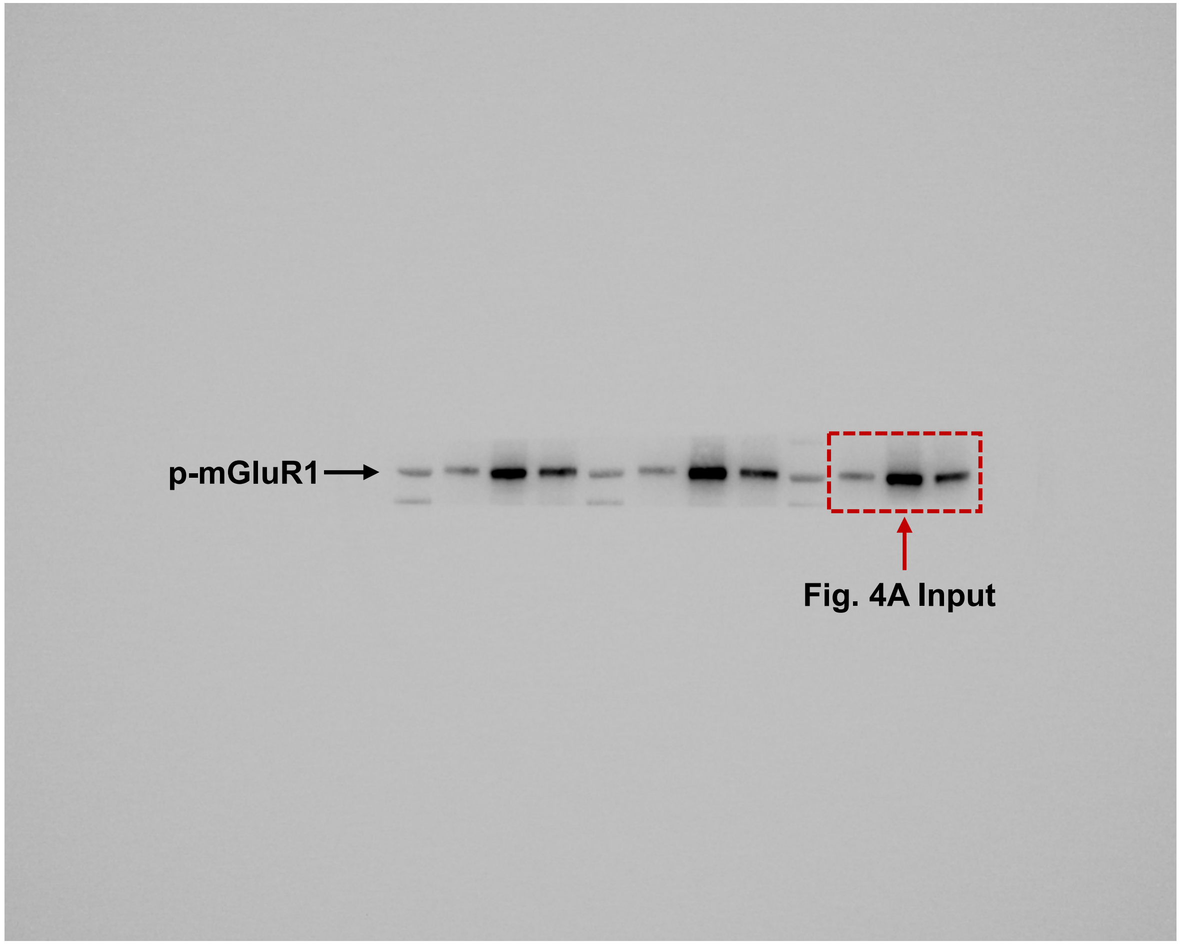
p-mGluR1
Fig. 4A Input

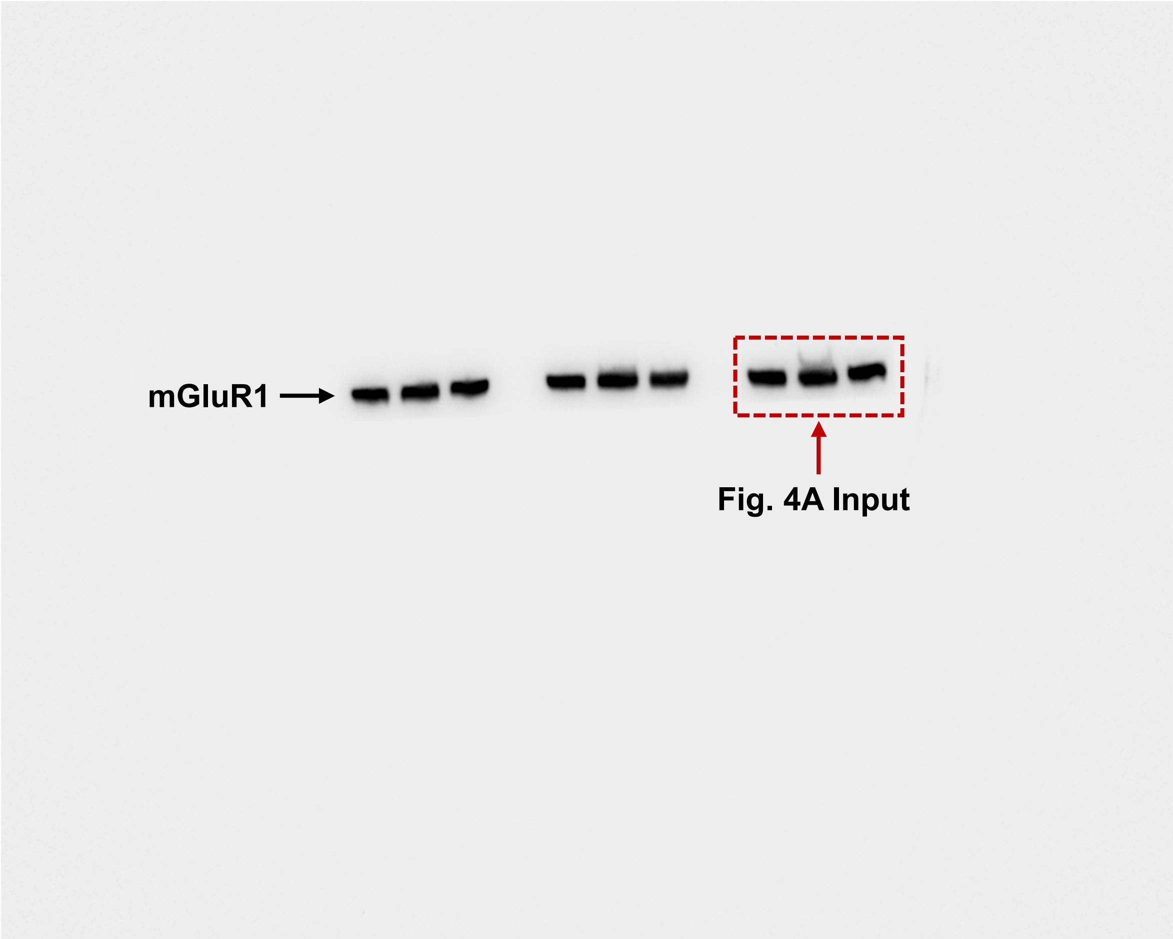
mGluR1
Fig. 4A Input

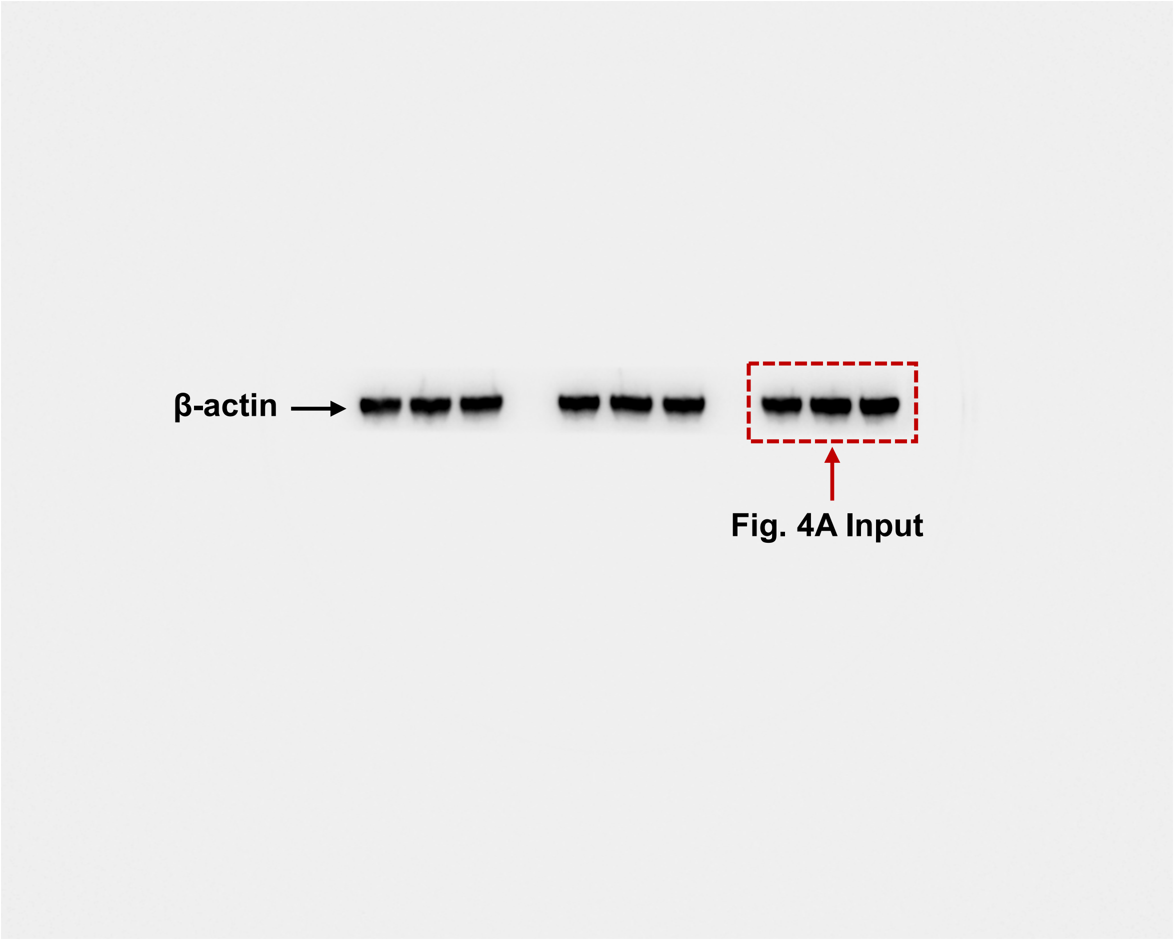
β-actin
Fig. 4A Input

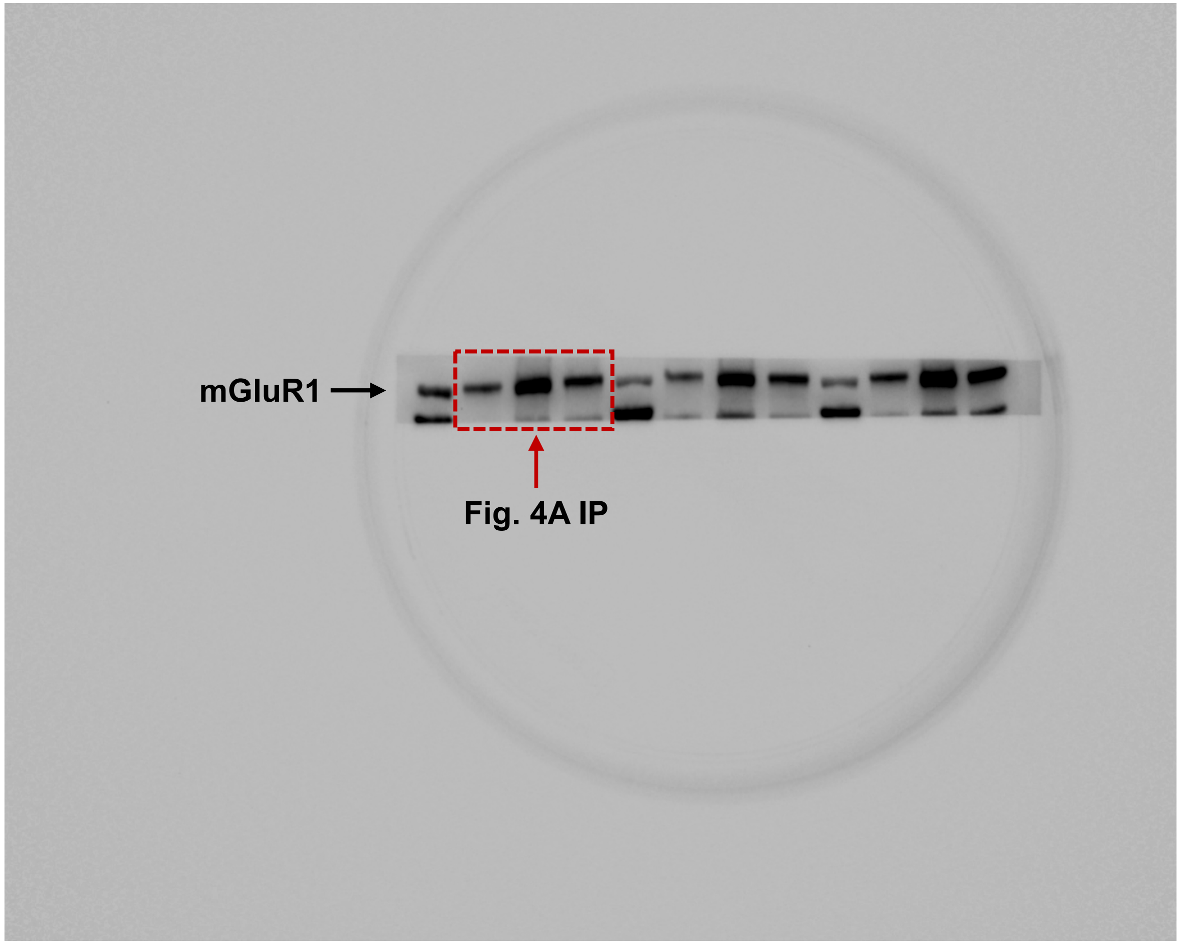
mGluR1
Fig. 4A IP

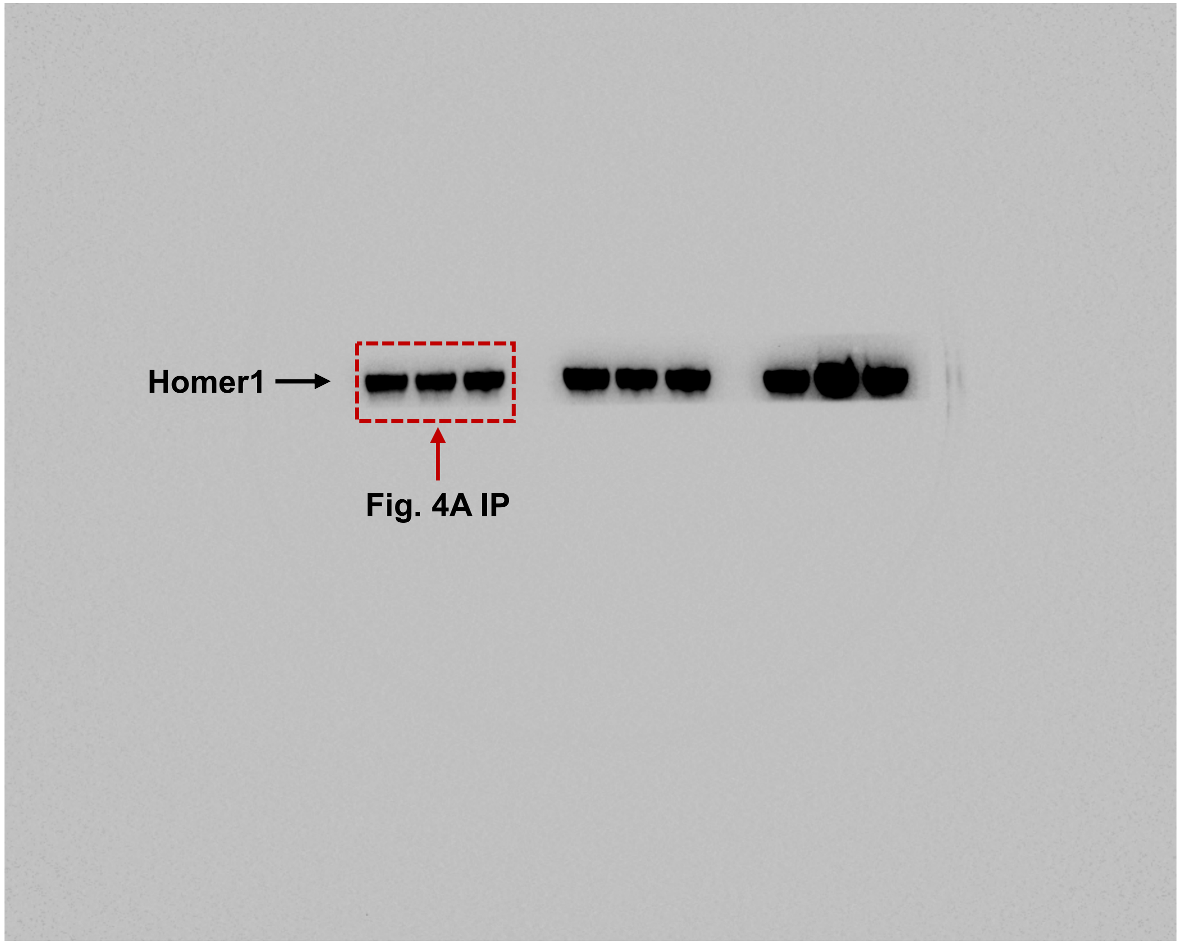
Homer1
Fig. 4A IP

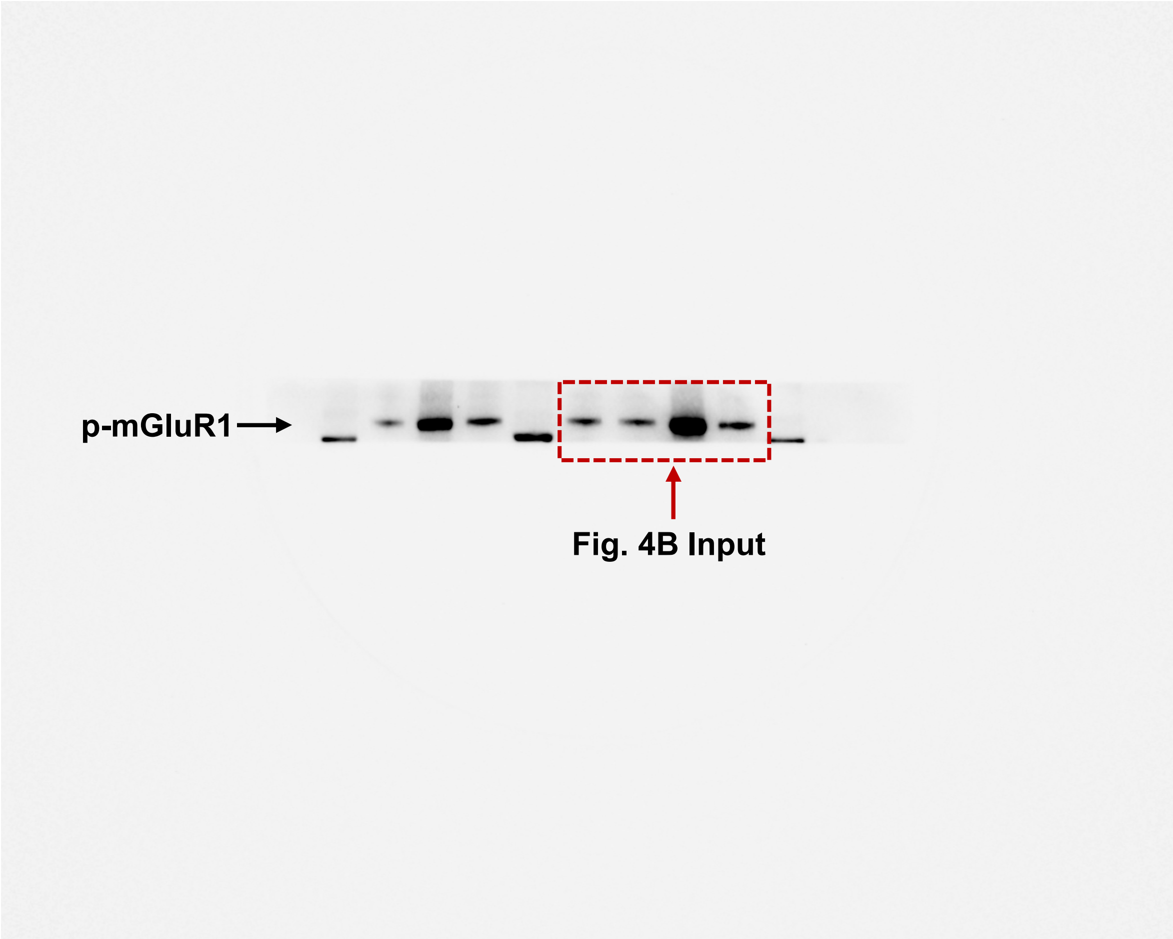
p-mGluR1
Fig. 4B Input

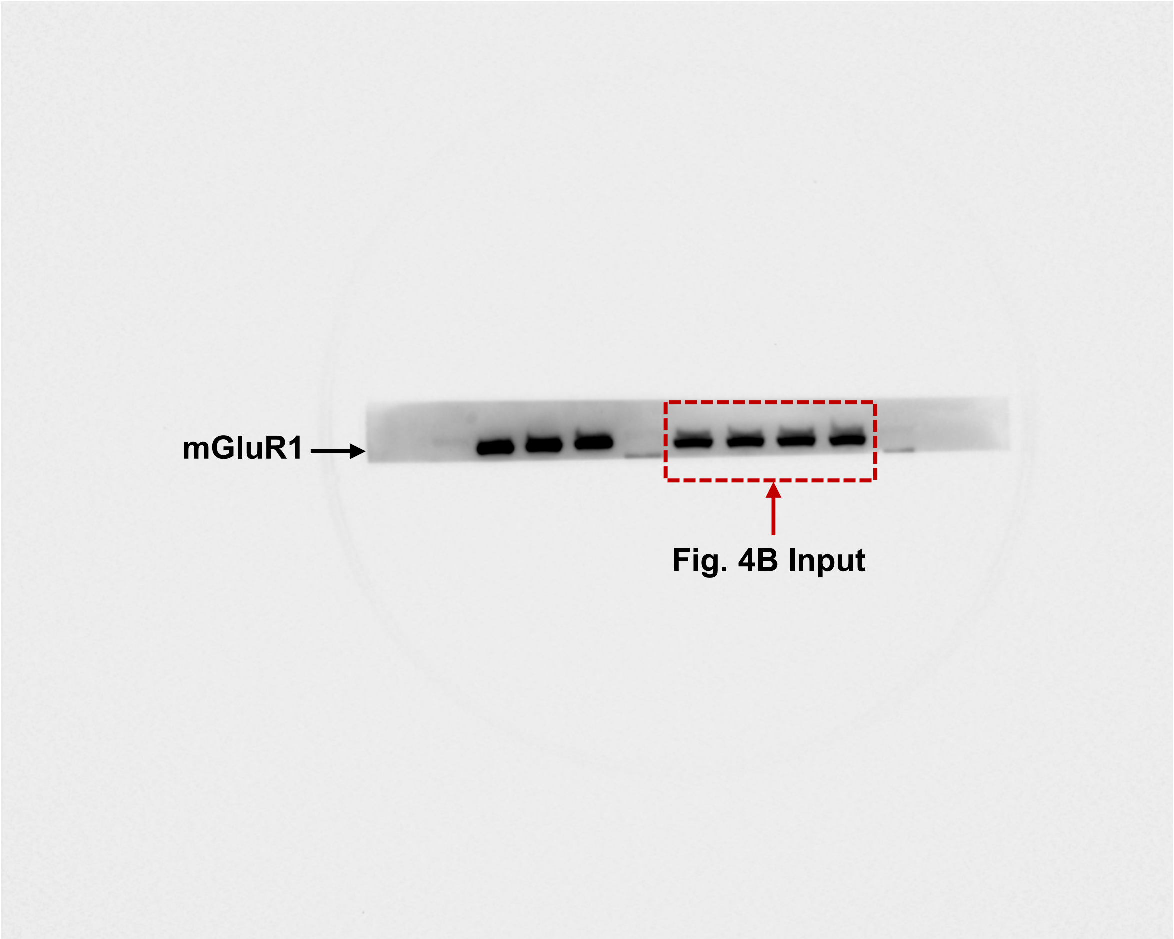
mGluR1
Fig. 4B Input

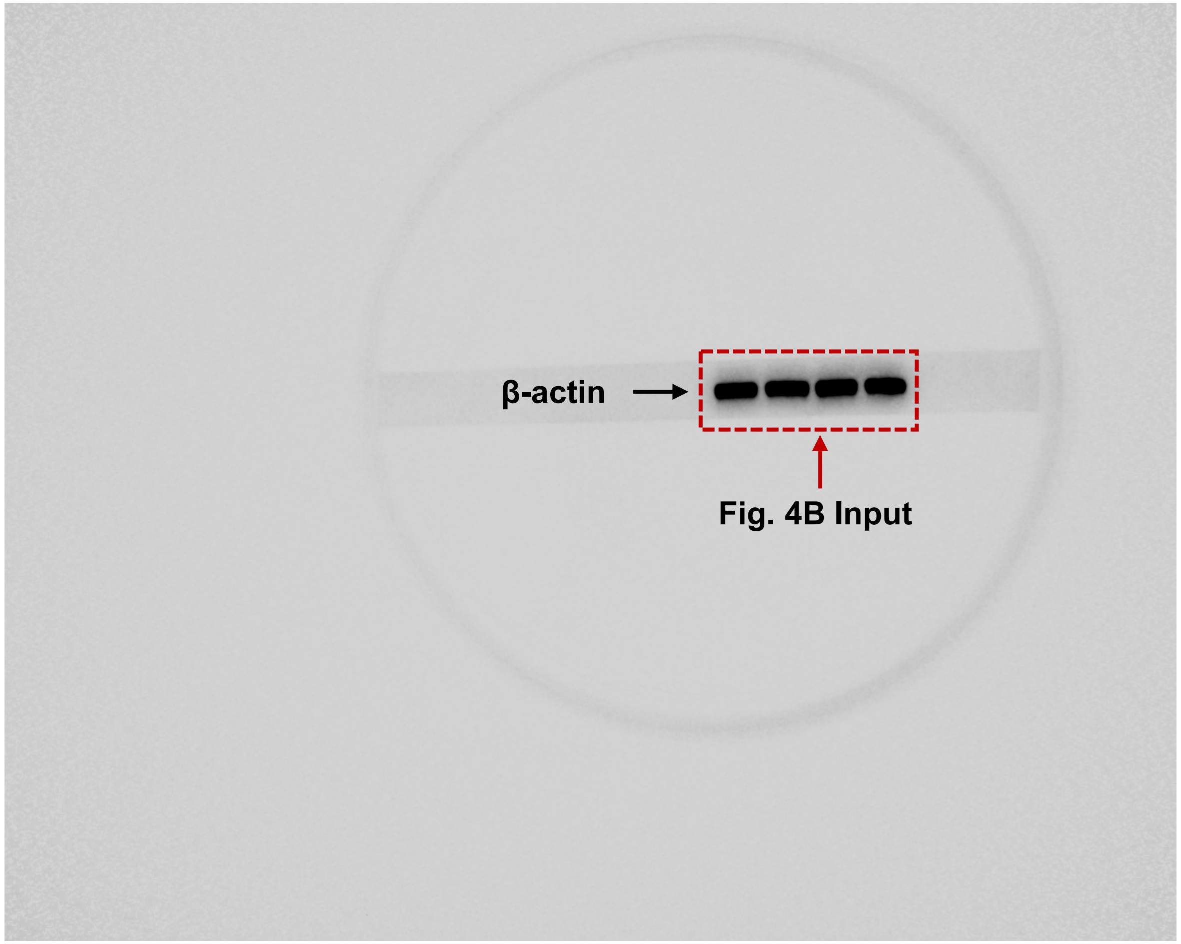
β-actin
Fig. 4B Input

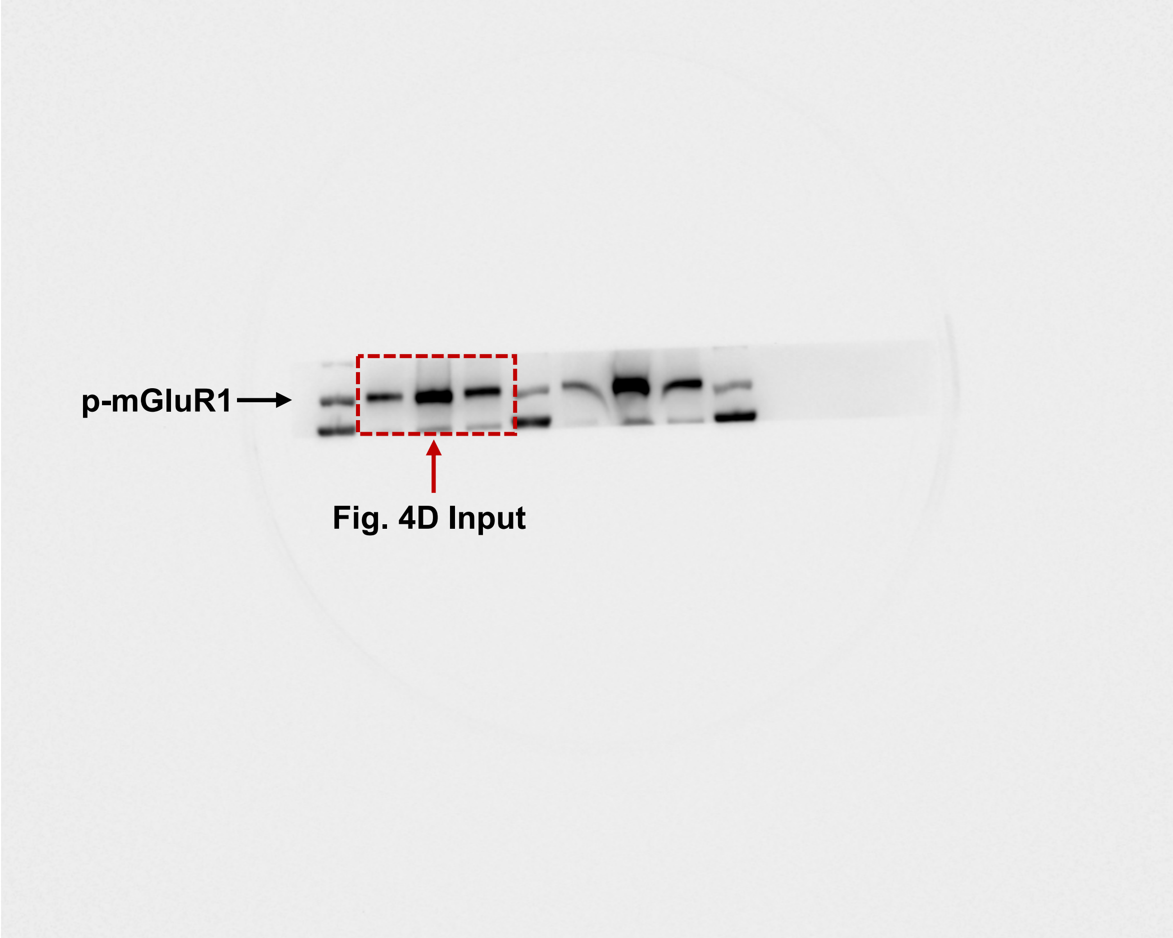
p-mGluR1
Fig. 4D Input

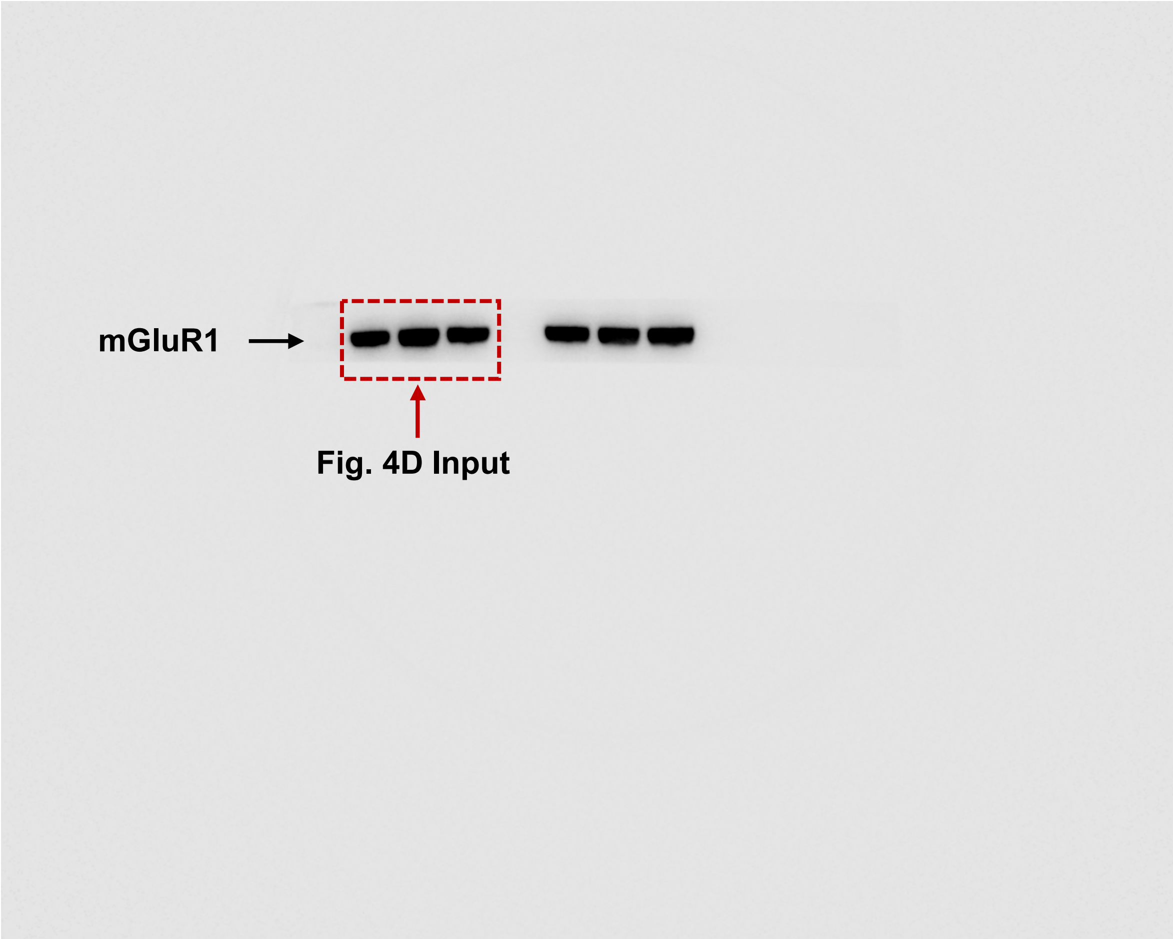
mGluR1
Fig. 4D Input

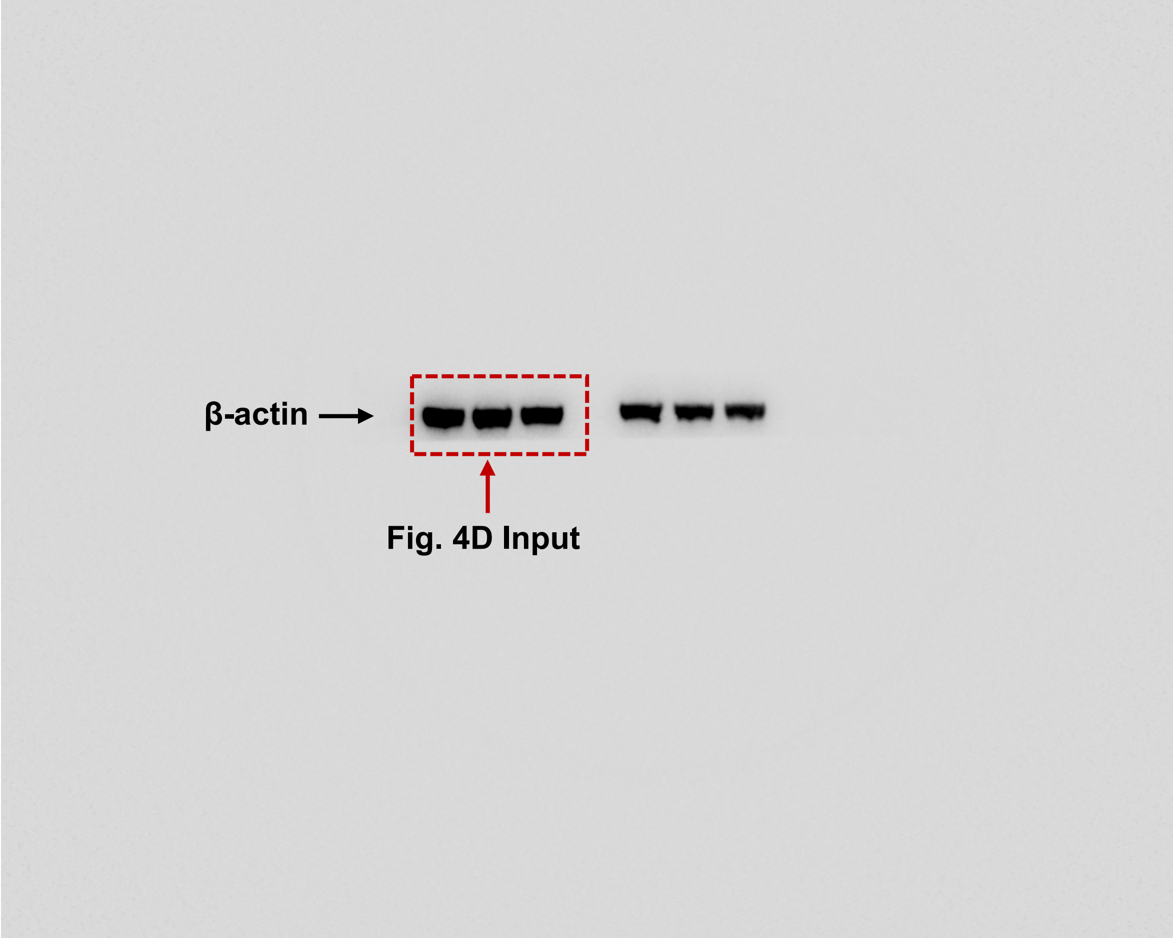
β-actin
Fig. 4D Input

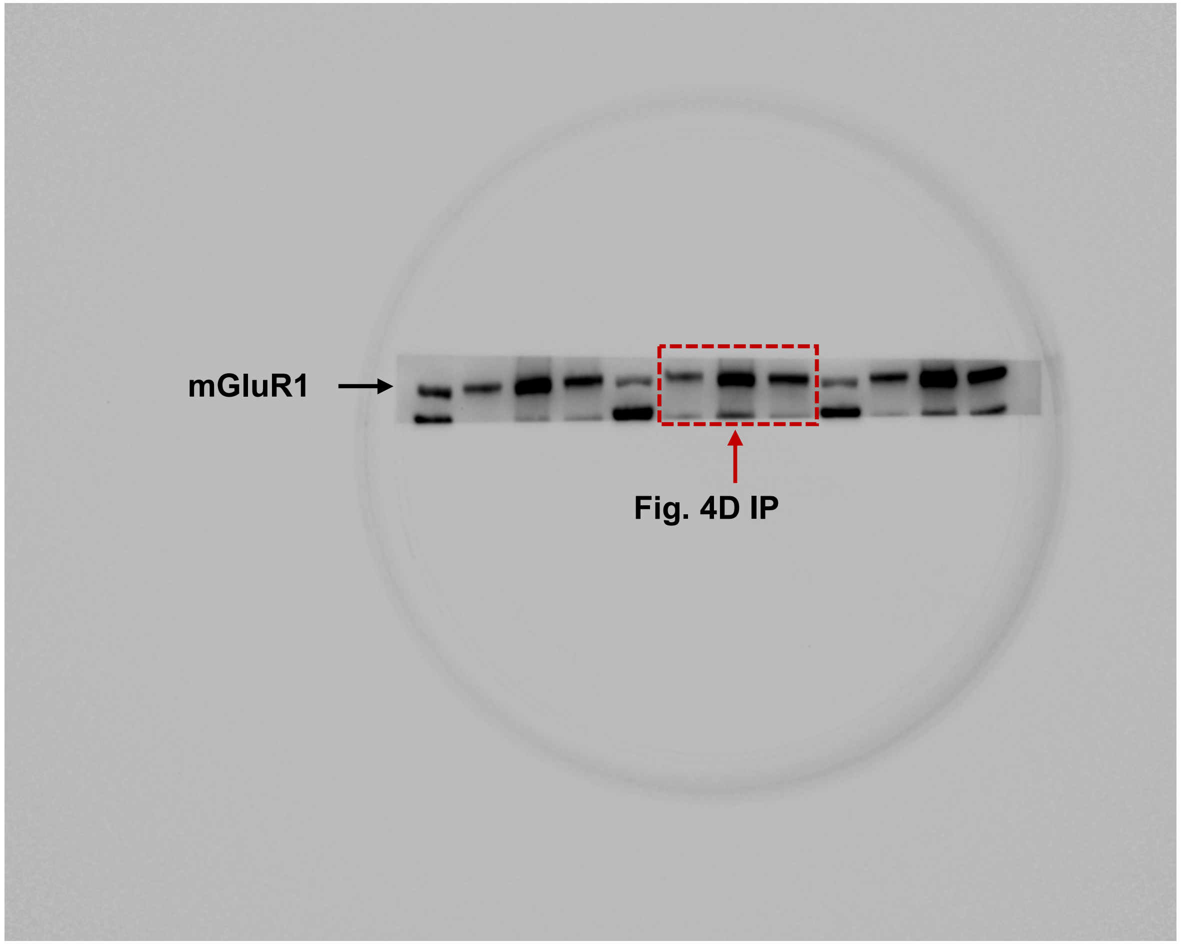

mGluR1
Fig. 4D IP

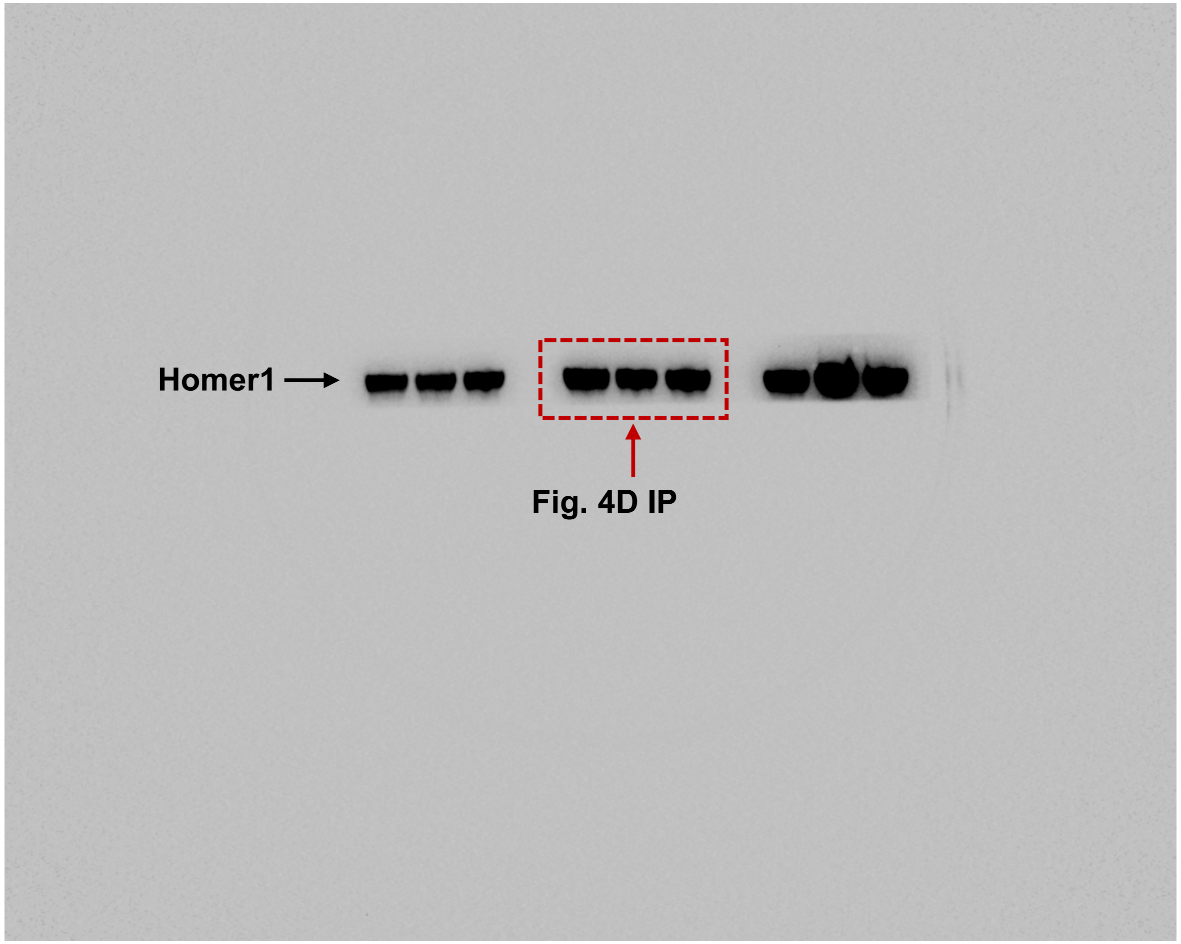
Homer1
Fig. 4D IP

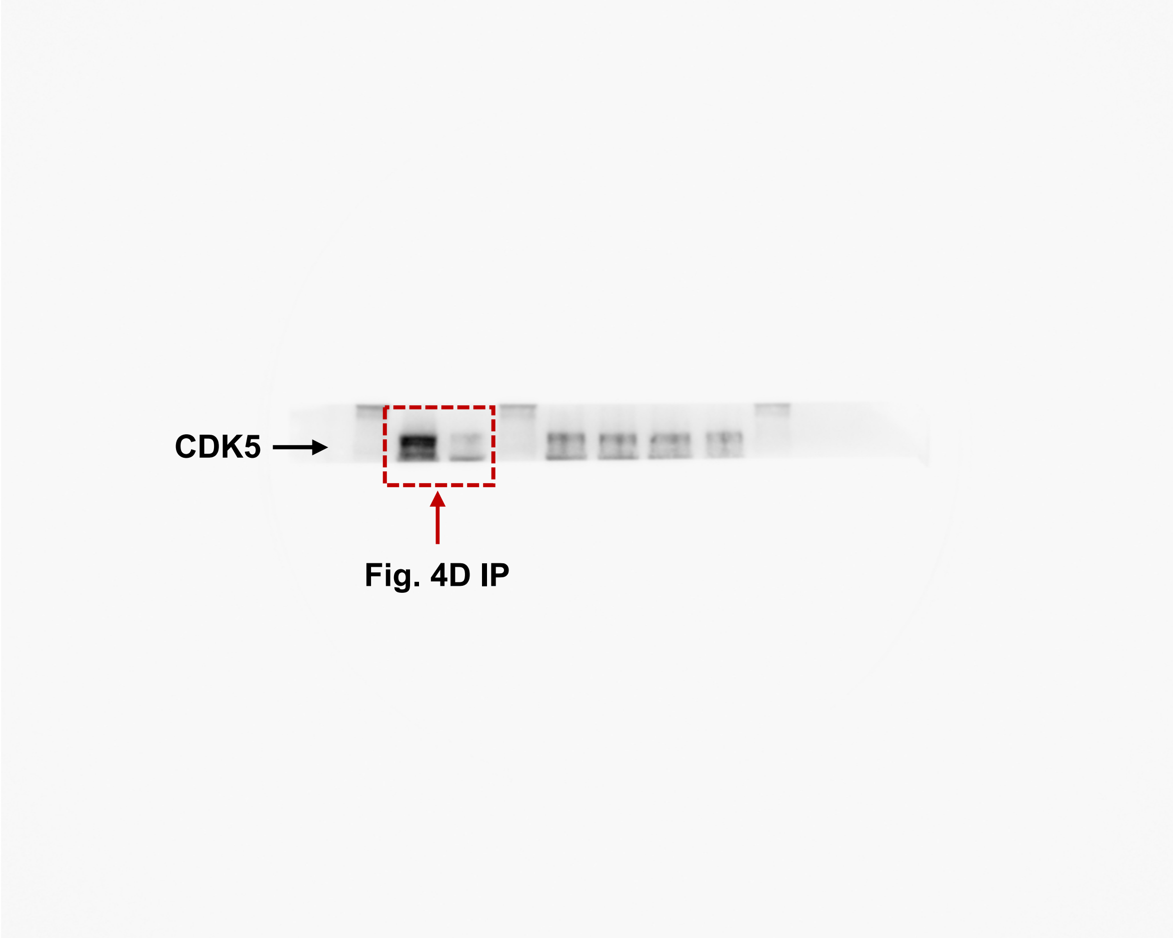
CDK5
Fig. 4D IP

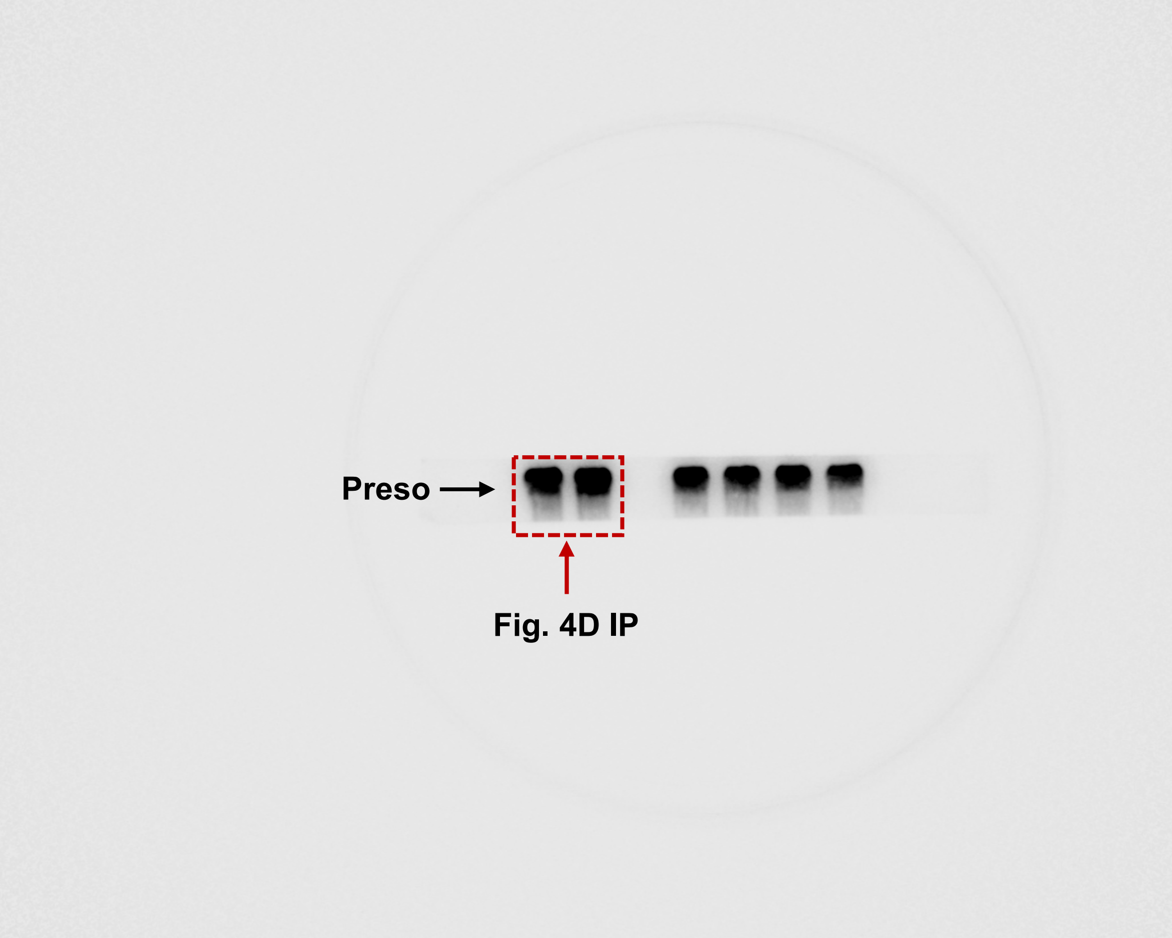
Preso
Fig. 4D IP

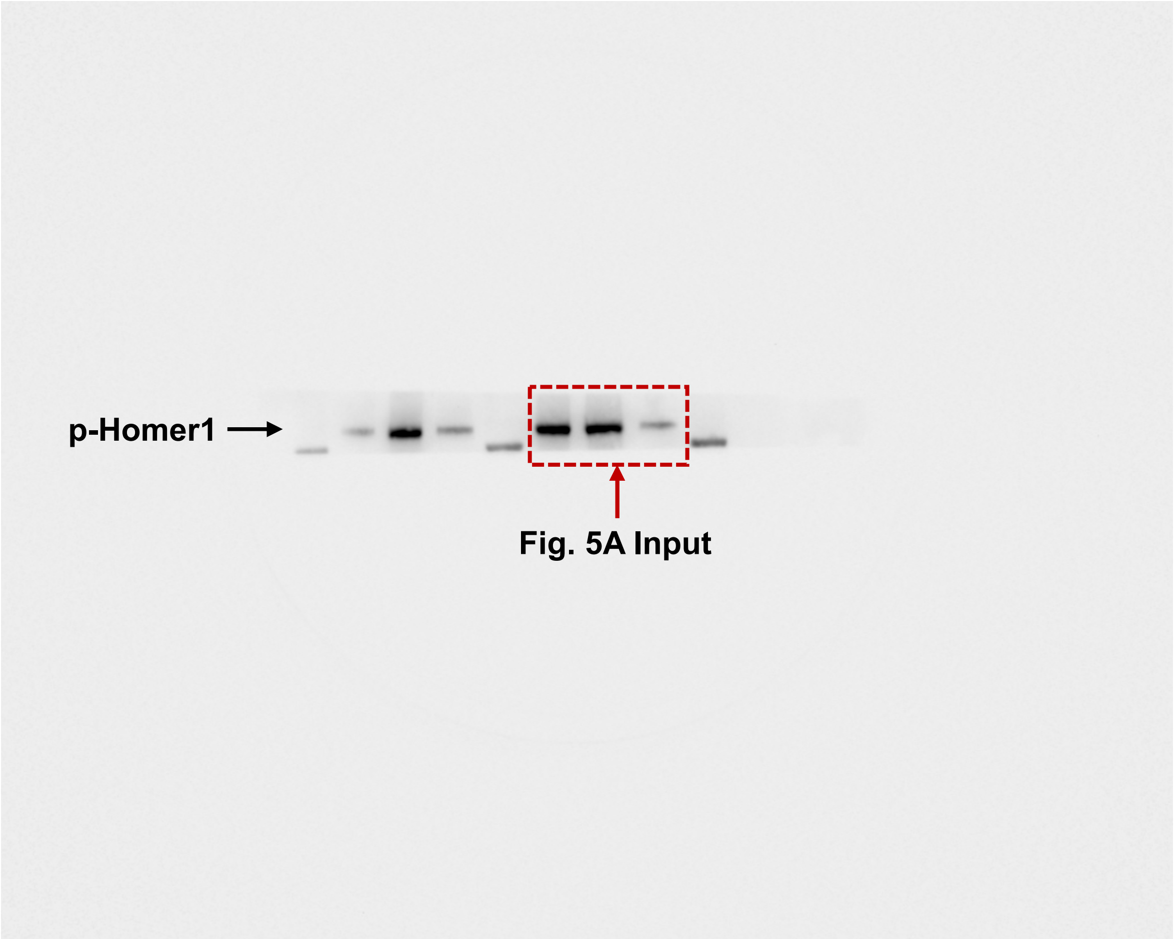
p-Homer1
Fig. 5A Input

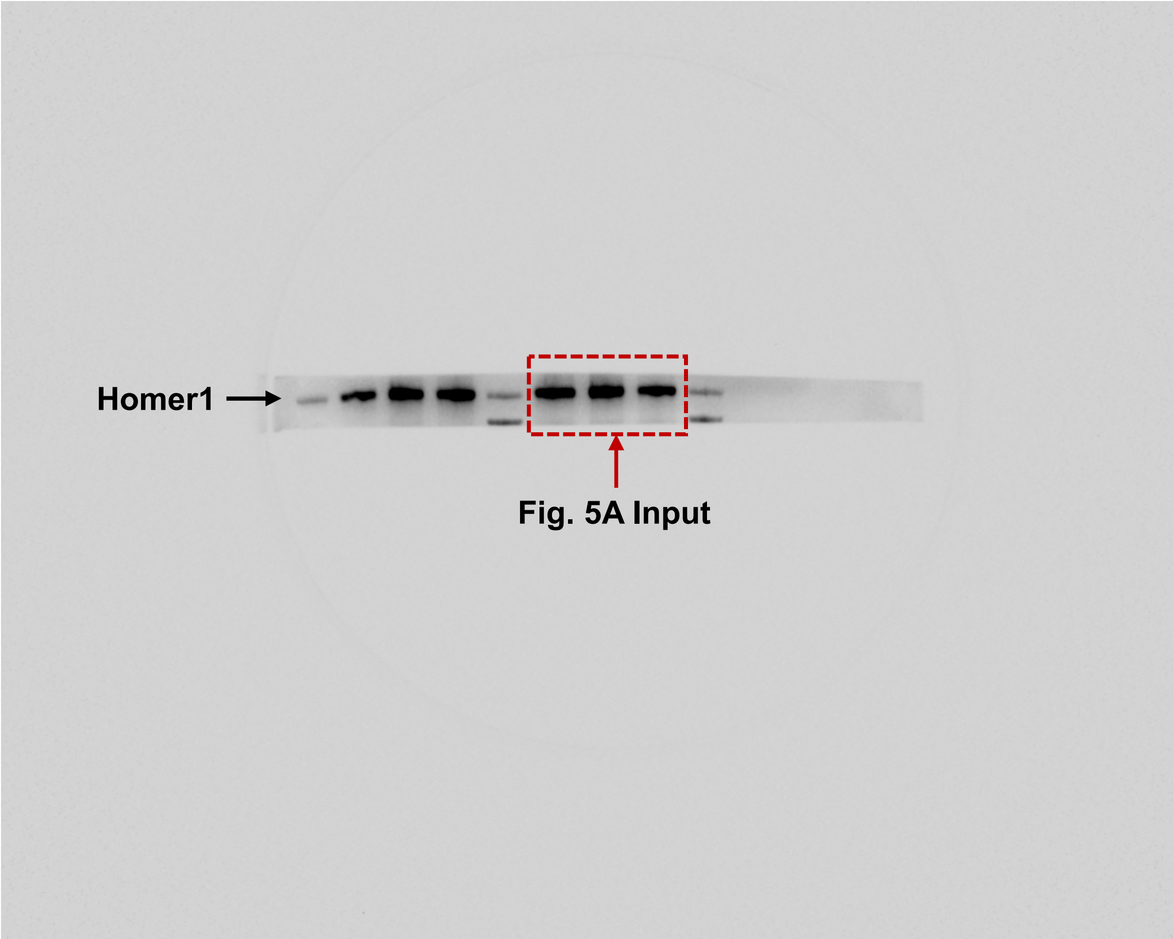
Homer1
Fig. 5A Input

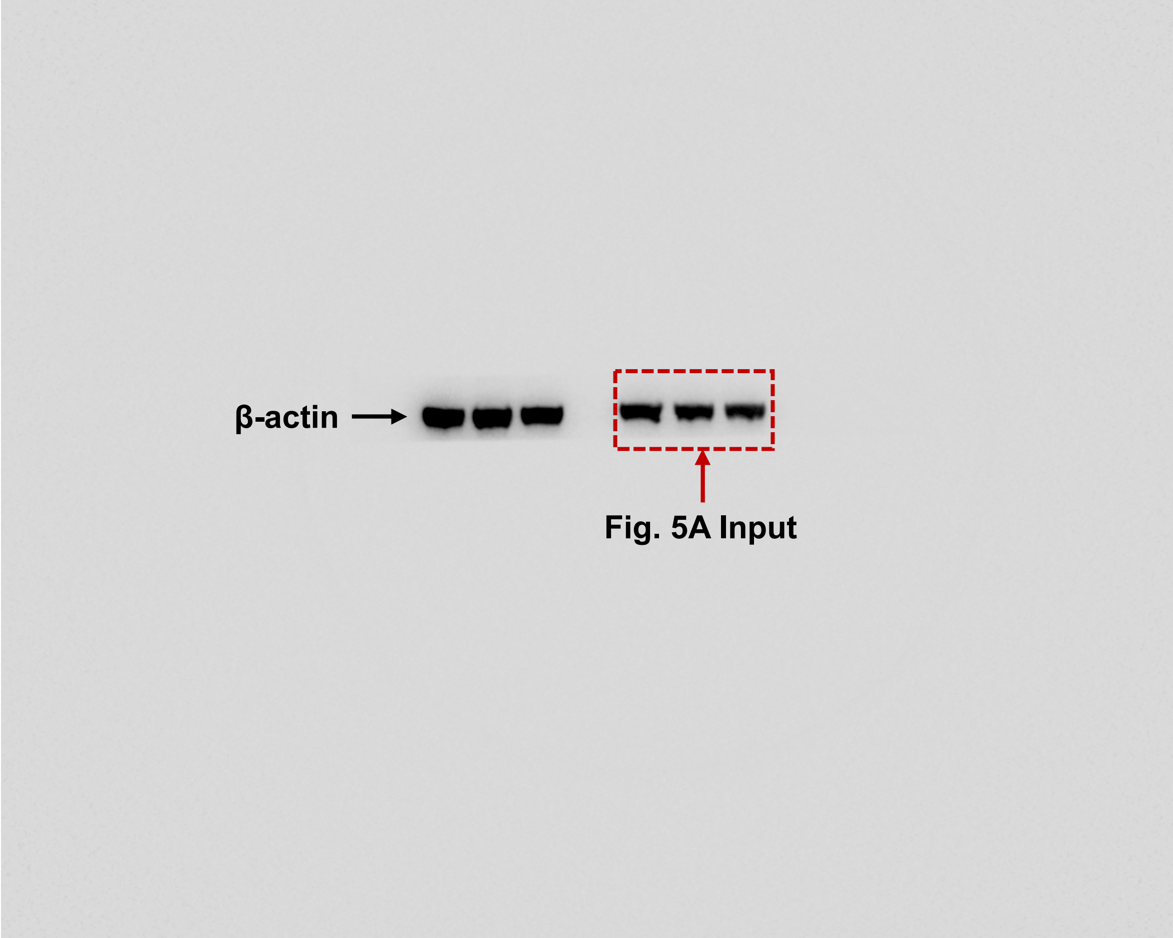
β-actin
Fig. 5A Input

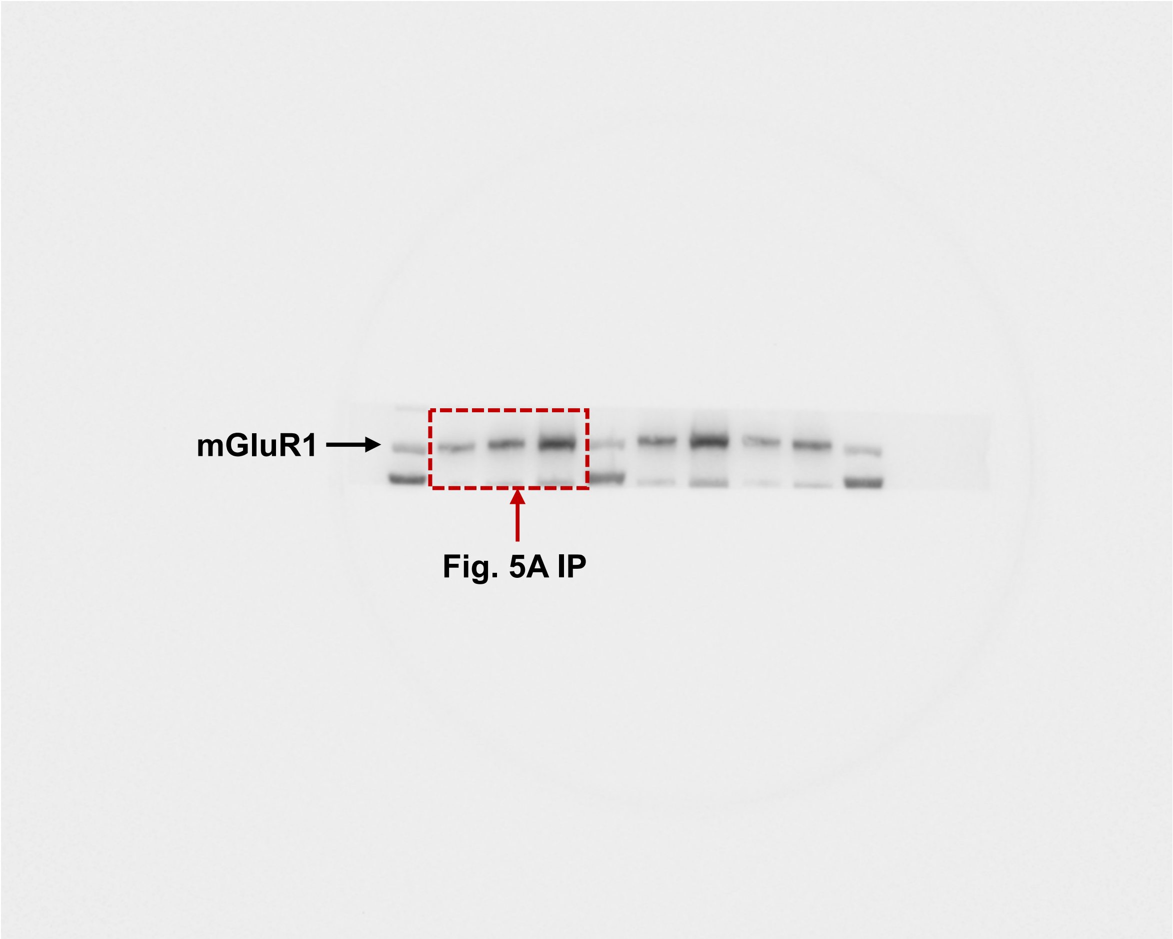
mGluR1
Fig. 5A IP

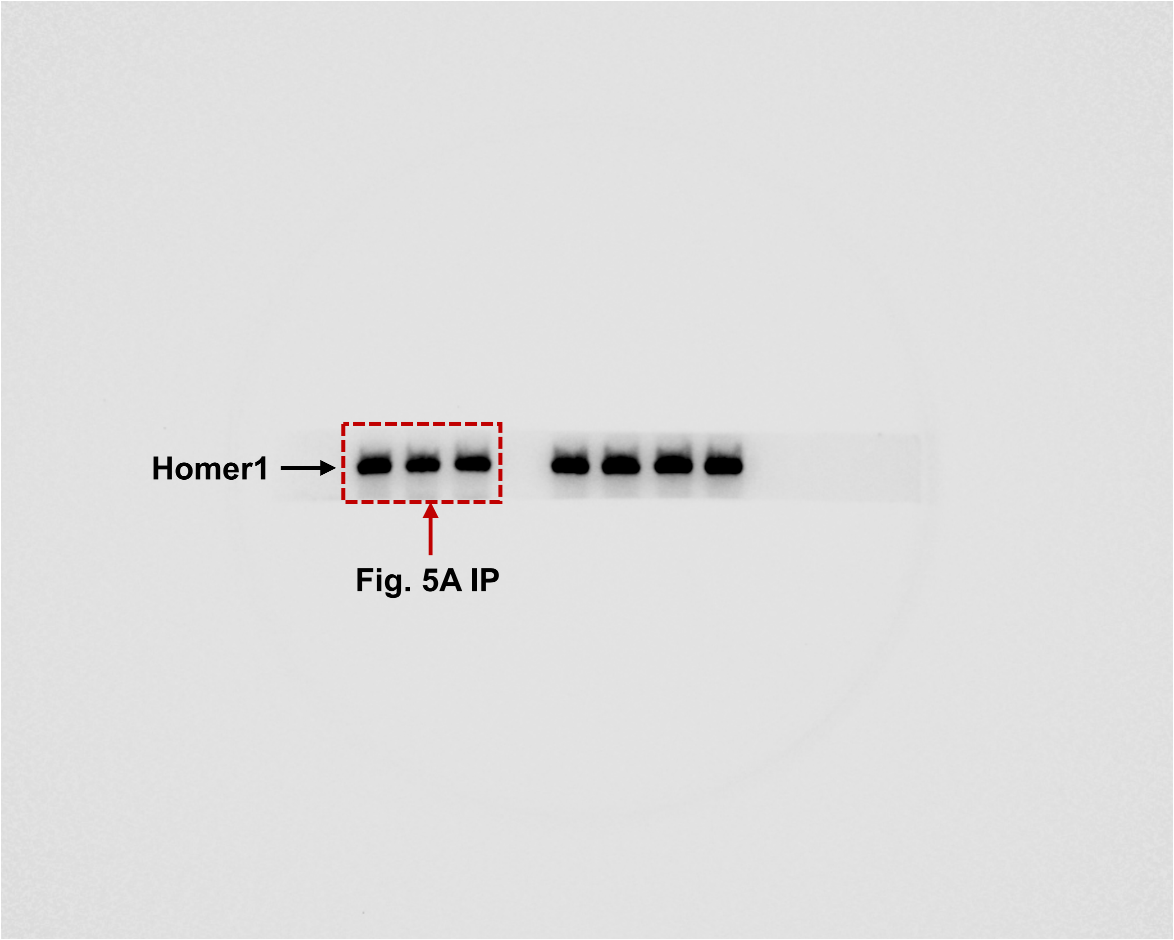

Homer1
Fig. 5A IP

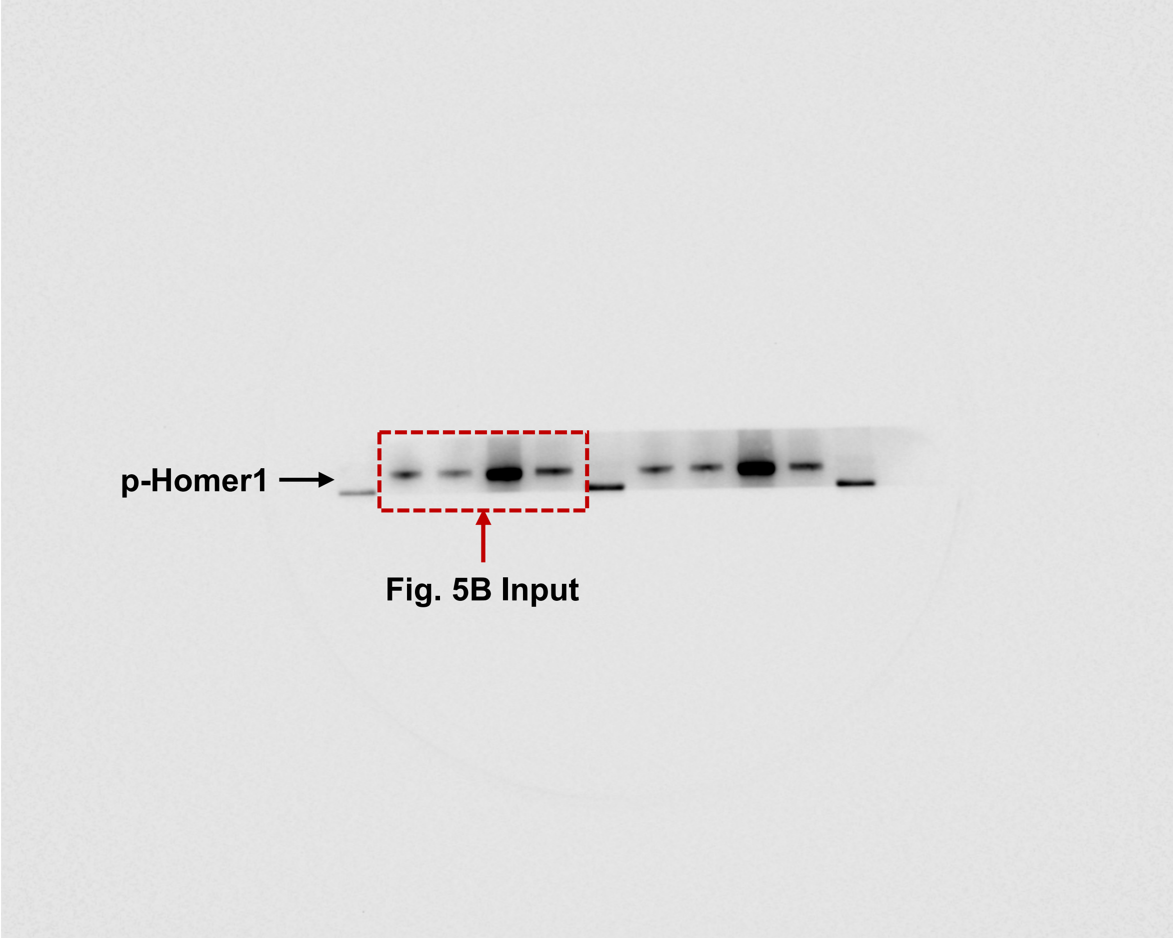
p-Homer1
Fig. 5B Input

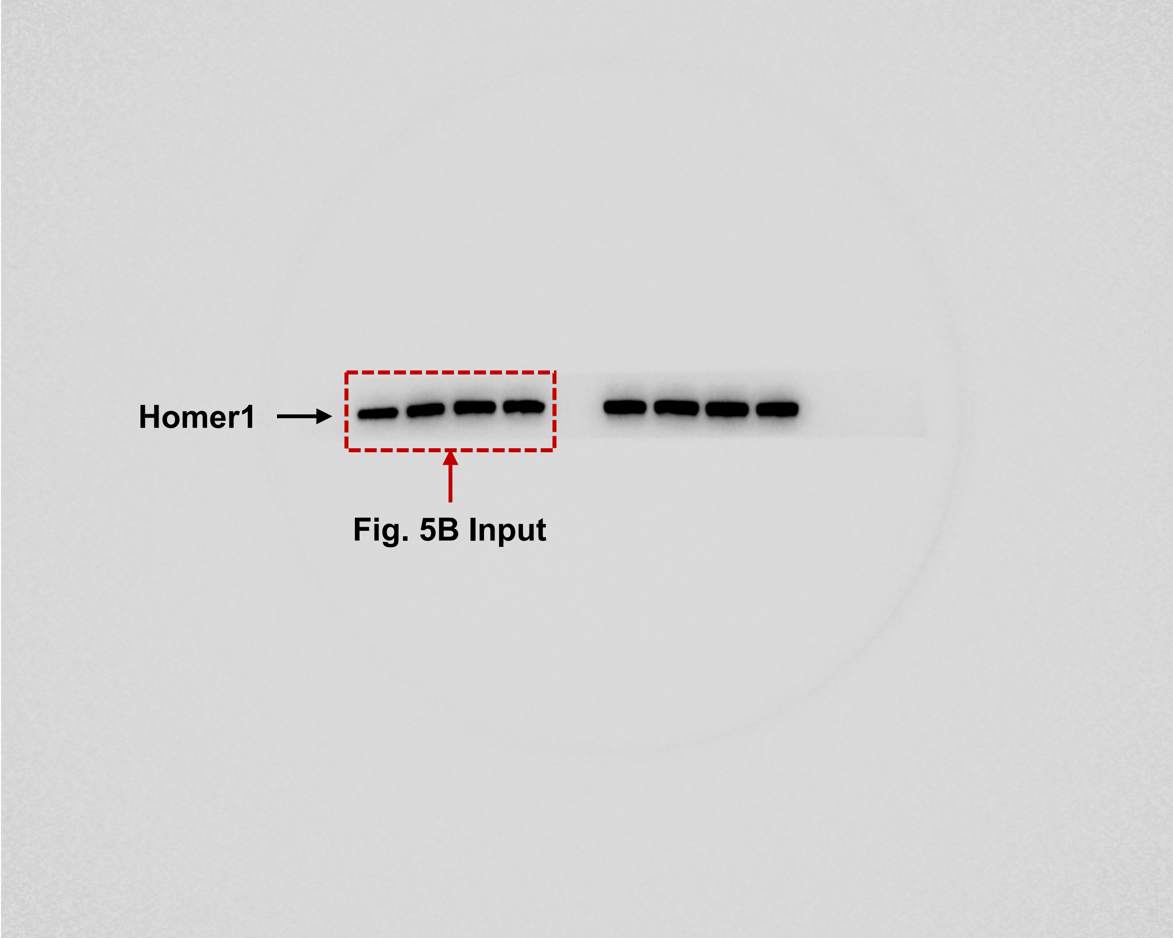
Homer1
Fig. 5B Input

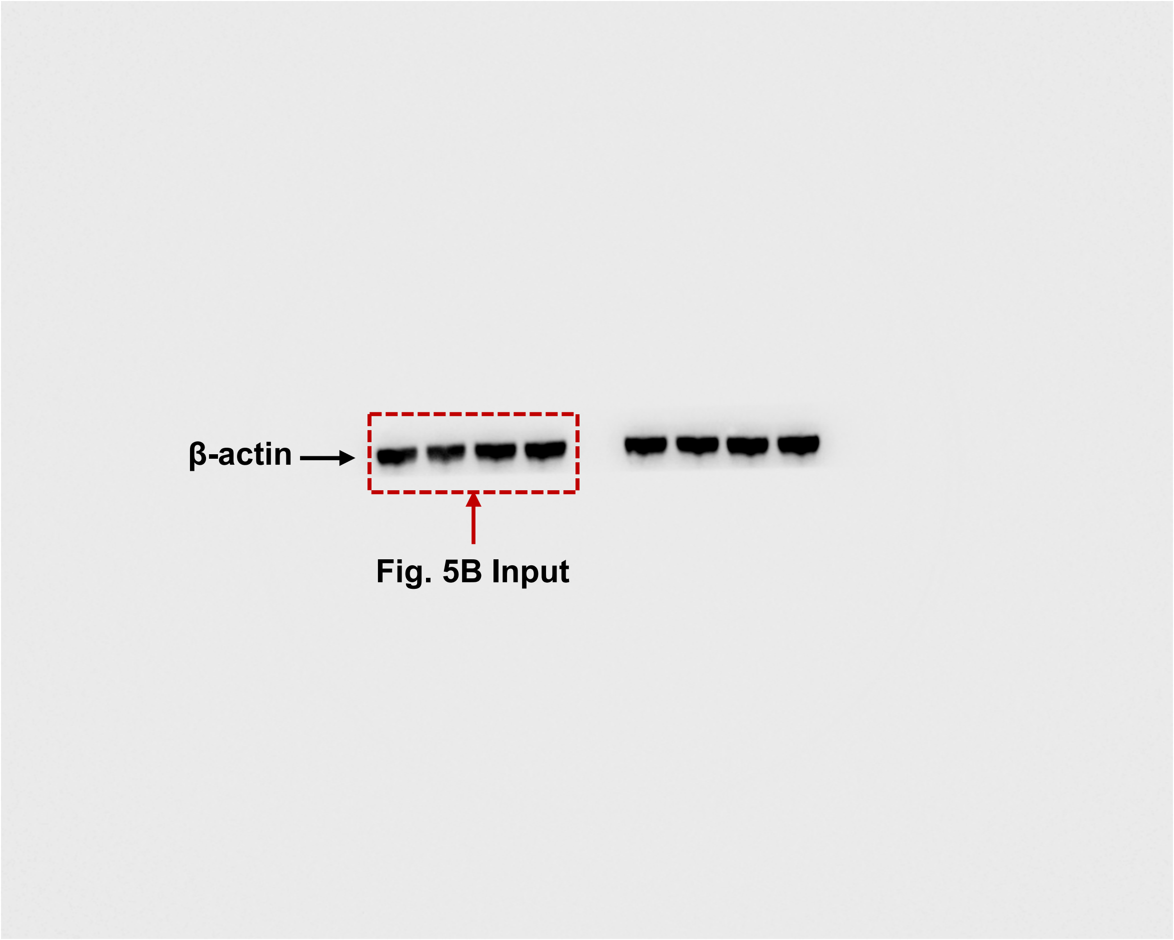
β-actin
Fig. 5B Input

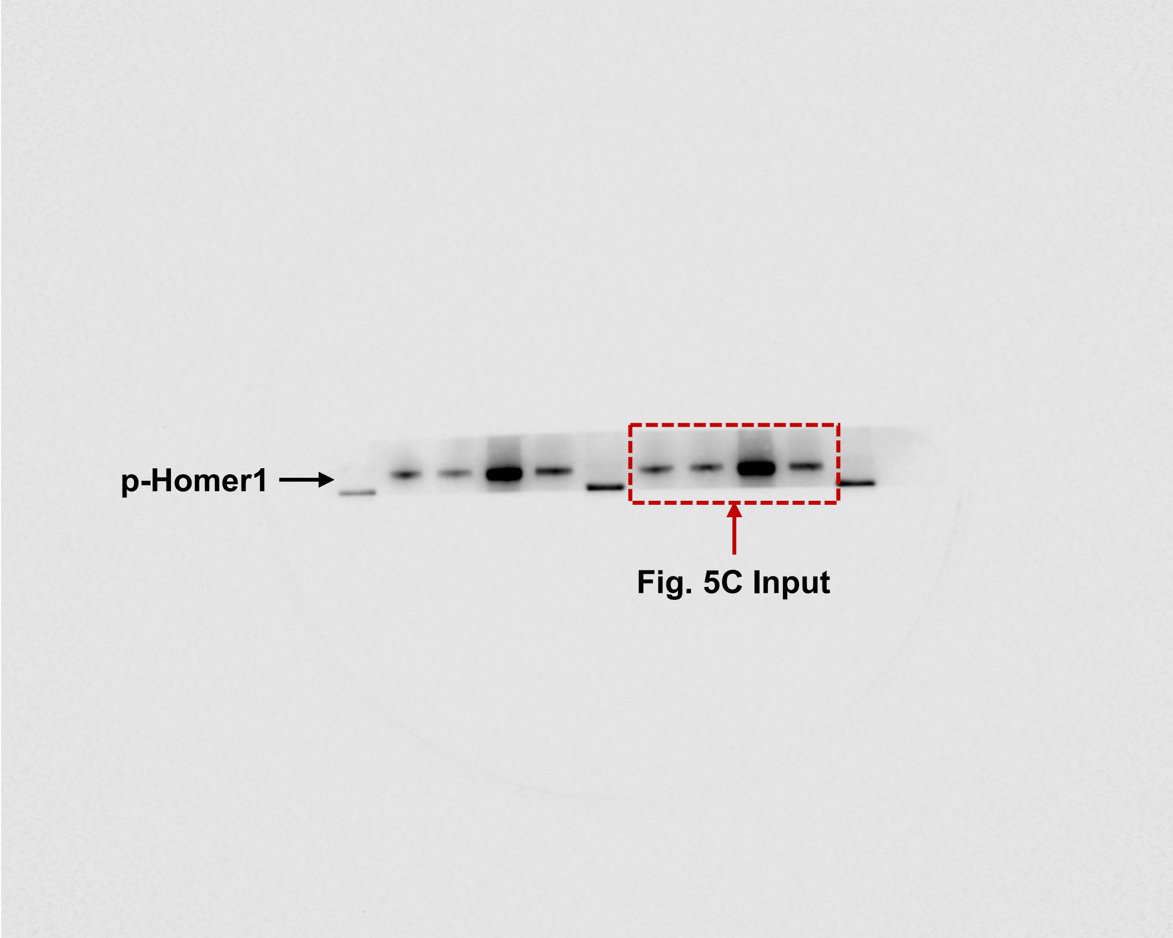
p-Homer1
Fig. 5C Input

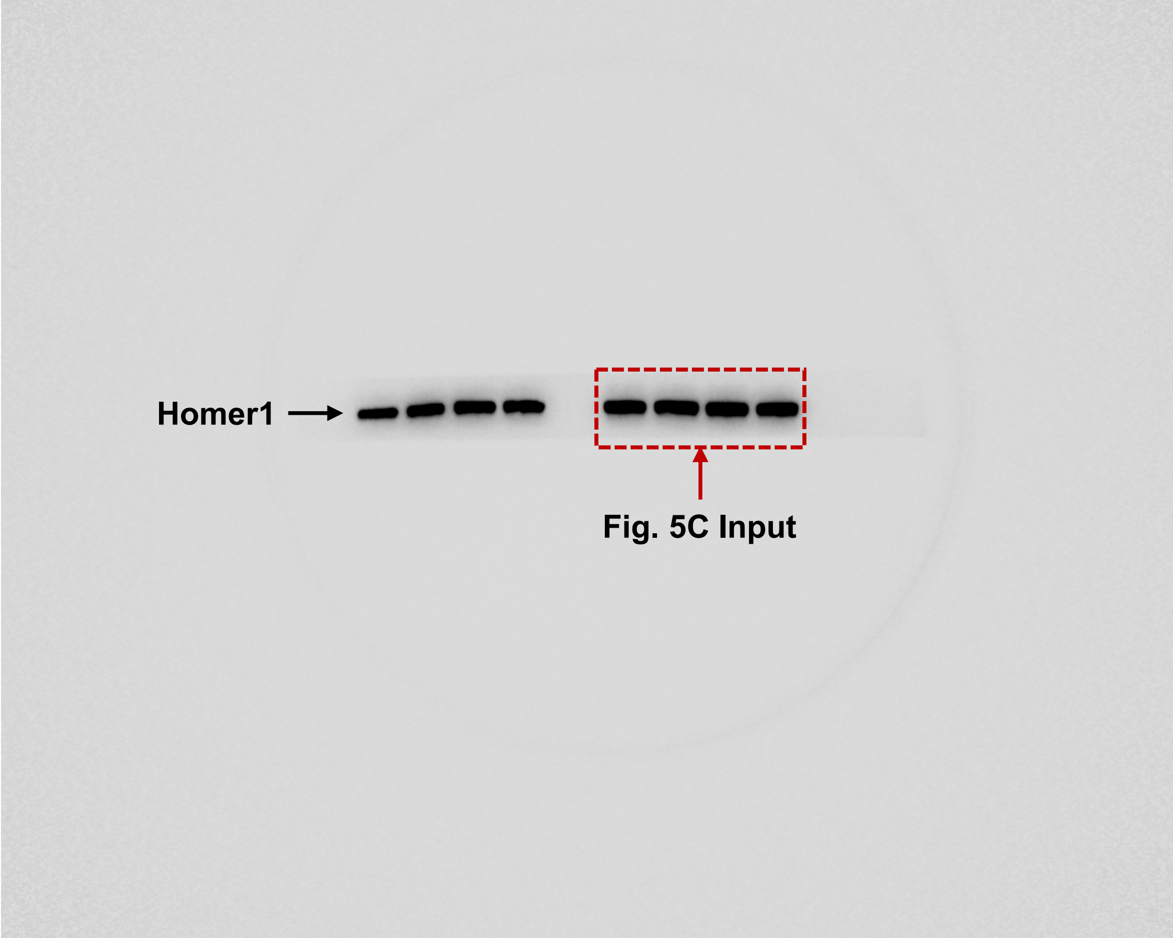
Homer1
Fig. 5C Input

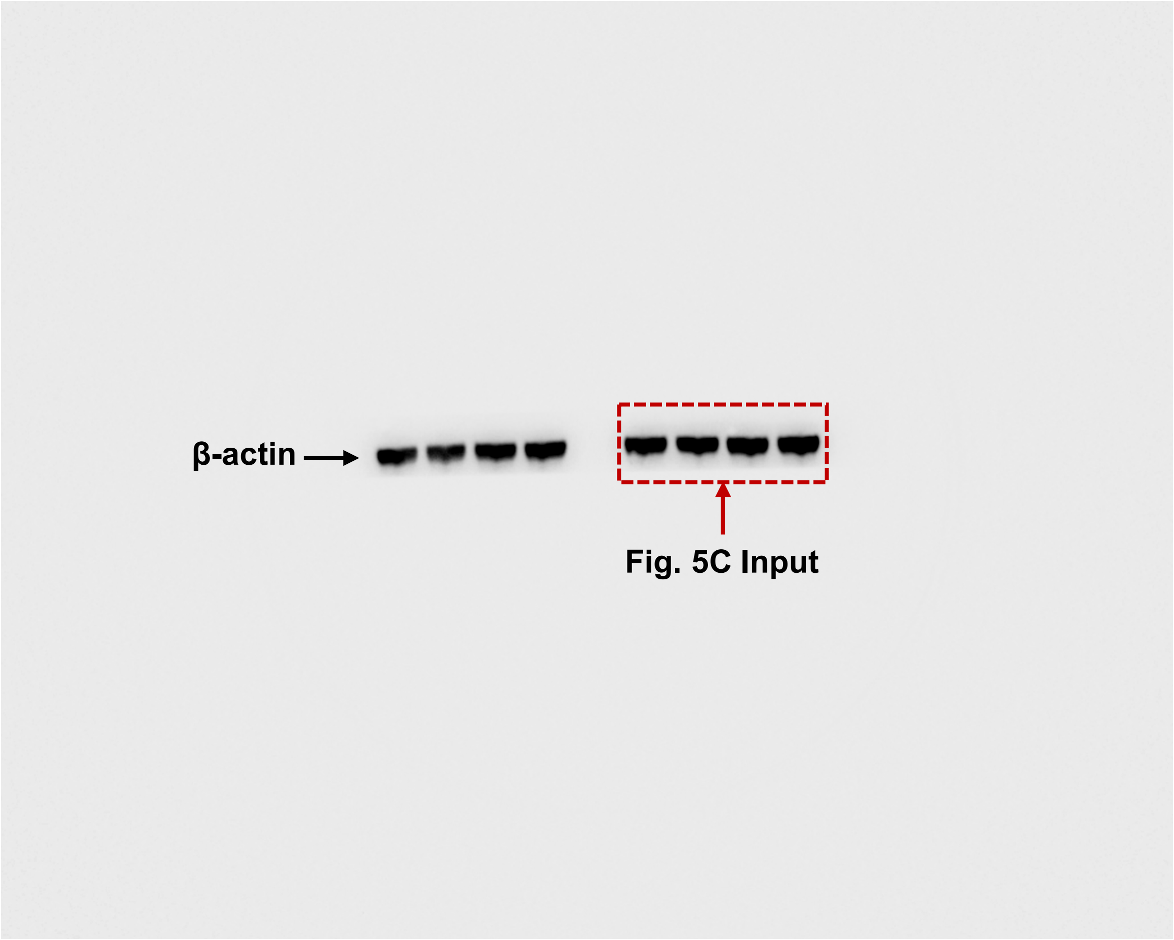
β-actin
Fig. 5C Input

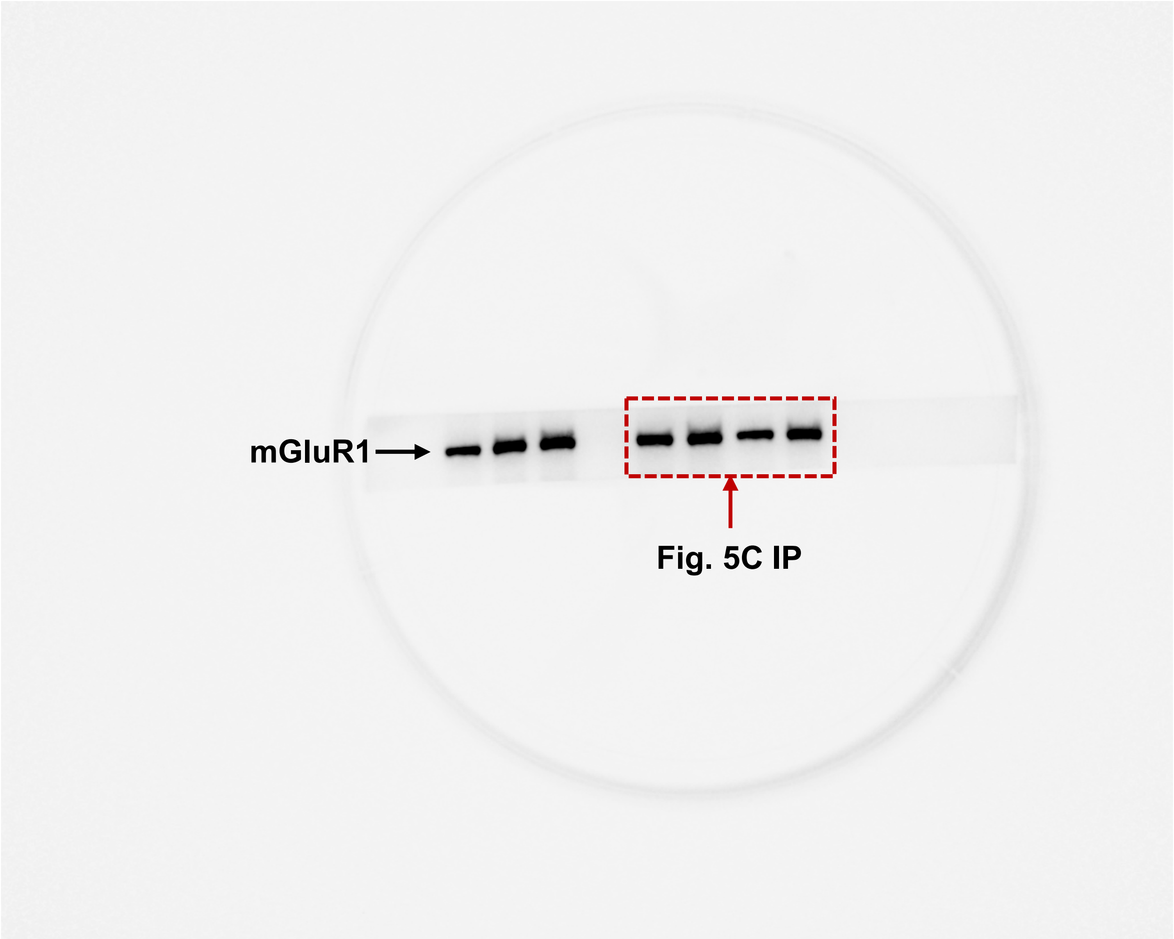
mGluR1
Fig. 5C IP

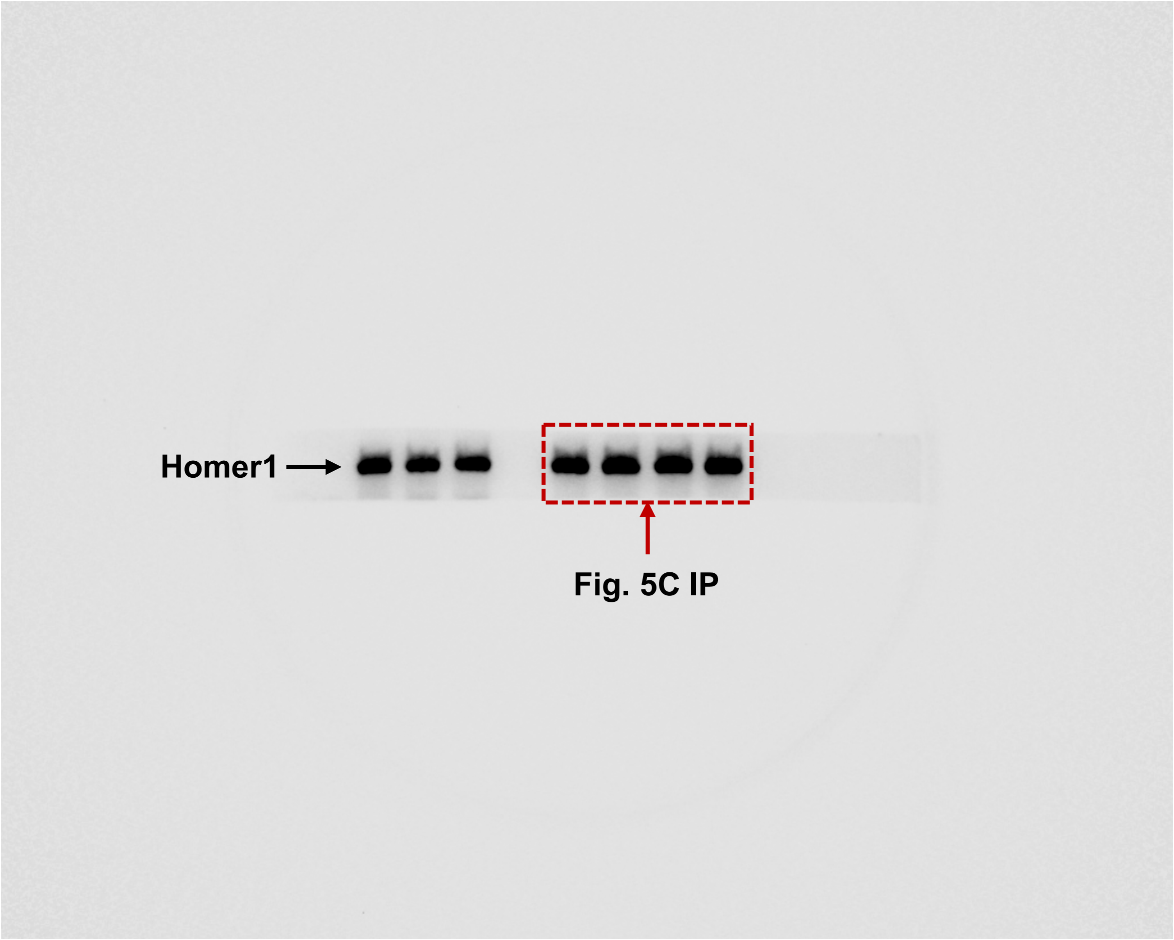

Homer1
Fig. 5C IP

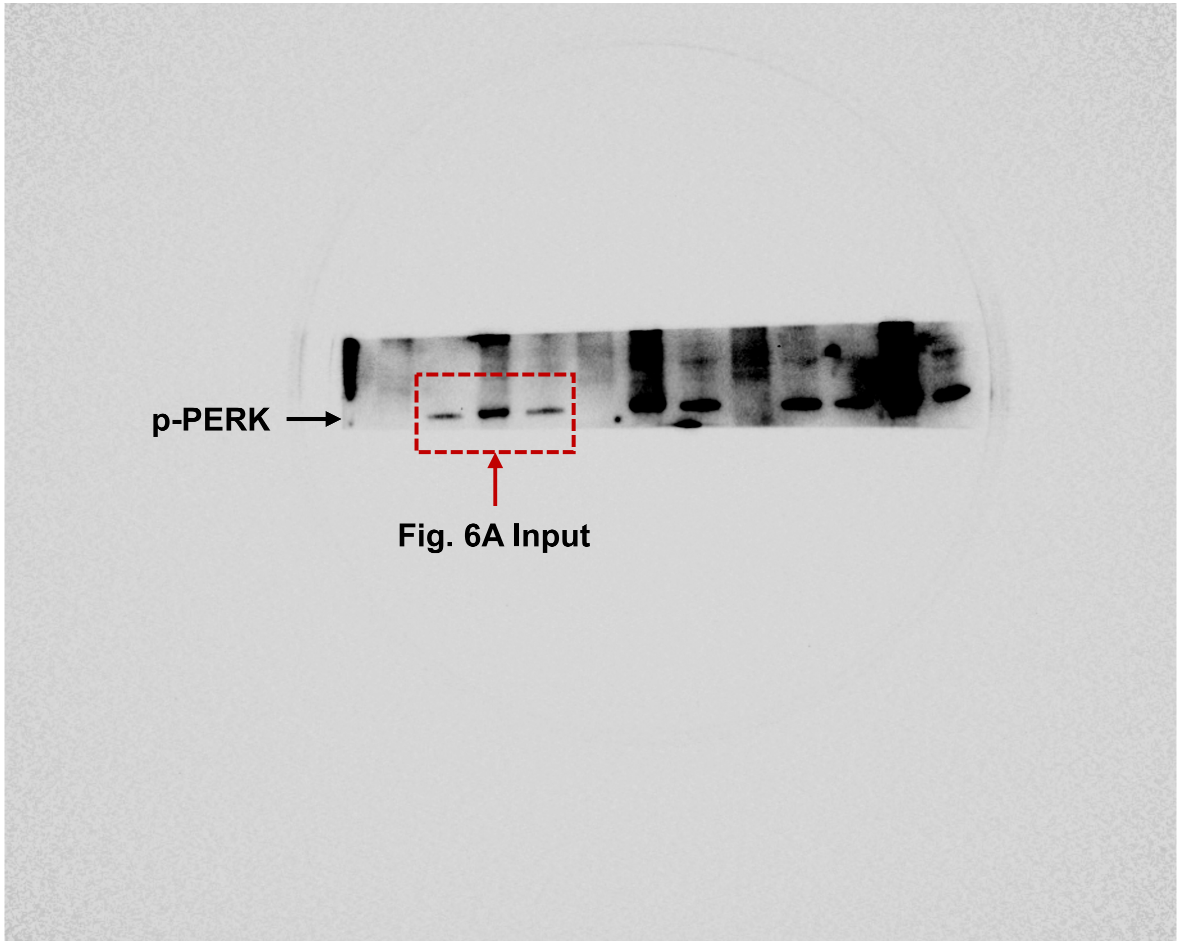
p-PERK
Fig. 6A Input

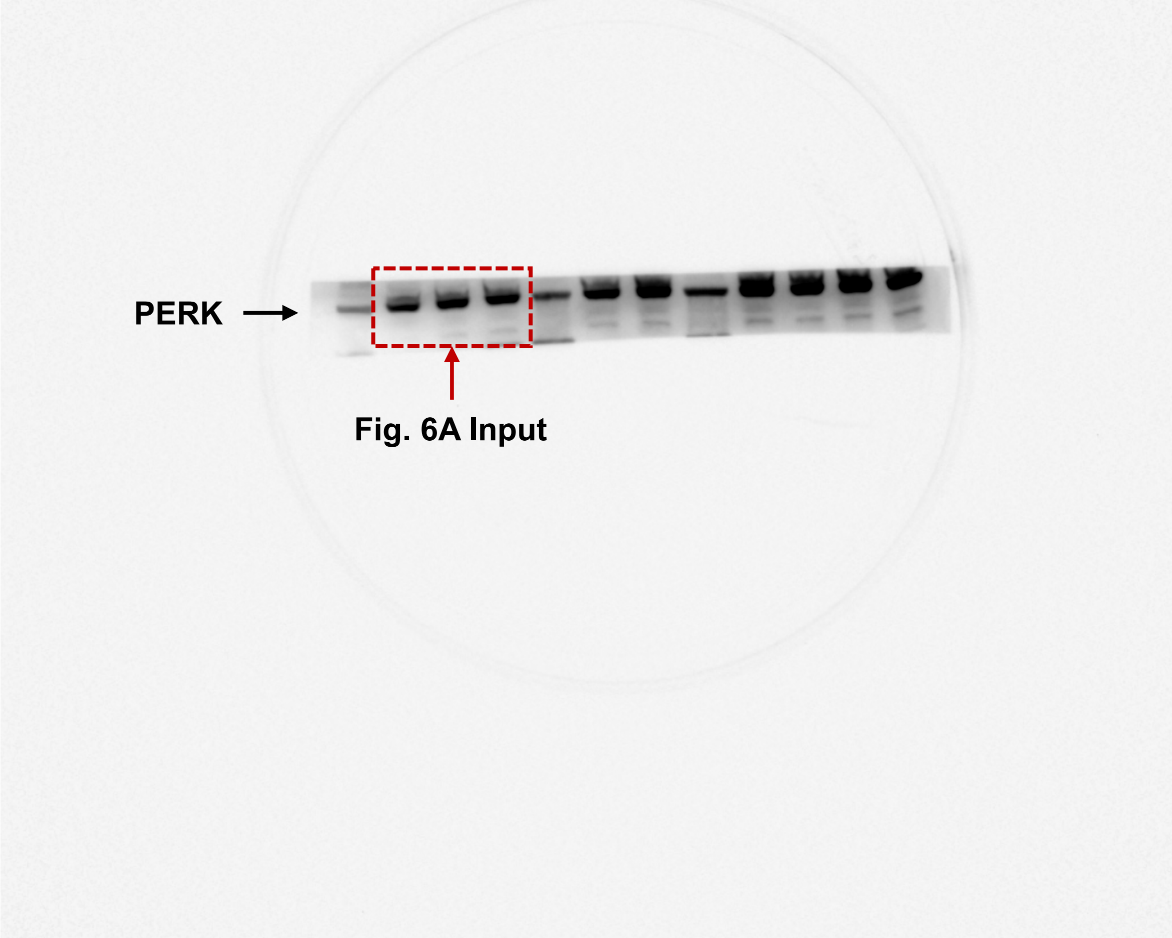
PERK
Fig. 6A Input

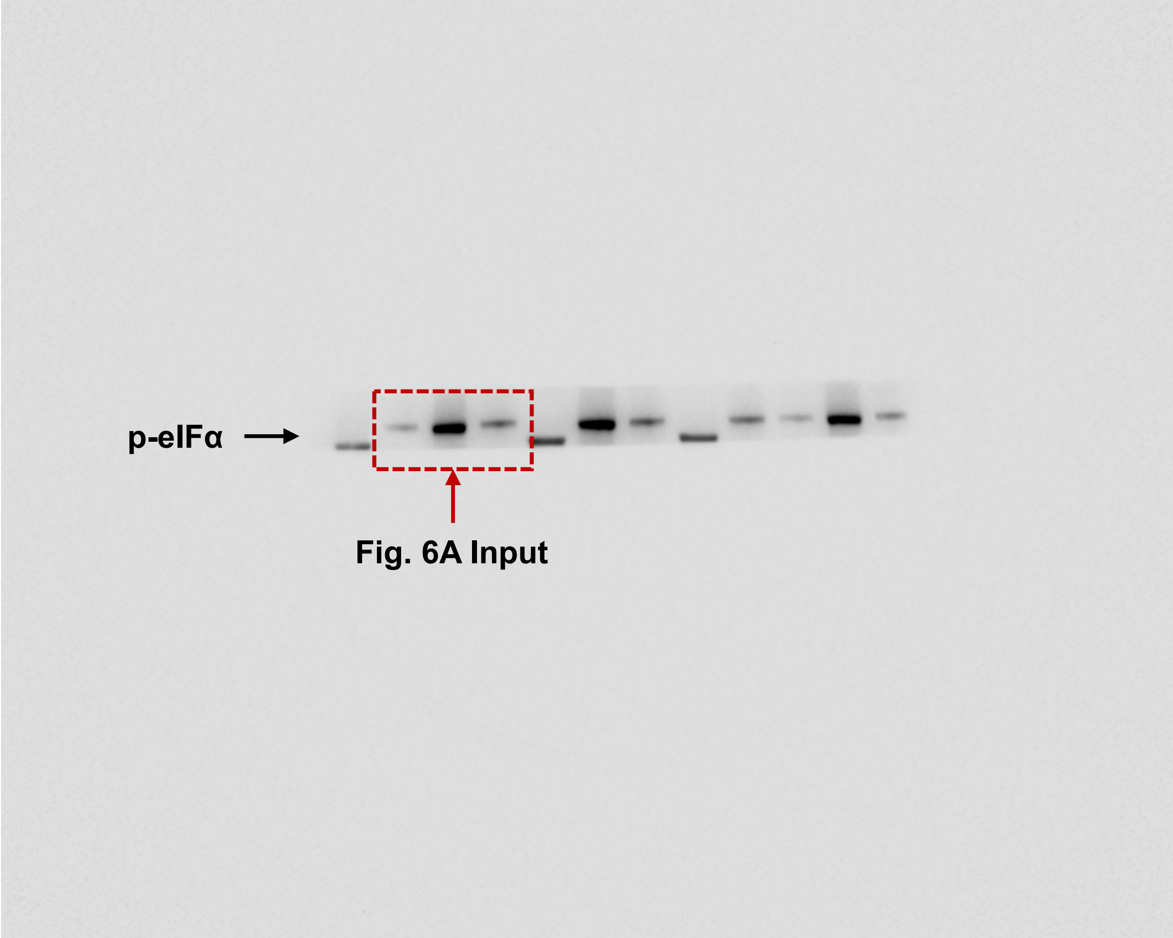
p-eIFα
Fig. 6A Input

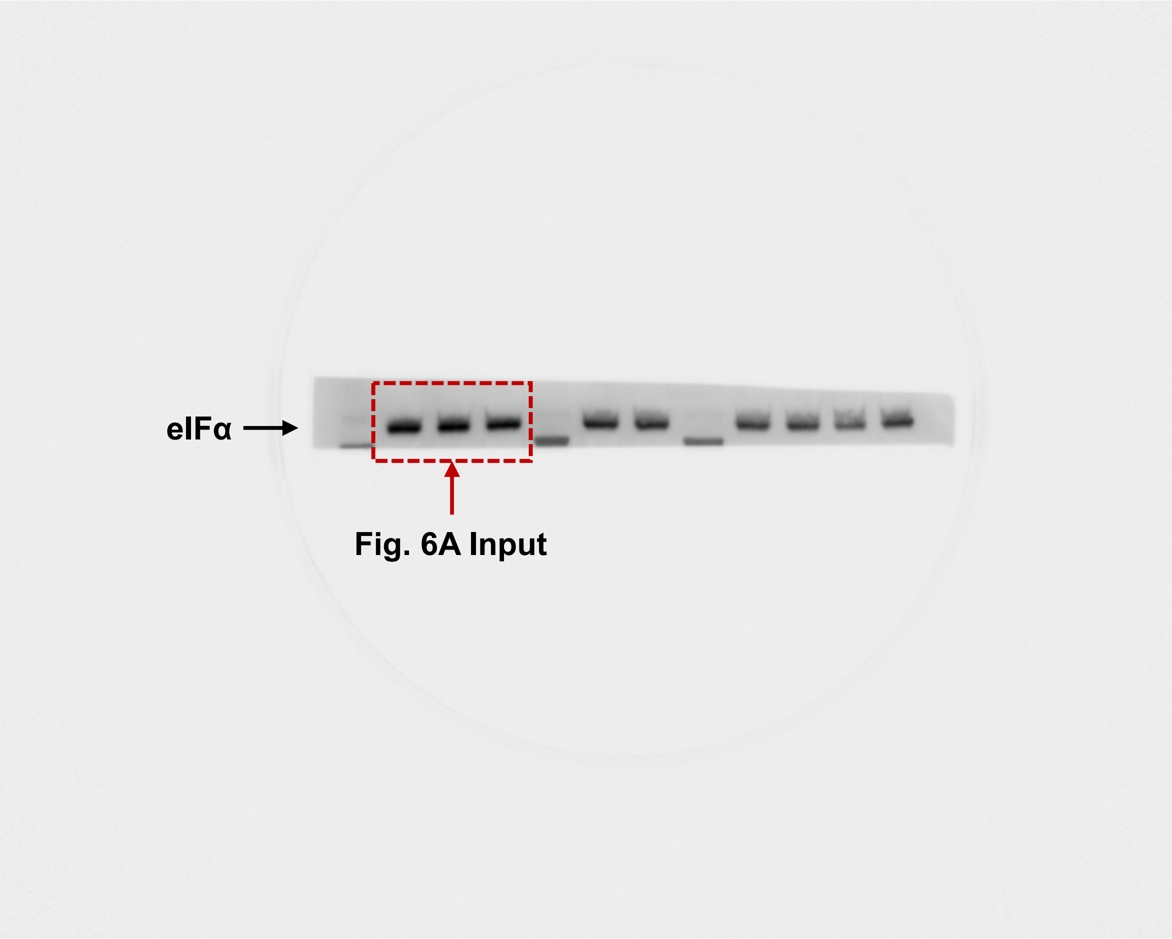
eIFα
Fig. 6A Input

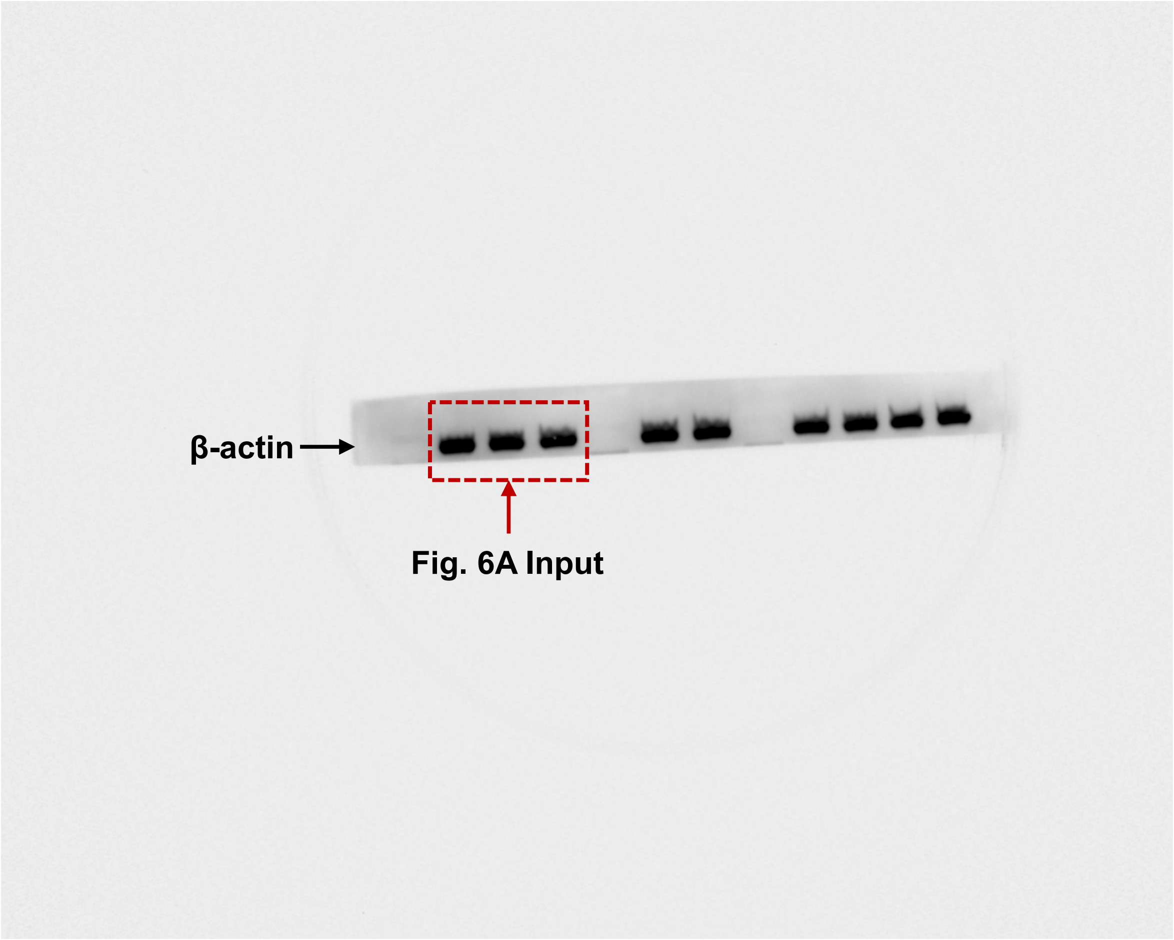

β-actin
Fig. 6A Input

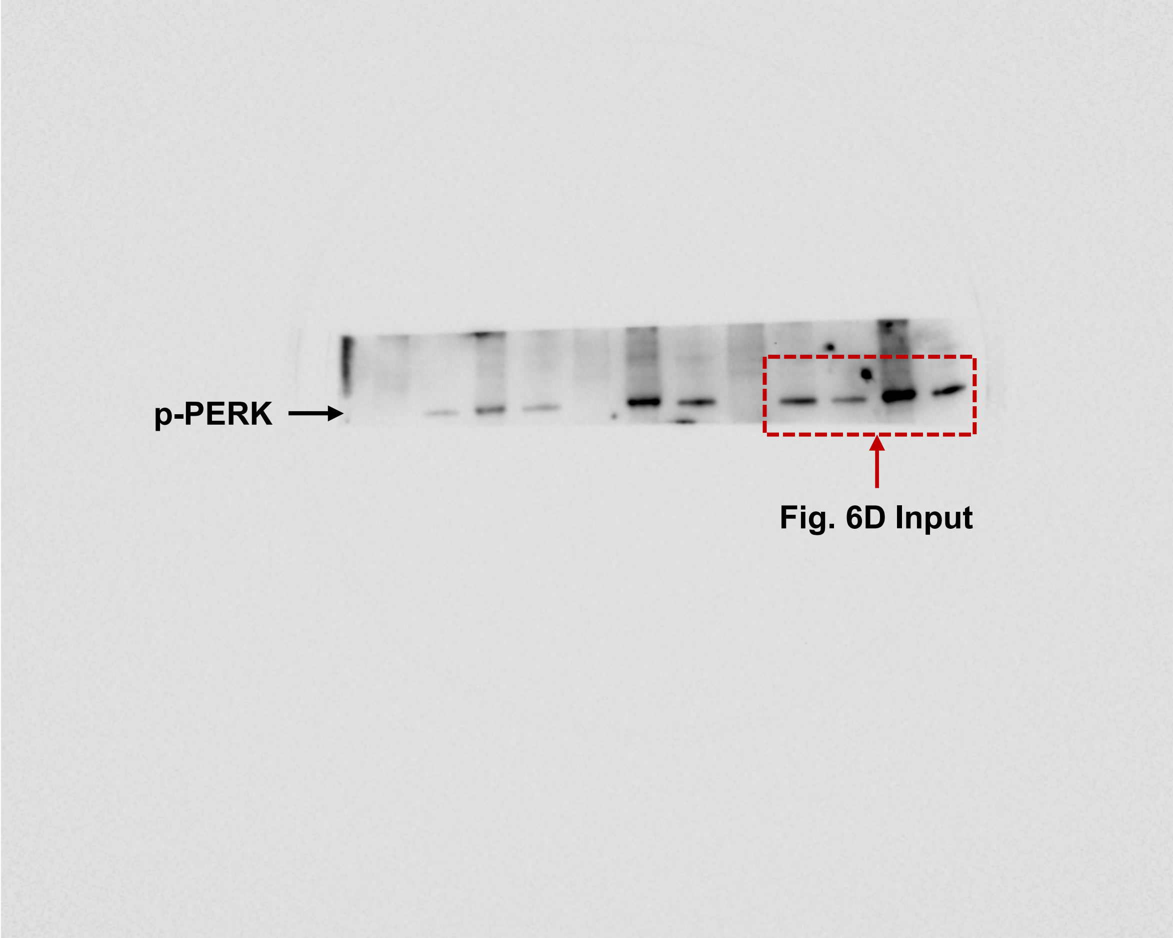

p-PERK
Fig. 6D Input

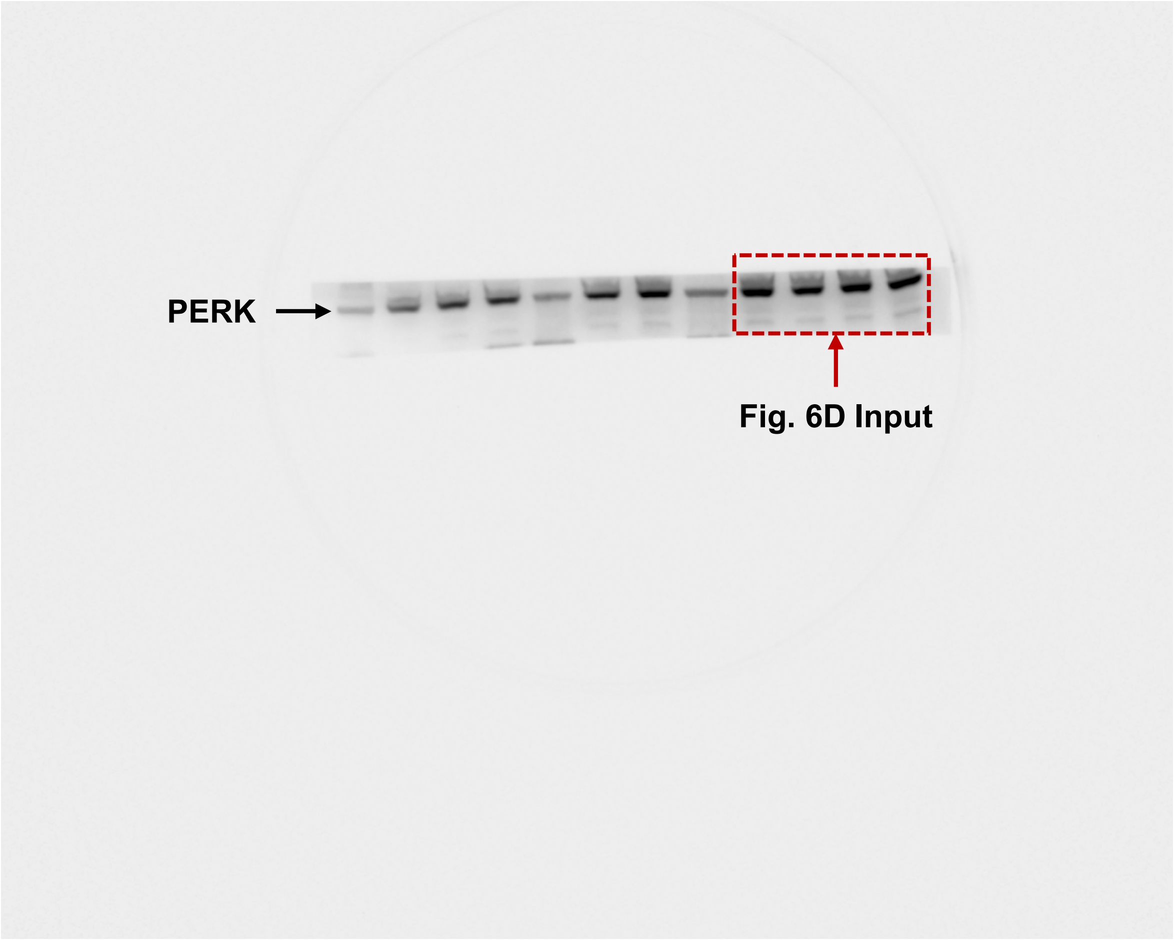
PERK
Fig. 6D Input

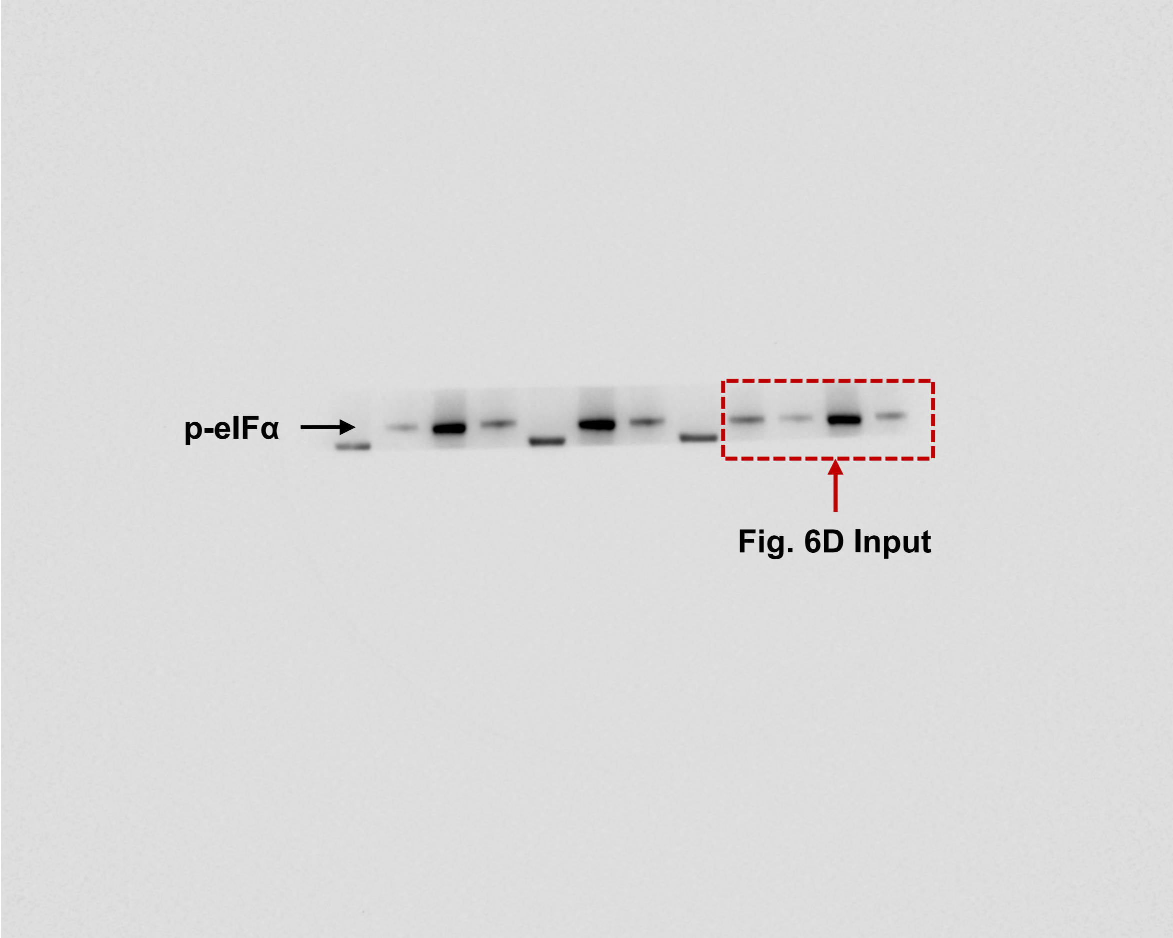
p-eIFα
Fig. 6D Input

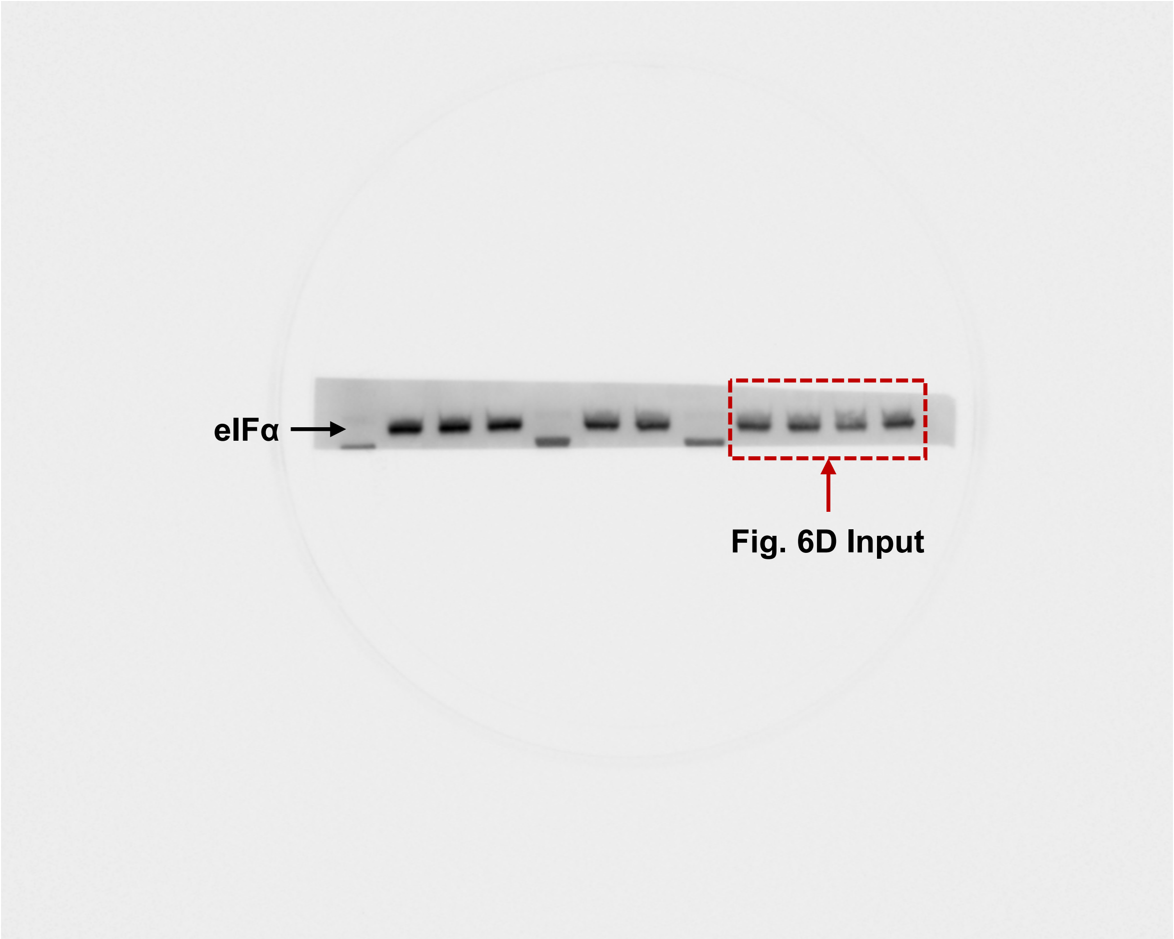
eIFα
Fig. 6D Input

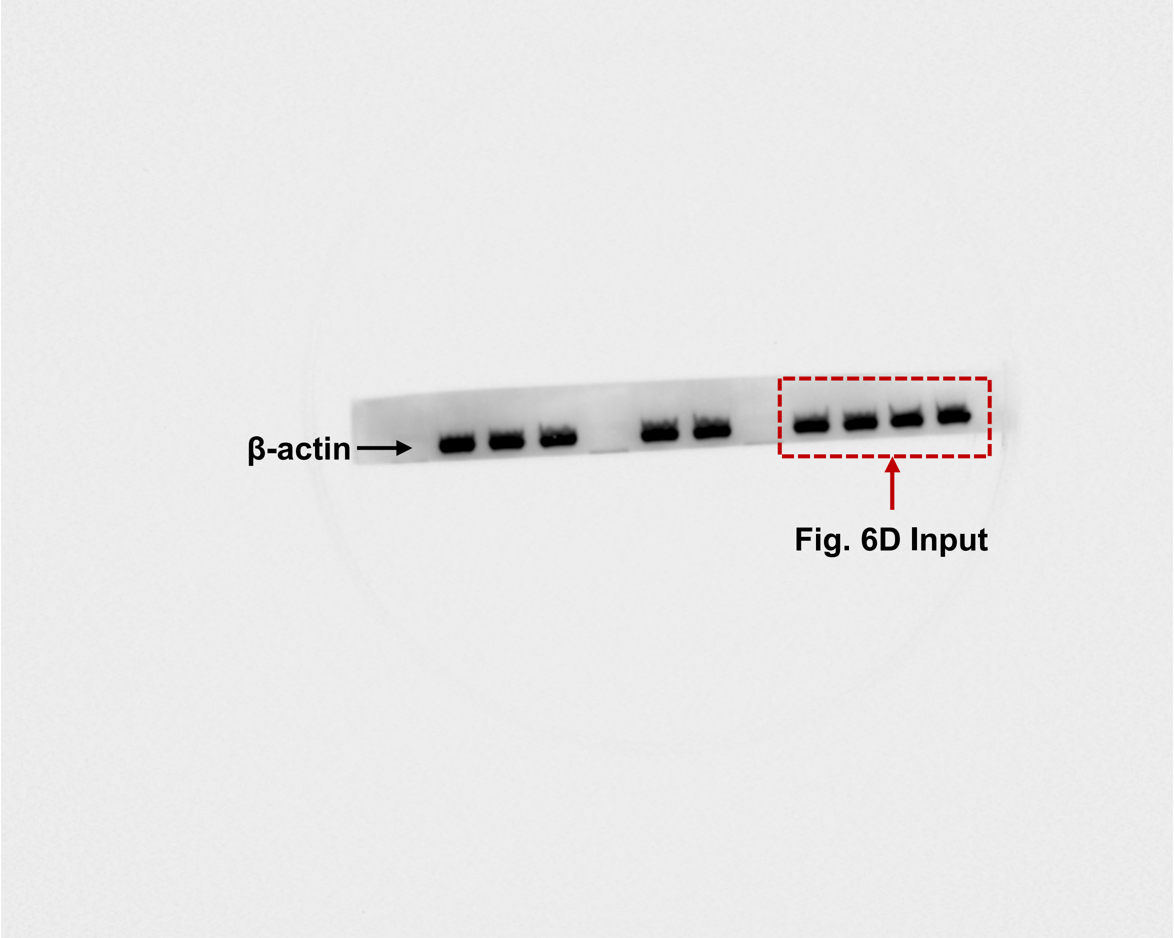
β-actin
Fig. 6D Input

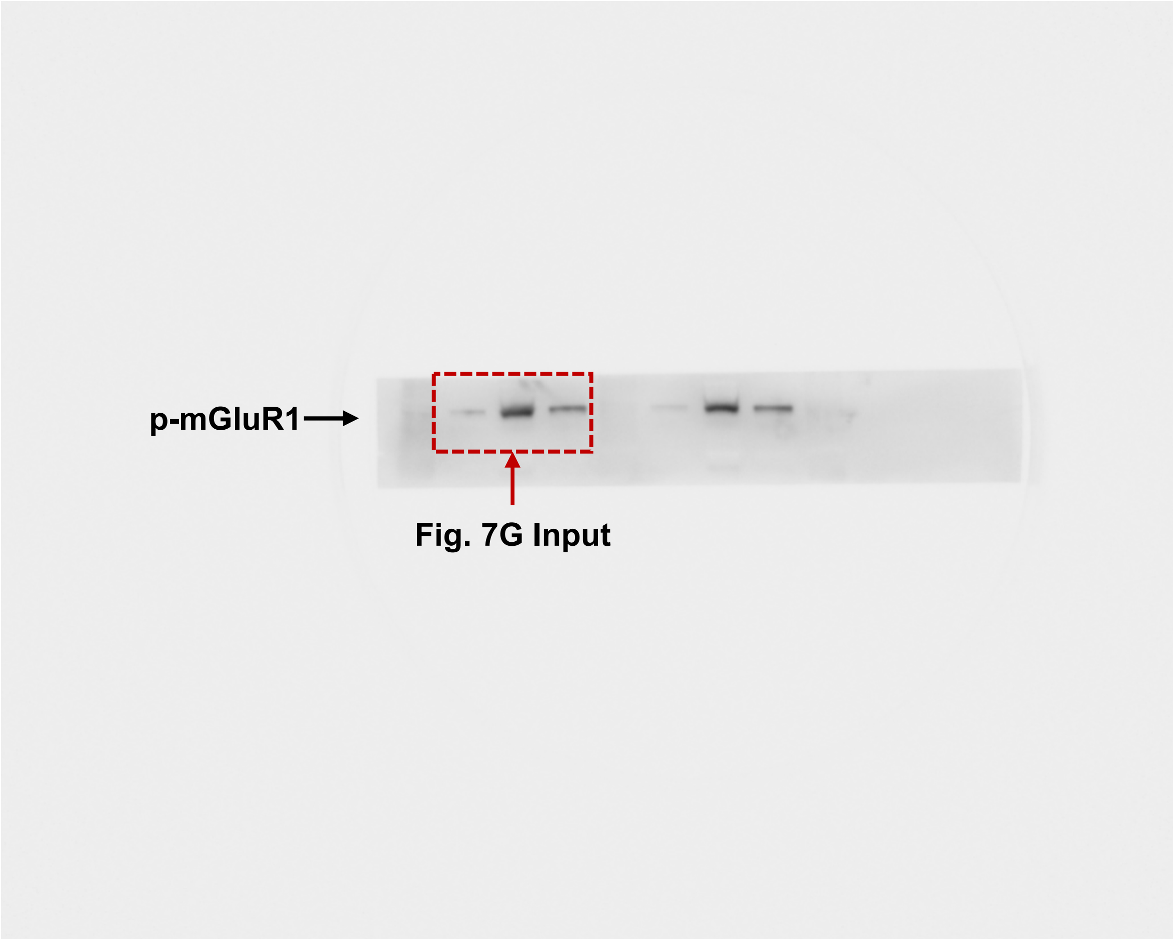
p-mGluR1
Fig. 7G Input

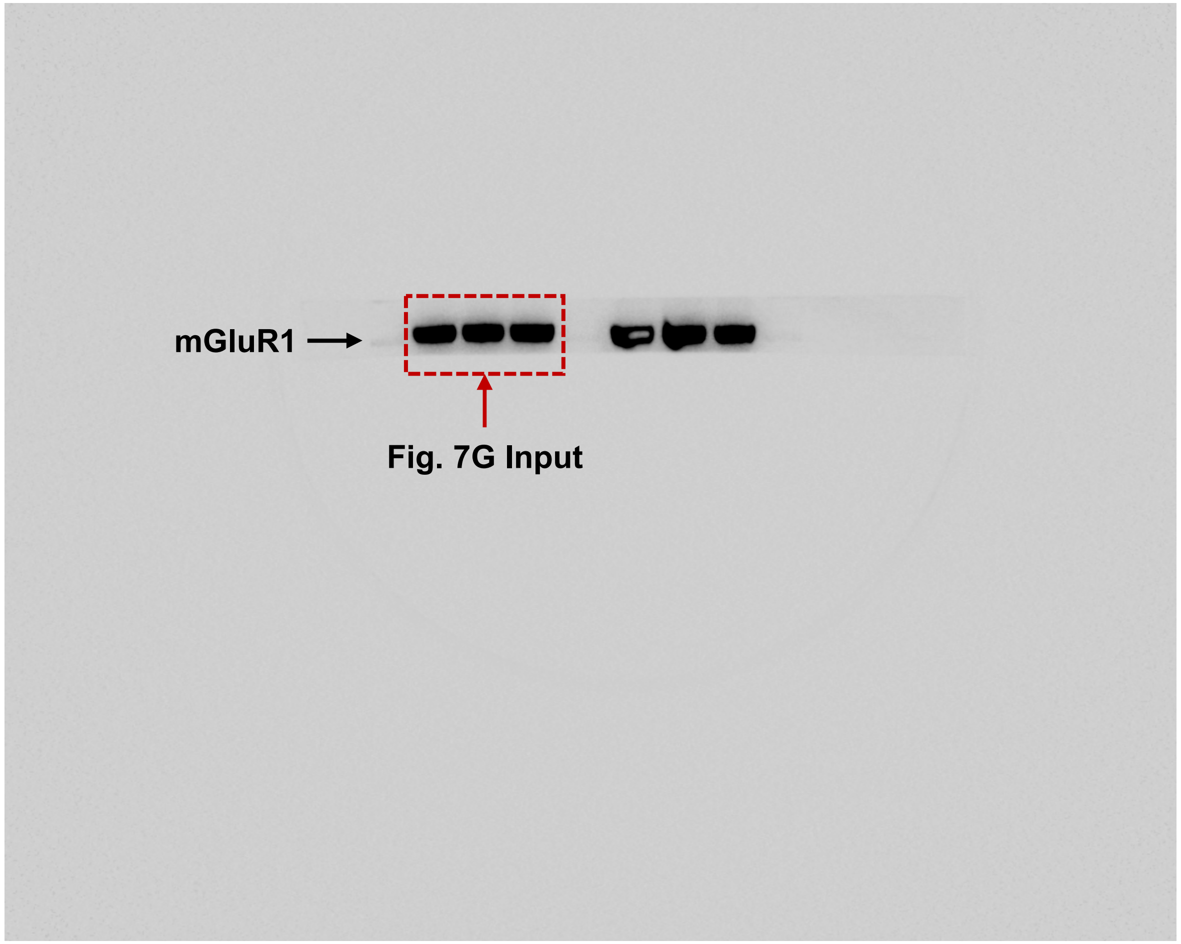
mGluR1
Fig. 7G Input

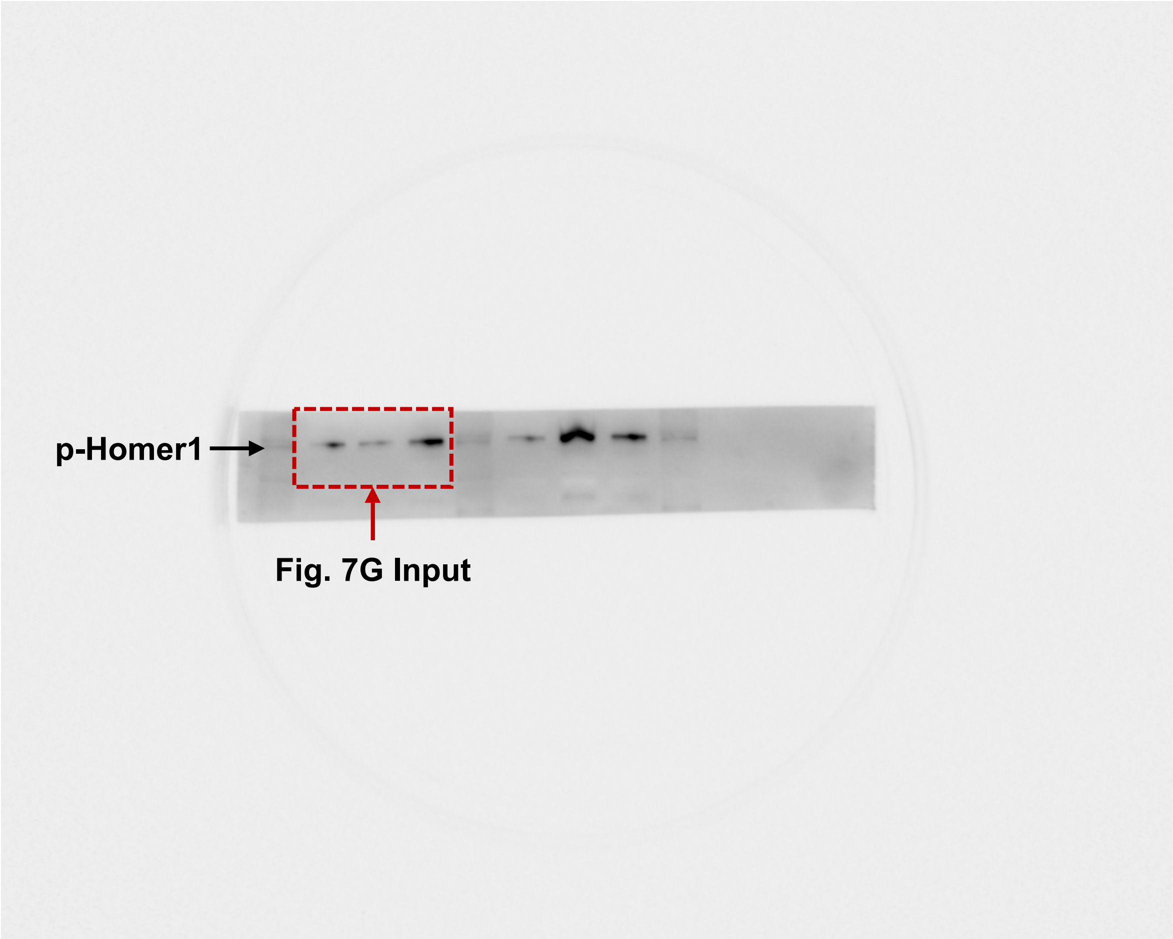
p-Homer1
Fig. 7G Input

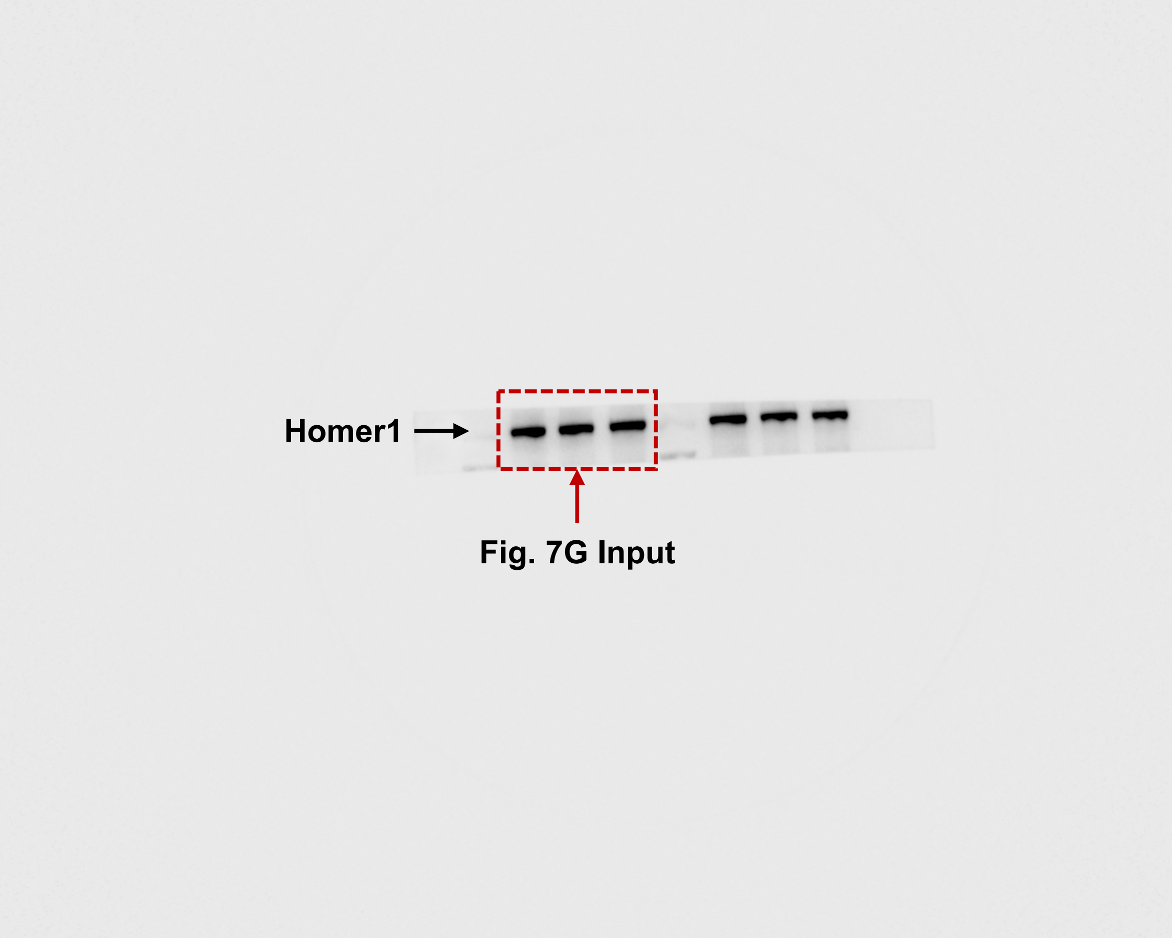
Homer1
Fig. 7G Input

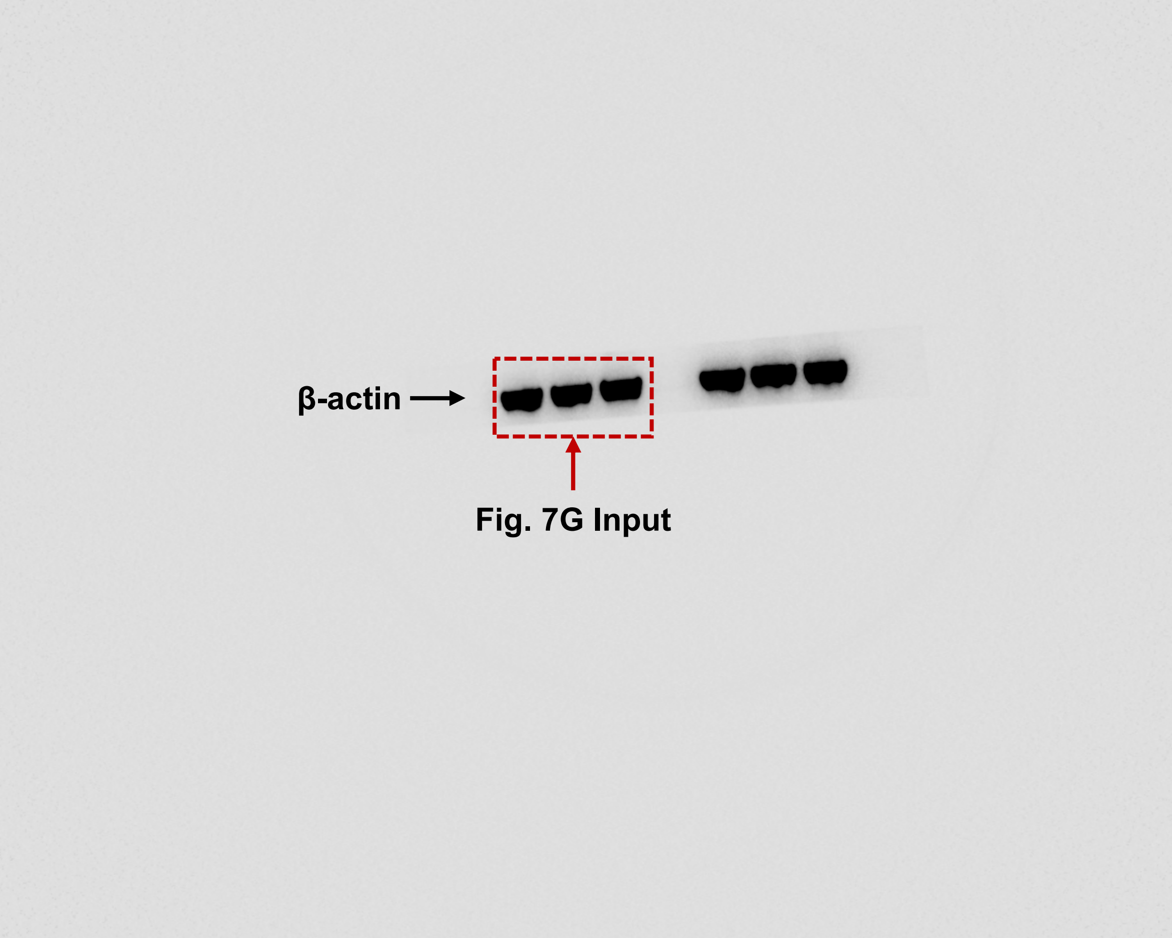
β-actin
Fig. 7G Input

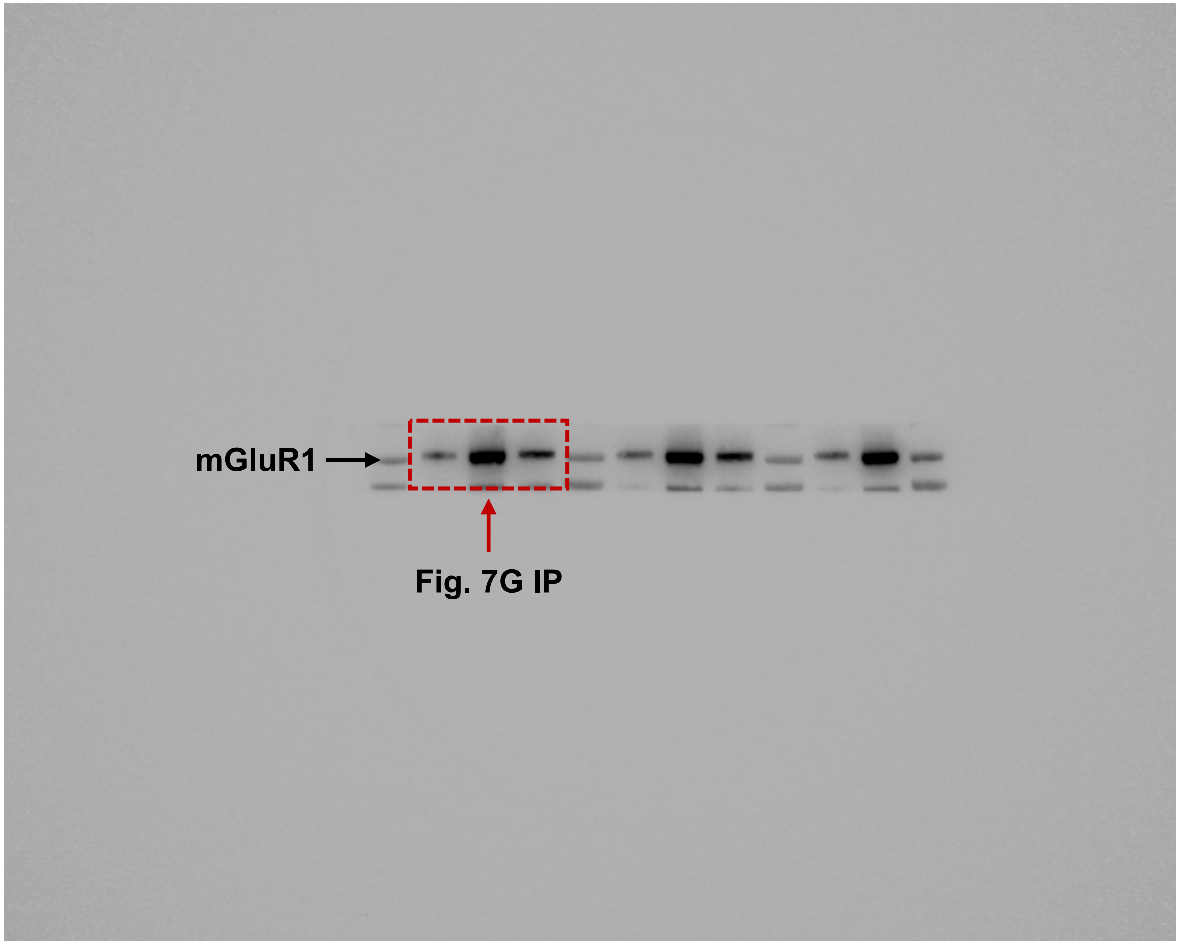
mGluR1
Fig. 7G IP

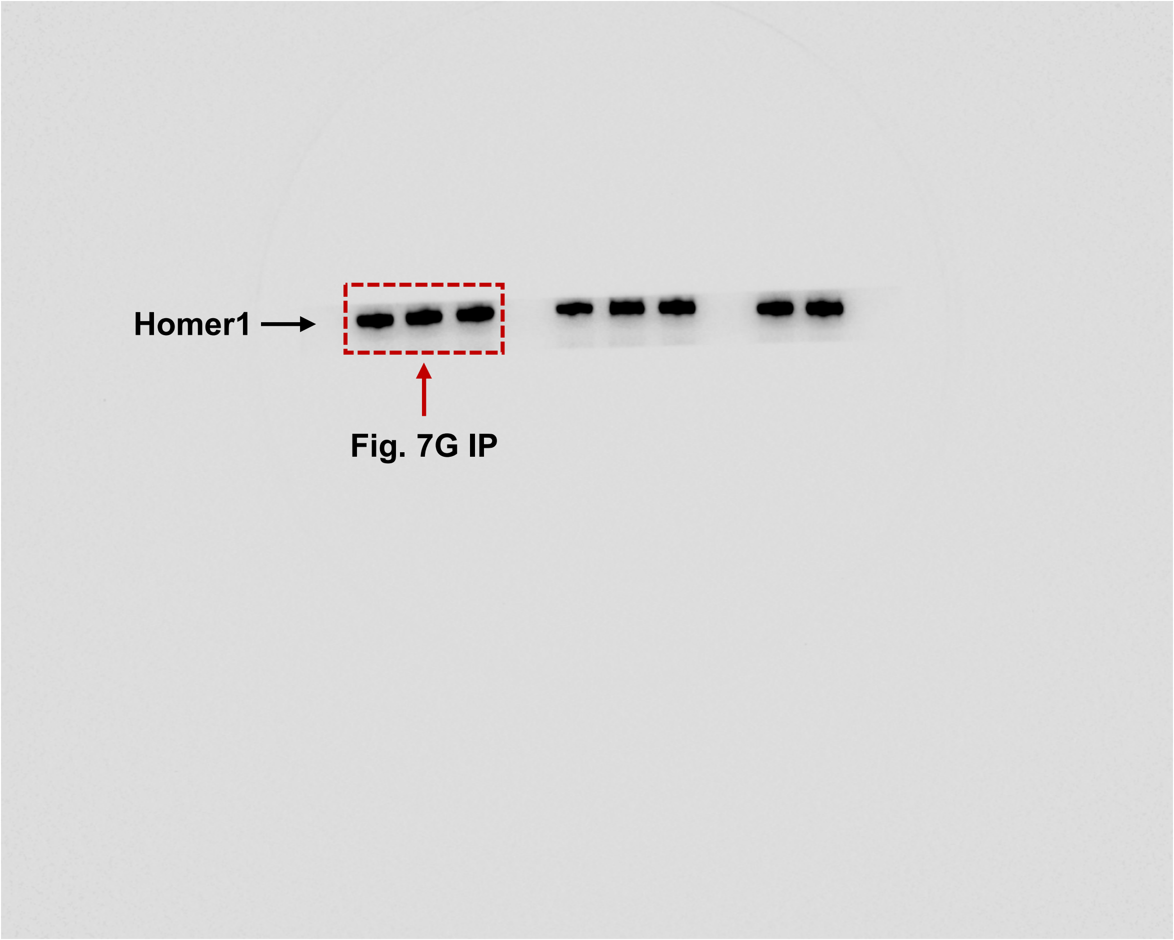

Homer1
Fig. 7G IP

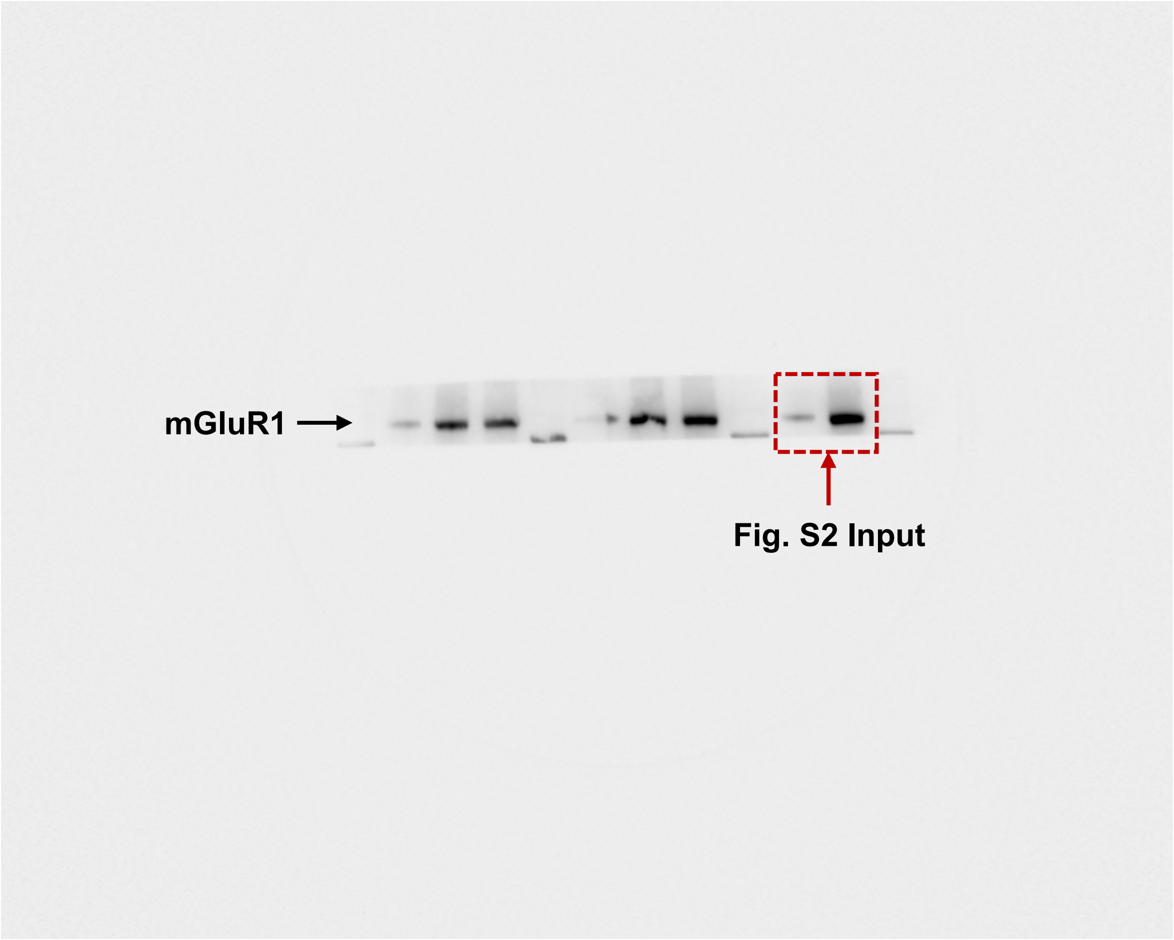
mGluR1
Fig. S2 Input

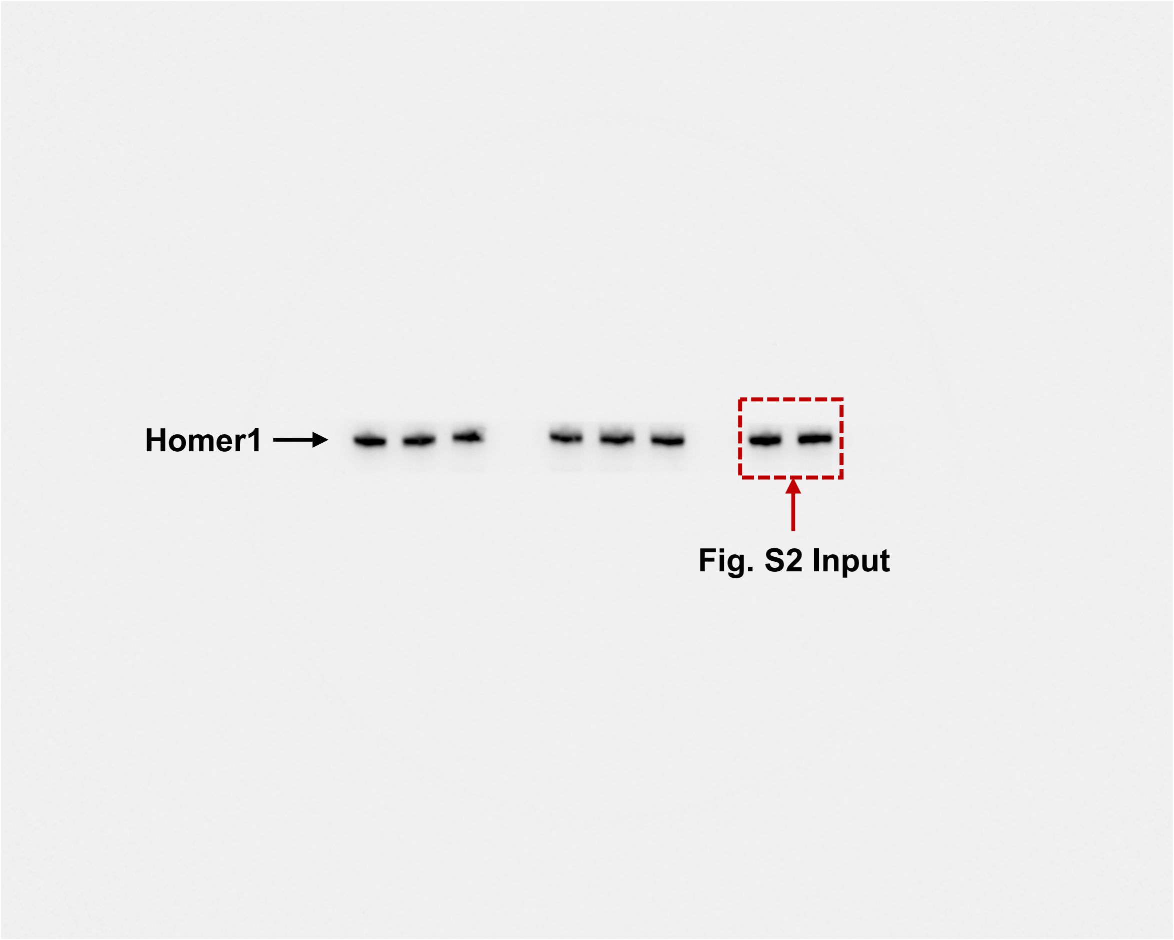

Homer1
Fig. S2 Input

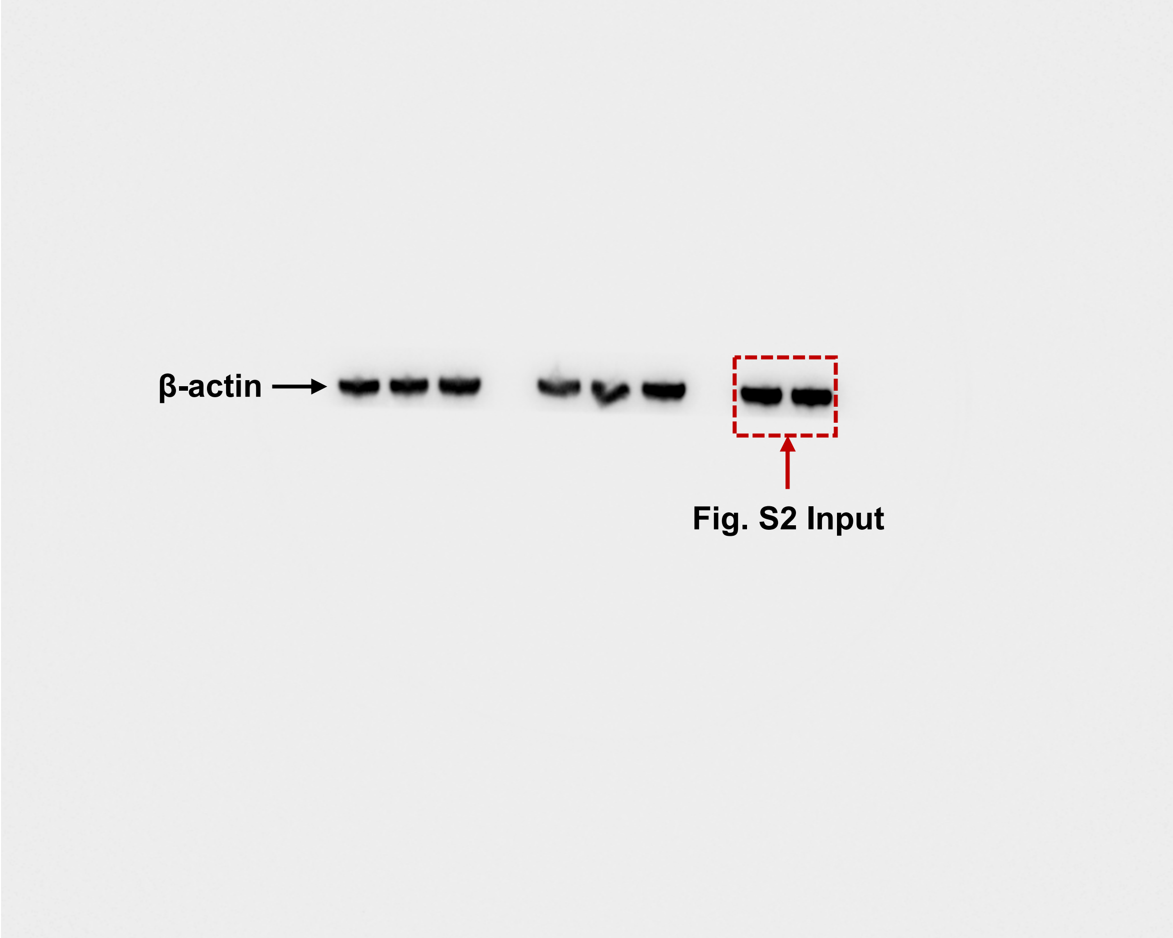
β-actin
Fig. S2 Input

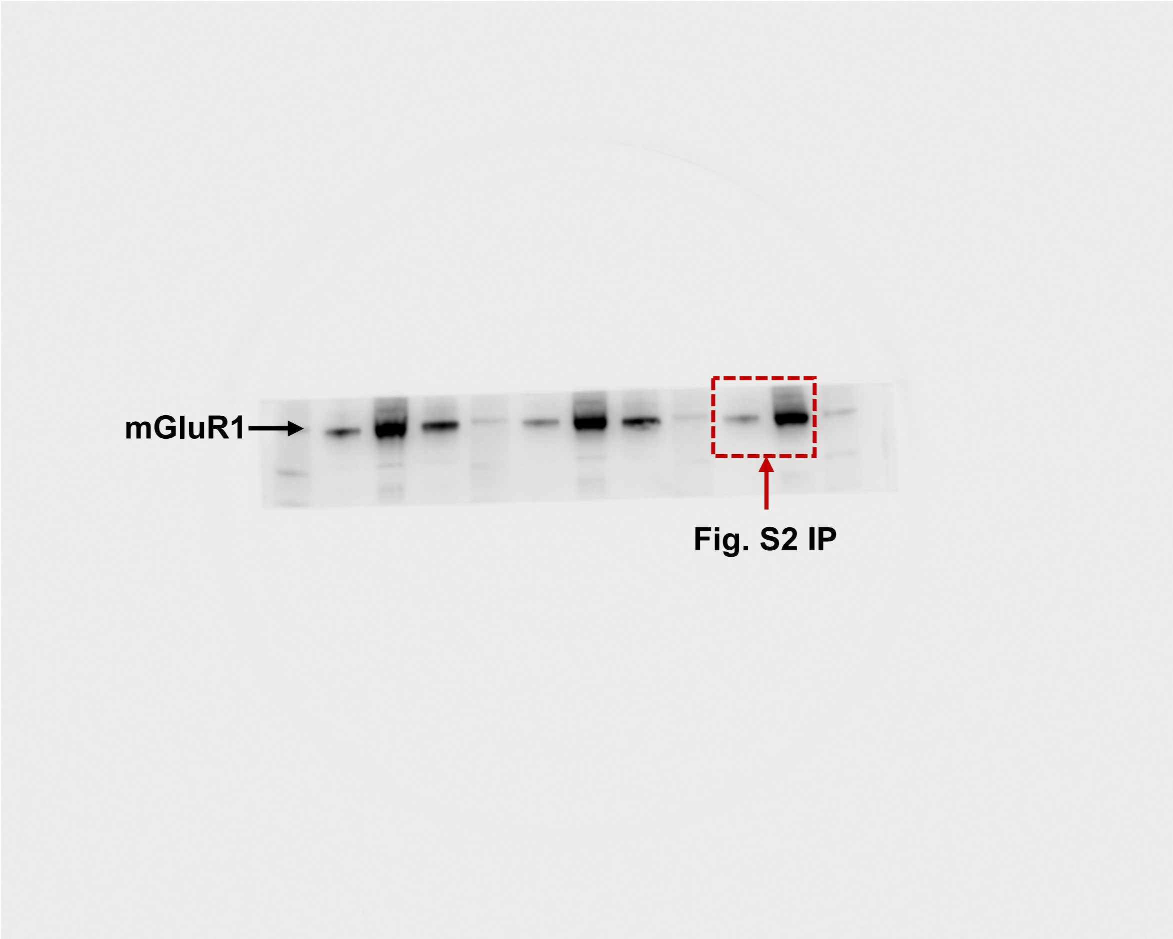
mGluR1
Fig. S2 IP

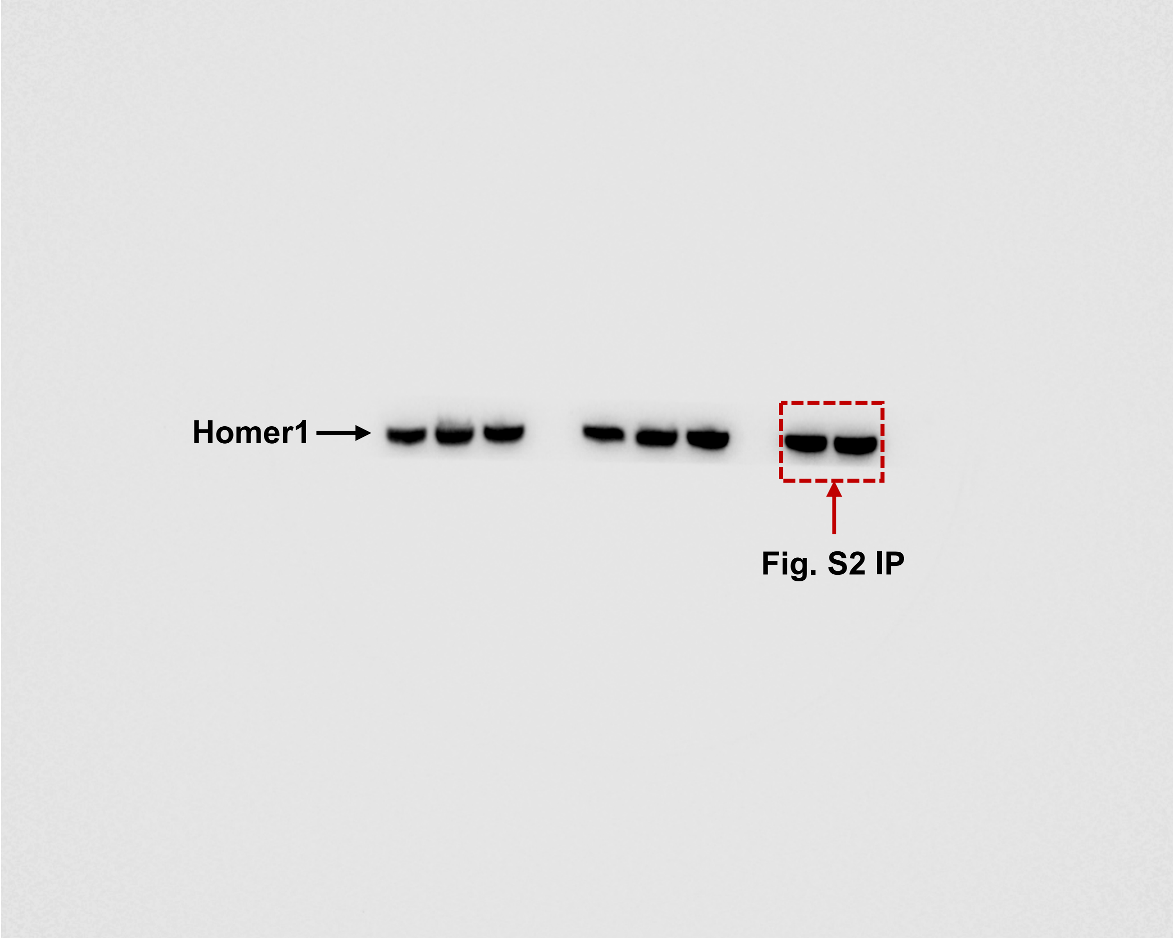
Homer1
Fig. S2 IP

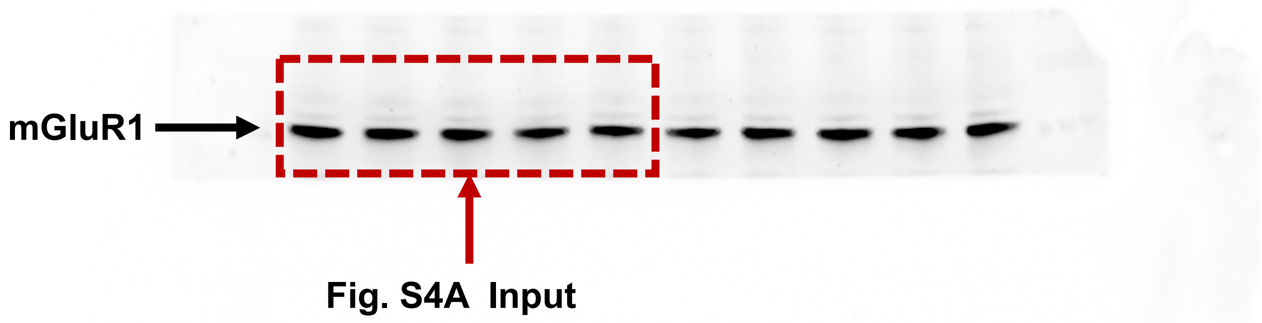
mGluR1
Fig. S4A Input

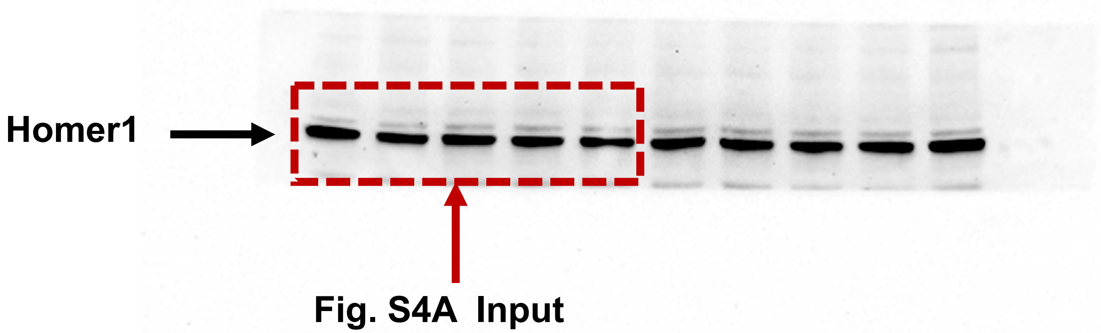
Homer1
Fig. S4A Input

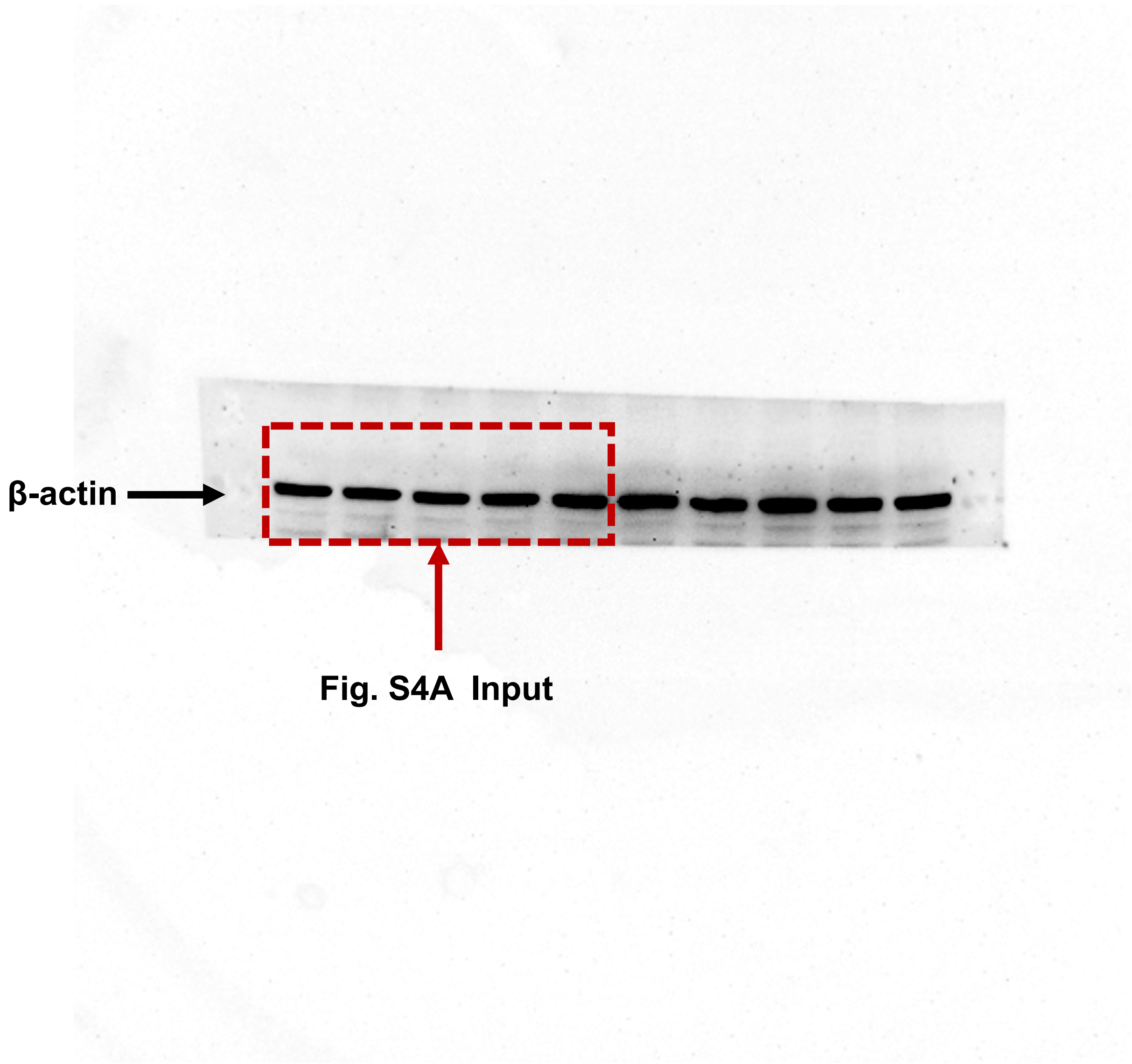
β-actin
Fig. S4A Input

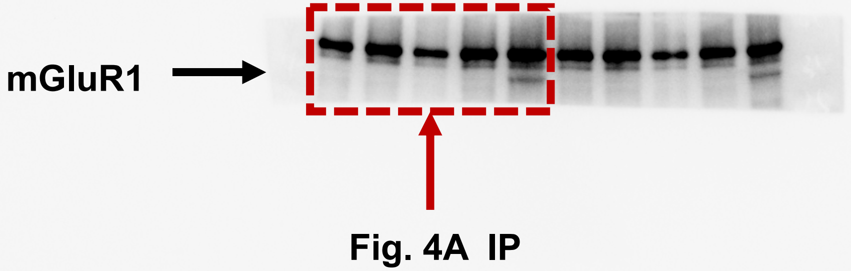

mGluR1
Fig. 4A IP

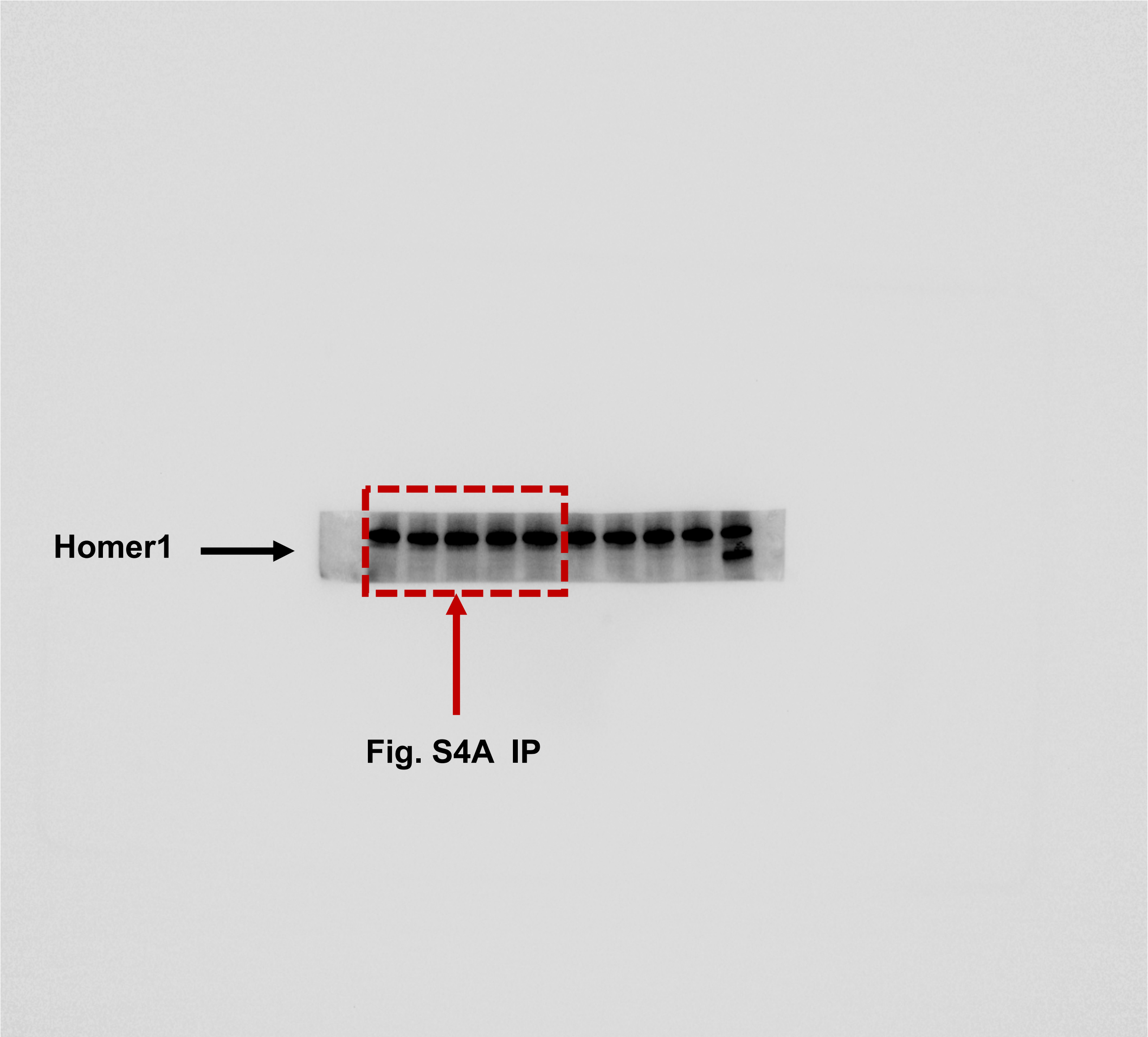
Homer1
Fig. S4A IP

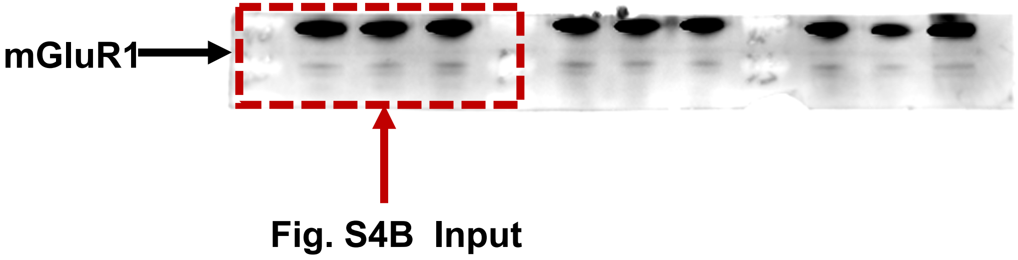
mGluR1
Fig. S4B Input

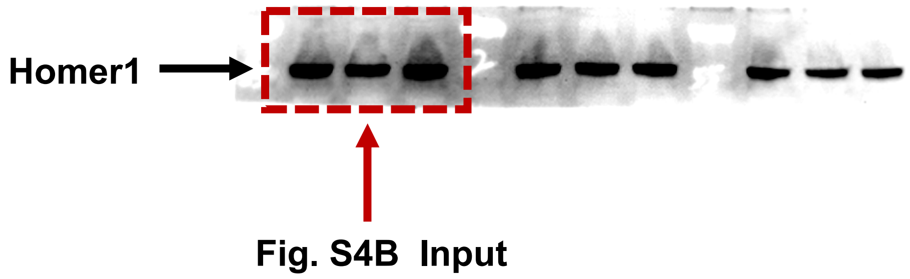
Homer1
Fig. S4B Input

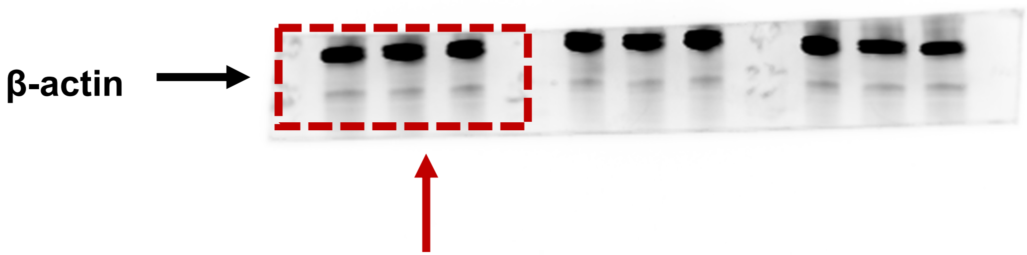

Fig. S4B Input

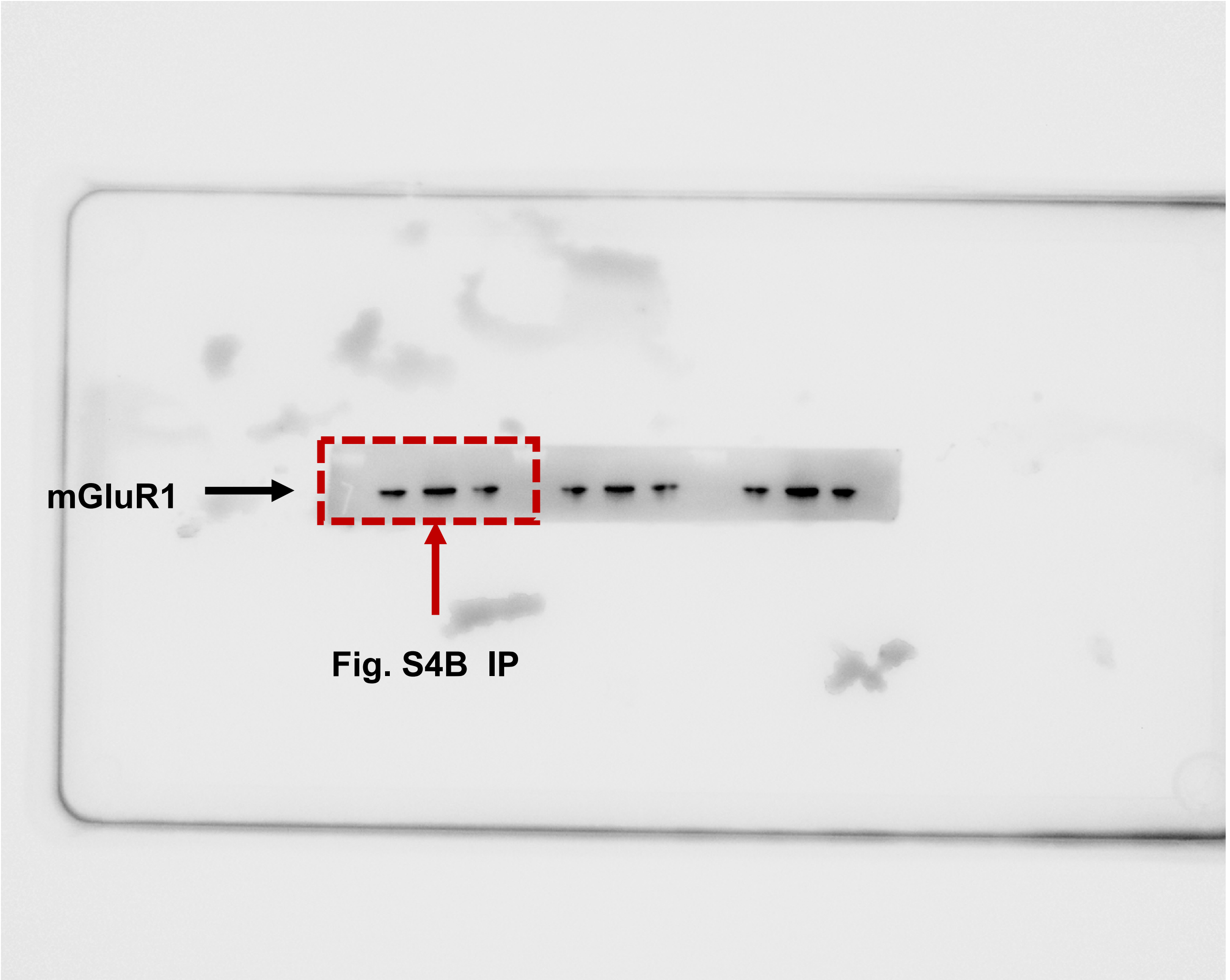
mGluR1
Fig. S4B IP

Homer1

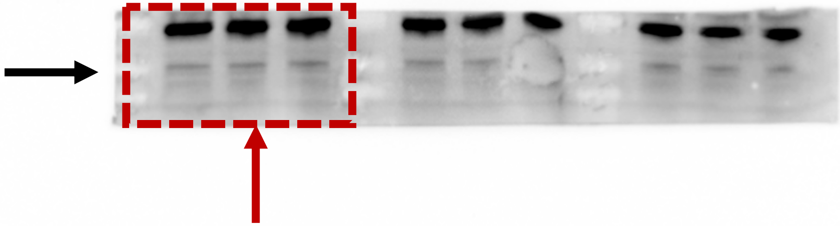

Fig. S4B IP

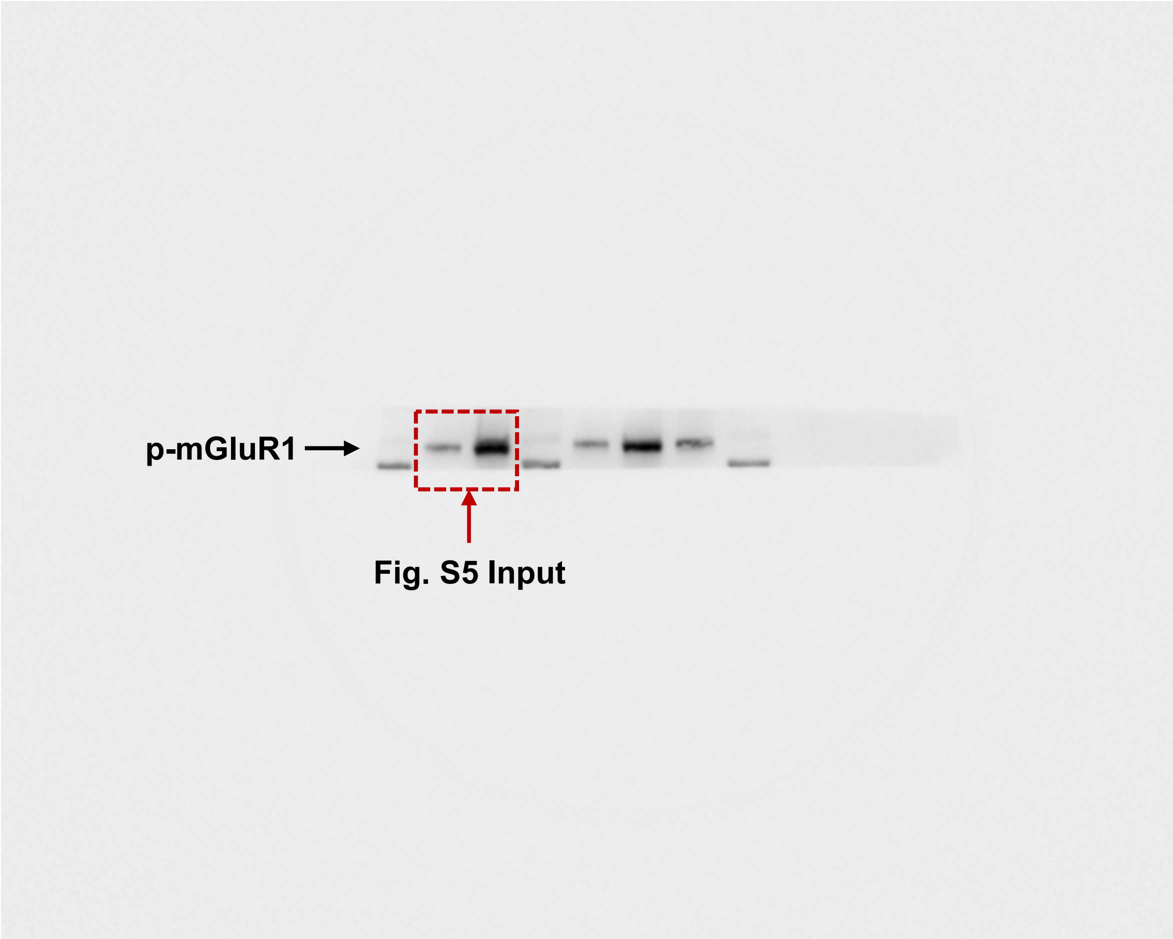
p-mGluR1
Fig. S5 Input

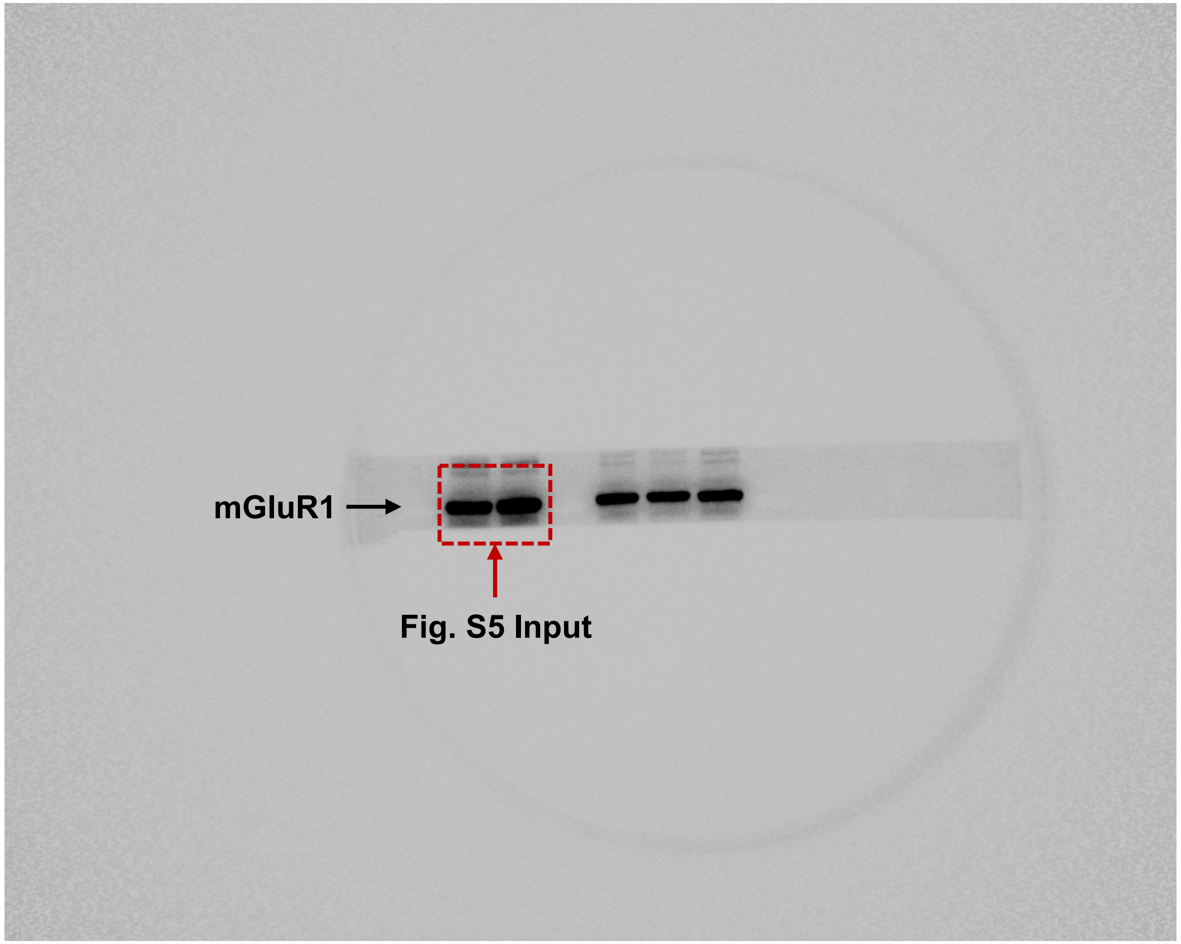

mGluR1
Fig. S5 Input

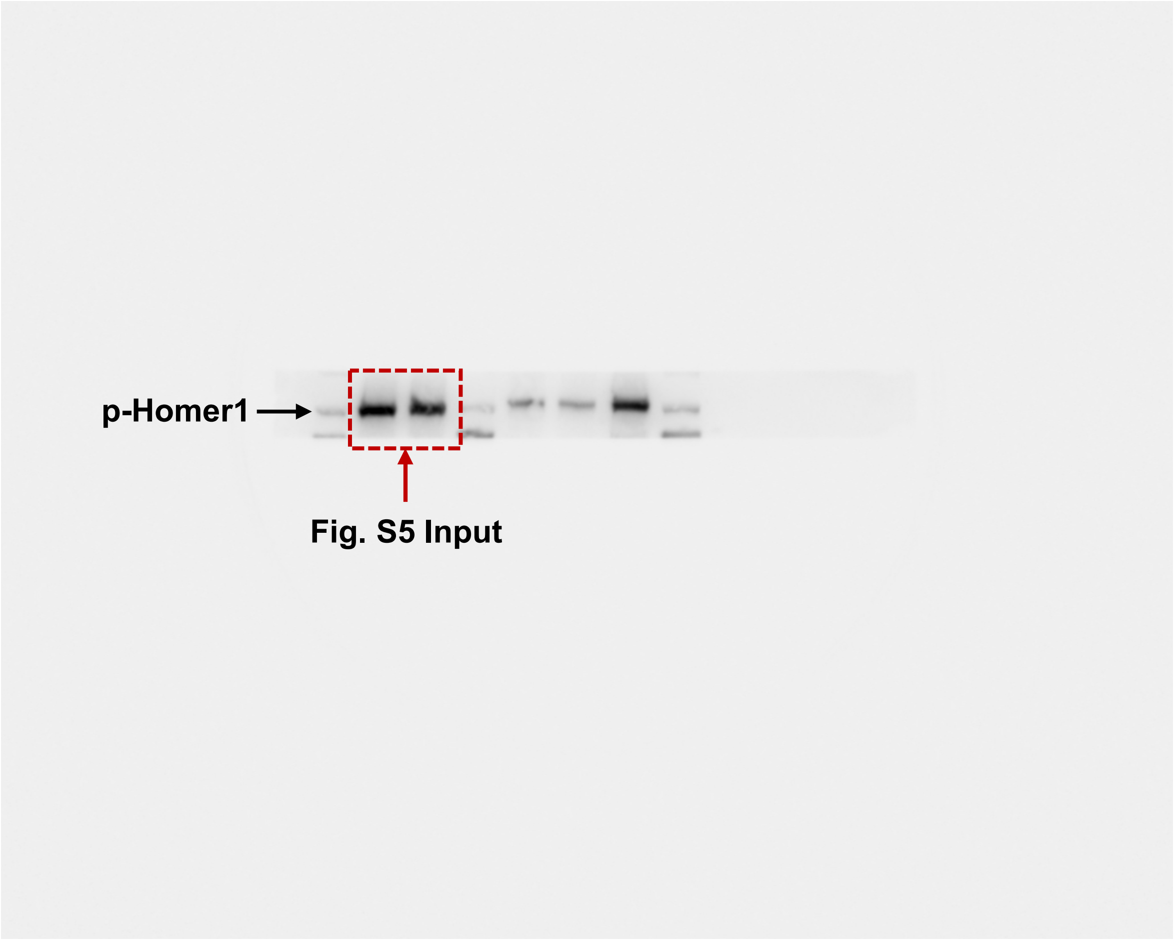
p-Homer1
Fig. S5 Input

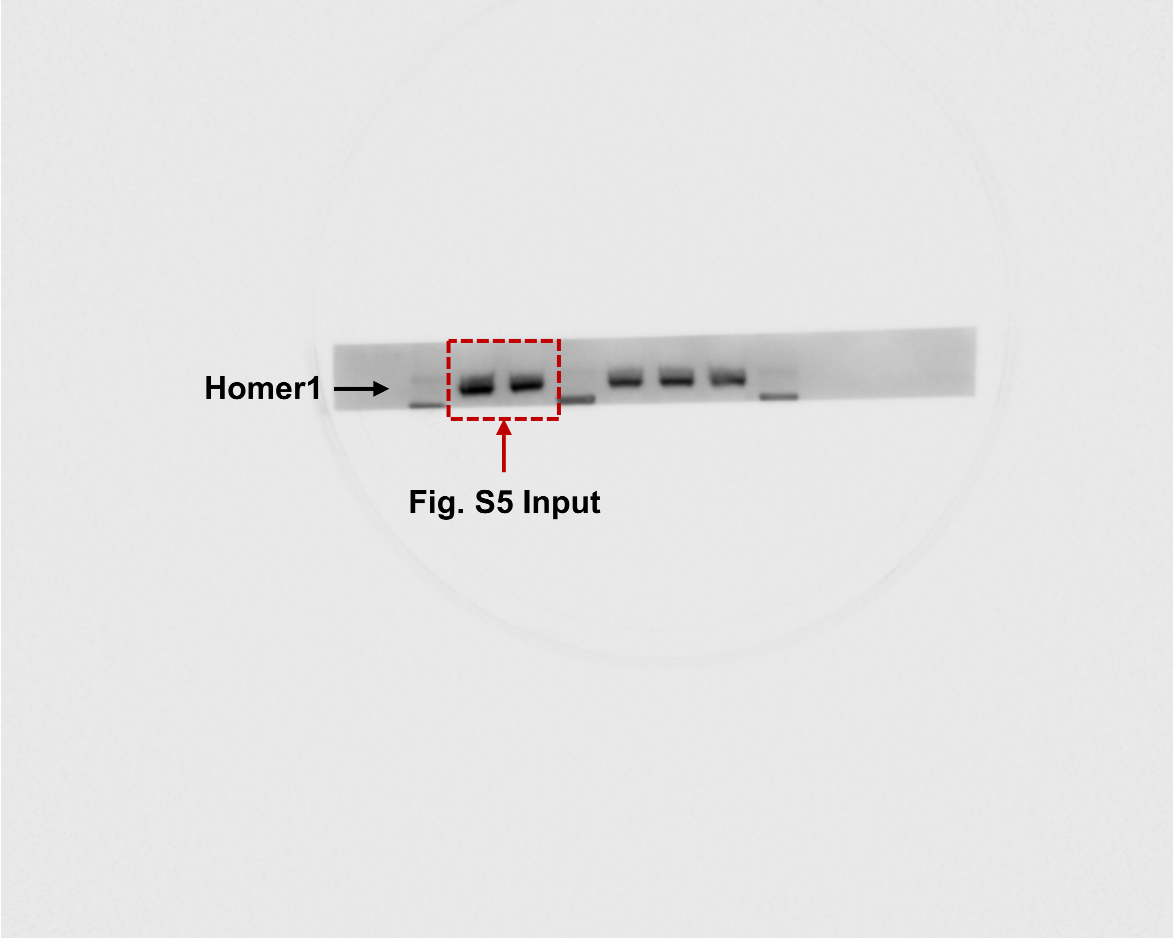
Homer1
Fig. S5 Input

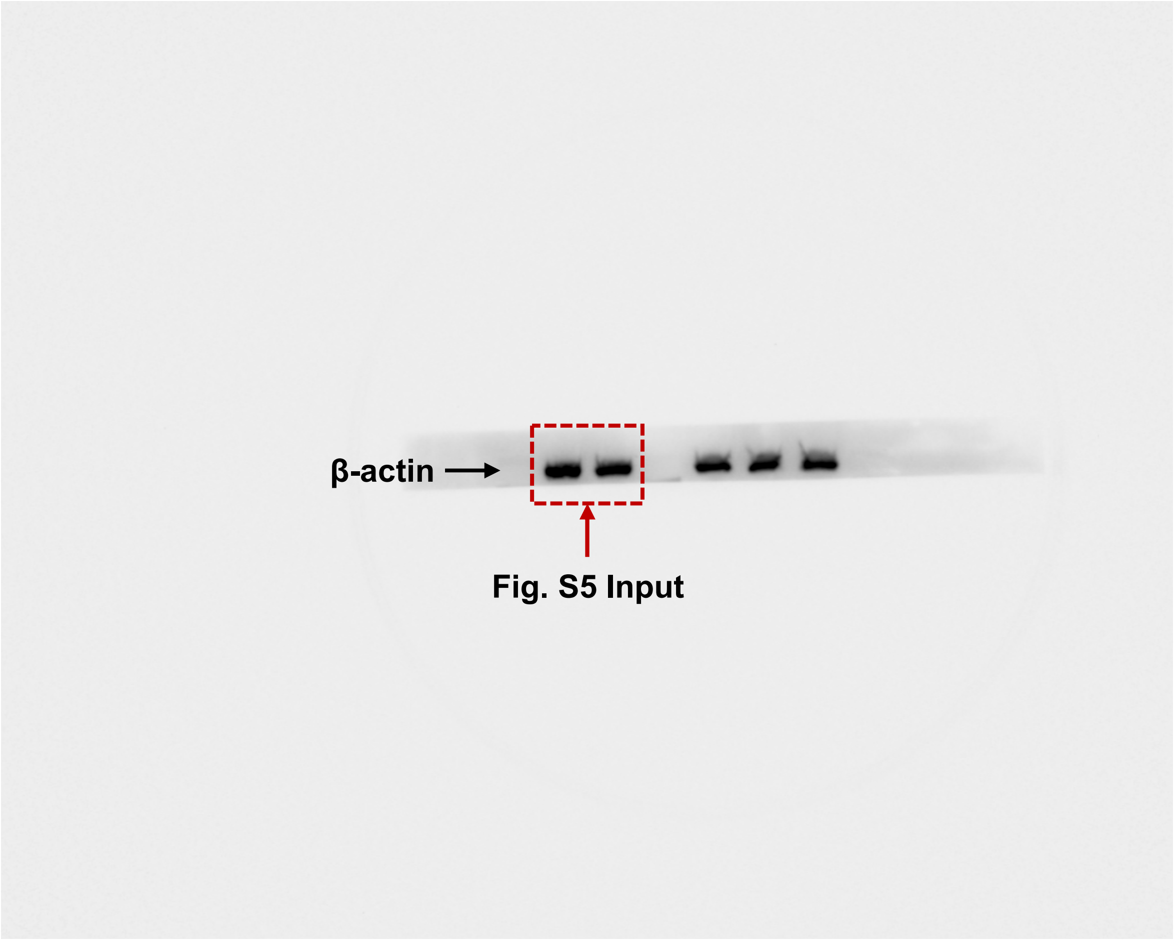
β-actin
Fig. S5 Input

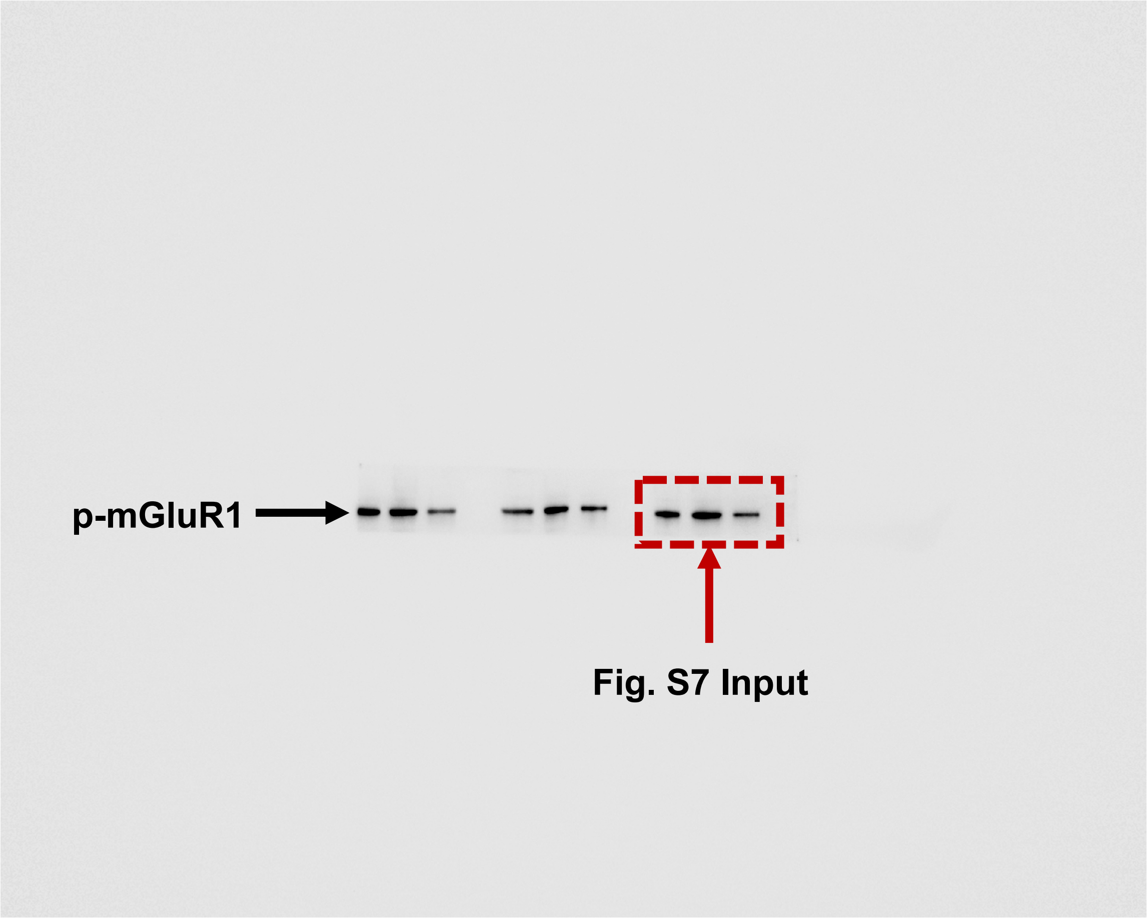
p-mGluR1
Fig. S7 Input

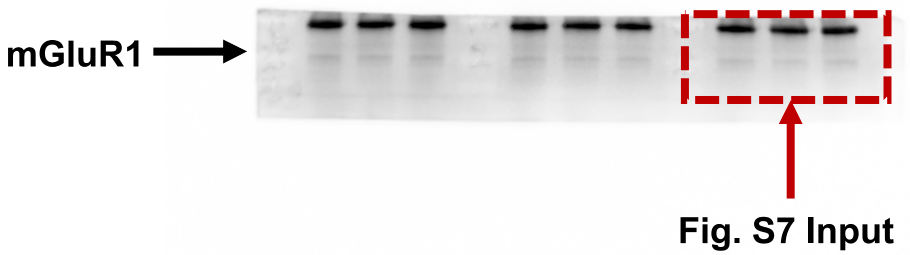
mGluR1
Fig. S7 Input

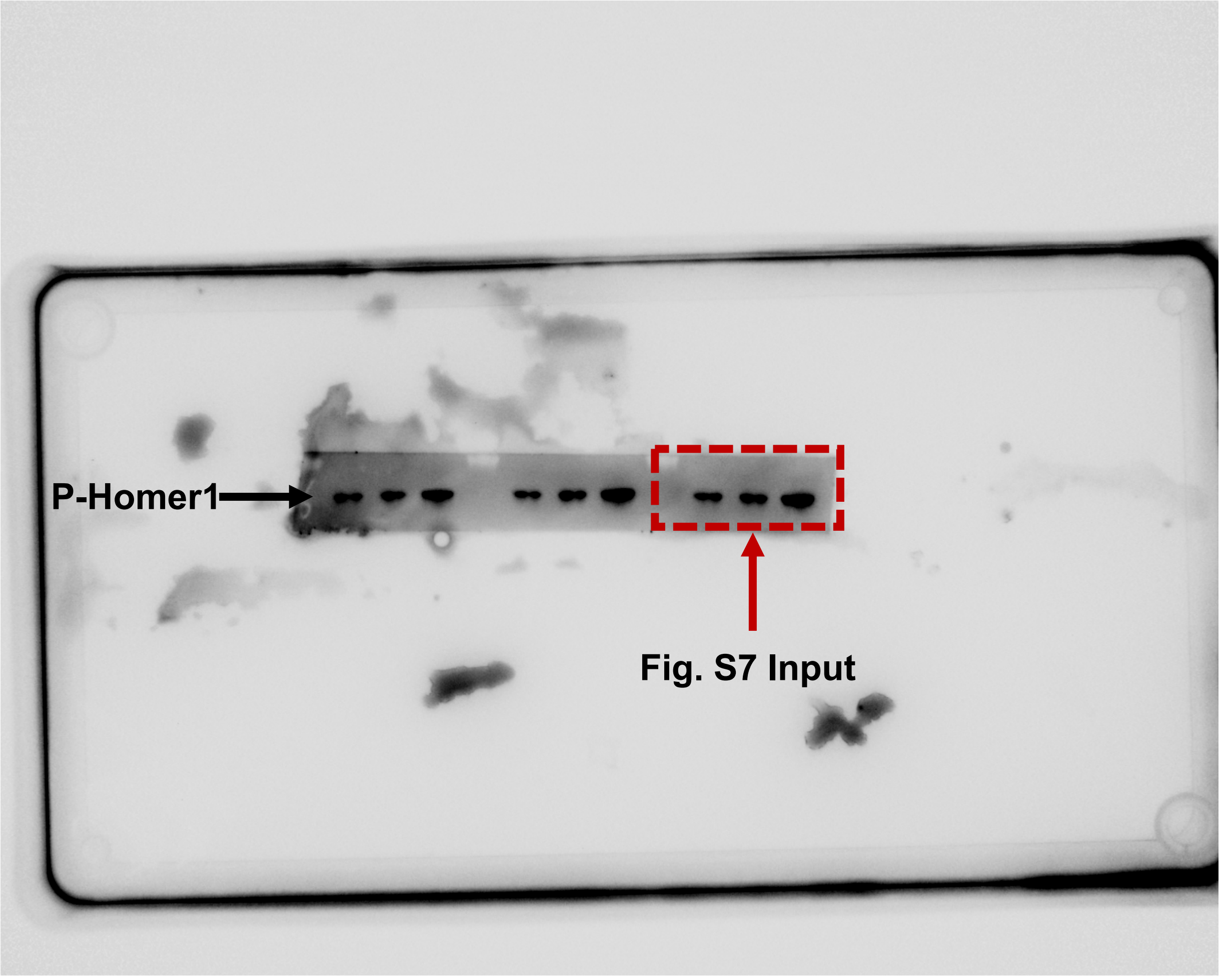
P-Homer1
Fig. S7 Input

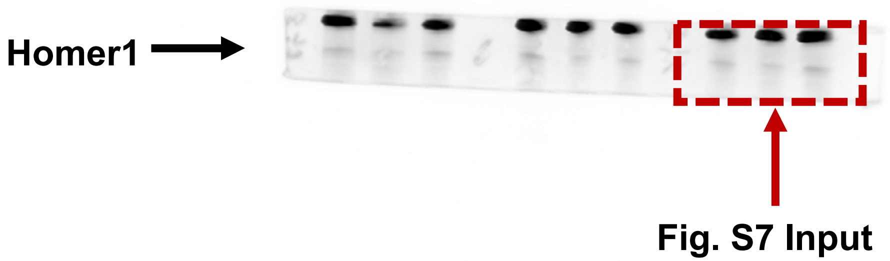

Homer1
Fig. S7 Input

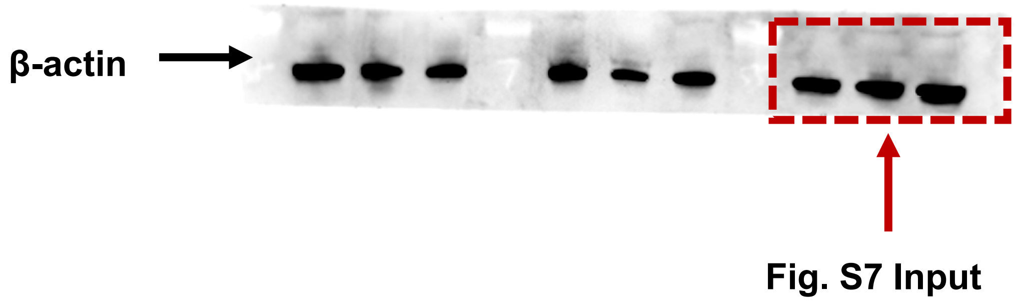
β-actin
Fig. S7 Input

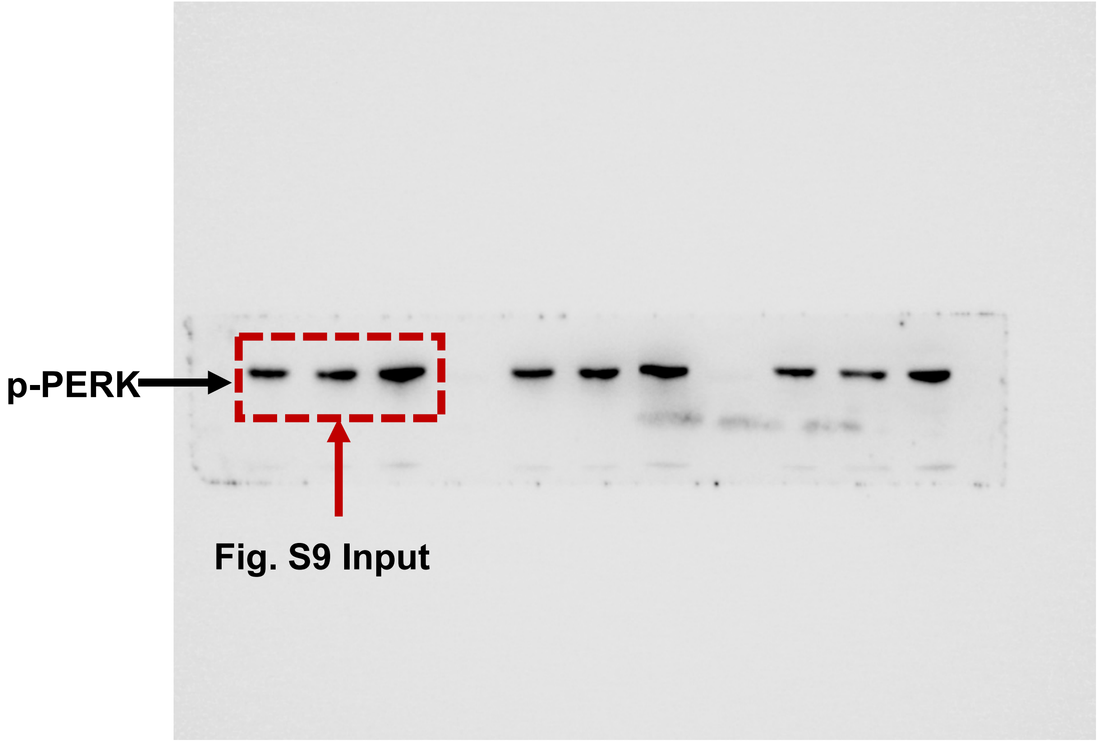
p-PERK
Fig. S9 Input

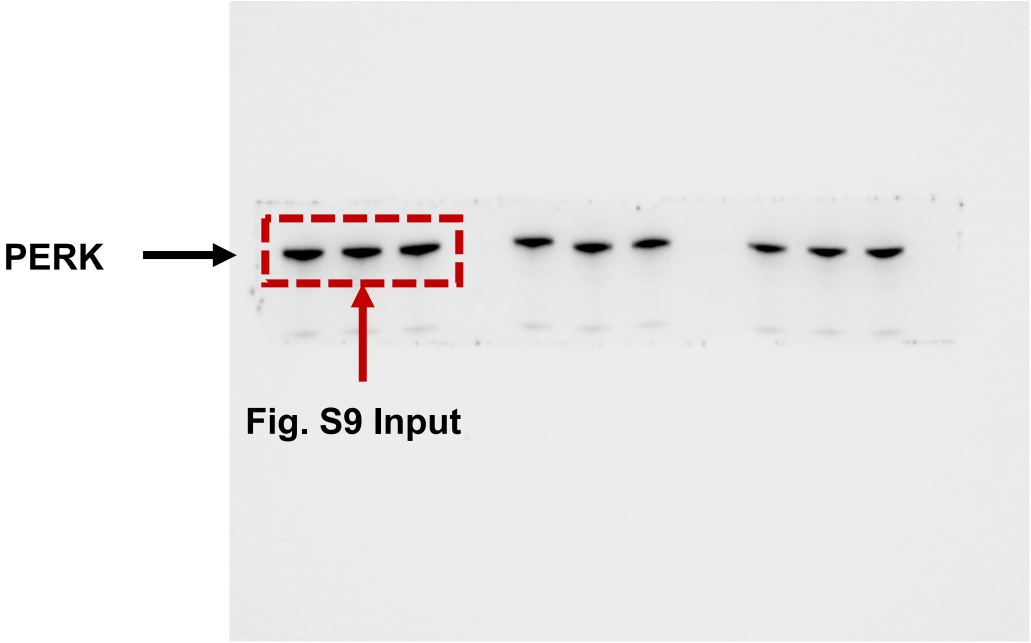
PERK
Fig. S9 Input

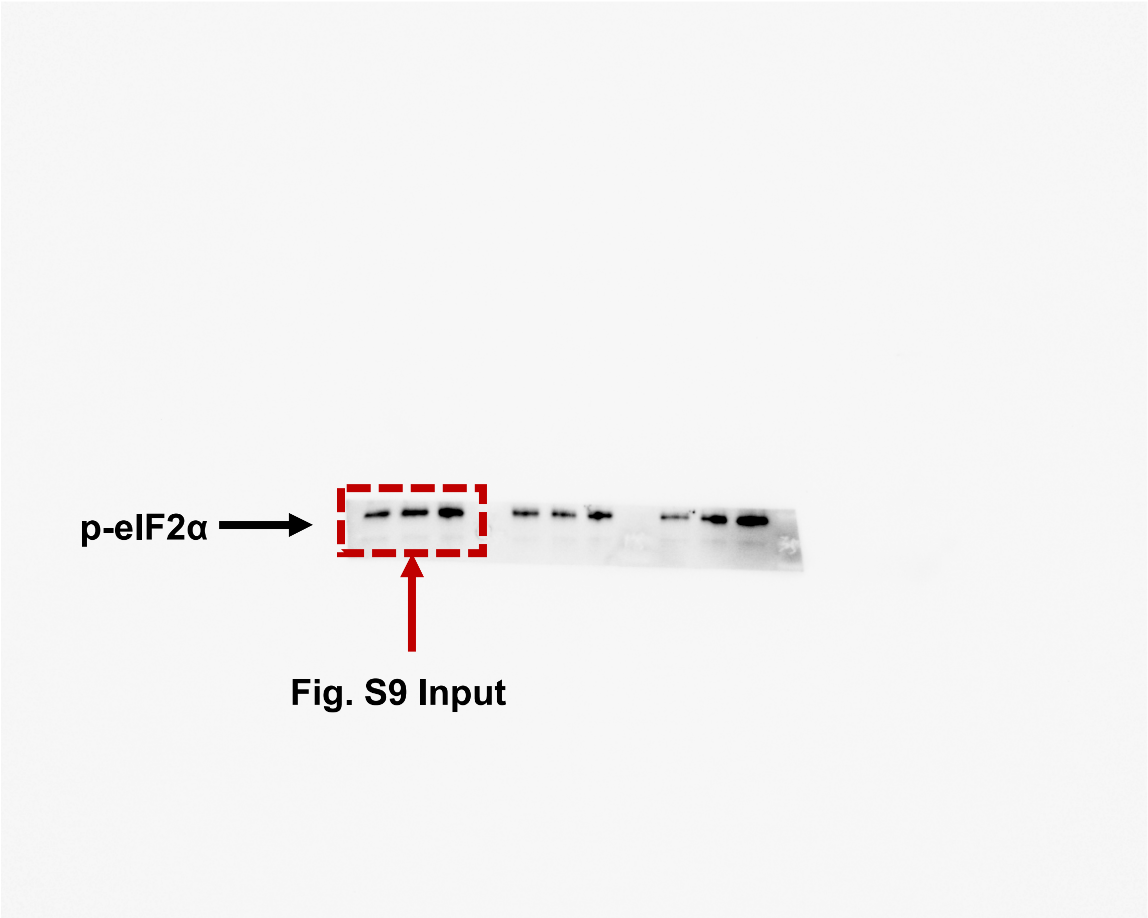
p-eIF2α
Fig. S9 Input

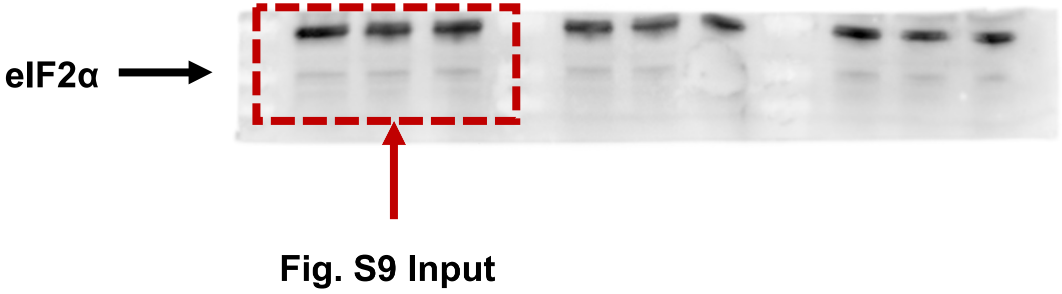
eIF2α
Fig. S9 Input

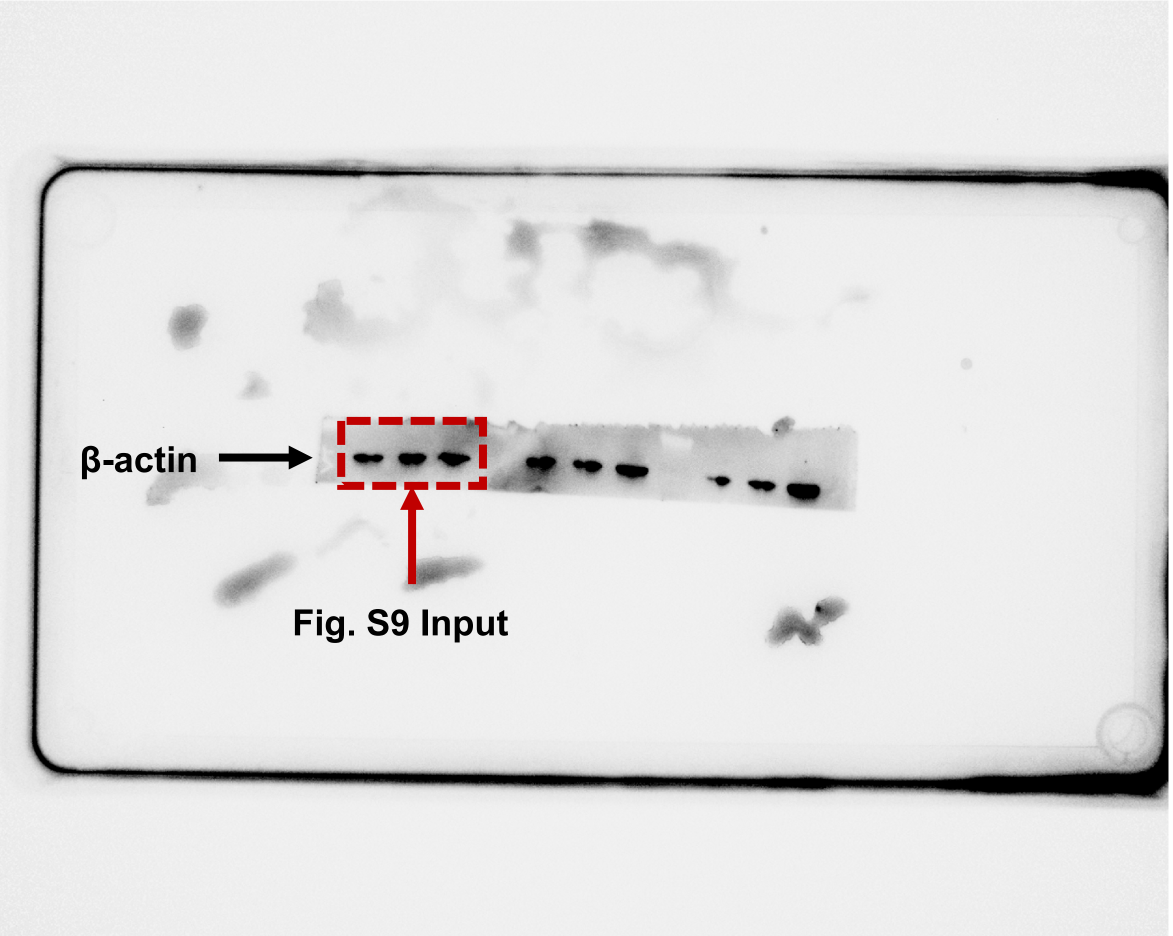
β-actin
Fig. S9 Input

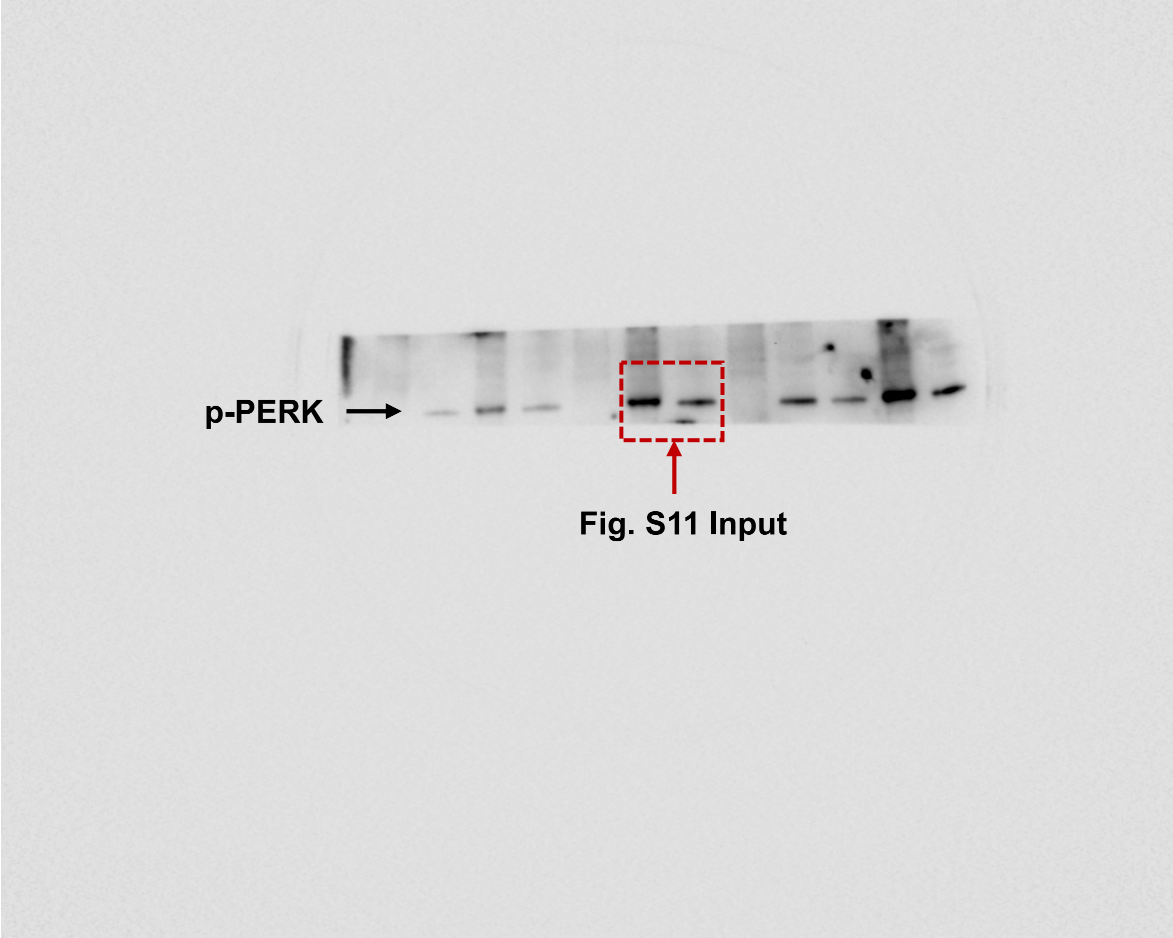
p-PERK
Fig. S11 Input

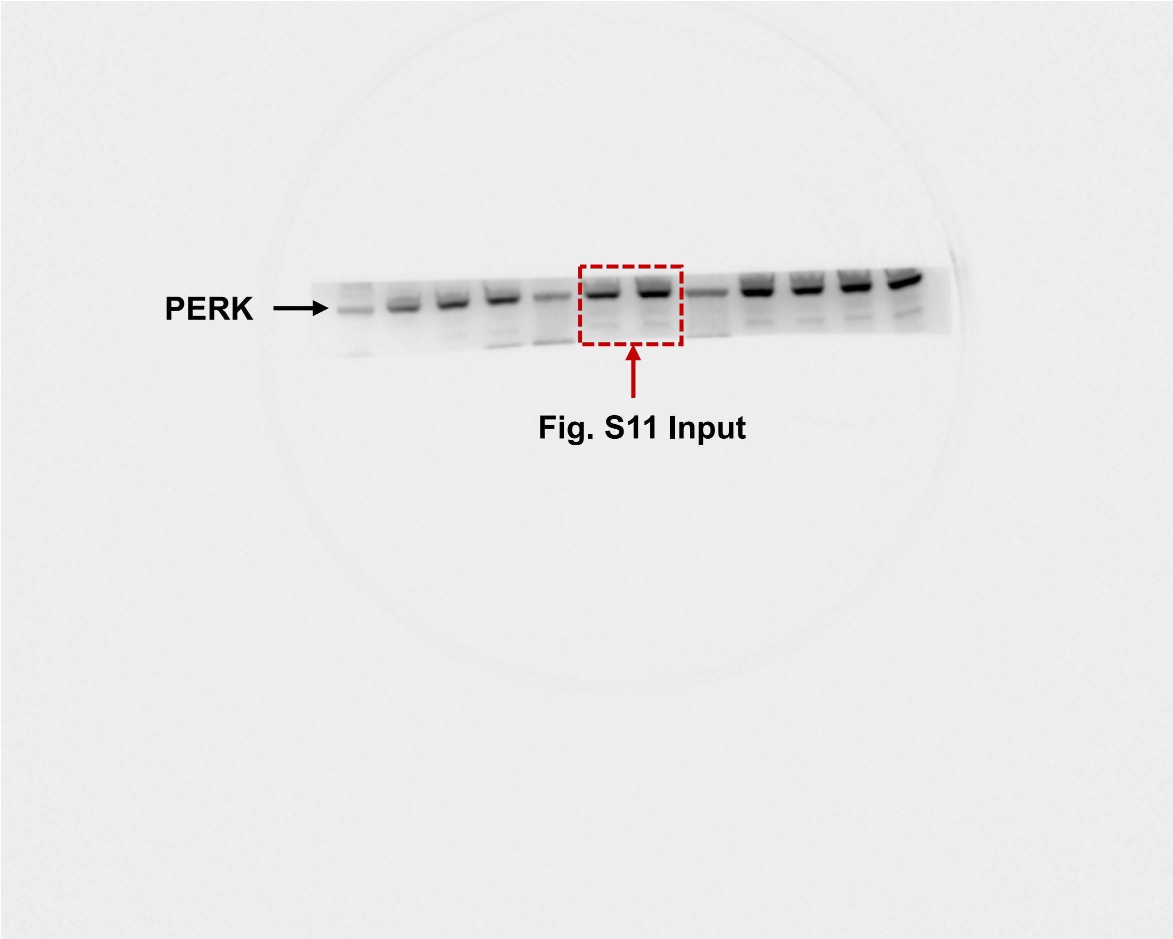
PERK
Fig. S11 Input

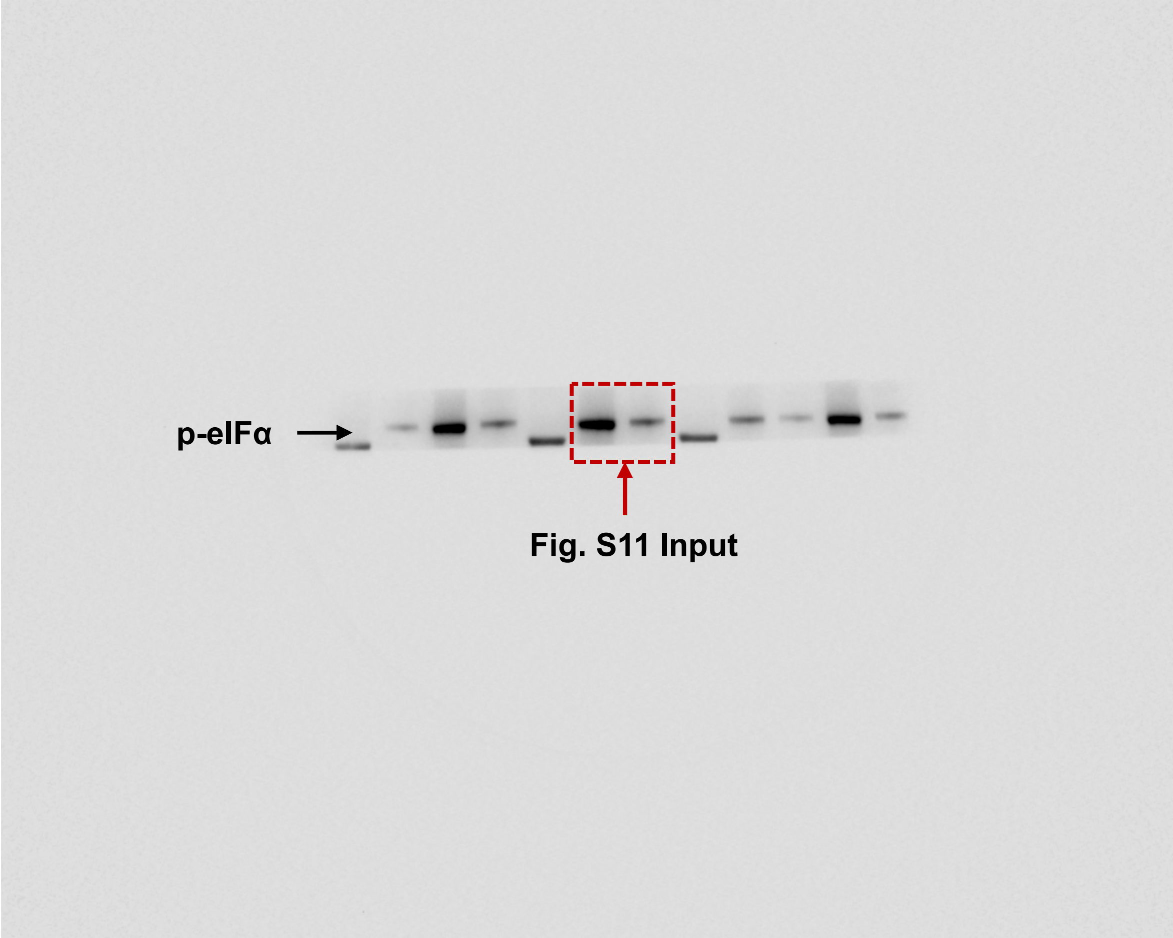

p-eIFα
Fig. S11 Input

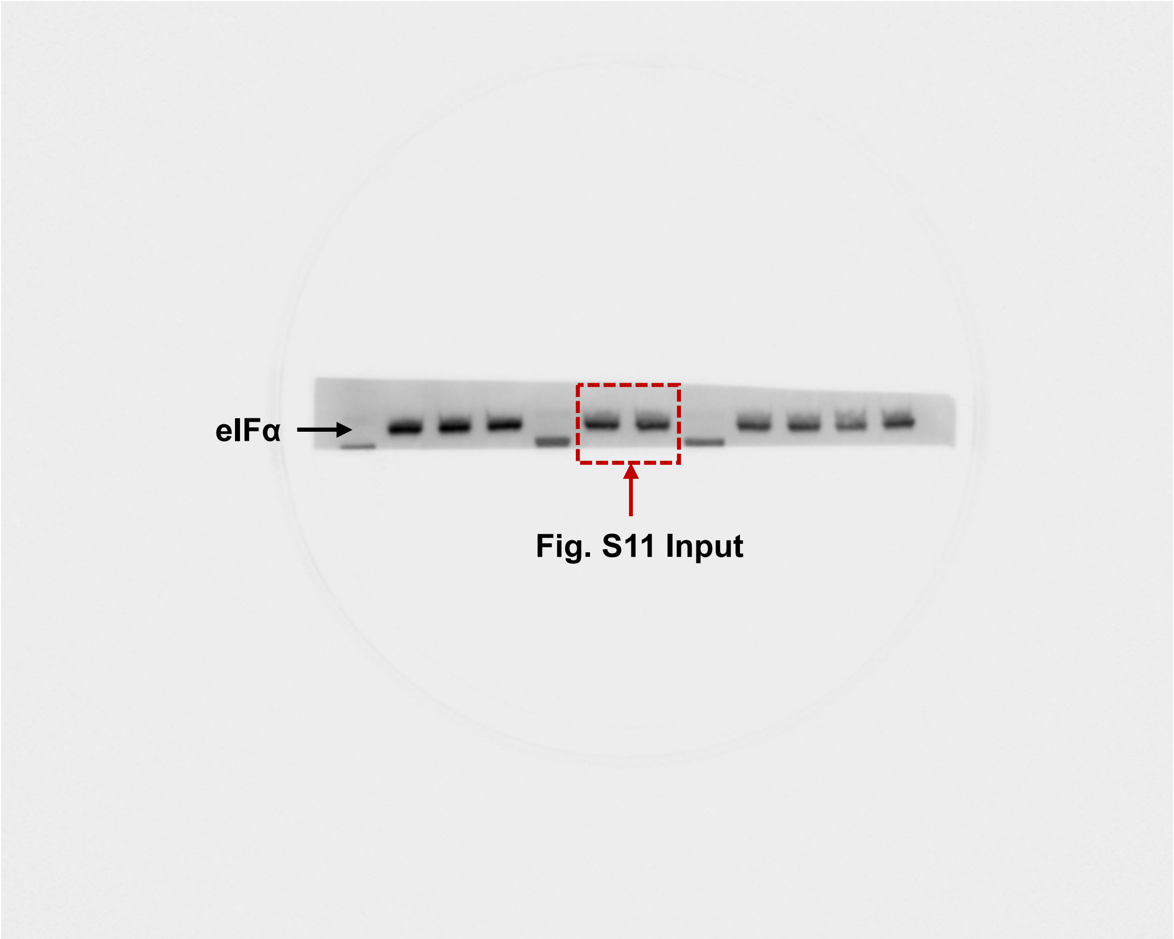
eIFα
Fig. S11 Input

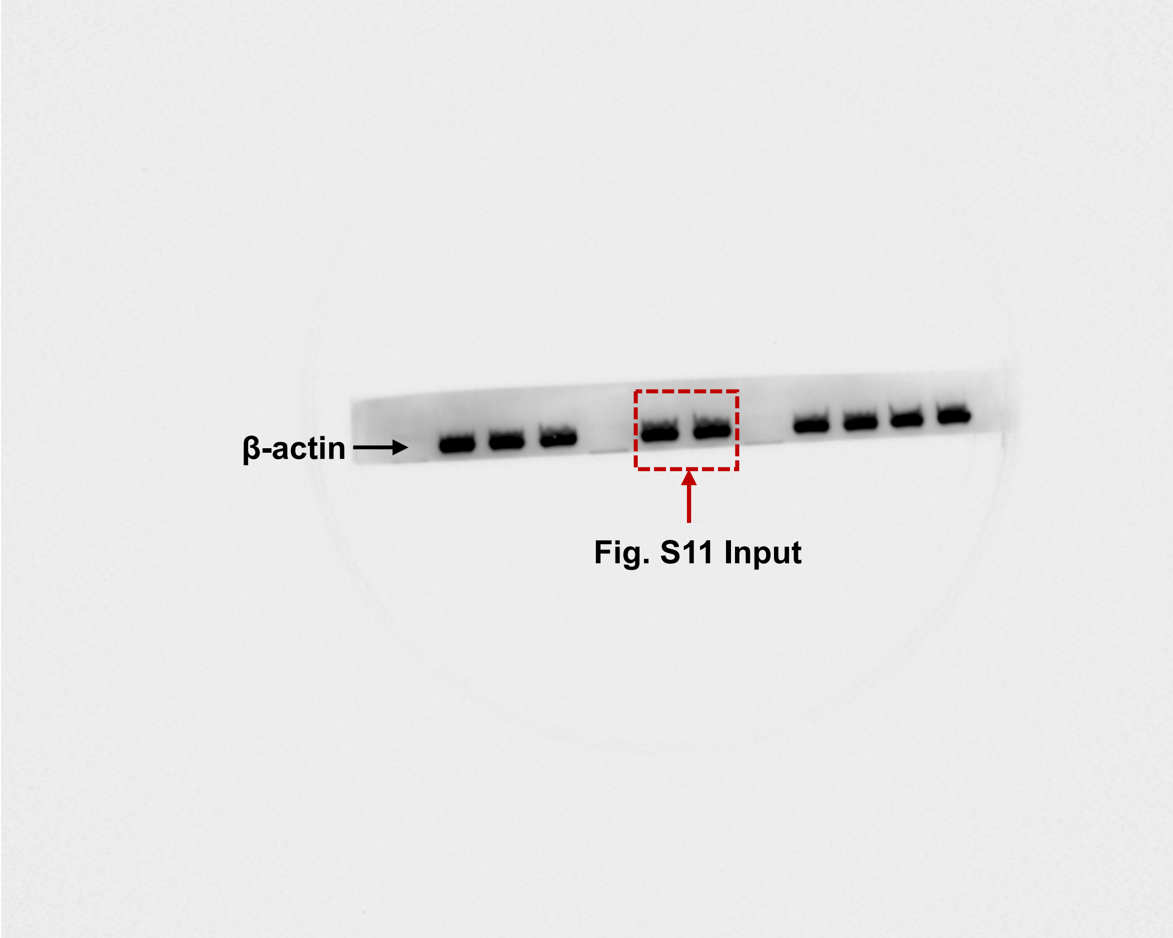
β-actin
Fig. S11 Input

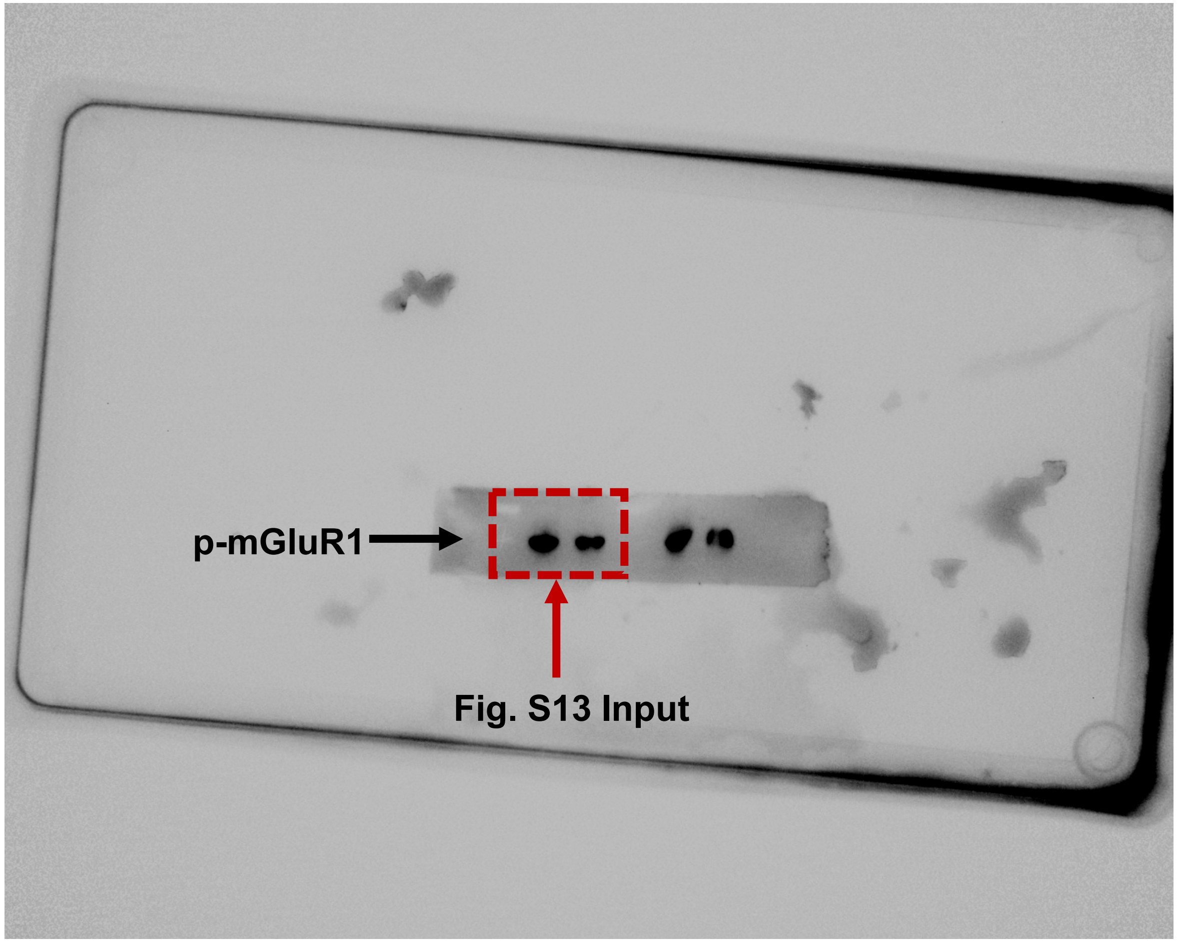
p-mGluR1
Fig. S13 Input

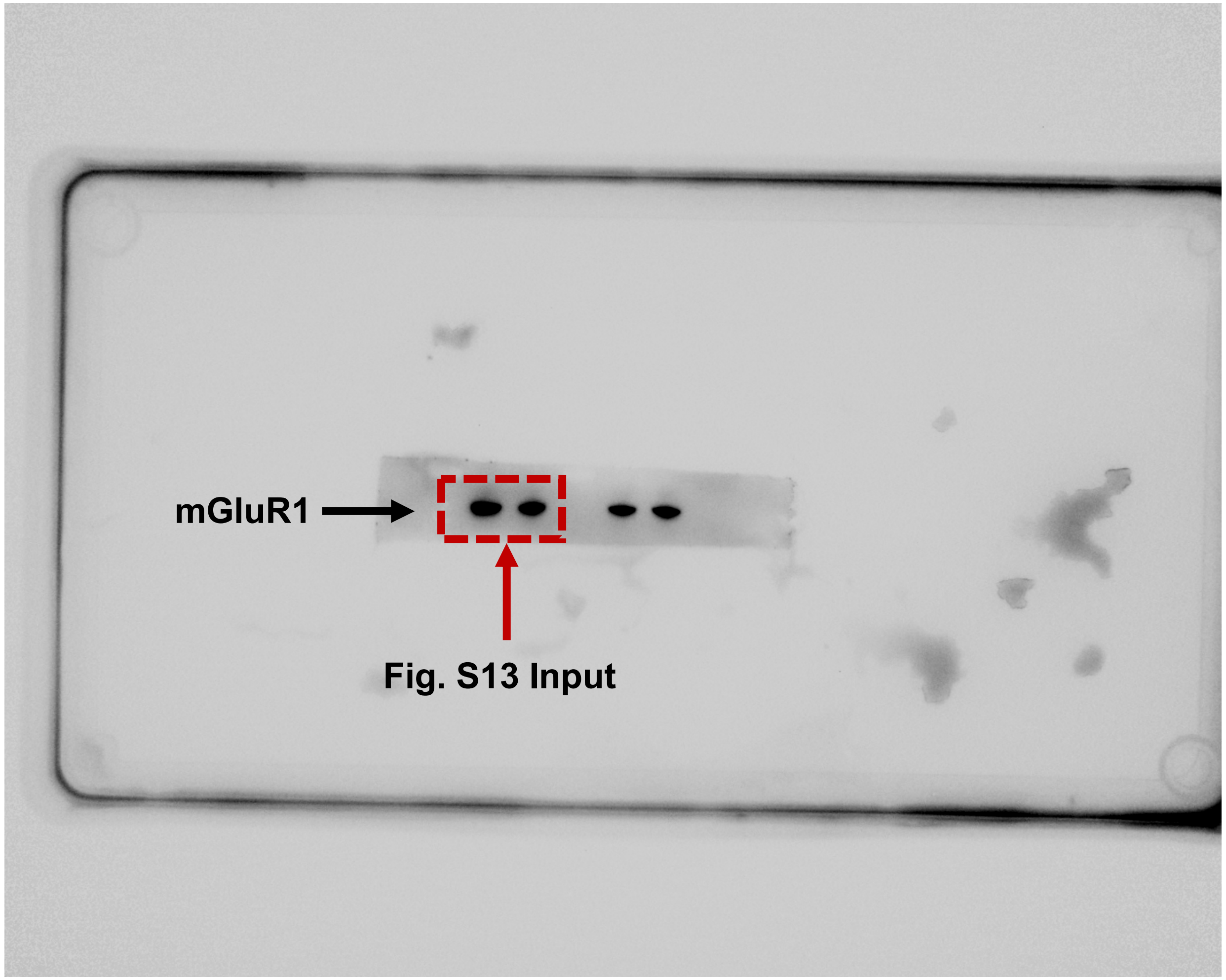
mGluR1
Fig. S13 Input

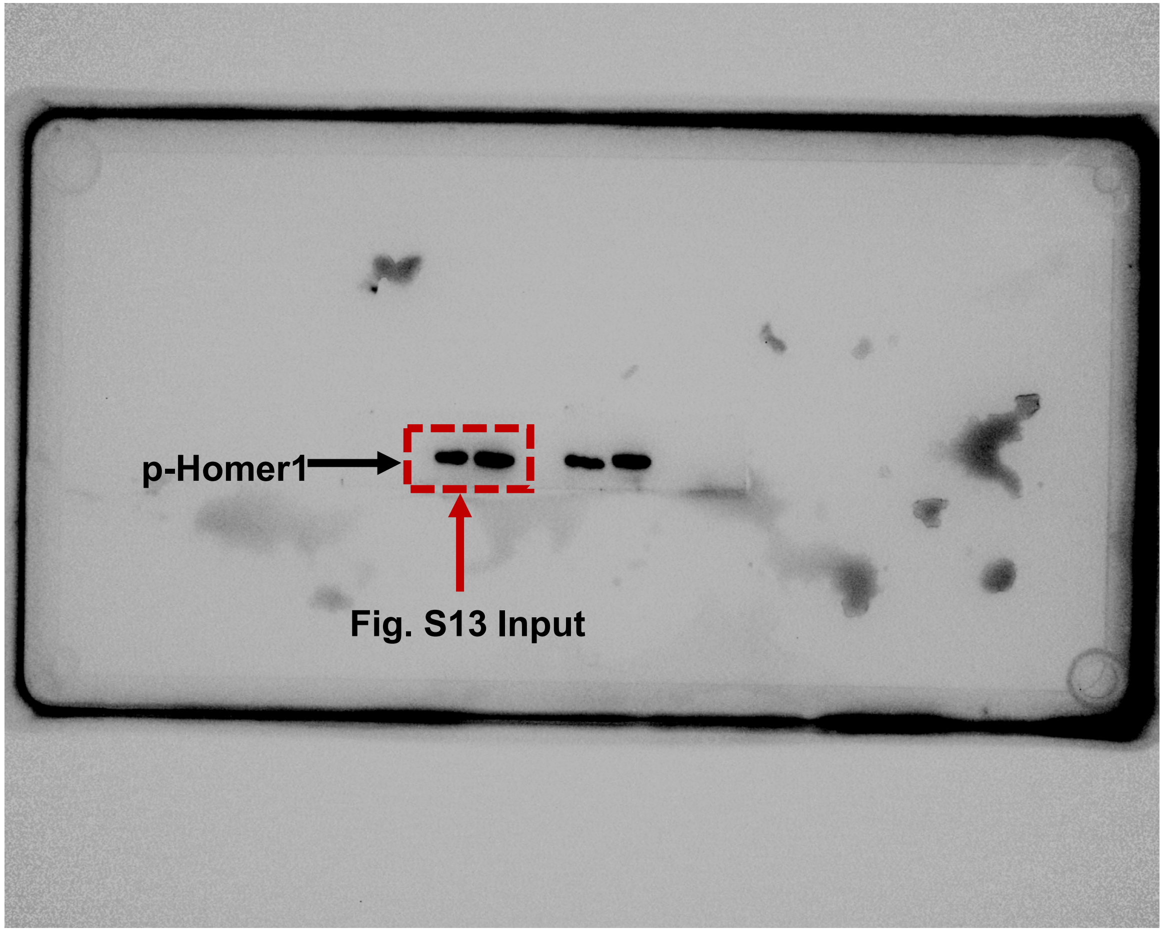
p-Homer1
Fig. S13 Input

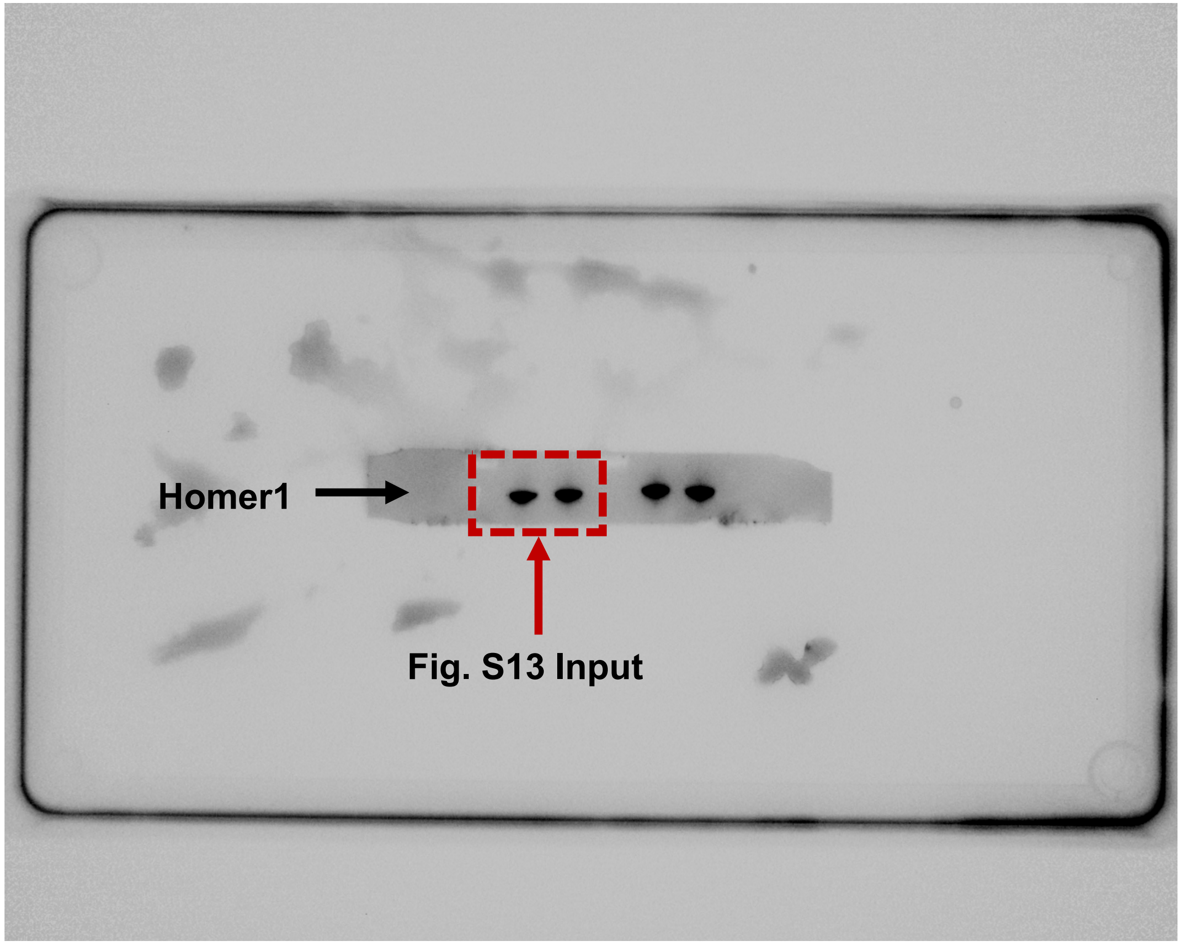
Homer1
Fig. S13 Input

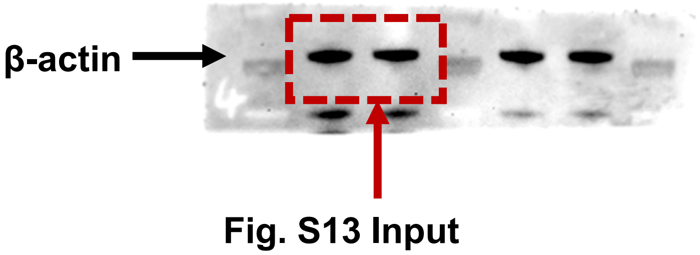
β-actin
Fig. S13 Input

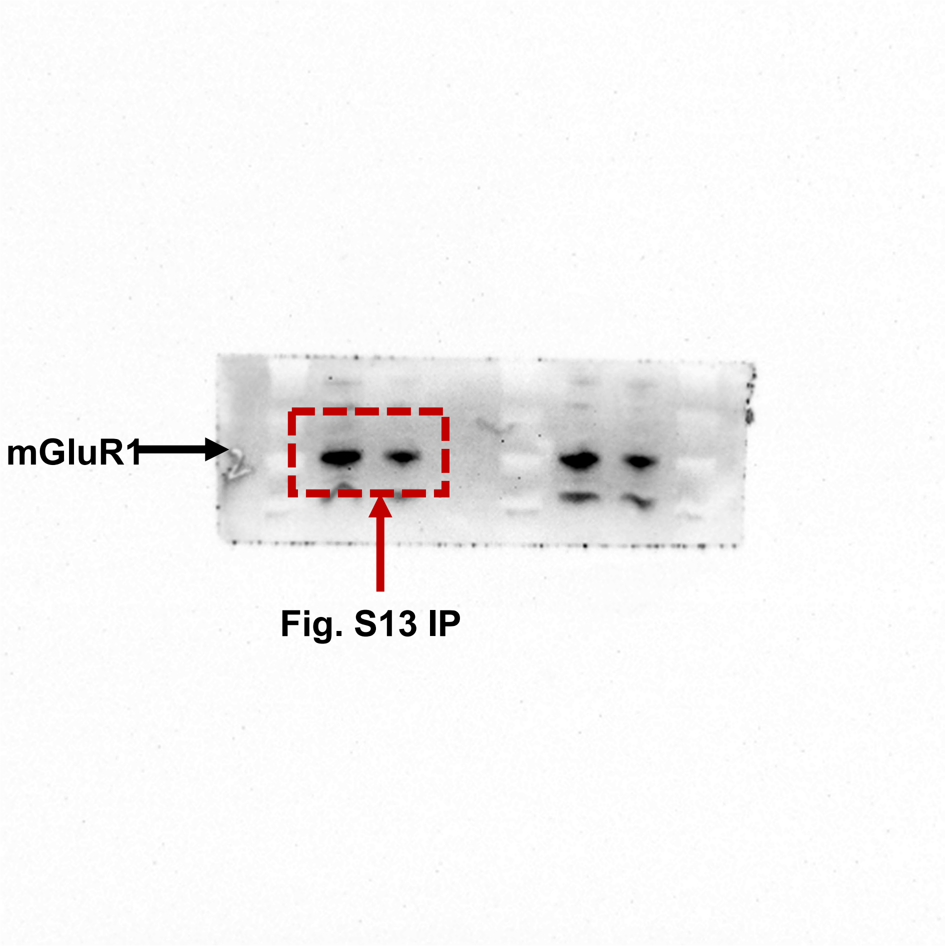
mGluR1
Fig. S13 IP

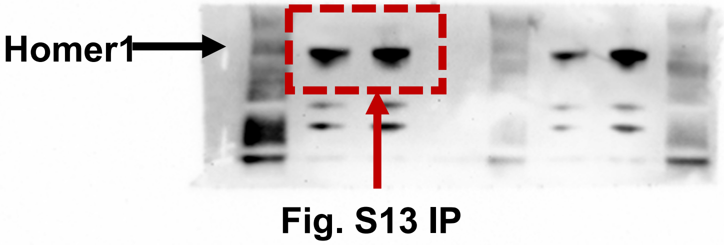
Homer1
Fig. S13 IP
